# Supramolecular Entrapping and Extraction of Selenate, Molybdate and Tungstate Ions from Water by Nanojars

**DOI:** 10.1021/acs.inorgchem.4c04544

**Published:** 2025-01-03

**Authors:** Wisam A. Al Isawi, Angel S. Philip, Pooja Singh, Matthias Zeller, Gellert Mezei

**Affiliations:** † Department of Chemistry, Western Michigan University, Kalamazoo, Michigan 49008, United States; ‡ Department of Chemistry, Purdue University, West Lafayette, Indiana 47907, United States

## Abstract

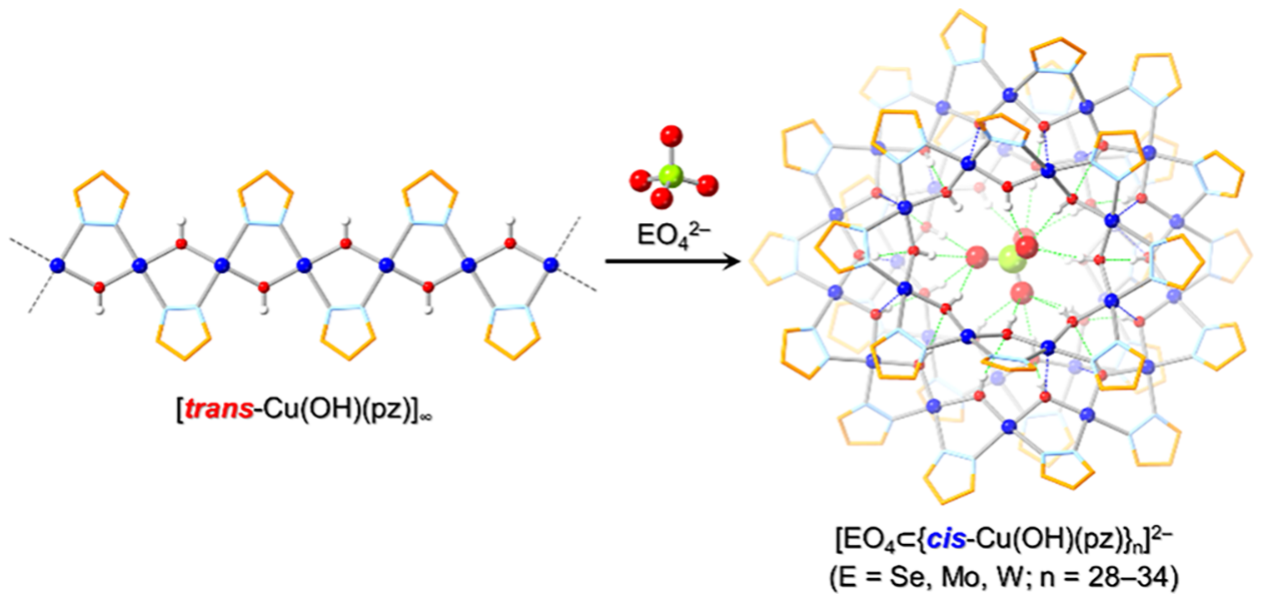

The supramolecular binding exclusively by H-bonds of SeO_4_^2–^, MoO_4_^2–^ and WO_4_^2–^ ions to form nanojars of the formula [EO_4_^2–^⊂{*cis*-Cu^II^(μ-OH)(μ-pz)}_*n*_]^2–^ (**Cu**_***n***_**EO**_**4**_; E = Se, Mo, W; *n* = 28–34; pz = pyrazolate) was studied in solution by electrospray ionization mass spectrometry, variable temperature, paramagnetic ^1^H NMR and UV–vis spectroscopy, and in the solid state by single-crystal X-ray crystallography. These large anions allow for the observation of a record nanojar size, **Cu**_**34**_**EO**_**4**_ (E = Mo, W). Six crystal structures are described of nanojars of varying sizes with either SeO_4_^2–^, MoO_4_^2–^ or WO_4_^2–^ entrapped ions, including the first example of a cocrystal of two different nanojars in crystallographically unique positions, **Cu**_**31**_**MoO**_**4**_ and **Cu**_**32**_**MoO**_**4**_. The latter provides unprecedented structural information about the Cu_8_+Cu_14_+Cu_10_ ring combination of a nanojar with an entrapped tetrahedral anion. Also, the first crystal structure of a supramolecular host–guest complex with an entrapped WO_4_^2–^ ion, **Cu**_**31**_**WO**_**4**_ is reported in this work. The relative strength of binding of SeO_4_^2–^, MoO_4_^2–^ and WO_4_^2–^ ions by nanojars of different sizes was assessed by reactivity studies toward Ba^2+^ ions and NH_3_. Thermal stability studies of the various **Cu**_**n**_**EO**_**4**_ nanojars were conducted in DMSO-*d*_6_ solutions over a 22–150 °C range. Furthermore, liquid–liquid extraction of SeO_4_^2–^, MoO_4_^2–^ and WO_4_^2–^ ions from water into an organic solvent by nanojars was investigated.

## Abstract 2

The binding of heavy metal oxoanions by nanojars was explored using mass spectrometry, variable temperature, paramagnetic ^1^H NMR and UV–vis spectroscopy, single-crystal X-ray crystallography and reactivity studies toward Ba^2+^ ions and NH_3_, along with the extraction of these anions from water into an organic solvent by liquid–liquid extraction.

## Introduction

Se, Mo and W are rare elements in Earth’s crust, with abundances of 0.05, 1.2, and 1.25 ppm, respectively,^1^ whereas in the human body their average amounts are 0.21, 0.07, and 0.0003 ppm.^2^ Molybdenum is an essential trace element for most life forms, including humans,^3−5^ whereas selenium is only essential for animals, humans and microorganisms (although it is beneficial for many plants),^6−9^ and tungsten is an essential trace element only for some bacteria and archaea.^10,11^ Although not directly involved in human metabolism, it has been recently shown that tungsten enzymes play a role in detoxifying food and antimicrobial aldehydes in the human gut microbiome.^12^ Molybdenum and tungsten enzymes also play an important role in the global biogeochemical cycles of nitrogen, carbon and sulfur.^13^ The principal sources of these elements are selenate (SeO_4_^2–^), molybdate (MoO_4_^2–^) and tungstate (WO_4_^2–^).^14^

In Nature, selenate, molybdate and tungstate minerals are rare except for scheelite (CaWO_4_) and wolframite (Fe/MnWO_4_), which are important ores of tungsten worldwide. They are mostly found as secondary minerals in the oxidized zone of a base-metal deposit.^15^ While rock weathering and volcanic eruptions can release Se, Mo and W into the surrounding environment, the most worrisome sources of pollution with these elements are anthropogenic activities, such as mining, industrial manufacturing and fossil fuel burning.^16−21^ While Mo and W are currently perceived as posing less risk,^22−25^ Se is very toxic in doses slightly higher than the recommended daily intake.^26−29^ Consequently, the maximum amount of Se in drinking water allowed by the U.S. Environmental Protection Agency is limited to 0.05 mg/L (50 ppb). No federal drinking water standard has yet been established for Mo and W, although it is being considered.^30^

Due to the environmental and health concerns caused by the accumulation of toxic or harmful oxoanions in certain water bodies, oxoanion recognition and extraction has been receiving increasing attention.^31−35^ Traditional selenate,^36−38^ molybdate,^39^ and tungstate^40^ removal methods employ flocculation/precipitation followed by sedimentation/filtration, and adsorption onto solid phases. Because these methods present disadvantages, especially when the recovery of the oxoanion is sought, alternative methods are also being considered. These include ionic exchange resins, metal–organic frameworks,^41−46^ crystallization,^47^ coprecipitation,^48^ and solvent extraction.^49^ An attractive approach is liquid–liquid extraction using supramolecular receptors, which offers advantages such as selectivity and recyclability, and avoids the generation of large amounts of contaminated byproducts.^50^ However, the supramolecular chemistry of many anions is still in its infancy. Only a few supramolecular receptor/SeO_4_^2–^ host–guest complexes have been structurally characterized to date, including a cryptand,^51^ tripodal urea-based capsules^52−55^ and urea-functionalized metal–organic cages.^56^ With MoO_4_^2–^, only one supramolecular host–guest structure has been reported,^56^ whereas with WO_4_^2–^ no examples are known. The rarity of receptors for tetrahedral MoO_4_^2–^ and WO_4_^2–^ might be attributable to the fact that these anions tend to form polyoxoanions under acidic conditions, and many supramolecular receptors are based on protonated amines.

**Scheme 1:**
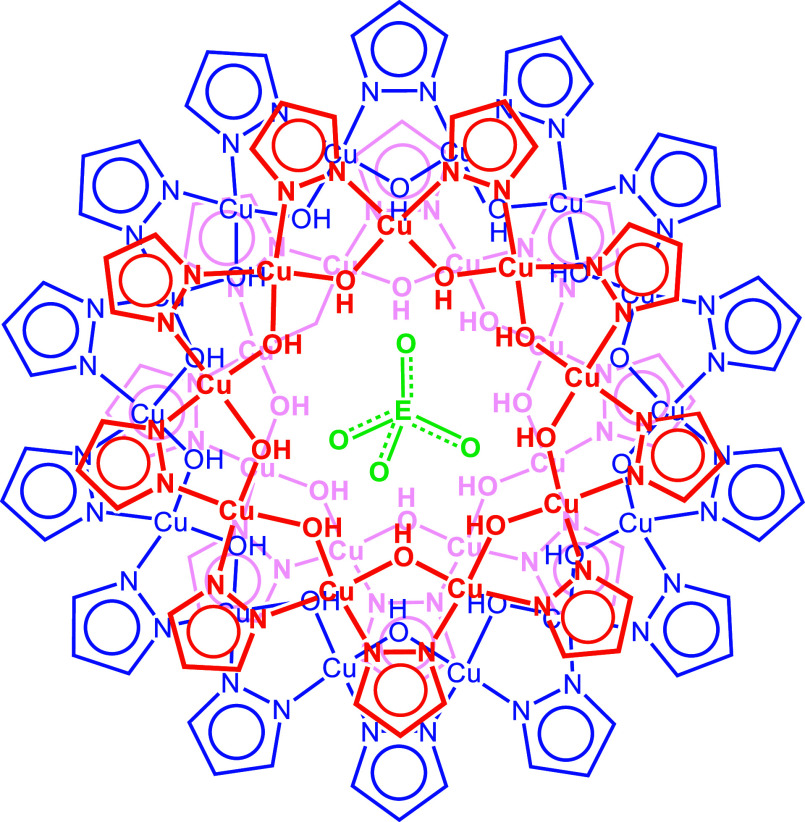
Schematic Representation of a Cu_31_ Nanojar, [EO_4_⊂{Cu(μ-OH)(μ-pz)}_8+14+9_]^2–^ (**Cu**_**31**_**EO**_**4**_; E = Se, Mo, W) Color code: persimmon, Cu_9_ ring; blue, Cu_14_ ring; pink, Cu_8_ ring.

**Figure 1:**
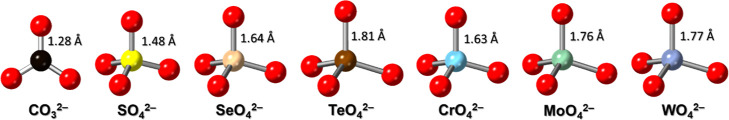
Structural representation of different oxoanions illustrating their average E–O distances (E = C, S, Se, Te, Cr, Mo, W) based on the Cambridge Structural Database.^63^

We have recently developed a class of neutral anion recognition and extraction agents termed nanojars, which consist of supramolecular coordination complexes based on the [*cis*-Cu^II^(μ-OH)(μ-pz)] repeating unit. Nanojars of the formula [anion⊂{*cis*-Cu^II^(μ-OH)(μ-pz)}_*n*_]^2–^ (**Cu**_**n**_**anion**; *n* = 27–33; pz = pyrazolate, C_3_H_3_N_2_^–^; Scheme 1) form by self-assembly around the target anion guest from Cu^2+^ ions and pyrazole (Hpz) in the presence of a base, and offer total selectivity for anions with large hydration energies over anions with small hydration energies. Indeed, we demonstrated the extraction of 2– charged anions such as CO_3_^2–^ (Δ*G*_h_^°^ = −1315 kJ/mol),^57,58^ SO_4_^2–^ (Δ*G*_h_^°^ = −1080 kJ/mol)^59,60^ and CrO_4_^2–^ (Δ*G*_h_^°^ = −950 kJ/mol)^61^ from water into organic solvents by nanojars in the presence of excess 1– charged anions including NO_3_^–^ (Δ*G*_h_^°^ = −300 kJ/mol), ClO_4_^–^ (Δ*G*_h_^°^ = −430 kJ/mol) and halides (Δ*G*_h_^°^ = −275 to −465 kJ/mol).^62^ In this work, we explore the supramolecular binding and extraction from water of even larger oxoanion analogues, SeO_4_^2–^, TeO_4_^2–^, MoO_4_^2–^ and WO_4_^2–^, using nanojars (Figure 1).

## Results and Discussion

### Synthesis and Mass Spectrometric Studies

**Figure 2:**
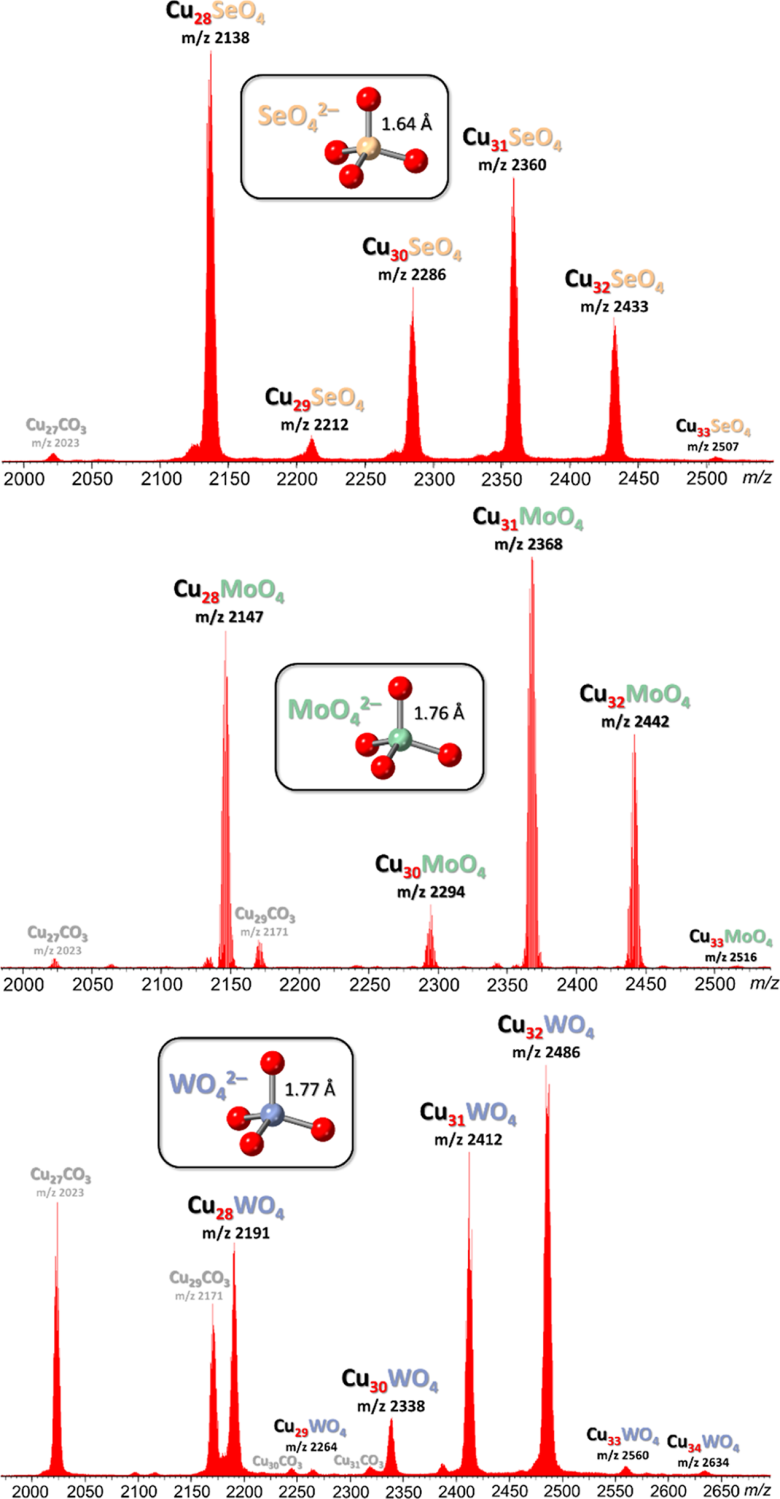
ESI-MS(−) spectra (in CH_3_CN) of the as-synthesized selenate-, molybdate- and tungstate-entrapping nanojar mixtures [EO_4_⊂{Cu(OH)(pz)}_*n*_]^2–^ (**Cu**_**n**_**EO**_**4**_; E = Se, Mo, W; *n* = 28–34). *m*/*z* values indicated are averages of isotopic distributions (detailed isotopic distributions are shown in Figure S11).

Selenate-, molybdate- and tungstate-entrapping nanojar mixtures were obtained by the anion-induced depolymerization of [*trans*-Cu^II^(μ-OH)(μ-pz)]_∞_. This polymer combines not only the Cu^2+^ source and the base, but also the deprotonated pyrazolate ion thereby eliminating the need for any additional base in the reaction. Refluxing with *in situ* prepared (Bu_4_N)_2_SeO_4_ in toluene (bp 111 °C) turns [*trans*-Cu^II^(μ-OH)(μ-pz)]_∞_ into [SeO_4_⊂{*cis*-Cu^II^(μ-OH)(μ-pz)}_*n*_]^2–^ (**Cu**_**n**_**SeO**_**4**_), in which only a trace of **Cu**_**27**_**CO**_**3**_ is detected. ESI-MS(−) analysis of the product shows that the obtained **Cu**_**n**_**SeO**_**4**_ mixture contains **Cu**_**28**_**SeO**_**4**_ (*m*/*z* 2138), **Cu**_**30**_**SeO**_**4**_ (*m*/*z* 2286), **Cu**_**31**_**SeO**_**4**_ (*m*/*z* 2360) and **Cu**_**32**_**SeO**_**4**_ (*m*/*z* 2433) as major species, along with a small amount of **Cu**_**29**_**SeO**_**4**_ (*m*/*z* 2212) and a trace of **Cu**_**33**_**SeO**_**4**_ (*m*/*z* 2507) (Figure 2). When chlorobenzene (bp 132 °C) is used instead of toluene as solvent, a carbonate-free **Cu**_**n**_**SeO**_**4**_ mixture is obtained in which **Cu**_**31**_**SeO**_**4**_ predominates (Figure S12). When excess (Bu_4_N)_2_SeO_4_ is used, substituted nanojar species also form, in which one or two pz^–^ units are apparently replaced by selenate (tentative assignments are provided in Table S1 and spectra are shown in Figure S13). Analogous depolymerization reactions using *in situ* prepared (Bu_4_N)_2_EO_4_ (E = Mo and W) also yielded an almost carbonate-free mixture of **Cu**_**n**_**MoO**_**4**_ (*n* = 28, *m*/*z* 2147; *n* = 30, *m*/*z* 2294; *n* = 31, *m*/*z* 2368; *n* = 32, *m*/*z* 2442; traces of *n* = 33, *m*/*z* 2516 and *n* = 34, *m*/*z* 2590), whereas the mixture of **Cu**_***n***_**WO**_**4**_ (*n* = 28, *m*/*z* 2191; *n* = 30, *m*/*z* 2338; *n* = 31, *m*/*z* 2412; *n* = 32, *m*/*z* 2486; traces of *n* = 29, *m*/*z* 2264, *n* = 33, *m*/*z* 2560 and *n* = 34, *m*/*z* 2634) contains significant amounts of **Cu**_***n***_**CO**_**3**_ (*n* = 27, 29–31) (Figure 2). Occasionally, 1– charged adducts such as [(Bu_4_N)EO_4_^2–^⊂{Cu(OH)(pz)}_*n*_]^1–^ are observed, but only in trace amounts. Because the solvent used for the mass spectrometric characterization (CH_3_CN) is highly polar, the two Bu_4_N^+^ countercations are dissociated from the 2– charged nanojars.

While the reaction of CuSO_4_, pyrazole, NaOH and Bu_4_NOH (1:1:1.93:0.07 molar ratio) in tetrahydrofuran (THF) produces a clean **Cu**_***n***_**SO**_**4**_ (*n* = 27–33) nanojar mixture,^64^ an analogous reaction with CuSeO_4_ under identical conditions yields a **Cu**_***n***_**SeO**_**4**_ (*n* = 27–33) nanojar mixture mixed with **Cu**_***n***_**CO**_**3**_ (*n* = 27, 29, 31) impurities (Figure S1). Various different reaction conditions, as detailed in the Supporting Information, failed to provide a carbonate-free **Cu**_***n***_**SeO**_**4**_ mixture by self-assembly (Figures S2–S10). With MoO_4_^2–^ and WO_4_^2–^, similar interference from carbonate was observed when attempting to synthesize the corresponding nanojars directly by self-assembly. Figures S14–S20 illustrate the results of the reactions yielding CO_3_-contaminated **Cu**_***n***_**MoO**_**4**_ and **Cu**_***n***_**WO**_**4**_, respectively. In the case of tungstate when Et_3_N is used as the base (which produces acidic Et_3_NH^+^ as a byproduct), the Lindqvist hexatungstate W_6_O_19_^2–^ is also obtained (Figure S20).^65^

Since bases are used in the syntheses by self-assembly, the presence of CO_2_ in air can be detrimental. To put this in perspective, a nanojar synthesis reaction ran with 100 mg of Cu(NO_3_)_2_·2.5H_2_O needs 0.652 mg of CO_2_ to form [CO_3_⊂{Cu(OH)(pz)}_*n*_]^2–^ exclusively. This amount of CO_2_ is contained in ∼0.8 L of pure air (∼0.04% CO_2_) or ∼8 mL of air exhaled by a person (∼4% CO_2_). In other words, one single breath of exhaled air (∼500 mL) contains more CO_2_ than needed to obtain pure carbonate nanojars. The concentration of CO_2_ in the air can be further exacerbated by the presence of dry ice in a nearby open container.

With CrO_4_^2–^, a virtually carbonate-free **Cu**_***n***_**CrO**_**4**_ nanojar mixture could be obtained under an N_2_ atmosphere.^61^ With the larger anions, however, the concomitant formation of **Cu**_***n***_**CO**_**3**_ could not be avoided using a CO_2_-free atmosphere even in the presence of excess SeO_4_^2–^, MoO_4_^2–^ and WO_4_^2–^ ions. This suggests that carbonate is originating from the reagents used. Fresh ACS grade NaOH, for example, can contain up to 1% Na_2_CO_3_, which already provides 22% of the carbonate needed for the formation of pure **Cu**_**n**_**CO**_**3**_. In the other reagents used, carbonate is not quantitated. An increasing amount of **Cu**_***n***_**CO**_**3**_ impurity is observed as the size of the intended anion guest becomes larger, indicating a lesser preference of nanojars for these anions in the presence of competing carbonate.

**Cu**_***n***_**MoO**_**4**_ and **Cu**_***n***_**WO**_**4**_ nanojars were also obtained inadvertently when the binding of the thio-derivatives MoS_4_^2–^ and WS_4_^2–^ by nanojars was attempted. During the reaction, the tetrathiomolybdate/tungstate species hydrolyzed giving rise to the corresponding oxoanions and sulfide, accompanied by the partial oxidation of S^2–^ to SO_4_^2–^ which is also entrapped by nanojars as **Cu**_**31**_**SO**_**4**_ (Figures S21 and S22). Moreover, **Cu**_***n***_**MoO**_**4**_ (*n* = 28, 31, 32) was also obtained from the reaction of [Mo^VI^_8_O_12_(μ-O)_9_(μ-pz)_6_(pzH)_6_·3pzH] with Cu(NO_3_)_2_ and Bu_4_NOH in THF (Figure S23).^66^

The intensity of the peaks corresponding to [SeO_4_⊂{Cu(OH)(pz)}_*n*_]^2–^ (*n* = 28–32) in the ESI-MS(−) spectrum increases when the sampling cone voltage is increased from 0 to ∼40 V. At higher voltages, these peaks in the *m*/*z* 2100–2500 window gradually disappear, while a new set of peaks appears in the *m*/*z* 1650–2055 window (Figure S24). At 100 V, only these new peaks persist, along with a peak at *m*/*z* 198 corresponding to [Cu^I^(pz)_2_]^−^. The new set of peaks was tentatively assigned to nanojar daughter species of the general formula [SeO_4_⊂{Cu_*n*–3_O_(n–y+2)/2_(pz)_*n*+*y*−8_}]^2–^ [*y* = (−2)–6; *y* has the same parity as *n*], which form when nanojars shrink by losing neutral Cu_3_(OH)_6_(Hpz)_8–y_(H_2_O)_(*n*+*y*−14)/2_ fragments, similarly to the sulfate analogues.^64^ Thus, three or four daughter species are observed for each **Cu**_***n***_**SeO**_**4**_ nanojar (Table S2). These proposed formulas are not intended to imply particular structures, as the exact structures of these daughter species are unknown. Analogous shrunken nanojar species are also observed with molybdate and tungstate as well, along with derivatives in the case of the Cu_32_ nanojar wherein one pz^–^ unit is replaced by HO^–^ (Table S3 and Figures S25–S28). In contrast, the carbonate nanojars present in the sample lose Cu_5_(OH)_10_(Hpz)_10–*y*_(H_2_O)_(*n*+*y*−20)/2_ fragments (*y* = 3–11) and produce shrunken nanojars of the formula [Cu_n–5_O_(*n*–*y*)/2_(pz)_*n*+*y*−10_CO_3_]^2–^.^64^ These 100 V spectra are especially useful for differentiating between possibly overlapping peaks and avoiding erroneous assignments in the corresponding 40 V spectra. For example, **Cu**_**31**_**SO**_**4**_ and **Cu**_**30**_**WO**_**4**_ nanojars have very similar *m*/*z* values (2336 vs 2338). In contrast, daughter nanojars of **Cu**_**31**_**SO**_**4**_ have completely different *m*/*z* values (1871, 1930, and 1989 for *y* = 1, 3 and 5) from **Cu**_**30**_**WO**_**4**_ (1848, 1907, and 1966 for *y* = 0, 2 and 4).

No analogous nanojars could be obtained with the TeO_4_^2–^ ion (Figure S29). Telluric acid does not exist as H_2_TeO_4_, but as Te(OH)_6_ (H_2_TeO_4_ + 2H_2_O ⇌ H_6_TeO_6_), and most tellurates also contain hexacoordinated tellurium. Yet, examples of individual, tetrahedral TeO_4_^2–^ ions in crystal structures are known, including Rb_6_(TeO_4_)(TeO_5_)^67^ and (Et_4_N)_2_TeO_4_·2H_2_O.^68^ As opposed to tetrahedral tellurate in which the Te–O bonds have partial double-bond character and an average length of 1.81 Å, in octahedral tellurate all six Te–O bonds are single bonds, with an average length of 1.93 Å.

### Fractionation of the Cu_*n*_SeO_4_ Nanojar Mixture

The different solubility of the various **Cu**_***n***_**SeO**_**4**_ nanojars in toluene allows for the separation of **Cu**_**28**_**SeO**_**4**_ from the other species. Specifically, after the **Cu**_***n***_**SeO**_**4**_ (*n* = 28–32) mixture is dissolved in the minimum amount of toluene at ambient temperature, **Cu**_**28**_**SeO**_**4**_ starts precipitating out leaving the nanojars of other sizes (*n* = 29–32) in solution. Figure S30 shows the composition of the two fractions as indicated by their corresponding ESI-MS spectra.

### Etching of the **Cu**_***n***_**EO**_**4**_ (E = Se, Mo, W) Nanojar Mixtures with NH_3_

Treatment of a **Cu**_***n***_**SeO**_**4**_ (*n* = 28–33) nanojar mixture with an excess of gaseous ammonia in THF solution reveals that only **Cu**_**31**_**SeO**_**4**_ and **Cu**_**32**_**SeO**_**4**_ survive the effect of the strongly coordinating NH_3_, which breaks up the smaller (*n* = 28–30) and larger (*n* = 33) nanojars and cleanly converts them into the most stable nanojars with the SeO_4_^2–^ ion. By contrast, all **Cu**_***n***_**CO**_**3**_ (*n* = 27, 29–31) species convert into the smallest nanojar, **Cu**_**27**_**CO**_**3**_, which is the most stable one with the smaller CO_3_^2–^ ion in the presence of NH_3_ (Figure S31). With the larger MoO_4_^2–^ and WO_4_^2–^ ions, a similar conversion is observed as with SeO_4_^2–^: after 1 day of NH_3_ treatment, only the Cu_31_ and Cu_32_ species remain. Unlike with SeO_4_^2–^, however, the Cu_31_ species only persist for about a week and eventually completely convert into Cu_32_ after standing for 10 weeks in the NH_3_-saturated solution (Figures S32 and S33). This observation reflects the most favorable binding of the larger EO_4_^2–^ (E = Mo and W) ions by the Cu_32_ nanojar.

### Structural Analysis by X-ray Crystallography

To obtain structural details of the nanojar hosts and the supramolecular binding of the SeO_4_^2–^, MoO_4_^2–^ and WO_4_^2–^ ions within their cavities lined with H-bond donor OH groups, the crystal structures of (Bu_4_N)_2_[SeO_4_⊂{Cu(OH)(pz)}_8+14+9_] (**1**, from chlorobenzene/*n*-pentane), (Bu_4_N)_2_[SeO_4_⊂{Cu(OH)(pz)}_9+14+9_] (**2**, from 1,2-dichlorobenzene/*n*-heptane), (Bu_4_N)_2_[MoO_4_⊂{Cu(OH)(pz)}_28_] (**3**, from nitrobenzene/bromobenzene/*n*-pentane with tri(benzyl)methylammonium nitrate as an additive), (Bu_4_N)_2_[MoO_4_⊂{Cu(OH)(pz)}_31_] (**4**, from chlorobenzene/hexanes with tri(benzyl)methylammonium nitrate as an additive), (Bu_4_N)(MePh_3_P)_4_[MoO_4_⊂{Cu(OH)(pz)}_8+14+9_][MoO_4_⊂{Cu(OH)(pz)}_8+14+10_]Br_0.48_(NO_3_)_0.52_ (**5**, from 1,2-dichlorobenzene/hexanes with tri(benzyl)methylammonium nitrate and methyltri(phenyl)phosphonium bromide as additives) and (Bu_4_N)_2_[WO_4_⊂{Cu(OH)(pz)}_31_] (**6**, from chlorobenzene/hexanes) were studied using single-crystal X-ray diffraction (Figures S40–S45 and Tables S4–S25).

**Figure 3:**
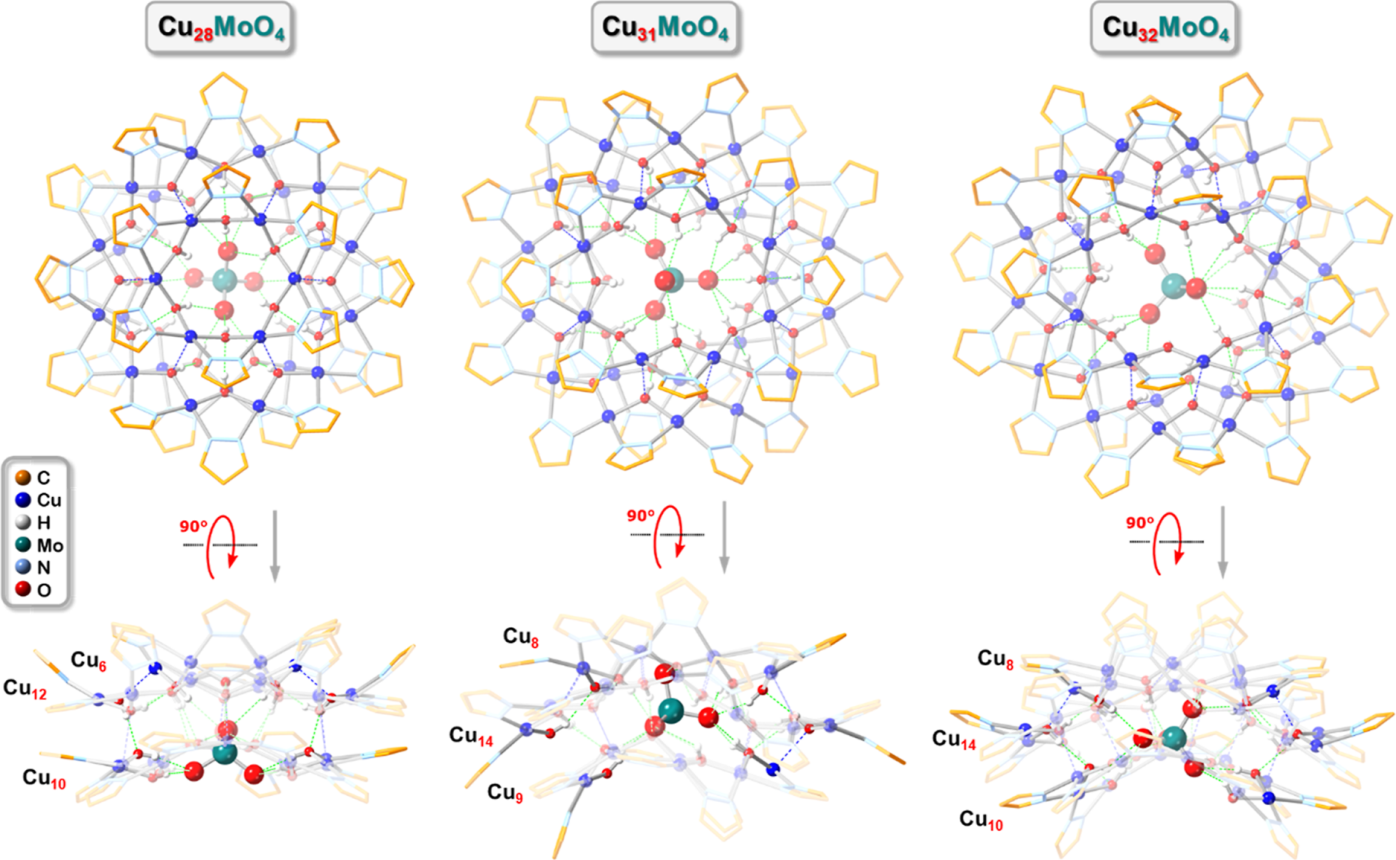
Ball-and-stick representation of the crystal structures of **Cu**_**28**_**MoO**_**4**_ (**3**), **Cu**_**31**_**MoO**_**4**_ (**5**) and **Cu**_**32**_**MoO**_**4**_ (**5**) (top- and side-views). Green and blue dotted lines indicate hydrogen bonds and axial Cu···O interactions, respectively. Counterions, lattice solvent molecules and C–H bond H atoms are omitted for clarity, and only the major component is shown for disordered moieties.

**Figure 4:**
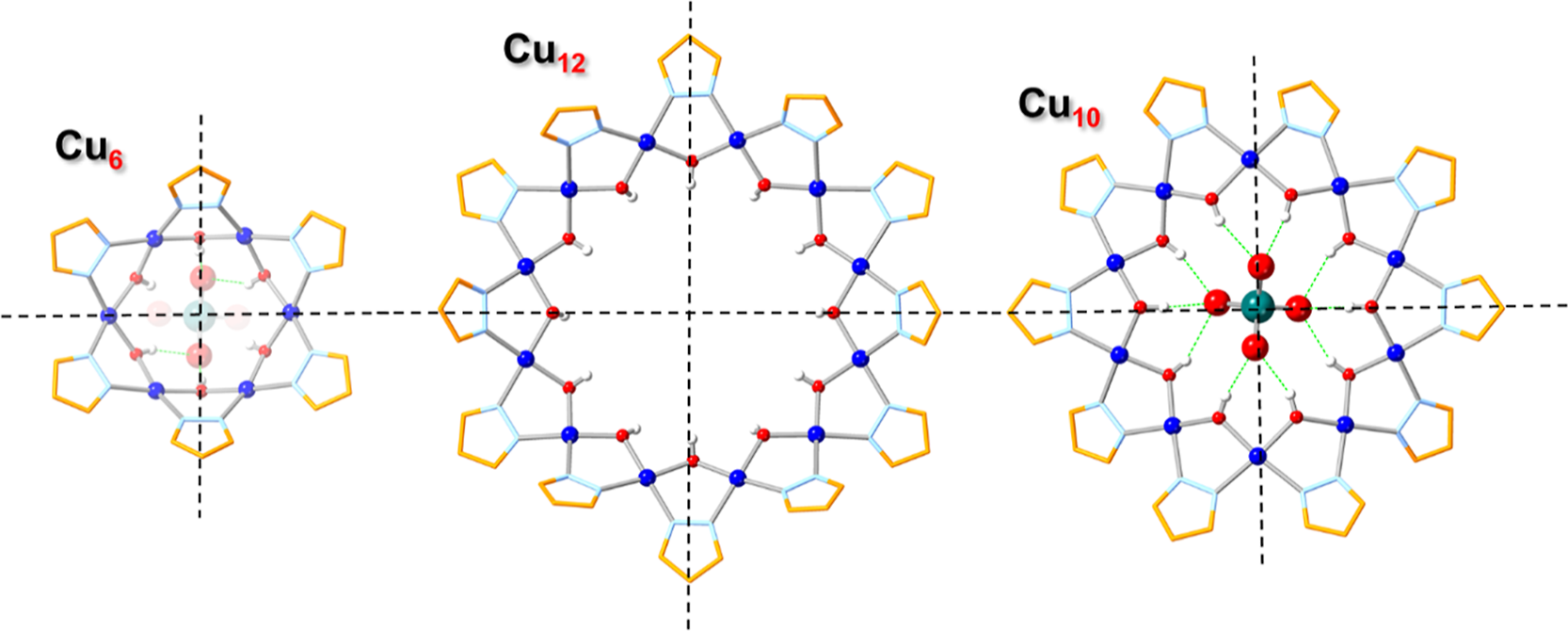
Illustration of the pseudomirror-planes bisecting the Cu_6_, Cu_12_ and Cu_10_ rings in **Cu**_**28**_**MoO**_**4**_ (**3**), as well as the hydrogen bonding pattern (green dashed lines) between MoO_4_^2–^ and the Cu_6_ and Cu_10_ rings.

Single-crystals of **Cu**_**28**_**EO**_**4**_ (E = Se, Mo or W) appropriate for X-ray diffraction could only be obtained in the case of MoO_4_^2–^ (Figure 3). As opposed to the triclinic (*P*1̅) crystal lattices of the previously characterized SO_4_^2–^, CrO_4_^2–^ and BeF_4_^2–^ analogues,^59,61,69^ (Bu_4_N)_2_[MoO_4_⊂{*cis*-Cu^II^(μ-OH)(μ-pz)}_6+12+10_] (**Cu**_**28**_**MoO**_**4**_; **3**) crystallizes in the monoclinic space group *P*2_1_/*n* (Figure 4). As shown in Figure S46, one of the two Bu_4_N^+^ counterions is in a rather different position next to the Cu_10_ ring of those nanojars, whereas the position of the other one (next to the Cu_6_ ring) is nearly unchanged in the three structures. In the case of **Cu**_**28**_**MoO**_**4**_ an additional, partially occupied H_2_O solvent molecule is also present, which is hydrogen-bonded to the Cu_10_ ring [O37···O21:2.766(10) Å; H37A···O21:2.00(11) Å; O37–H37A···O21:153(6)°] (Figure S47).

The altered position of the Bu_4_N^+^ counterions relative to the nanojar units might explain the different unit cells of the Cu_28_ nanojars with MoO_4_^2–^, CrO_4_^2–^, SO_4_^2–^ and BeF_4_^2–^ ions, which have very similar overall structures with only minor conformational variations of the Cu_*x*_ rings. The different solvents used for the crystallization of the **Cu**_**28**_**EO**_**4**_ (E = S, Cr and Mo) and **Cu**_**28**_**BeF**_**4**_ nanojars does not appear to be causing the different crystal symmetry observed for **Cu**_**28**_**MoO**_**4**_ (**3**), since isomorphous crystal structures were obtained in the case of **Cu**_**28**_**CrO**_**4**_ (chlorobenzene/*n*-heptane) and **Cu**_**28**_**BeF**_**4**_ (nitrobenzene/*n*-pentane),^61,70^ as well as **Cu**_**28**_**SO**_**4**_ (toluene/hexanes) and **Cu**_**28**_**BeF**_**4**_ (chlorobenzene/*n*-pentane).^59,70^

While the average Cu–O (1.93 Å) and Cu–N (1.97 Å) bond lengths, as well as the average *trans* (172°) and *cis* (86°) N–Cu–O angles within the **Cu**_**28**_**EO**_**4**_ (E = S, Cr and Mo) nanojars are virtually identical, small differences in their supramolecular structures are observed (Tables S6 and S7). Thus, the average of axial Cu···O distances between individual Cu_*x*_ rings shorter than the sum of the van der Waals radii of Cu and O (2.92 Å) increases from 2.486(3) and 2.478(3) Å for **Cu**_**28**_**SO**_**4**_ and **Cu**_**28**_**CrO**_**4**_, respectively, to 2.501(2) Å in the case of **Cu**_**28**_**MoO**_**4**_ (six between the Cu_6_ and Cu_12_ rings in each case, but only four between Cu_10_ and Cu_12_ rings in the case of **Cu**_**28**_**SO**_**4**_ as opposed to five in the case of **Cu**_**28**_**CrO**_**4**_ and **Cu**_**28**_**MoO**_**4**_). Similarly, the average of O–H···O hydrogen bonding distances (with D···A separations shorter than 3.2 Å) between Cu_*x*_ rings increases from 2.764(6) Å for **Cu**_**28**_**SO**_**4**_ to 2.789(4) Å for **Cu**_**28**_**CrO**_**4**_ and 2.791(3) Å for **Cu**_**28**_**MoO**_**4**_ (six between the Cu_6_ and Cu_12_ rings and six between the Cu_10_ and Cu_12_ rings in each case). The average of Cu···Cu distances also increases from 3.281(1) Å for **Cu**_**28**_**SO**_**4**_ to 3.288(1) Å for **Cu**_**28**_**CrO**_**4**_ to 3.298(1) Å for **Cu**_**28**_**MoO**_**4**_, indicating a slight expansion of the nanojar host as it accommodates increasingly larger guest molecules. The average O–H···O hydrogen bonding distance between nanojar host and anion guest is 2.92(2) Å in **Cu**_**28**_**SO**_**4**_, 2.920(6) Å in **Cu**_**28**_**CrO**_**4**_ and 2.936(4) Å in **Cu**_**28**_**MoO**_**4**_, while the number of corresponding H-bonds (with O···O separation shorter than 3.2 Å) increases from 12 to 14 and to 15, respectively. The hydrogen-bonding pattern with the three anions is rather different. With both SO_4_^2–^ and CrO_4_^2–^, one O atom forms six H-bonds with the Cu_6_ ring, whereas the other three O atoms form either two H-bonds each (SO_4_^2–^) or three H-bonds each (CrO_4_^2–^) with the Cu_10_ ring (Figure S48). In **3**, the MoO_4_^2–^ ion has a different orientation within the nanojar cavity, so that two of its O atoms form two H-bonds each with the Cu_6_ and Cu_10_ rings, while the other two form three H-bonds each with the Cu_10_ ring (Figure S49).

**Figure 5:**
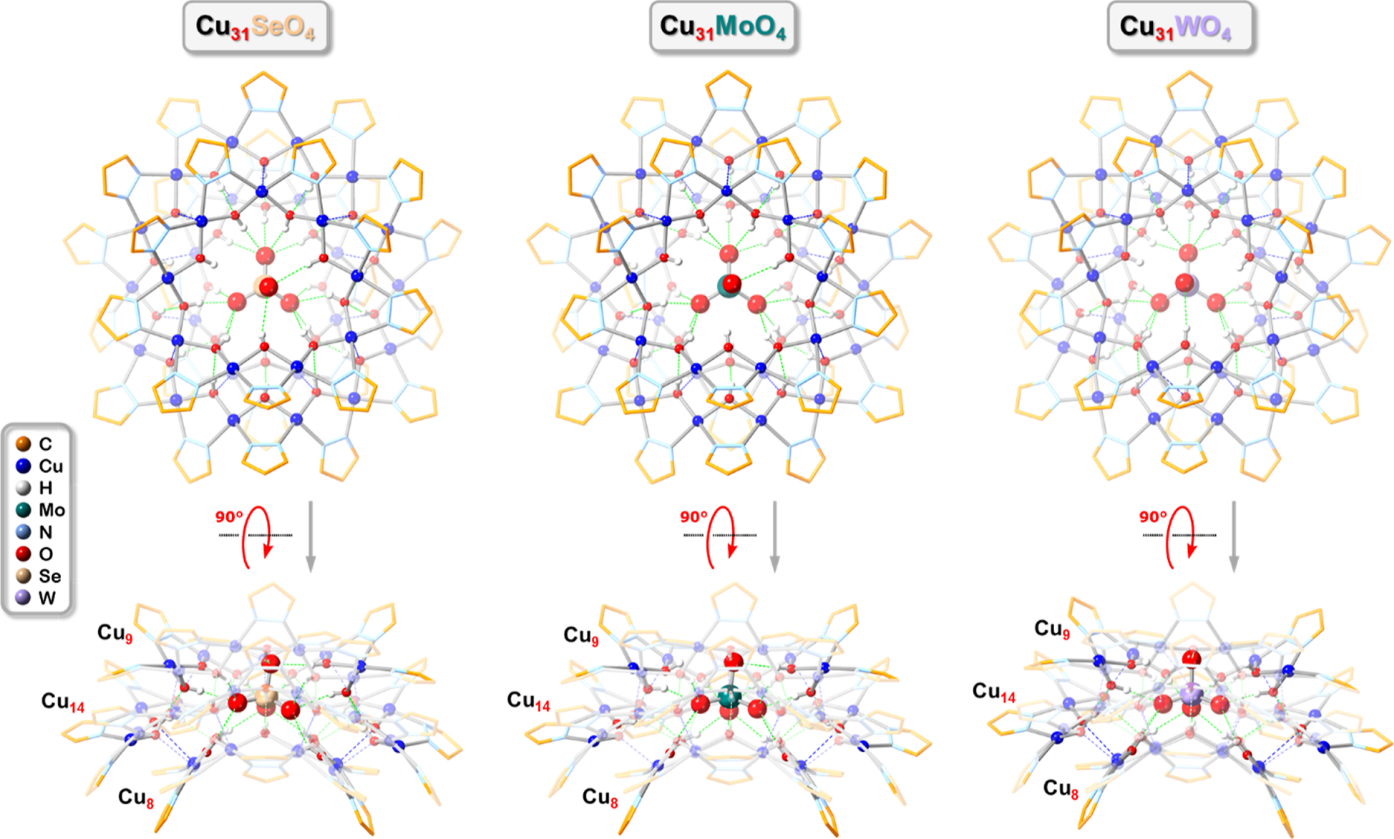
Ball-and-stick representation of the crystal structures of **Cu**_**31**_**SeO**_**4**_ (**1**), **Cu**_**31**_**MoO**_**4**_ (**4**) and **Cu**_**31**_**WO**_**4**_ (**6**) (top- and side-views). Green and blue dotted lines indicate hydrogen bonds and axial Cu···O interactions, respectively. Counterions, lattice solvent molecules and C–H bond H atoms are omitted for clarity, and only the major component is shown for disordered moieties.

**Figure 6:**
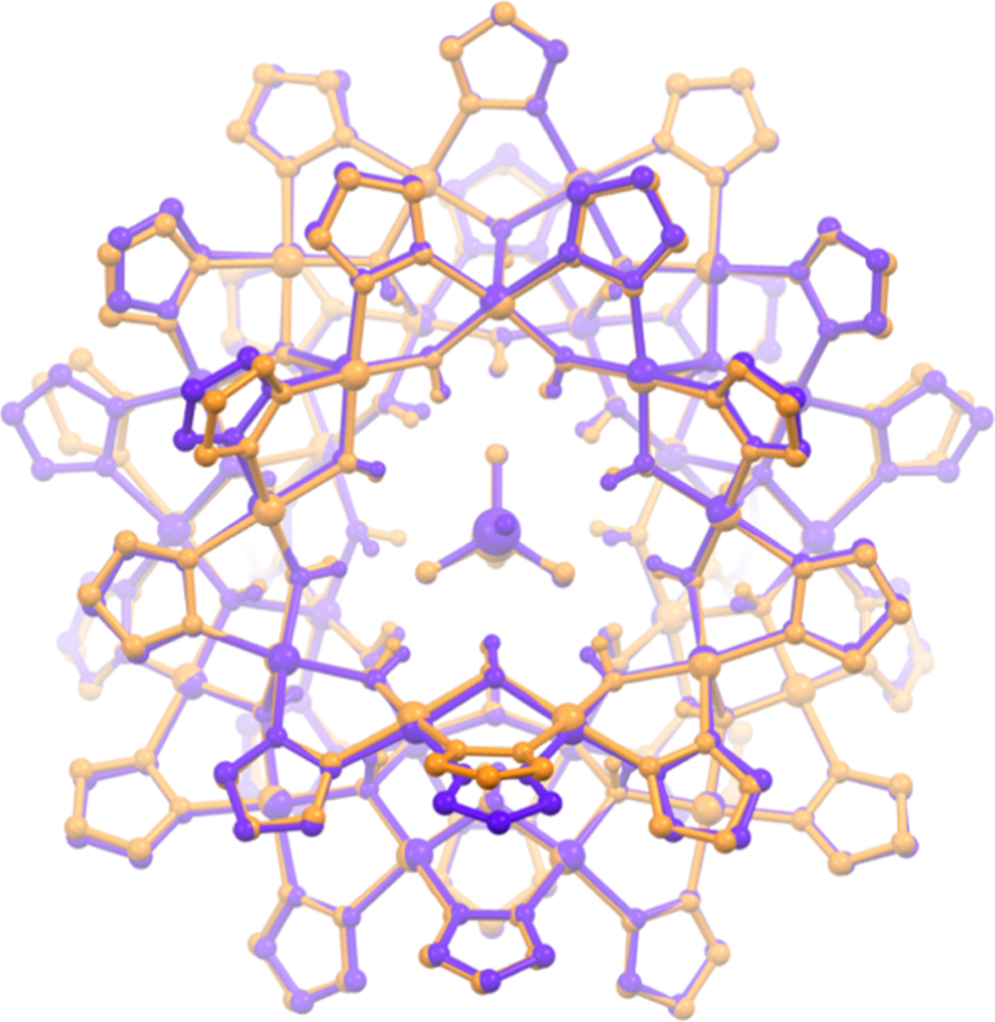
Overlay of the crystal structures of **Cu**_**31**_**MoO**_**4**_ in **4** (orange) and **5** (purple). C–H bond hydrogen atoms, counterions and solvent molecules are omitted for clarity, and only the major component is shown for disordered moieties.

**Figure 7:**
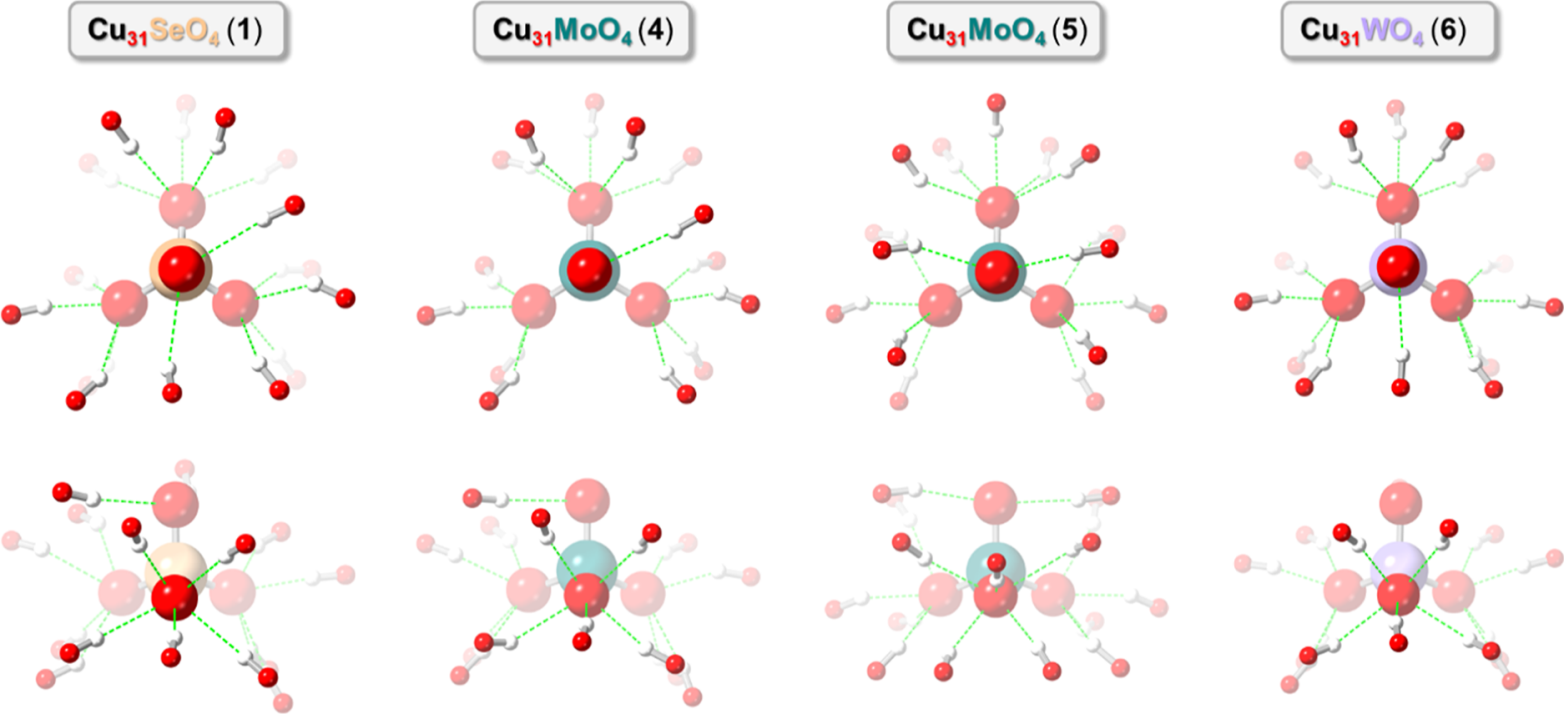
Comparison of the H-bonding patterns in the different **Cu**_**31**_**EO**_**4**_ (E = Se, Mo, W) nanojars.

The crystal structures of **Cu**_**31**_**SeO**_**4**_ (**1**), **Cu**_**31**_**MoO**_**4**_ (**4**) and **Cu**_**31**_**WO**_**4**_ (**6**) (Figure 5) are isomorphous with each other and with the ones of **Cu**_**31**_**SO**_**4**_,^70^**Cu**_**31**_**CrO**_**4**_,^61^ and **Cu**_**31**_**BeF**_**4**_.^69^ The nanojar units are located in general positions within the triclinic (*P*1̅) lattices of **1**, **4** and **6**, and as opposed to **Cu**_**28**_**EO**_**4**_ (E = Be, S, Cr, Mo), only their Cu_8_ ring displays pseudosymmetry relative to two orthogonal mirror planes, whereas their Cu_9_ and Cu_14_ rings are pseudosymmetrical relative to only one mirror plane. Figure S50 shows overlays of the crystal structures of **Cu**_**31**_**SeO**_**4**_ (**1**) with **Cu**_**31**_**MoO**_**4**_ (**4**) and **Cu**_**31**_**WO**_**4**_ (**6**), illustrating their nearly superimposable frameworks. Interestingly, the MoO_4_^2–^ anion is found in opposite orientations within the nanojar cavity in **4** compared to **5** (Figure S51), whereas the corresponding nanojar frameworks are almost superimposable (Figure 6). This suggests that the anion could be disordered over two different positions inside the Cu_31_ nanojar cavity. Indeed, in **1** the SeO_4_^2–^ anion is found disordered over three positions in a 0.49/0.35/0.16 ratio. In contrast, the anion is not disordered either in **4** or **5**, nor in **6**. A closer inspection of the structural details of the Cu_31_ nanojars in **4** and **5** does reveal subtle differences that might explain the different preferred orientation of the MoO_4_^2–^ anion in **4** vs **5**. Whereas the average of inter-ring Cu···O distances shorter than the sum of the van der Waals radii of Cu and O (2.92 Å) is practically identical [2.501(4) and 2.509(6) Å], there are only seven such interactions between the Cu_8_ and Cu_14_ rings in **4** but eight in **5**. Consequently, there are only 14 hydrogen-bonding interactions between the nanojar and the MoO_4_^2–^ anion with O···O distances shorter than 3.2 Å in **4**, but 15 in **5**. It should also be mentioned that the countercations neighboring the entrapped anion by the Cu_8_ and Cu_9_ side-rings in **4** (Bu_4_N^+^) are different from the ones in **5** (MePh_3_P^+^), which might be the origin of the observed differences. In all **Cu**_**31**_**EO**_**4**_ (E = Se, Mo or W) nanojars, one O atom of the EO_4_^2–^ anion forms five H-bonds and two O atoms form four H-bonds each with the nanojar, whereas the fourth O atom forms either one [E = Mo (**4**) or W] or two H-bonds [E = Mo (**5**) or Se] (Figures 7, S52 and S53).

**Cu**_**9+14+9**_**SeO**_**4**_ (**2**) crystallizes in the noncentrosymmetric, orthorhombic space group *P*2_1_2_1_2_1_ and it is isomorphous with **Cu**_**9+14+9**_**BeF**_**4**_.^69,70^ As shown in Figure S54, however, the overlay of the two structures is not as good as the one of **Cu**_**8+14+9**_**SeO**_**4**_ (**1**) and the isomorphous **Cu**_**8+14+9**_**BeF**_**4**_, although both were crystallized from 1,2-dichlorobenzene/*n*-heptane with Bu_4_N^+^ as the countercation. The average Se–O [1.59(2) Å] and Be–F [1.54(2) Å] bond lengths in the two Cu_32_ nanojars are similar, but the orientations of the anion within the nanojar cavity are different (both are disordered: SeO_4_^2–^ over two positions in a 0.66/0.34 ratio, and BeF_4_^2–^ over three positions in a 0.42/0.34/0.24 ratio). The differing H-bonding patterns with the SeO_4_^2–^ and BeF_4_^2–^ anions is shown in Figure S55.

The crystal structure of **Cu**_**8+14+10**_**MoO**_**4**_ provides for the first time structural information about the Cu_8+14+10_ nanojar with an entrapped tetrahedral anion (Figure 3). Although it contains the same Cu_8+14_ ring combination as the Cu_8+14+9_ nanojar, the Cu_8_ and Cu_14_ rings have slightly different conformations in **Cu**_**8+14+10**_**MoO**_**4**_ and **Cu**_**8+14+9**_**MoO**_**4**_, which are found cocrystallized in **5** (Figure S56). This is in contrast with the structure of the cocrystallized **Cu**_**6+12+9**_**PhPO**_**3**_ and **Cu**_**6+12+10**_**PhPO**_**3**_ nanojars reported recently, in which the Cu_6+12_ ring combination is not only identical, but is shared between the two overlapping nanojar units.^71^ The orientation of the MoO_4_^2–^ anion within the cavities of **Cu**_**8+14+10**_**MoO**_**4**_ and **Cu**_**8+14+9**_**MoO**_**4**_ is also different, with one of its O atoms pointing slightly to one side of the Cu_8_ ring (forming two H-bonds with it) in the former, but to the opposite side of the Cu_8_ ring (forming four H-bonds with it) in the latter (Figures S57 and S58).

### ^1^H NMR Spectroscopy

**Figure 8:**
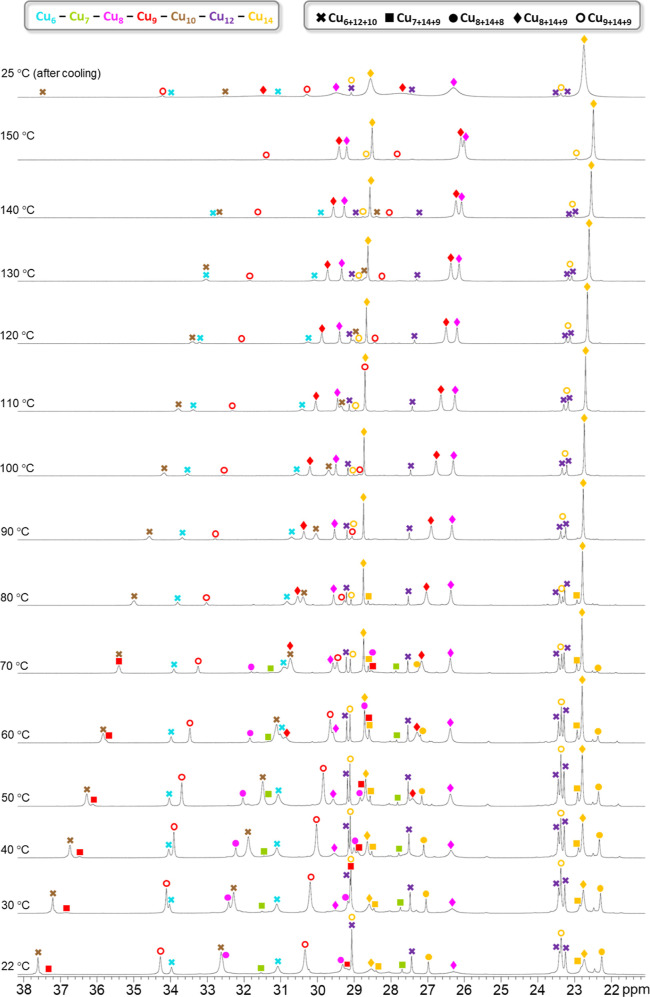
Variable-temperature ^1^H NMR spectra of the **Cu**_**n**_**SeO**_**4**_ (*n* = 28–32) nanojar mixture in DMSO-*d*_6_, showing pyrazolate proton signals in the 22–38 ppm window. The given temperatures are the target temperatures of the probe.

**Figure 9:**
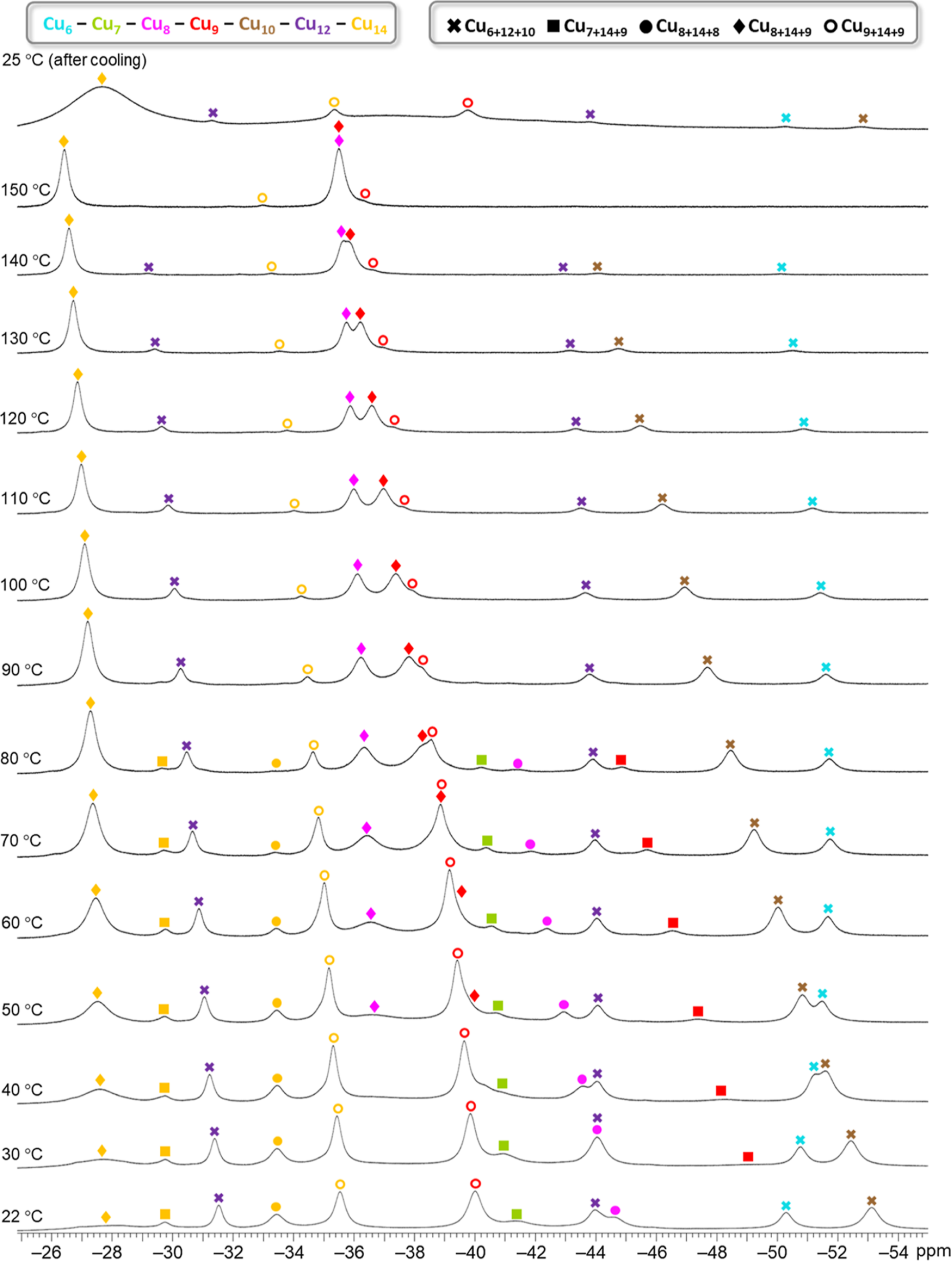
Variable-temperature ^1^H NMR spectra of the **Cu**_**n**_**SeO**_**4**_ (*n* = 28–32) nanojar mixture in DMSO-*d*_6_, showing OH proton signals in the −26 to −54 ppm window. The given temperatures are the target temperatures of the probe.

^1^H nuclear magnetic resonance (NMR) spectroscopy provides the most comprehensive description of the composition of nanojar mixtures in solution (Table S26). While ESI-MS indicates nanojar sizes, it is ^1^H NMR that can differentiate between isomers for a given size. For example, two isomers of the Cu_29_ nanojar (Cu_7+13+9_ and Cu_8+13+8_)^60,64,66,69^ and two isomers of the Cu_32_ nanojar (Cu_8+14+10_ and Cu_9+14+9_) were described earlier.^60,69^ During the present work, we also recognized ^1^H NMR signatures of two isomers of the Cu_30_ nanojar, Cu_7+14+9_ and Cu_8+14+8_. Although individual crystals of these Cu_30_ isomers were isolated earlier (as heteroleptic nanojars with pz/3,5-Me_2_pz ligands and CO_3_^2–^ as the entrapped anion) and their crystal structures were studied,^57^ it is for the first time that NMR peaks of a Cu_30_ nanojar, which usually has been obtained in minor amounts, could be assigned. Indeed, the **Cu**_**n**_**SeO**_**4**_ mixture obtained by the depolymerization of [*trans*-Cu^II^(μ-OH)(μ-pz)]_∞_ in refluxing toluene in the presence of (Bu_4_N)_2_SeO_4_ contains the largest fraction of Cu_30_ ever observed with a homoleptic pz-nanojar (Figure 2). Variable-temperature (VT) ^1^H NMR measurements in DMSO-*d*_6_ over the 22–150 °C range show that the **Cu**_**7+14+9**_**SeO**_**4**_ and **Cu**_**8+14+8**_**SeO**_**4**_ nanojars gradually decompose by 100 °C and transform into the most stable nanojar with the SeO_4_^2–^ ion, **Cu**_**31**_**SeO**_**4**_. The Cu_28_ and Cu_9+14+9_ nanojars are also unstable on heating and at 150 °C only **Cu**_**31**_**SeO**_**4**_ prevails, with traces of **Cu**_**9+14+9**_**SeO**_**4**_ (Figures 8 and 9).

Since an almost pure sample of **Cu**_**28**_**SeO**_**4**_ could be isolated by fractional precipitation (as described above), its gradual, clean transformation into **Cu**_**31**_**SeO**_**4**_ on heating in DMSO-*d*_6_ solution was demonstrated by VT ^1^H NMR over the 22–150 °C range (Figures S59 and S60). After cooling to ambient temperature, the corresponding ^1^H NMR spectrum shows mostly **Cu**_**31**_**SeO**_**4**_ with small amounts of **Cu**_**28**_**SeO**_**4**_, indicating re-equilibration that allows for the formation of minor amounts of **Cu**_**28**_**SeO**_**4**_ at 23 °C.

Interestingly, the **Cu**_**n**_**SeO**_**4**_ mixture obtained by depolymerization in refluxing chlorobenzene has a very different composition than the one obtained from refluxing toluene. As shown by its ESI-MS spectrum in Figure S12, this sample contains **Cu**_**31**_**SeO**_**4**_ and **Cu**_**32**_**SeO**_**4**_ with only traces of **Cu**_**28**_**SeO**_**4**_. Even more intriguingly, in this case ^1^H NMR shows two isomers of the Cu_32_ nanojar, **Cu**_**9+14+9**_**SeO**_**4**_ and **Cu**_**8+14+10**_**SeO**_**4**_, whereas in the previous case only **Cu**_**9+14+9**_**SeO**_**4**_ was observed. The peaks corresponding to the Cu_8_–Cu_10_ rings of **Cu**_**8+14+9**_**SeO**_**4**_ and **Cu**_**8+14+10**_**SeO**_**4**_ nanojars are very broad at room temperature, but become sharper at higher temperatures. As observed with the other sample, **Cu**_**9+14+9**_**SeO**_**4**_ and the traces of **Cu**_**28**_**SeO**_**4**_ disappear on increasing the temperature. In contrast, the other Cu_32_ isomer which was not found in the sample obtained from refluxing toluene, **Cu**_**8+14+10**_**SeO**_**4**_ is stable and it is found unchanged along with **Cu**_**31**_**SeO**_**4**_ at 150 °C (Figures S61 and S62).

A similar behavior is observed in the case of the molybdate and tungstate nanojar mixtures obtained by depolymerization in refluxing toluene, whereby the Cu_6+12+10_ and Cu_9+14+9_ nanojars gradually decompose on heating and the Cu_8+14+9_ and Cu_8+14+10_ nanojars prevail at 150 °C (Figures S63–S66).

The ^1^H NMR chemical shifts of the selenate, molybdate and tungstate nanojars, however, are quite different (Table S26). In fact, a correlation between these chemical shifts and the size of the entrapped anion is observed from the smallest sulfate anion to the largest tungstate anion. For example, in the case of the proton in the 4-position of the pyrazolate units of the Cu_10_ ring of the Cu_28_ nanojar, the corresponding chemical shifts at 22 °C are 40.27 (SO_4_^2–^), 37.66 (SeO_4_^2–^), 37.48 (CrO_4_^2–^), 35.24 (MoO_4_^2–^) and 34.95 ppm (WO_4_^2–^) (change: 5.3 ppm units). The chemical shift change of the protons in the 3/5-position of the same pyrazolate units is slightly smaller, ranging from 34.50 with SO_4_^2–^ to 30.64 ppm with WO_4_^2–^ (change: 3.9 units). The largest disparity is observed for the OH protons of the Cu_10_ ring, which vary from −57.75 with SO_4_^2–^ to −46.66 ppm with WO_4_^2–^ (change: 11.1 units). The protons of the Cu_6_ ring are less affected by changing the anion and an inverse correlation is observed for the pyrazolate 4- and 3/5-positions, with an increase in the chemical shift from 33.61 (SO_4_^2–^) to 34.45 (WO_4_^2–^) (change: 0.8 ppm units) and from 30.77 (SO_4_^2–^) to 31.37 ppm (WO_4_^2–^) (change: 0.6 units), respectively. The corresponding OH proton signals shift from −51.49 with SO_4_^2–^ to −46.19 ppm with WO_4_^2–^ (change: 5.3 ppm units). The least affected are the Cu_12_ ring protons, which vary by only 0.12/0.21 ppm in the case of the pyrazolate 4-position and 3.24/0.60 ppm in the case of the OH protons (with half of them pointing toward the Cu_10_ ring and the other half toward the Cu_6_ ring in each case), and they are practically identical in the case of the pyrazolate 3/5-position.

**Figure 10:**
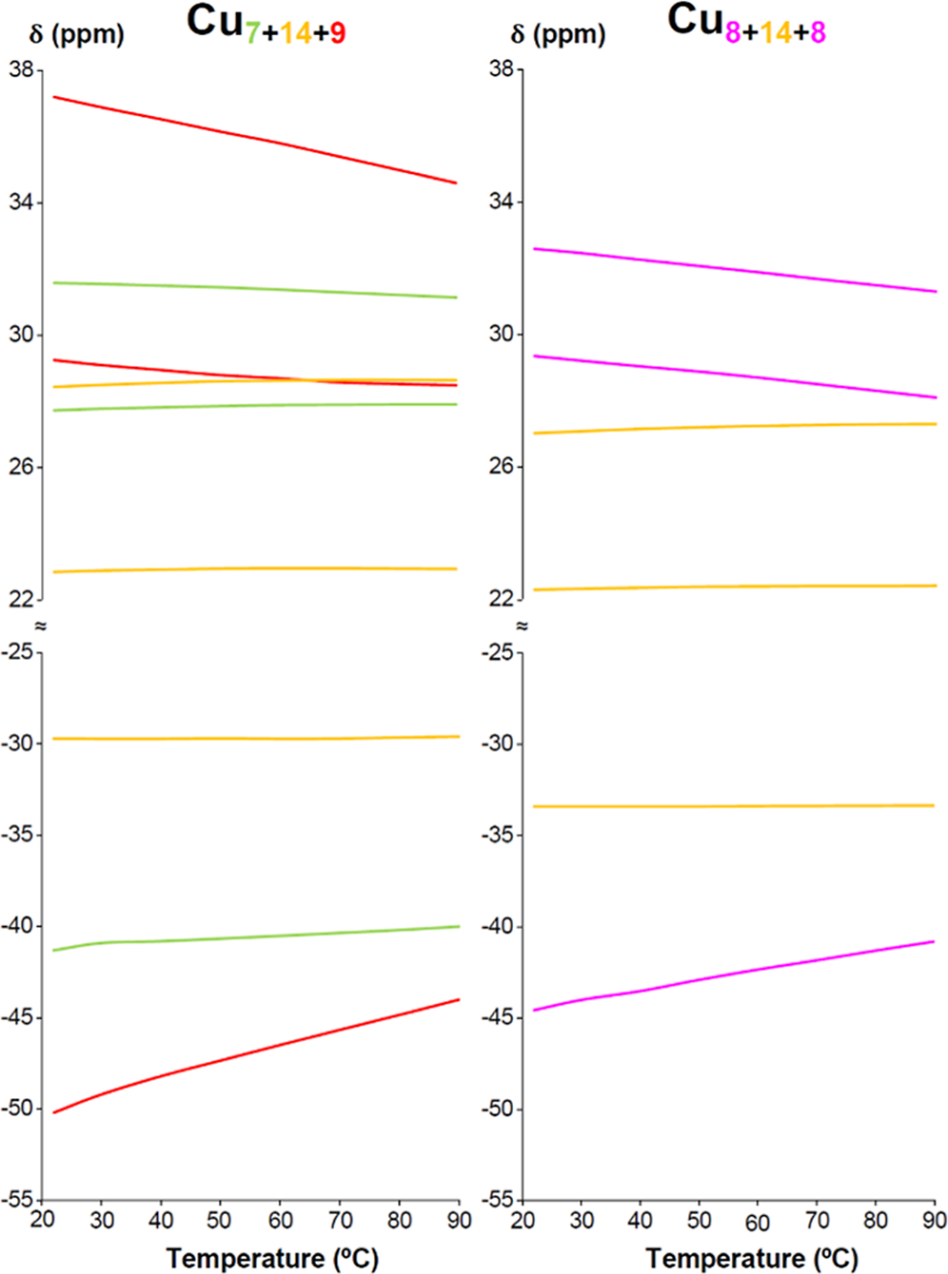
Temperature-dependent variation of the chemical shifts (δ) in DMSO-*d*_6_ of different Cu_*x*_ ring protons in the two **Cu**_**30**_**SeO**_**4**_ nanojars.

As observed earlier, the nanojar proton peaks experience strong downfield and upfield shifts in the case of the pyrazolate and OH protons, respectively. This is due to the presence of the paramagnetic Cu(II) centers, which also lead to significant peak broadening and loss of the *J* coupling between nuclei. With SeO_4_^2–^, MoO_4_^2–^ and WO_4_^2–^, the peaks of the Cu_8_ and Cu_9_ rings of the Cu_31_ nanojar, as well as the Cu_8_ and Cu_10_ rings of the Cu_32_ nanojar are especially broad, to the point that some are barely observable at ambient temperature. Nevertheless, most nanojar peaks are relatively sharp, owing to the strong antiferromagnetic coupling between the Cu(II) centers.^66^ As with paramagnetic compounds in general, increasing the temperature leads to sharpening of the peaks. Furthermore, most peaks become less paramagnetically shifted at higher temperatures, indicating a Curie behavior (δ ∝ 1/T) (Figures 10 and S67–S69). The most notable exception is the OH peak of the Cu_6_ rings in the Cu_28_ nanojars, which shows a slight anti-Curie behavior (more paramagnetically shifted at higher temperatures) from 22 °C up to 70–90 °C. The largest temperature dependence is observed in the case of the Cu_9_ and Cu_10_ rings, whereas the pyrazolate protons of the central rings (Cu_12_–Cu_14_) show almost no variation with temperature.

### UV–vis Spectroscopy

**Figure 11:**
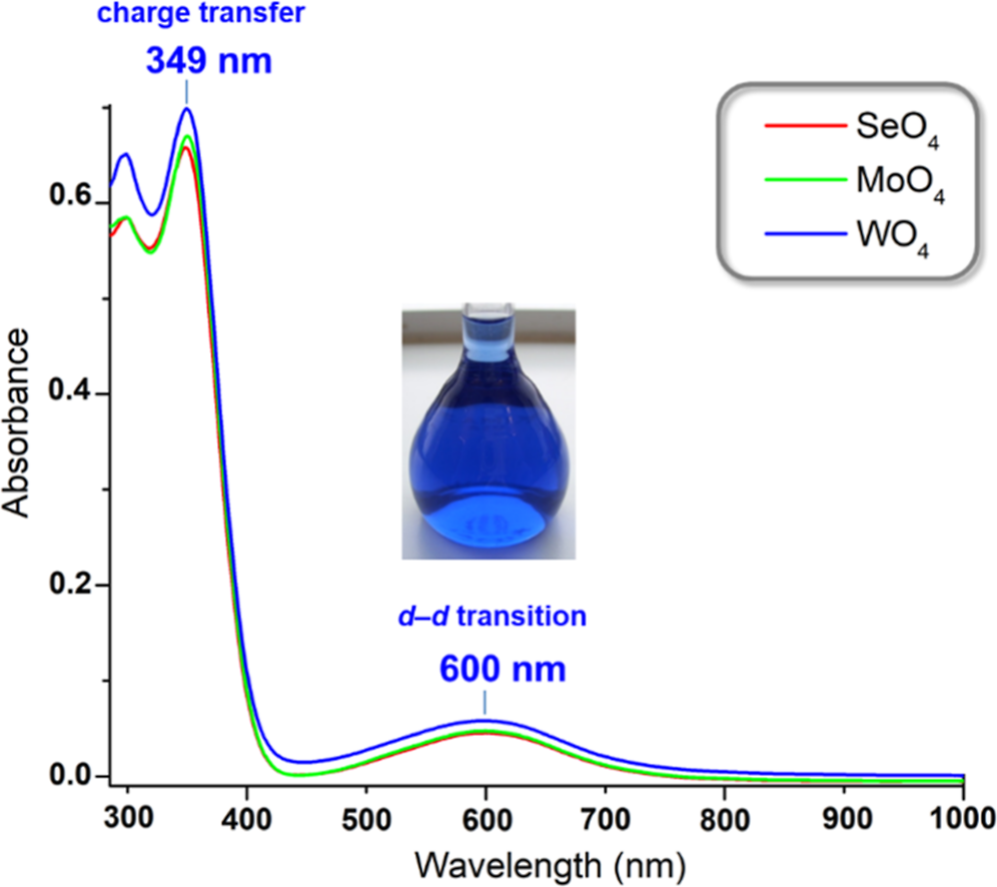
UV–vis spectra of **Cu**_***n***_**EO**_**4**_ (E = Se, Mo, W; *n* = 28–33) in THF (20 μM).

The UV–vis spectra of **Cu**_**n**_**EO**_**4**_ (E = Se, Mo, W; *n* = 28–33) in THF display two peaks with absorption maxima at 349–350 and 600 nm (Figure 11), corresponding to ligand-to-metal charge-transfer and *d*–*d* transitions, respectively (**Cu**_***n***_**SeO**_**4**_: ε_349nm_ = 3.3 × 10^4^ L mol^–1^ cm^–1^ and ε_600nm_ = 2.3 × 10^3^ L mol^–1^ cm^–1^; **Cu**_***n***_**MoO**_**4**_: ε_350nm_ = 3.4 × 10^4^ L mol^–1^ cm^–1^ and ε_600nm_ = 2.4 × 10^3^ L mol^–1^ cm^–1^; **Cu**_***n***_**WO**_**4**_: ε_349nm_ = 3.5 × 10^4^ L mol^–1^ cm^–1^ and ε_600nm_ = 2.9 × 10^3^ L mol^–1^ cm^–1^). The observed λ_max_ values and extinction coefficients are very similar to the ones measured for analogous nanojar mixtures with other entrapped anions (λ_max_ = 345–351 and 599–608 nm; ε = 2 × 10^4^ to 3 × 10^4^ and 2 × 10^3^ to 3 × 10^3^ L mol^–1^ cm^–1^),^58,59,61,71^ indicating that the anion has negligible effects on the UV–vis absorption of the nanojar framework. It is noteworthy that the extinction coefficient of the *d*–*d* transition of nanojars is much larger (∼16-fold) than the corresponding one of low-nuclearity analogues with similar chromophores, such as Cu_3_(μ_3_-OH)(μ-pz)_3_(NO_3_)_2_(H_2_O), which displays an identical λ_max_ value.^72^ This is attributable to the much higher density of chromophores in nanojars.

### Assessment of Selenate, Molybdate and Tungstate Binding Strength by Competitive Anion Binding

Nanojars can only be isolated with an entrapped anion. Due to the inaccessibility of an anion-free nanojar host and the impossibility of carrying out host–guest titrations, the strength of SeO_4_^2–^, MoO_4_^2–^ and WO_4_^2–^ binding by nanojars was assessed by competitive binding experiments with Ba^2+^, which forms insoluble salts with these anions (*K*_sp_ in H_2_O at 25 °C: BaSeO_4_ – 3.40 × 10^–8^; BaMoO_4_ – 3.54 × 10^–8^).^1^ Experiments were conducted under two different conditions: (a) heterogeneously, by vigorously stirring a solution of **Cu**_***n***_**EO**_**4**_ (E = Se, Mo, W; *n* = 28–33) in water-immiscible 2-methyltetrahydrofuran (2-MeTHF) with an aqueous solution of Ba(NO_3_)_2_, and (b) homogeneously, by using barium dioctyl sulfosuccinate, Ba(DOSS)_2_, which is soluble in 2-MeTHF together with the nanojar mixture. In neither case was a BaEO_4_ precipitate observed, and ESI-MS(−) of the organic layer shows no nanojar degradation products (such as low-nuclearity copper pyrazolate complexes). Nevertheless, changes in the composition of the nanojar mixtures are observed in certain cases. The heterogeneous treatment with aqueous Ba^2+^ leads to the reduction of the amount of **Cu**_**28**_**SeO**_**4**_ and the complete disappearance of **Cu**_**29**_**SeO**_**4**_ and **Cu**_**30**_**SeO**_**4**_, the almost complete disappearance of **Cu**_**31**_**MoO**_**4**_, and the significant reduction of the amount of **Cu**_**28**_**WO**_**4**_ and **Cu**_**31**_**WO**_**4**_ along with the disappearance of **Cu**_**30**_**WO**_**4**_ (Figures S34–S36). The heterogeneous treatment does not cause significant changes in the case of **Cu**_***n***_**SeO**_**4**_ and **Cu**_***n***_**MoO**_**4**_ (*n* = 28–32), but leads to the disappearance of **Cu**_**28**_**WO**_**4**_ and **Cu**_**30**_**WO**_**4**_ in the case of **Cu**_***n***_**WO**_**4**_. These results indicate a weaker binding of the SeO_4_^2–^, MoO_4_^2–^ and WO_4_^2–^ anions by the smaller Cu_28_–Cu_30_ nanojars and a preferential binding by the larger Cu_31_ and Cu_32_ nanojars in the case of SeO_4_^2–^ and MoO_4_^2–^/WO_4_^2–^, respectively.

### Liquid–Liquid Extraction of SeO_4_^2–^, MoO_4_^2–^ and WO_4_^2–^ from Water into an Organic Solvent

Similarly to other tetrahedral oxodianions such as SO_4_^2–^ and CrO_4_^2–^, SeO_4_^2–^ (Δ*G*_h_^°^ = −900 kJ/mol), MoO_4_^2–^ and WO_4_^2–^ are highly hydrophilic and are difficult to extract from aqueous solutions into an organic solvent. Although experimental values are not available, the free energies of hydration for MoO_4_^2–^ and WO_4_^2–^ are estimated to be −963 and −949 kJ/mol, respectively.^73^ Extraction experiments were conducted by stirring an aqueous solution of Na_2_EO_4_ (E = Se, Mo, W) with nanojar ingredients [Cu(NO_3_)_2_, pyrazole, NaOH and Bu_4_NOH] in THF. Pure THF and water are miscible in any ratio, but in the presence of inorganic salts the THF layer, which contains the nanojars, separates from the aqueous layer. Analysis of the organic layer by ESI-MS indicates the presence of mostly **Cu**_***n***_**EO**_**4**_ (E = Se, Mo, W; *n* = 28–34), together with small amounts of **Cu**_**27**_**CO**_**3**_ and traces of **Cu**_**29**_**CO**_**3**_ (Figures S37–S39). The extraction efficiency (as measured by the obtained yields of nanojars) is 69–79%.

## Conclusions

Previously, we described the supramolecular binding and extraction from water into organic solvents of small oxoanions, including CO_3_^2–^ (C–O: 1.28 Å), SO_4_^2–^ (S–O: 1.48 Å) and CrO_4_^2–^ (Cr–O: 1.63 Å) by nanojars, documenting an extremely strong binding by the inability of Ba^2+^ ions to precipitate the corresponding barium salts from [anion⊂{*cis*-Cu^II^(μ-OH)(μ-pz)}_*n*_]^2–^ (*n* = 27–33) solutions. In this work, we explored the boundaries of oxoanion binding by nanojars using even larger oxoanion analogues, SeO_4_^2–^ (Se–O: 1.64 Å), MoO_4_^2–^ (Mo–O: 1.76 Å), WO_4_^2–^ (W–O: 1.77 Å) and TeO_4_^2–^ (Te–O: 1.81 Å).

With entrapped oxoanions, nanojar sizes ranging from Cu_27_ to Cu_34_ have been observed so far. When self-assembled in solution from their components (Cu^2+^ ions, pyrazole and a base) under ambient conditions, nanojars of various different sizes are templated by the anion under kinetic control. Nevertheless, a correlation between the sizes of the anion guest and the nanojar host becomes apparent. With the smallest oxoanion studied, CO_3_^2–^, nanojars ranging from Cu_27_ to Cu_31_ (except Cu_28_) have been obtained. With the second smallest anion, SO_4_^2–^, the range extends to Cu_33_. With CrO_4_^2–^ and SeO_4_^2–^, only traces of the Cu_27_ nanojar form. With MoO_4_^2–^ and WO_4_^2–^ no Cu_27_ nanojars are observed at all, and the range extends to Cu_34_. Gradual heating in a DMSO-*d*_6_ solution to 150 °C and monitoring by ^1^H NMR spectroscopy reveals varying thermal stabilities for nanojars of different sizes with a given anion. With CO_3_^2–^, only Cu_27_ and Cu_29_ (Cu_8+13+8_) survive at 150 °C. With SO_4_^2–^, in contrast, Cu_31_ and Cu_29_ (Cu_8+13+8_) are still observed at that temperature, whereas with CrO_4_^2–^ only Cu_31_ prevails. With SeO_4_^2–^, MoO_4_^2–^ and WO_4_^2–^, Cu_32_ (Cu_8+14+10_) along with Cu_31_ survive heating to 150 °C. Further evidence for the preferred size match between guest and host is provided by NH_3_ etching studies. Saturating a THF solution at ambient temperature with NH_3_ leads to a rearrangement of nanojar sizes in a mixture to favor the most stable nanojar. Thus, pure Cu_27_ or Cu_31_ nanojars are obtained with CO_3_^2–^ and SO_4_^2–^, respectively. With CrO_4_^2–^ and SeO_4_^2–^, Cu_32_ survives the NH_3_ treatment along with Cu_31_, whereas with the largest MoO_4_^2–^ and WO_4_^2–^ only Cu_32_ remains.

In contrast to traditional binding/extraction agents, which are preformed entities that can be isolated on their own, nanojars only exist with an entrapped anion. Moreover, self-assembly of the nanojar only occurs around suitable anions. For example, anions similar in size to the ones mentioned above but with a 1– overall charge, such as NO_3_^–^ (N–O: 1.29 Å) and ClO_4_^–^ (Cl–O: 1.43 Å), are not suitable for nanojar formation. Consequently, 2– charged anions are bound selectively in the presence of 1– charged anions even when the latter are found in large excess. The present work reveals that the anti-Hofmeister bias (preference for doubly charged anions with larger hydration energies) observed extends within the series of doubly charged anions as well. In particular, the preference of nanojars for the smallest doubly charged oxoanion, CO_3_^2–^, is noted. The self-assembly of SO_4_^2–^ nanojars (using a Cu^2+^/SO_4_^2–^ ratio of 1:1) proceeds cleanly without the need of an inert atmosphere within the closed reaction flask. With the larger anions, however, interference from CO_3_^2–^ is observed, which becomes more substantial with the larger anions.

The amount of **Cu**_***n***_**CO**_**3**_ impurity in the **Cu**_***n***_**EO**_**4**_ (E = Se, Mo, W) nanojar mixtures could be substantially reduced (almost completely excluded in the case of SeO_4_^2–^) when the Cu^2+^, pyrazolate and base sources were combined into [*trans*-Cu^II^(μ-OH)(μ-pz)]_∞_, thus eliminating the need for additional base in the synthesis. The formation of the nanojars by depolymerization, however, is not ideal for the liquid–liquid extraction of anions from water. Nevertheless, the SeO_4_^2–^, MoO_4_^2–^ and WO_4_^2–^ ions can be successfully extracted from water into an organic phase by nanojars obtained by self-assembly, which contain relatively small amounts of carbonate. Also, competitive binding experiments with Ba^2+^ ions confirm strong binding even of these larger anions by the Cu_31_ and Cu_32_ nanojars, which do not release the entrapped anion preventing precipitation of the corresponding insoluble barium salts.

No nanojars could be obtained with the TeO_4_^2–^ ion. Crystallographic studies have confirmed that nanojars such as Cu_31_ can accommodate increasingly larger tetrahedral oxoanions, from SO_4_^2–^ (S–O: 1.48 Å) to WO_4_^2–^ (W–O: 1.77 Å). Also, the crystal structure of a larger Cu_32_ nanojar with entrapped MoO_4_^2–^ has been described. Furthermore, even larger Cu_33_ and Cu_34_ nanojars have been detected by ESI-MS with these anions. Therefore, we attribute the absence of nanojars with TeO_4_^2–^ (Te–O: 1.81 Å) not to the size of the anion, but to the strong preference of tellurium to be hexacoordinate resulting in complexes other than nanojars. Although the spatial fit of octahedral TeO_6_^6–^ ion in nanojars is imaginable, its large charge makes this species unlikely to be found in a neutral supramolecular receptor bound solely by H-bonds. Multiply protonated species, H_*x*_TeO_6_^(6–*x*)–^ are also unlikely to be bound by nanojars, due to their OH groups interfering with H-bonding within the nanojar cavity.

The binding studies of SeO_4_^2–^, MoO_4_^2–^ and WO_4_^2–^ ions by nanojars expanded not only the range of anions that can be bound by these unique anion sequestration agents, but also the range of nanojar sizes. The X-ray crystal structure of the Cu_8+14+10_ nanojar represents the tenth confirmed combination of three Cu_*x*_ rings observed so far in nanojars. Furthermore, the observation of Cu_33_ and Cu_34_ nanojars by ESI-MS foretells the existence of Cu_9+14+10_ and Cu_10+14+10_ ring combinations as well, and hints to the possibility of obtaining even larger nanojars with anions of increasing size.

## Experimental Section

### General

All commercially available chemicals were used as received [solvents are ACS or high-performance liquid chromatography (HPLC) grade, and THF is inhibited with 250 ppm BHT]. Cu(NO_3_)_2_·2.5H_2_O (ACS reagent, 98%), CuSO_4_·5H_2_O (ACS reagent, 98.0–102.0%), NaOH (ACS reagent, 97%), Et_3_N (≥99%), ^*n*^Bu_3_N (≥98.5%), H_2_MoO_4_ (ACS reagent, ≥85.0% MoO_3_), H_2_WO_4_ (99%), (NH_4_)_2_MoS_4_ (99.97%), (NH_4_)_2_WS_4_ (≥99.9%) and methyltri(phenyl)phosphonium bromide (98%) were manufactured by Sigma-Aldrich; Na_2_SeO_4_ (anhydrous, ≥99.8%), Na_2_WO_4_·2H_2_O (ACS reagent, ≥99%), H_2_SeO_4_ (40% in H_2_O), H_2_TeO_4_·2H_2_0 (≥99%), Bu_4_NOH (HPLC grade, 1.0 M in H_2_O) and NH_4_OH (ACS reagent, 28–30%) by Thermo Scientific; pyrazole (99%) by Oakwood Chemical; Ag_2_SeO_4_ (C.P.) by City Chemical; Cu(OEt)_2_ by ProChem, Inc.; Ba(NO_3_)_2_ (ACS reagent, 99%) by Spectrum Chemical; Pb(NO_3_)_2_ (ACS reagent, 100%) by J. T. Baker Chemical; CuBr_2_ (Analytical Reagent) and Na_2_MoO_4_·2H_2_O (Analytical Reagent, ≥99.5%) by Mallinckrodt Chemical Works. [*trans*-Cu^II^(μ-OH)(μ-pz)]_∞_,^71^ Mo^VI^_8_O_12_(μ-O)_9_(μ-pz)_6_(pzH)_6_·3pzH·0.5H_2_0,^66^ tri(benzyl)methylammonium nitrate^74^ and Ba(DOSS)_2_ were prepared using published procedures.^69^ (Bu_4_N)_2_EO_4_ (E = Se, Te, Mo, W) was prepared *in situ* by combining Bu_4_NOH (1 M in H_2_O) with H_2_EO_4_ in a 2:1 molar ratio. NH_3_(g) was generated by gently heating an NH_4_OH solution in a stoppered Erlenmeyer flask with side arm, connected to a Pasteur pipet with Tygon 2375 tubing. Deionized water was freshly boiled and cooled to room temperature under N_2_(g). NMR spectra were collected on a Jeol JNM-ECZS (400 MHz) instrument, and UV–vis measurements were carried out on a Shimadzu UV-1650PC spectrophotometer. No noticeable solvent effects on the composition of nanojar mixtures were observed with the solutions in different solvents used for these studies. Pyrazole is very stable and is resistant to oxidation and reduction,^75^ and no reactivity at the pyrazole ring was observed.

### Synthesis of Copper(II) Selenate (CuSeO_4_·5H_2_O)

(76) Ag_2_SeO_4_ (9.000 g, 25.09 mmol) was added to a solution of CuBr_2_ (5.408 g, 24.21 mmol) in H_2_O (60 mL) under vigorous stirring. The green color of the solution quickly turned blue, and the white color of the solid suspension turned yellow. After stirring for 2 h in the absence of light, the solid was filtered out and was rinsed with water. The blue filtrate was left to evaporate slowly, yielding large blue crystals and a blue microcrystalline powder (7.215 g total). After dissolving the crude product in H_2_O (50 mL) and filtering out a small amount of light-yellow insoluble powder, acetone (50 mL) was added to the filtrate under stirring. A light-blue microcrystalline solid formed which was filtered out, washed with acetone and dried in air. Yield: 6.809 g (95%).

### Synthesis of Lead(II) Selenate (PbSeO_4_)^77^

Pb(NO_3_)_2_ (17.530 g, 52.93 mmol) and Na_2_SeO_4_ (10.165 g, 53.80 mmol) were dissolved separately in H_2_O (1 L each). The lead nitrate solution was added dropwise to the vigorously stirred sodium selenate solution. The white precipitate formed was stirred in the mother liquor for 3 h, then it was filtered out, washed extensively with H_2_O, dried in air and then in an oven at 120 °C overnight. Yield: 18.134 g (98%).

### Synthesis of Copper(II) Molybdate Hydroxide (Cu_3_(MoO_4_)_2_(OH)_2_)^78^

A solution of CuSO_4_·5H_2_O (24.969 g, 0.100 mol) in H_2_O (200 mL) was brought to reflux and a solution of Na_2_MoO_4_·2H_2_O (24.195 g, 0.100 mol) in H_2_O (200 mL) was added dropwise under stirring. The light-green precipitate formed was refluxed for 7 h and then it was filtered warm. The solid was washed thoroughly with H_2_O and air-dried. Yield: 15.986 g (88%).

### Synthesis of Copper(II) Tungstate (CuWO_4_·2H_2_O)^76^

Cu(NO_3_)_2_·2.5H_2_O (10.000 g, 42.99 mmol) was dissolved in H_2_O (200 mL) and then it was added to a solution of Na_2_WO_4_·2H_2_O (14.182 g, 42.99 mol) in H_2_O (200 mL) under stirring. The light-green gelatinous precipitate was stirred for 15 min and was left to settle. After 3 days, the supernatant solution was decanted and the solid was filtered out, washed with H_2_O and air-dried. Yield: 14.404 g (96%).

### General Procedure for the Synthesis of (Bu_4_N)_2_[EO_4_⊂{Cu(OH)(pz)}_*n*_] (**Cu**_***n***_**EO**_**4**_; E = Se, Mo, W; *n* = 28–34) by Self-Assembly

A Cu^2+^ source [CuSeO_4_·5H_2_O, Cu_3_(MoO_4_)_2_(OH)_2_, CuWO_4_·2H_2_O, Cu(NO_3_)_2_·2.5H_2_O or Cu(OEt)_2_], pyrazole, a base [NaOH, Bu_4_NOH (1 M in H_2_O), Et_3_N, ^*n*^Bu_3_N or Cu(OEt)_2_] and an anion source [Na_2_SeO_4_, Na_2_MoO_4_·2H_2_O, Na_2_WO_4_·2H_2_O, H_2_TeO_4_·2H_2_O, (Bu_4_N)_2_EO_4_, (NH_4_)_2_MoS_4_, (NH_4_)_2_WS_4_ or the Cu^2+^ salts listed above] were stirred in THF or DMF (250 mL) for 3 days in a sealed flask, using the molar ratios shown in Figures S1–S10 and S14–S22. The resulting deep-blue solutions were filtered and the solid residues were rinsed with THF. Evaporation of the solvent under vacuum afforded the products as dark blue powders. In the case of tellurate, a sticky, dark-green residue was obtained, the ESI-MS spectrum of which shows no nanojar species, only low-nuclearity Cu_3_ complexes (Figure S29).

### General Procedure for the Synthesis of (Bu_4_N)_2_[EO_4_⊂{Cu(OH)(pz)}_*n*_] (**Cu**_***n***_**EO**_**4**_; E = Se, Mo, W; *n* = 28–34) by Depolymerization

To (Bu_4_N)_2_EO_4_ (E = Se, Mo, W) prepared *in situ* by stirring Bu_4_NOH (1 M in H_2_O, 0.219 mL, 0.219 mmol) and H_2_SeO_4_, H_2_TeO_4_·2H_2_O, H_2_MoO_4_ or H_2_WO_4_ (0.120 mmol) in toluene or chlorobenzene (50 mL) was added [*trans*-Cu(OH)(pz)]_∞_ (0.5000 g, 3.387 mmol) and the mixture was refluxed overnight (14–16 h). The resulting reaction mixture was filtered, the solid was rinsed with toluene (or chlorobenzene) and then the solvent was removed from the deep blue filtrate under vacuum. The dark blue solid product was washed with water to remove excess TBA salts, and was dried under vacuum. Yields based on [*trans*-Cu(OH)(pz)]_∞_ (for an average *n* = 31): Se – 0.3324 g (59%); Mo – 0.4026 g (71%); W – 0.3517 g (61%). ESI-MS spectra for the products are shown in Figure 2, ^1^H NMR spectra in Figures 8, 9 and S61–S66, and UV–vis spectra in Figure 11. In the case of tellurate, a sticky, dark-green residue was obtained as described above, which contained no nanojars based on its ESI-MS spectrum.

### Conversion of a Mo_8_ Cluster into **Cu**_***n***_**MoO**_**4**_ (*n* = 28, 31, 32) Nanojars

Mo^VI^_8_O_12_(μ-O)_9_(μ-pz)_6_(pzH)_6_·3pzH·0.5H_2_0 (0.0500 g, 0.0235 mmol) was stirred together with Cu(NO_3_)_2_·2.5H_2_O (0.0823 g, 0.354 mmol) and Bu_4_NOH (1 M in H_2_O; 1.061 mL, 1.06 mmol) in THF (10 mL) for 3 days in a closed vessel. The reaction mixture was then filtered into water (100 mL) under stirring and the resulting blue precipitate was isolated by filtration and was dried under vacuum. ESI-MS reveals that the product consists of **Cu**_***n***_**MoO**_**4**_ (*n* = 28, 31, 32) together with small amounts of **Cu**_***n***_**CO**_**3**_ (*n* = 27, 29) (Figure S23).

### Fractionation of the **Cu**_***n***_**SeO**_**4**_ (*n* = 28–33) Nanojar Mixture

**Cu**_***n***_**SeO**_**4**_ (0.2150 g) was dissolved in toluene (3.3 mL) by sonication for 1 min to yield a deep-blue solution. After standing for 48 h at room temperature, the dark-blue precipitate formed was filtered out, washed with 5 mL of cold toluene (the slightly blue washing solution was collected separately from the filtrate), and dried under high vacuum (0.0980 g). The filtrate was evaporated under reduced pressure and dried under high vacuum. The ESI-MS(−) spectra of the two fractions are shown in Figure S30.

### Reaction of **Cu**_***n***_**EO**_**4**_ (E = Se, Mo, W; *n* = 28–34) with NH_3_

**Cu**_***n***_**EO**_**4**_ (0.100 g) was dissolved in THF (25 mL) and gaseous NH_3_ was bubbled through the resulting solution for 20 min. Then, the flask was stoppered and left standing. After 10 weeks, the solution was filtered and the solvent was evaporated to give a dark blue residue. ESI-MS(−) spectra of the products are shown in Figures S31–S33.

### Competitive Anion Binding under Heterogeneous Conditions

**Cu**_***n***_**EO**_**4**_ (E = Se, Mo, W; *n* = 28–32) (0.0400 g, 7.53–7.69 μmol) was dissolved in 2-MeTHF (5 mL) to give a clear, blue solution, which was then cannulated over a solution of Ba(NO_3_)_2_ (0.0040 g, 15 μmol) in water (5 mL). After stirring vigorously for 1 h, the aqueous and organic layers were separated and the 2-MeTHF layer was analyzed by ESI-MS (Figures S34–S36).

### Competitive Anion Binding under Homogeneous Conditions

**Cu**_***n***_**EO**_**4**_ (E = Se, Mo, W; *n* = 28–32) (0.0400 g, 7.53–7.69 μmol) and Ba(DOSS)_2_ (0.0076 g, 7.8 μmol) were dissolved in 2-MeTHF (10 mL) to give a clear, blue solution. The solution was stirred for 1 h and then it was analyzed by ESI-MS (Figures S34–S36).

### Extraction of EO_4_^2–^ (E = Se, Mo, W) from Water into THF

To a solution of Na_2_SeO_4_, Na_2_MoO_4_·2H_2_O or Na_2_WO_4_·2H_2_O (4.30 mmol) in H_2_O (10 mL) were added NaOH (0.334 g, 8.35 mmol) and Bu_4_NOH (54% in H_2_O, 0.139 mL, 0.138 g, 0.287 mmol). The resulting solution was stirred together vigorously with a solution of Cu(NO_3_)_2_ 2.5H_2_O (1.000 g, 4.30 mmol) and pyrazole (0.293 g, 4.30 mmol) in THF (10 mL) in a sealed flask. The deep-blue THF layer was separated from the aqueous layer (which contained a brown suspension) using a separatory funnel, then it was filtered and evaporated. The blue solid residue was washed with H_2_O (150 mL) and dried in vacuum. Yield: 0.503–0.582 g (69–79% based on copper). The ESI-MS(−) spectra of the products are shown in Figures S37–S39.

### Mass Spectrometry

Mass spectrometric analysis of the nanojars was performed with a Waters Synapt G1 HDMS or Q-TOF Micro instrument, using electrospray ionization (ESI). 10^–4^ to 10^–5^ M solutions were prepared in CH_3_CN using either solids or aliquots taken from solutions. Samples were infused by a syringe pump at 5 μL/min and nitrogen was supplied as the nebulizing gas at 500 L/h. The electrospray capillary voltage was set to −2.5 or +2.5 kV, respectively, with a desolvation temperature of 110 °C. Unless stated otherwise, the sampling and extraction cones were maintained at 40 and 4.0 V, respectively, at 80 °C. *m*/*z* values indicated are averages of isotopic distributions.

### X-ray Crystallography

Single-crystals were grown at room temperature by *n*-pentane (**1**) or hexanes (**4** and **6**) vapor diffusion into a chlorobenzene solution, by vapor diffusion of *n*-heptane into a 1,2-dichlorobenzene solution (**2**), by *n*-pentane vapor diffusion into a nitrobenzene/bromobenzene solution (**3**), and by hexanes vapor diffusion into a 1,2-dichlorobenzene solution of **Cu**_**n**_**EO**_**4**_ (E = Se, Mo or W; *n* = 28–34). Tribenzylmethylammonium nitrate (in the case of **3**–**5**) and methyltriphenylphosphonium bromide (in the case of **5**) were employed as additives intended to provide additional counterions that may lead to better crystals. Once removed from the mother liquor, the crystals are extremely sensitive to solvent loss at ambient conditions and were quickly mounted under a cryostream (150 K) to prevent decomposition. X-ray diffraction data were collected from a single-crystal mounted atop a MiTeGen micromesh mount under Fomblin or polybutene oil with a Bruker AXS D8 Quest diffractometer equipped with a Photon II charge-integrating and photon counting pixel array detector (CPAD) using graphite-monochromated Mo-*K*_α_ (λ = 0.71073 Å) radiation (for **3**) or a Bruker AXS D8 Quest diffractometer with Photon III C14 CPAD using Cu *K*_α_ (λ = 1.54178 Å) radiation monochromated using X-ray mirror optics (for **1**, **2**, **4**–**6**). The data were collected using APEX4,^79^ integrated using SAINT^80^ and scaled and corrected for absorption and other effects using SADABS.^81^ The structures were solved by employing direct or dual methods using ShelXS^82^ or ShelXT^83^ and refined by full-matrix least-squares on *F*^2^ using ShelXL^84^ with ShelXle as the graphical interface.^85^ Refinement details and thermal ellipsoid plots (Figures S40–S45) are provided in the Supporting Information. Crystallographic figures were generated using CrystalMaker^86^ or Mercury (structural overlays),^87^ and supramolecular features (angles and distances) were measured using OLEX2.^88^

## Supporting Information Available

Additional data for mass spectrometry, X-ray crystallography and ^1^H NMR spectroscopy (PDF)
