# Supplementary material for: Supramolecular Entrapping and Extraction of Selenate, Molybdate and Tungstate Ions from Water by Nanojars
Source: Inorg Chem. 2025 Jan 3;64(2):1048–63. doi: 10.1021/acs.inorgchem.4c04544 (PMC11752490; doi:10.1021/acs.inorgchem.4c04544)
Supplement: Supplementary file 1 — ic4c04544_si_001.pdf [file ic4c04544_si_001.pdf]

Supporting Information for

Supramolecular Entrapping and Extraction of Selenate, Molybdate and Tungstate Ions from Water by Nanojars

Wisam A. Al Isawi,[a] Angel S. Philip,[a] Pooja Singh,[a] Matthias Zeller[b] and Gellert Mezei[a*]

[a] *Department of Chemistry, Western Michigan University, Kalamazoo, Michigan 49008, USA*

[b] *Department of Chemistry, Purdue University, West Lafayette, Indiana 47907, USA*

[*] Corresponding author. Email: gellert.mezei@wmich.edu

CONTENTS **PAGE**

1. Synthesis S2–S3
2. Mass spectrometric data (Tables S1–S3; Figures S1–S39) S3–S26
3. X-ray crystallographic data and refinement details (Tables S4–S25; Figures S40–S58) S27–S70
4. NMR spectroscopic data (Table S26; Figures S59–S69) S71–S87
5. References S88

1. SYNTHESIS

While the reaction of $CuSO_4$, pyrazole, NaOH and Bu_4NOH (1:1:1.93:0.07 molar ratio) in tetrahydrofuran (THF) produces a clean $\mathbf{Cu_nSO_4}$ (n = 27–33) nanojar mixture,[1] ESI-MS(–) indicates that an analogous reaction with $CuSeO_4$ under identical conditions yields a $\mathbf{Cu_nSeO_4}$ (n = 27–33) nanojar mixture mixed with $\mathbf{Cu_nCO_3}$ (n = 27, 29, 31) impurities (Figure S1). With $CuSO_4$, a similar carbonate-contaminated nanojar mixture was obtained when KOH (which contains K_2CO_3) was used instead of NaOH. Yet, the addition of $PbSO_4$ to the reaction mixture led to a carbonate-free $\mathbf{Cu_nSO_4}$ mixture.[2] Similarly, $PbSeO_4$ scavenges carbonate from the reaction producing $\mathbf{Cu_nSeO_4}$, but leads to additional, hitherto unidentified lower-nuclearity species in the *m/z* 800–1300 window (Figures S2 and S3). A reaction employing $Cu(NO_3)_2$, pyrazole, NaOH, Bu_4NOH and Na_2SeO_4 (1:1:1.93:0.07:1 molar ratio) also leads to a CO_3-contaminated nanojar mixture (Figure S4). Substituting Na_2SeO_4 by $(Bu_4N)_2SeO_4$ (prepared from Bu_4NOH and H_2SeO_4 in a 2:1 molar ratio), and NaOH by Bu_4NOH does eliminate the amount of carbonate in the product almost completely, but various other nanojar peaks, possibly corresponding to substituted species/clusters, as well as low-nuclearity peaks are also obtained (Figure S5). Running a similar reaction with Et_3N instead of Bu_4NOH as the base completely eliminates carbonate from the reaction product, but lower-nuclearity species are again observed besides nanojars (Figure S6). ESI-MS of the reaction of $CuSeO_4$ with pyrazole and Et_3N or ${}^{n}Bu_3N$ (1:1:2 molar ratio) in DMF with or without added $(Bu_4N)NO_3$ (0.07 mol) displays small amounts of low-nuclearity species and/or $\mathbf{Cu_nCO_3}$ (Figures S7–S9). When R_3N (R = Et, ${}^{n}Bu$) is used as the base, R_3NH^+ forms as a byproduct which is acidic; therefore, capped-nanojars in which a $[Cu_3(\mu_3\text{-OH})(\mu\text{-pz})_3L_3]^{2+}$ ($\mathbf{Cu_3}$; L = solvent or an anion) "cap" is attached to the $[SeO_4{\subset}\{Cu(\mu\text{-OH})(\mu\text{-pz})\}_n]^{2-}$ nanojar is likely to form, as observed before with CO_3^{2-} as the entrapped anion.[3] In a final attempt to obtain carbonate-free $\mathbf{Cu_nSeO_4}$ nanojars by the direct reaction of a Cu^{2+} source with pyrazole and a base, copper(II) ethoxide (which combines the Cu^{2+} source and the base) was reacted with pyrazole and $(Bu_4N)_2SeO_4$ (1:1:0.036 molar ratio) in wet THF. No carbonate is observed in the resulting nanojar mixture, but low-nuclearity species are again present (Figure S10).

When excess $(Bu_4N)_2SeO_4$ is used, substituted nanojar species also form, in which a pz^- unit is apparently replaced by SeO_4^{2-} leading to an increase in the charge on the nanojar to 3–. In this case, ESI-MS(–) shows peaks that have tentatively been assigned to $[SeO_4{\subset}\{Cu_n(OH)_n(pz)_{n-}$

$_1(SeO_4)\}]^{3-}$ (n = 28–32) species and their Bu_4N^+ adducts, $[(Bu_4N)SeO_4{\subset}\{Cu_n(OH)_n(pz)_{n-1}(SeO_4)\}]^{2-}$ (Table S1, Figure S13). In addition, smaller peaks tentatively assigned to $[(Bu_4N)(HSeO_4)SeO_4{\subset}\{Cu_n(OH)_n(pz)_{n-2}(SeO_4)(HSeO_4)]^{3-}$ and $[(Bu_4N)_2(HSeO_4)SeO_4{\subset}\{Cu_n(OH)_n(pz)_{n-2}(SeO_4)(HSeO_4)]^{2-}$ clusters are also detected.

2. MASS SPECTROMETRIC DATA

Table S1. Selenate-substituted nanojar species observed by ESI-MS(−).

Parent nanojar (m/z)	Selenate-substituted analogues (m/z)
$[SeO_4{\subset}\{Cu(OH)(pz)\}_{28}]^{2-}$ (2138.22)	$[SeO_4{\subset}\{Cu_{28}(OH)_{28}(pz)_{27}(SeO_4)\}]^{3-}$ (1450.78)<br>$[(Bu_4N)SeO_4{\subset}\{Cu_{28}(OH)_{28}(pz)_{27}(SeO_4)\}]^{2-}$ (2297.40)<br>$[(Bu_4N)(HSeO_4)SeO_4{\subset}\{Cu_{28}(OH)_{28}(pz)_{26}(SeO_4)(HSeO_4)]^{3-}$ (1605.23)<br>$[(Bu_4N)_2(HSeO_4)SeO_4{\subset}\{Cu_{28}(OH)_{28}(pz)_{26}(SeO_4)(HSeO_4)]^{2-}$ (2529.08)
$[SeO_4{\subset}\{Cu(OH)(pz)\}_{29}]^{2-}$ (2212.03)	$[SeO_4{\subset}\{Cu_{29}(OH)_n(pz)_{28}(SeO_4)\}]^{3-}$ (1499.99)<br>$[(Bu_4N)SeO_4{\subset}\{Cu_{29}(OH)_{29}(pz)_{28}(SeO_4)\}]^{2-}$ (2371.22)<br>$[(Bu_4N)(HSeO_4)SeO_4{\subset}\{Cu_{29}(OH)_{29}(pz)_{27}(SeO_4)(HSeO_4)]^{3-}$ (1654.44)<br>$[(Bu_4N)_2(HSeO_4)SeO_4{\subset}\{Cu_{29}(OH)_{29}(pz)_{27}(SeO_4)(HSeO_4)]^{2-}$ (2602.89)
$[SeO_4{\subset}\{Cu(OH)(pz)\}_{30}]^{2-}$ (2285.84)	$[SeO_4{\subset}\{Cu_{30}(OH)_{30}(pz)_{29}(SeO_4)\}]^{3-}$ (1549.19)<br>$[(Bu_4N)SeO_4{\subset}\{Cu_{30}(OH)_{30}(pz)_{29}(SeO_4)\}]^{2-}$ (2445.03)<br>$[(Bu_4N)(HSeO_4)SeO_4{\subset}\{Cu_{30}(OH)_{30}(pz)_{28}(SeO_4)(HSeO_4)]^{3-}$ (1703.65)<br>$[(Bu_4N)_2(HSeO_4)SeO_4{\subset}\{Cu_{30}(OH)_{30}(pz)_{28}(SeO_4)(HSeO_4)]^{2-}$ (2676.70)
$[SeO_4{\subset}\{Cu(OH)(pz)\}_{31}]^{2-}$ (2359.66)	$[SeO_4{\subset}\{Cu_{31}(OH)_{31}(pz)_{30}(SeO_4)\}]^{3-}$ (1598.40)<br>$[(Bu_4N)SeO_4{\subset}\{Cu_{31}(OH)_{31}(pz)_{30}(SeO_4)\}]^{2-}$ (2518.84)<br>$[(Bu_4N)(HSeO_4)SeO_4{\subset}\{Cu_{31}(OH)_{31}(pz)_{29}(SeO_4)(HSeO_4)]^{3-}$ (1752.85)<br>$[(Bu_4N)_2(HSeO_4)SeO_4{\subset}\{Cu_{31}(OH)_{31}(pz)_{29}(SeO_4)(HSeO_4)]^{2-}$ (2750.52)
$[SeO_4{\subset}\{Cu(OH)(pz)\}_{32}]^{2-}$ (2433.47)	$[SeO_4{\subset}\{Cu_{32}(OH)_{32}(pz)_{31}(SeO_4)\}]^{3-}$ (1647.61)<br>$[(Bu_4N)SeO_4{\subset}\{Cu_{32}(OH)_{32}(pz)_{31}(SeO_4)\}]^{2-}$ (2592.65)<br>$[(Bu_4N)(HSeO_4)SeO_4{\subset}\{Cu_{32}(OH)_{32}(pz)_{30}(SeO_4)(HSeO_4)]^{3-}$ (1802.06)<br>$[(Bu_4N)_2(HSeO_4)SeO_4{\subset}\{Cu_{32}(OH)_{32}(pz)_{30}(SeO_4)(HSeO_4)]^{2-}$ (2824.33)

Table S2. Selenate-nanojar species observed by ESI-MS(−) at different cone voltages (40 V – parent nanojar; 100 V – daughter nanojars).

Parent nanojar (m/z)	Daughter nanojars (m/z)
$[SeO_4\subset\{Cu(OH)(pz)\}_{28}]^{2-}$ (2138.22)	$[SeO_4\subset\{Cu_{25}O_{15}(pz)_{20}\}]^{2-}$ (1656.51)<br>$[SeO_4\subset\{Cu_{25}O_{14}(pz)_{22}\}]^{2-}$ (1715.58)<br>$[SeO_4\subset\{Cu_{25}O_{13}(pz)_{24}\}]^{2-}$ (1774.65)<br>$[SeO_4\subset\{Cu_{25}O_{12}(pz)_{26}\}]^{2-}$ (1833.73)
$[SeO_4\subset\{Cu(OH)(pz)\}_{29}]^{2-}$ (2212.03)	$[SeO_4\subset\{Cu_{26}O_{16}(pz)_{20}\}]^{2-}$ (1696.28)<br>$[SeO_4\subset\{Cu_{26}O_{15}(pz)_{22}\}]^{2-}$ (1755.36)<br>$[SeO_4\subset\{Cu_{26}O_{14}(pz)_{24}\}]^{2-}$ (1814.43)<br>$[SeO_4\subset\{Cu_{26}O_{13}(pz)_{26}\}]^{2-}$ (1873.50)
$[SeO_4\subset\{Cu(OH)(pz)\}_{30}]^{2-}$ (2285.84)	$[SeO_4\subset\{Cu_{27}O_{16}(pz)_{22}\}]^{2-}$ (1795.13)<br>$[SeO_4\subset\{Cu_{27}O_{15}(pz)_{24}\}]^{2-}$ (1854.20)<br>$[SeO_4\subset\{Cu_{27}O_{14}(pz)_{26}\}]^{2-}$ (1913.27)<br>$[SeO_4\subset\{Cu_{27}O_{13}(pz)_{28}\}]^{2-}$ (1972.34)
$[SeO_4\subset\{Cu(OH)(pz)\}_{31}]^{2-}$ (2359.66)	$[SeO_4\subset\{Cu_{28}O_{17}(pz)_{22}\}]^{2-}$ (1834.90)<br>$[SeO_4\subset\{Cu_{28}O_{16}(pz)_{24}\}]^{2-}$ (1893.97)<br>$[SeO_4\subset\{Cu_{28}O_{15}(pz)_{26}\}]^{2-}$ (1953.04)<br>$[SeO_4\subset\{Cu_{28}O_{14}(pz)_{28}\}]^{2-}$ (2012.11)
$[SeO_4\subset\{Cu(OH)(pz)\}_{32}]^{2-}$ (2433.47)	$[SeO_4\subset\{Cu_{29}O_{18}(pz)_{22}\}]^{2-}$ (1874.67)<br>$[SeO_4\subset\{Cu_{29}O_{17}(pz)_{24}\}]^{2-}$ (1933.74)<br>$[SeO_4\subset\{Cu_{29}O_{16}(pz)_{26}\}]^{2-}$ (1992.82)<br>$[SeO_4\subset\{Cu_{29}O_{15}(pz)_{28}\}]^{2-}$ (2051.89)

Table S3. Molybdate- and tungstate-nanojar species observed by ESI-MS(−) at different cone voltages (40 V – parent nanojar; 100 V – daughter nanojars).

Parent nanojar (m/z)	Daughter nanojars (m/z)	Daughter nanojars (m/z)
$[MoO_4\subset\{Cu(OH)(pz)\}_{28}]^{2-}$<br>(2146.71)	$[MoO_4\subset\{Cu_{25}O_{15}(pz)_{20}\}]^{2-}$ (1665.00)<br>$[MoO_4\subset\{Cu_{25}O_{14}(pz)_{22}\}]^{2-}$ (1724.07)<br>$[MoO_4\subset\{Cu_{25}O_{13}(pz)_{24}\}]^{2-}$ (1783.14)<br>$[MoO_4\subset\{Cu_{25}O_{12}(pz)_{26}\}]^{2-}$ (1842.22)	
$[MoO_4\subset\{Cu(OH)(pz)\}_{31}]^{2-}$<br>(2368.15)	$[MoO_4\subset\{Cu_{28}O_{16}(pz)_{24}\}]^{2-}$ (1902.46)<br>$[MoO_4\subset\{Cu_{28}O_{15}(pz)_{26}\}]^{2-}$ (1961.53)<br>$[MoO_4\subset\{Cu_{28}O_{14}(pz)_{28}\}]^{2-}$ (2020.60)<br>$[MoO_4\subset\{Cu_{28}O_{13}(pz)_{30}\}]^{2-}$ (2079.68)	
$[MoO_4\subset\{Cu(OH)(pz)\}_{32}]^{2-}$<br>(2441.96)	$[MoO_4\subset\{Cu_{29}O_{17}(pz)_{24}\}]^{2-}$ (1942.23)<br>$[MoO_4\subset\{Cu_{29}O_{16}(pz)_{26}\}]^{2-}$ (2001.31)<br>$[MoO_4\subset\{Cu_{29}O_{15}(pz)_{28}\}]^{2-}$ (2060.38)<br>$[MoO_4\subset\{Cu_{29}O_{14}(pz)_{30}\}]^{2-}$ (2119.45)	$[MoO_4\subset\{Cu_{29}O_{17}(OH)(pz)_{23}\}]^{2-}$ (1917.20)<br>$[MoO_4\subset\{Cu_{29}O_{16}(OH)(pz)_{25}\}]^{2-}$ (1976.27)<br>$[MoO_4\subset\{Cu_{29}O_{15}(OH)(pz)_{27}\}]^{2-}$ (2035.35)<br>$[MoO_4\subset\{Cu_{29}O_{14}(OH)(pz)_{29}\}]^{2-}$ (2094.42)
$[WO_4\subset\{Cu(OH)(pz)\}_{28}]^{2-}$<br>(2190.65)	$[WO_4\subset\{Cu_{25}O_{15}(pz)_{20}\}]^{2-}$ (1708.95)<br>$[WO_4\subset\{Cu_{25}O_{14}(pz)_{22}\}]^{2-}$ (1768.02)<br>$[WO_4\subset\{Cu_{25}O_{13}(pz)_{24}\}]^{2-}$ (1827.09)<br>$[WO_4\subset\{Cu_{25}O_{12}(pz)_{26}\}]^{2-}$ (1886.16)	
$[WO_4\subset\{Cu(OH)(pz)\}_{31}]^{2-}$<br>(2412.09)	$[WO_4\subset\{Cu_{28}O_{16}(pz)_{24}\}]^{2-}$ (1946.41)<br>$[WO_4\subset\{Cu_{28}O_{15}(pz)_{26}\}]^{2-}$ (2005.48)<br>$[WO_4\subset\{Cu_{28}O_{14}(pz)_{28}\}]^{2-}$ (2064.55)<br>$[WO_4\subset\{Cu_{28}O_{13}(pz)_{30}\}]^{2-}$ (2123.62)	
$[WO_4\subset\{Cu(OH)(pz)\}_{32}]^{2-}$<br>(2485.90)	$[WO_4\subset\{Cu_{29}O_{17}(pz)_{24}\}]^{2-}$ (1986.18)<br>$[WO_4\subset\{Cu_{29}O_{16}(pz)_{26}\}]^{2-}$ (2045.25)<br>$[WO_4\subset\{Cu_{29}O_{15}(pz)_{28}\}]^{2-}$ (2104.32)<br>$[WO_4\subset\{Cu_{29}O_{14}(pz)_{30}\}]^{2-}$ (2163.39)	$[WoO_4\subset\{Cu_{29}O_{17}(OH)(pz)_{23}\}]^{2-}$ (1961.15)<br>$[WoO_4\subset\{Cu_{29}O_{16}(OH)(pz)_{25}\}]^{2-}$ (2020.22)<br>$[WoO_4\subset\{Cu_{29}O_{15}(OH)(pz)_{27}\}]^{2-}$ (2079.29)<br>$[WoO_4\subset\{Cu_{29}O_{14}(OH)(pz)_{29}\}]^{2-}$ (2138.36)

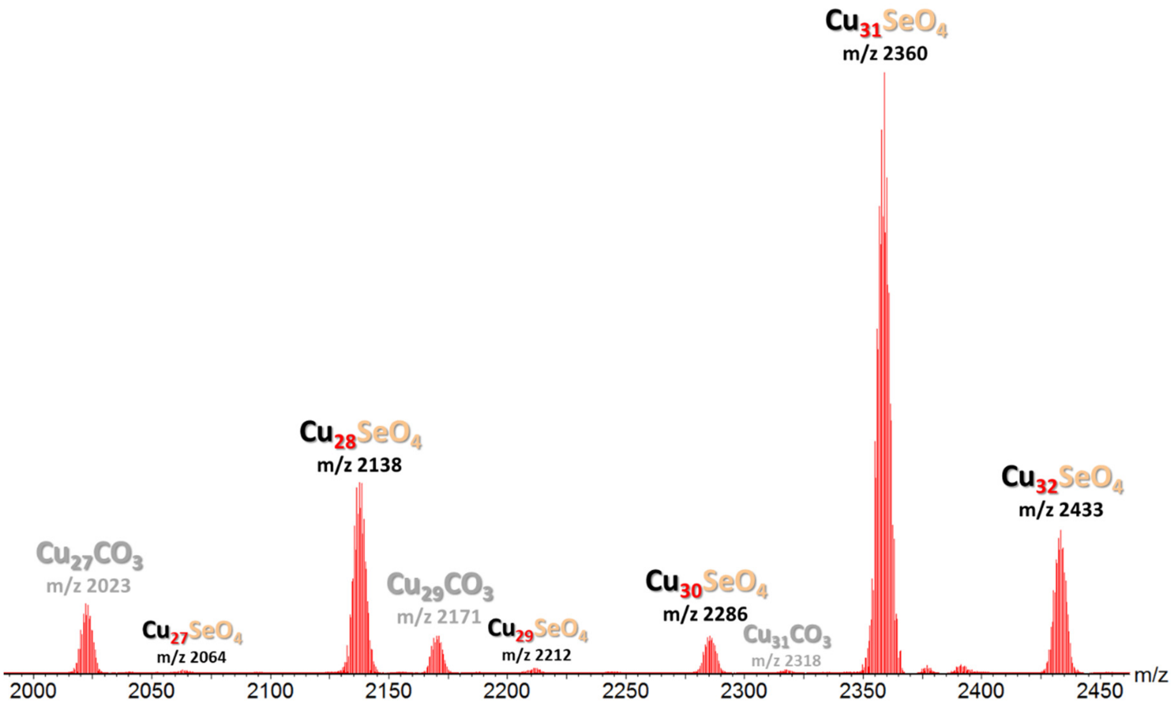

Figure S1. ESI-MS(−) spectrum (in CH_3CN) of the product of the reaction of $CuSeO_4{\cdot}5H_2O$ with pyrazole, NaOH and Bu_4NOH (1:1:1.93:0.07 molar ratio) in THF.

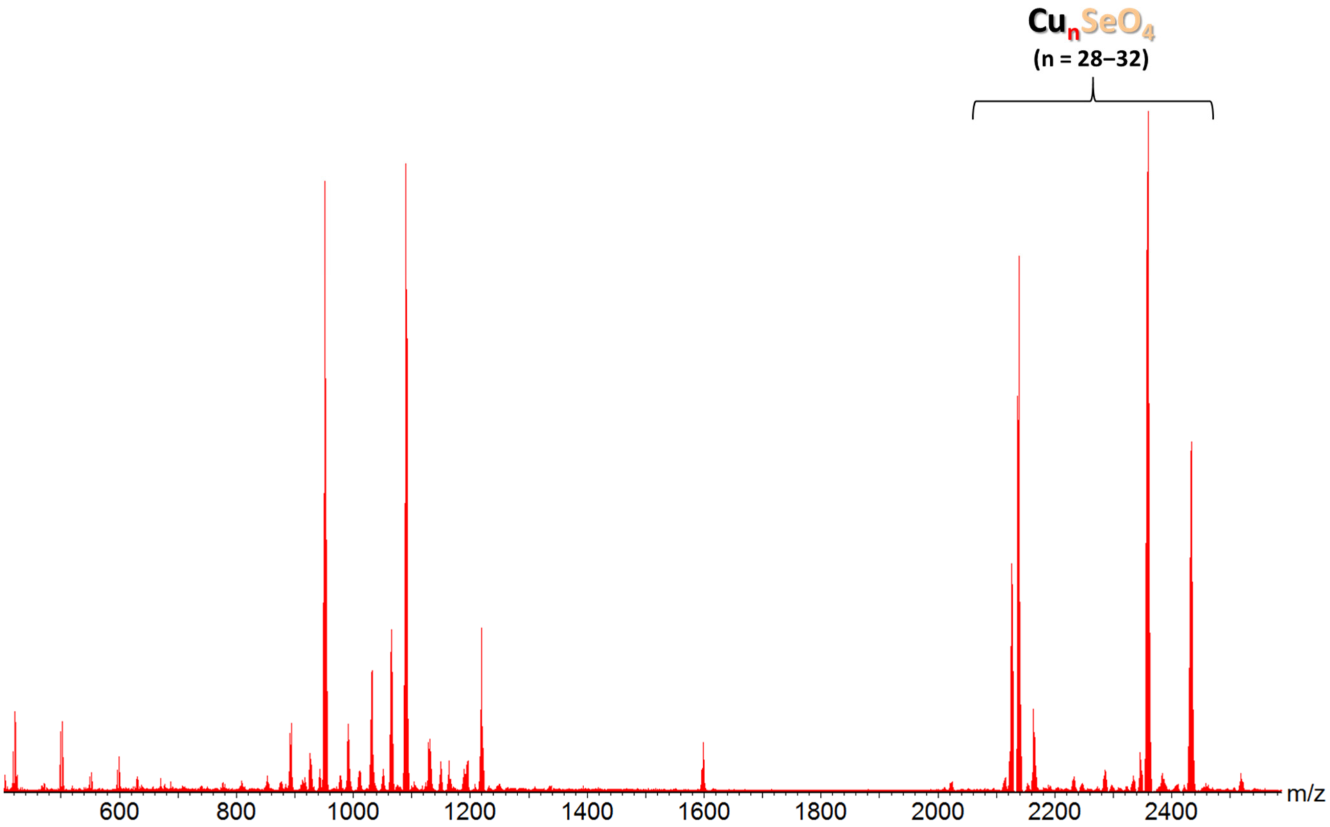

Figure S2. ESI-MS(−) spectrum (in CH_3CN) of the product of the reaction of $CuSeO_4{\cdot}5H_2O$ with pyrazole, NaOH, Bu_4NOH and $PbSeO_4$ (1:1:1.93:0.07:1 molar ratio) in THF.

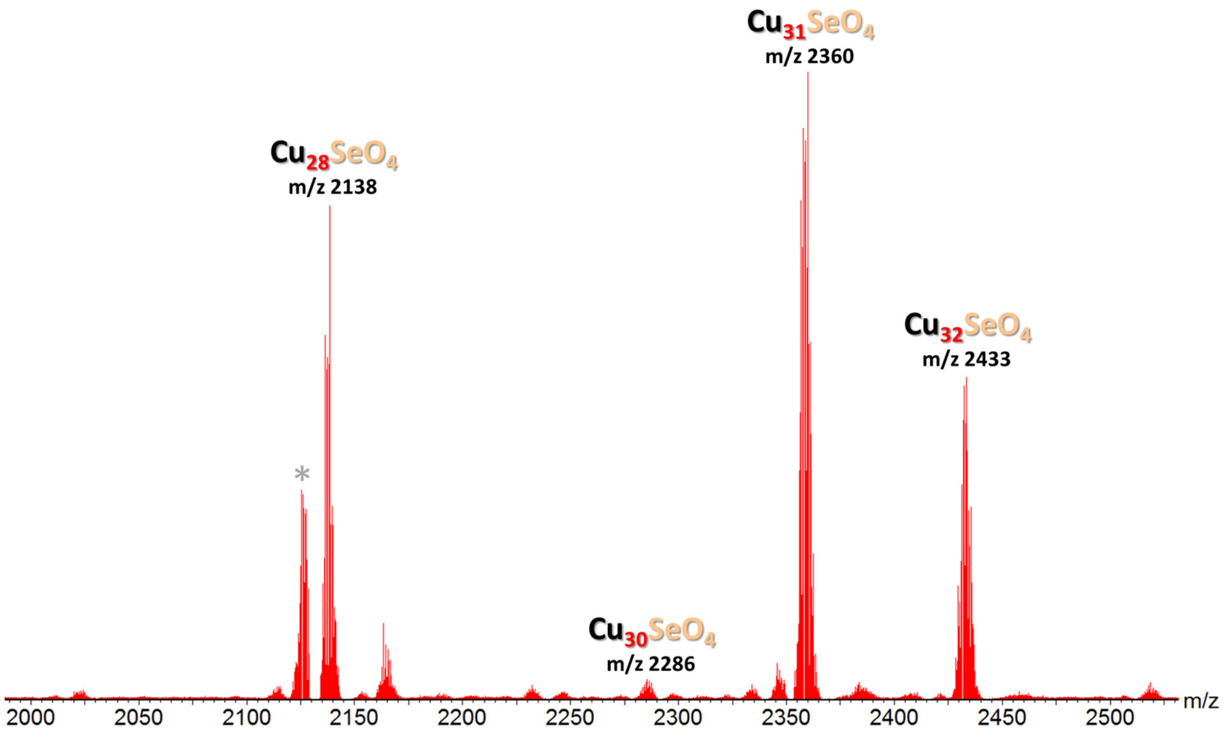

Figure S3. ESI-MS(−) spectrum (in CH_3CN) of the product of the reaction of $CuSeO_4 \cdot 5H_2O$ with pyrazole, NaOH, Bu_4NOH and $PbSeO_4$ (1:1:1.93:0.07:1 molar ratio) in THF (nanojar region). The asterisk denotes a formate-substituted species (see Figure S23).

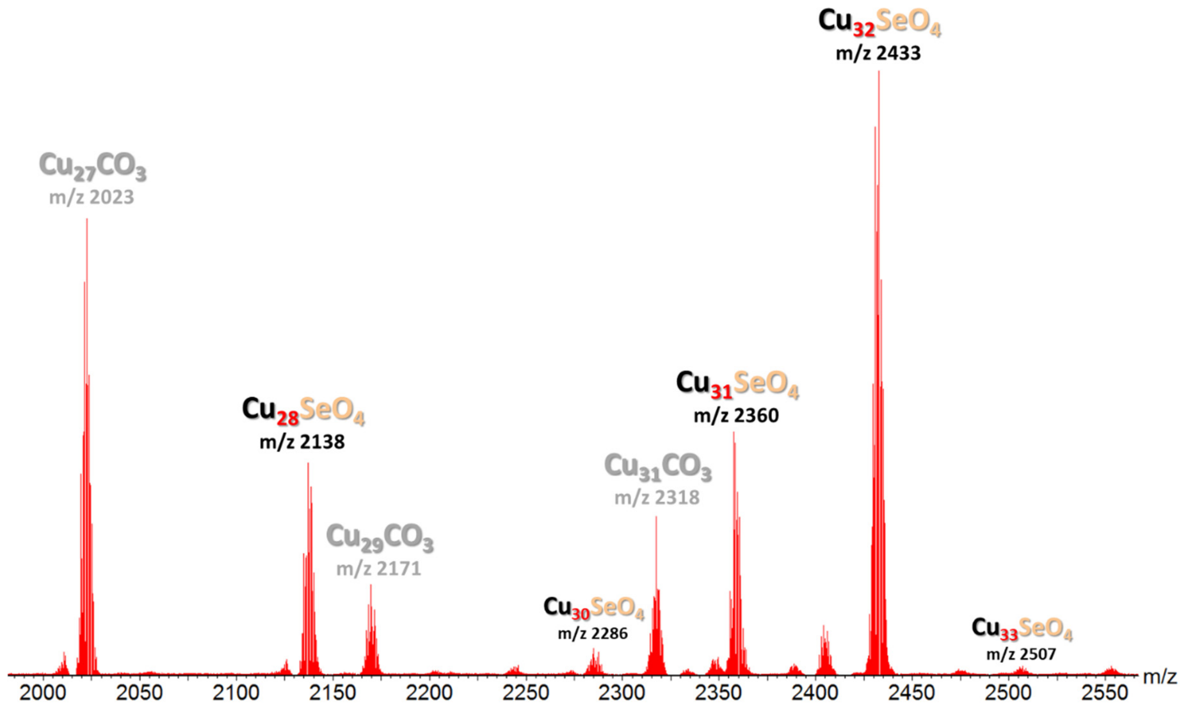

Figure S4. ESI-MS(−) spectrum (in CH_3CN) of the product of the reaction of $Cu(NO_3)_2 \cdot 2.5H_2O$, pyrazole, NaOH, Bu_4NOH and Na_2SeO_4 (1:1:1.93:0.07:1 molar ratio) in THF.

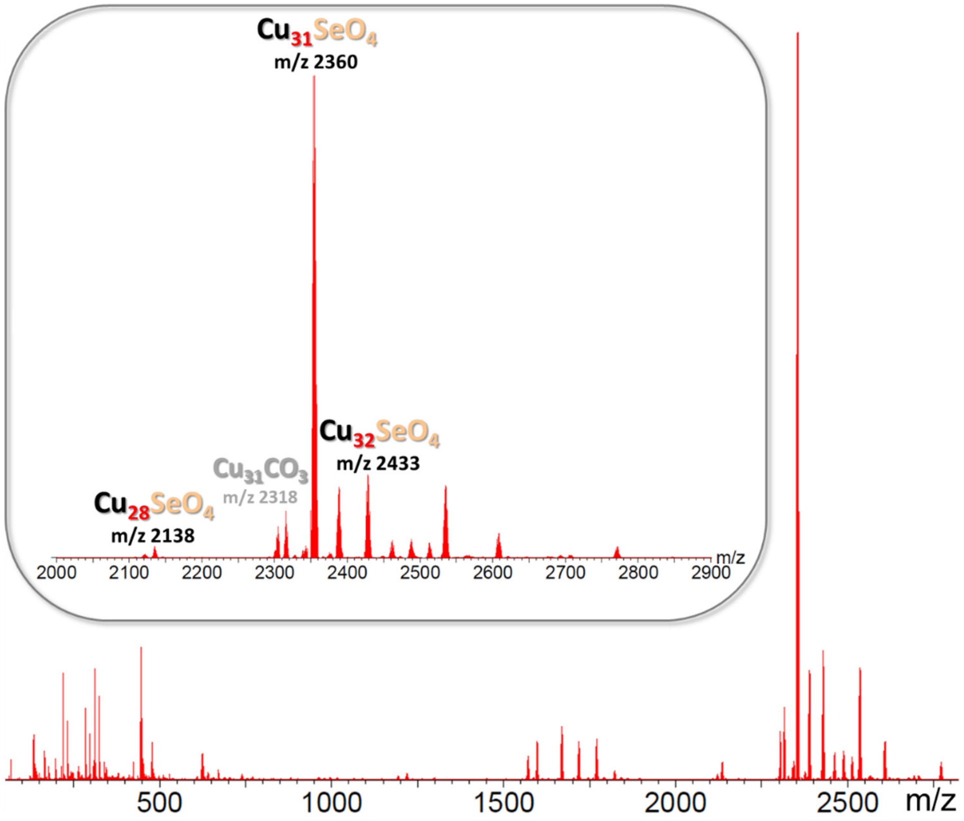

Figure S5. ESI-MS(−) spectrum (in CH_3CN) of the product of the reaction of $Cu(NO_3)_2{\cdot}2.5H_2O$, pyrazole, Bu_4NOH and $(Bu_4N)_2SeO_4$ (1:1:2:1 molar ratio) in THF under N_2.

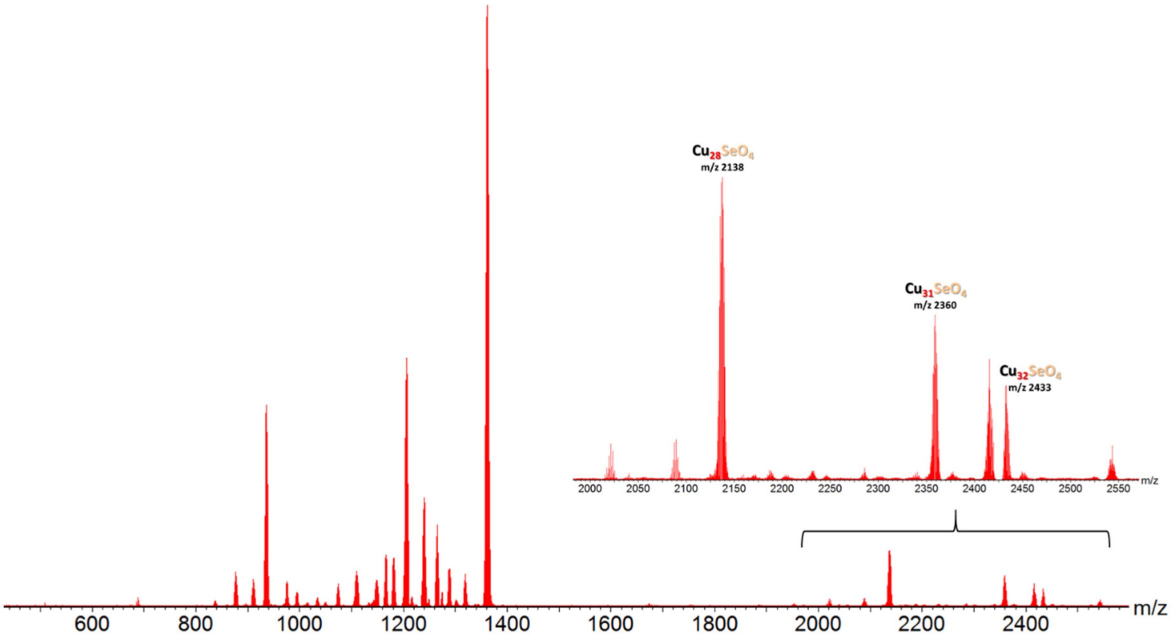

Figure S6. ESI-MS(−) spectrum (in CH_3CN) of the product of the reaction of $Cu(NO_3)_2{\cdot}2.5H_2O$, pyrazole, Et_3N and $(Bu_4N)_2SeO_4$ (1:1:2:1 molar ratio) in THF. The peaks at *m/z* 1953, 2021 and 2089 do not correspond to nanojars as they feature a 1− charge.

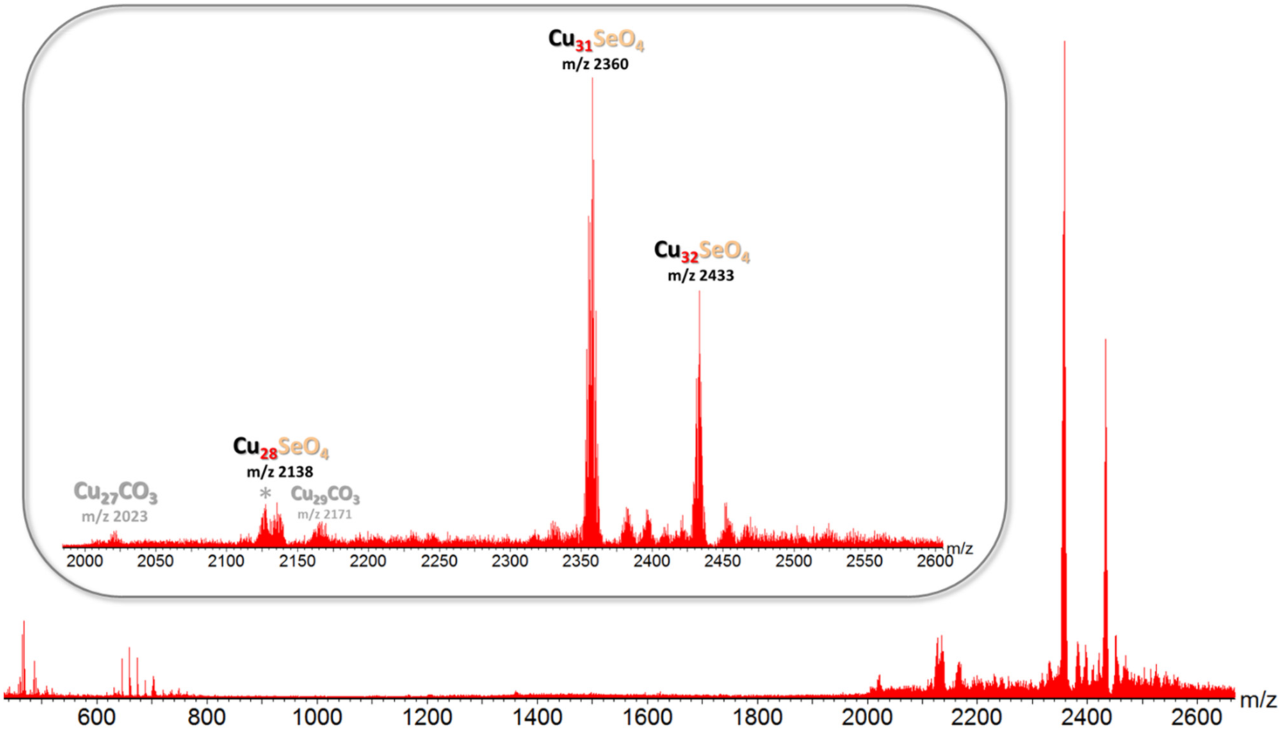

Figure S7. ESI-MS(−) spectrum (in CH_3CN) of the product of the reaction of $CuSeO_4{\cdot}5H_2O$, pyrazole and Et_3N (1:1:2 molar ratio) in DMF.

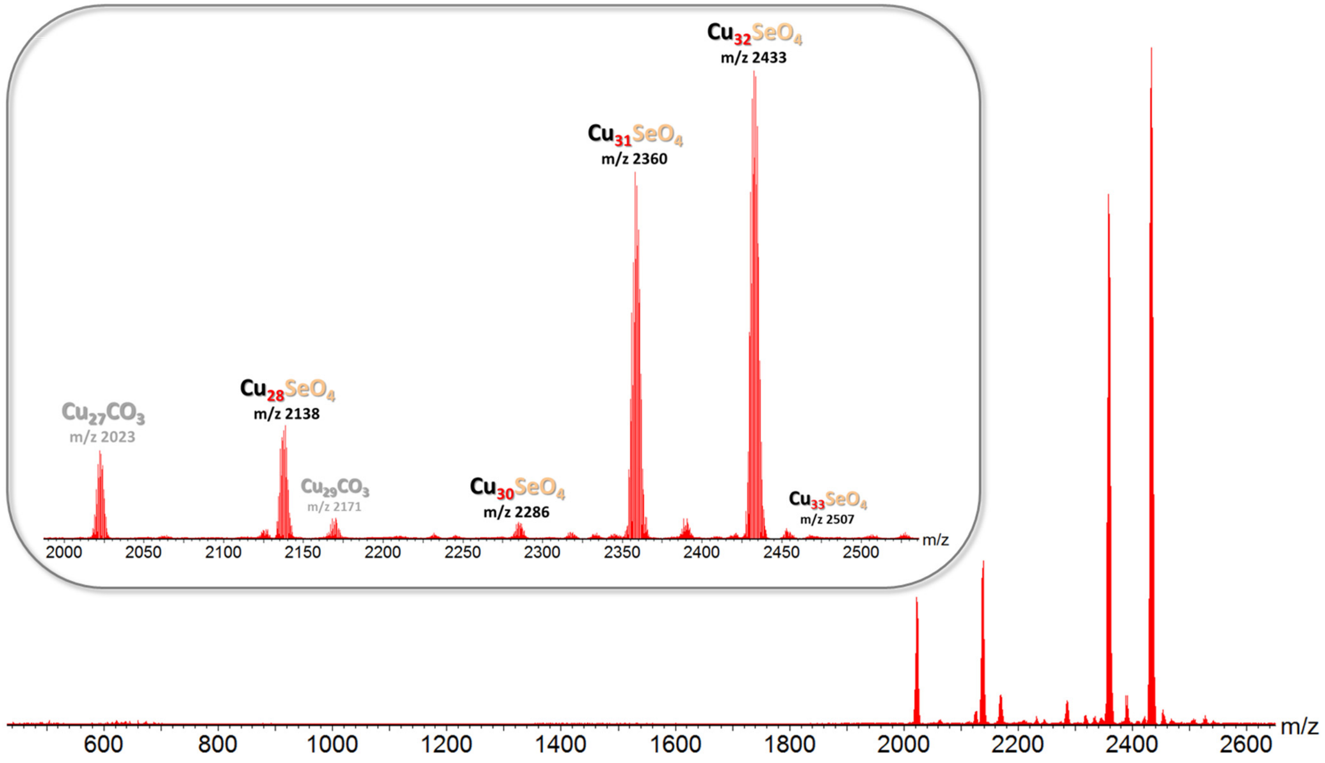

Figure S8. ESI-MS(−) spectrum (in CH_3CN) of the product of the reaction of $CuSeO_4{\cdot}5H_2O$, pyrazole, Et_3N and $(Bu_4N)NO_3$ (1:1:2:0.07 molar ratio) in DMF.

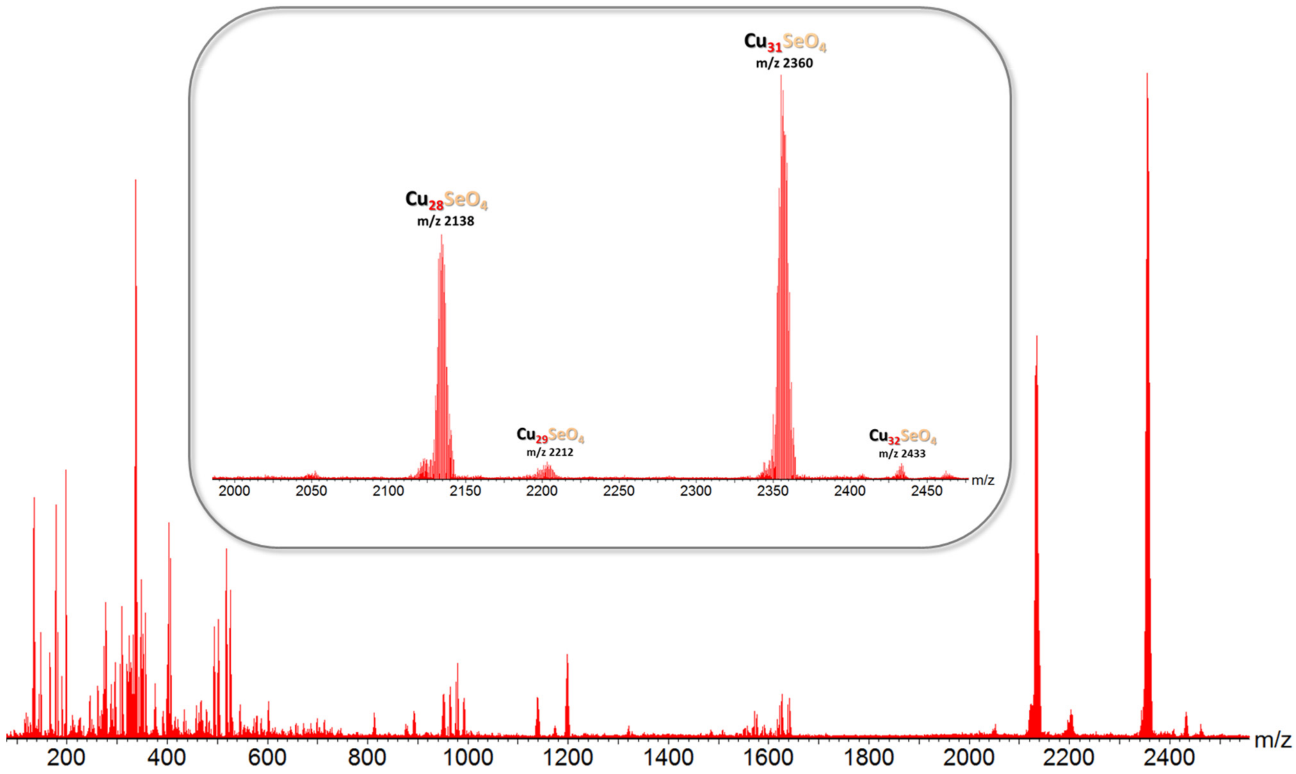

Figure S9. ESI-MS(−) spectrum (in CH_3CN) of the product of the reaction of $CuSeO_4{\cdot}5H_2O$, pyrazole and $^nBu_3N$ (1:1:2 molar ratio) in DMF.

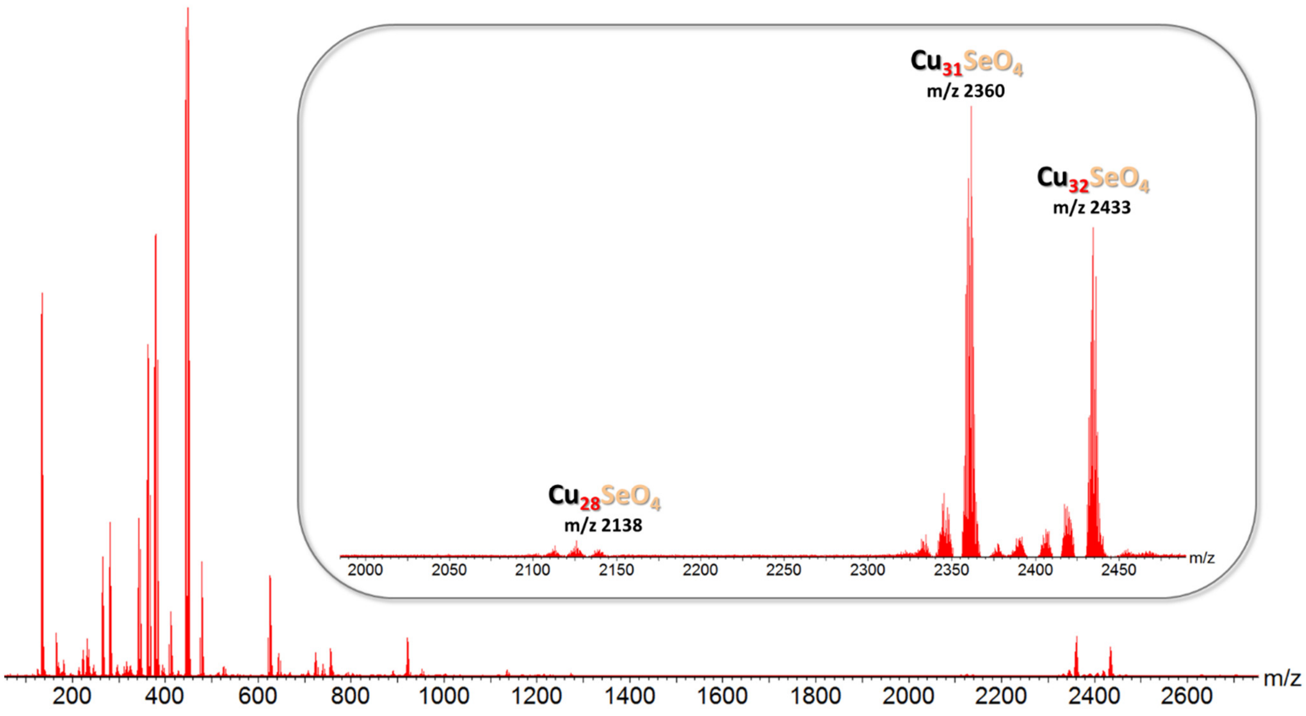

Figure S10. ESI-MS(−) spectrum (in CH_3CN) of the product of the reaction of $Cu(OEt)_2$, pyrazole and $(Bu_4N)_2SeO_4$ (1:1:0.036 molar ratio) in THF.

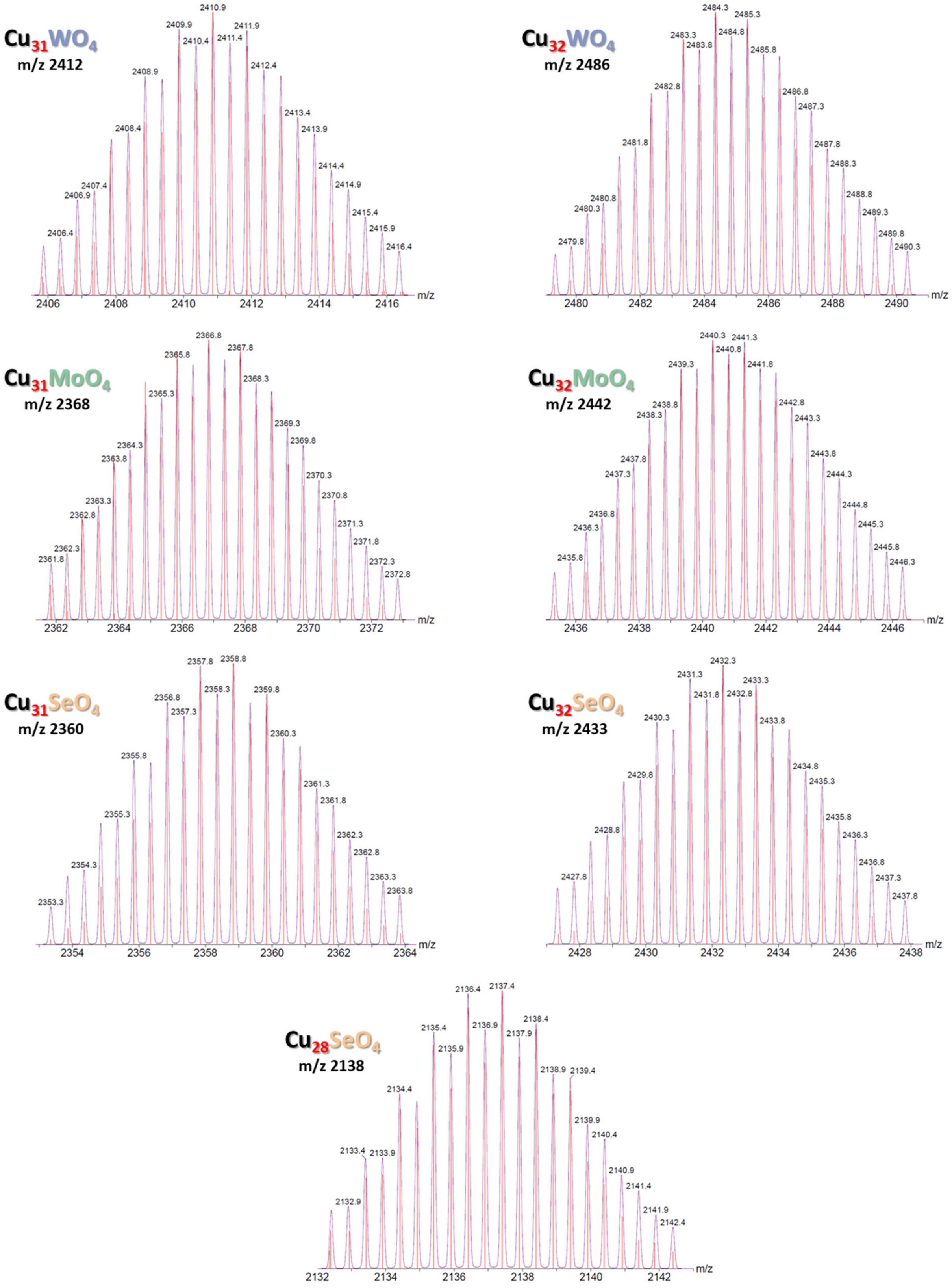

Figure S11. Isotopic distribution patterns observed (centroid) and predicted (continuum, *m/z* values shown) for the major selenate-, molybdate- and tungstate-entrapping nanojars.

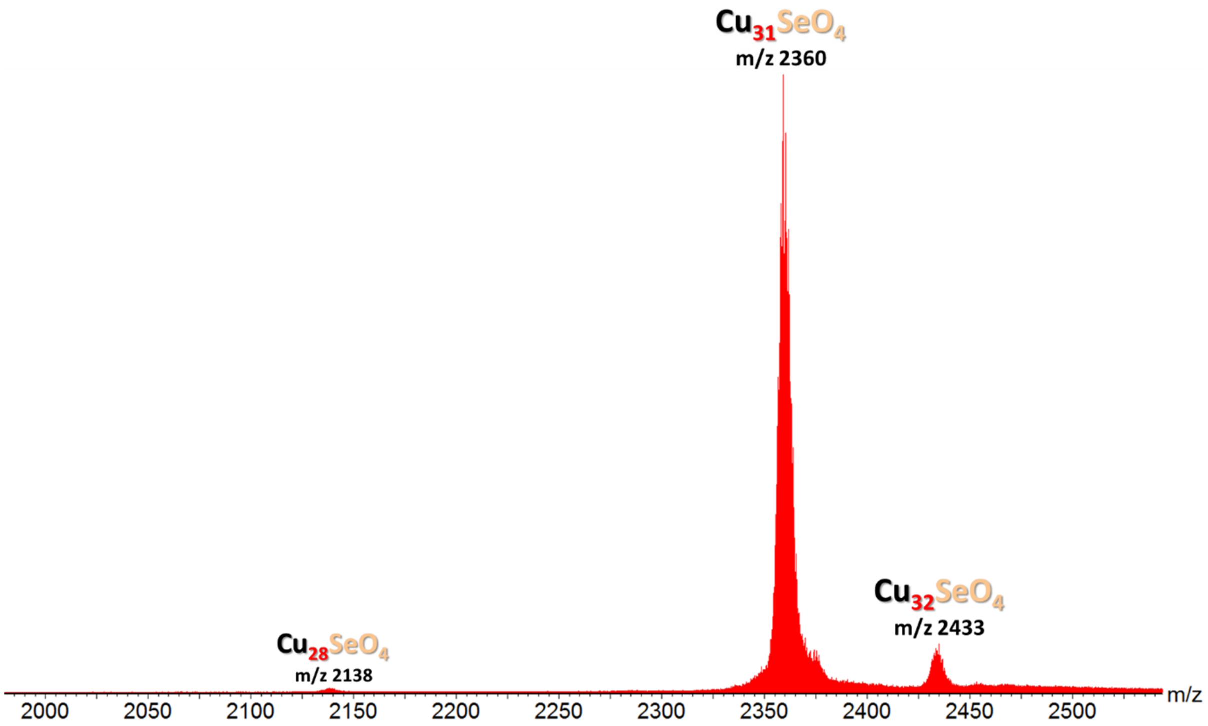

Figure S12. ESI-MS(−) spectrum (in CH_3CN) of the **Cu_nSeO_4** nanojar mixture obtained by the depolymerization of [*trans*-Cu^{II}(μ-OH)(μ-pz)]$_\infty$ in the presence of $(Bu_4N)_2SeO_4$ in refluxing chlorobenzene.

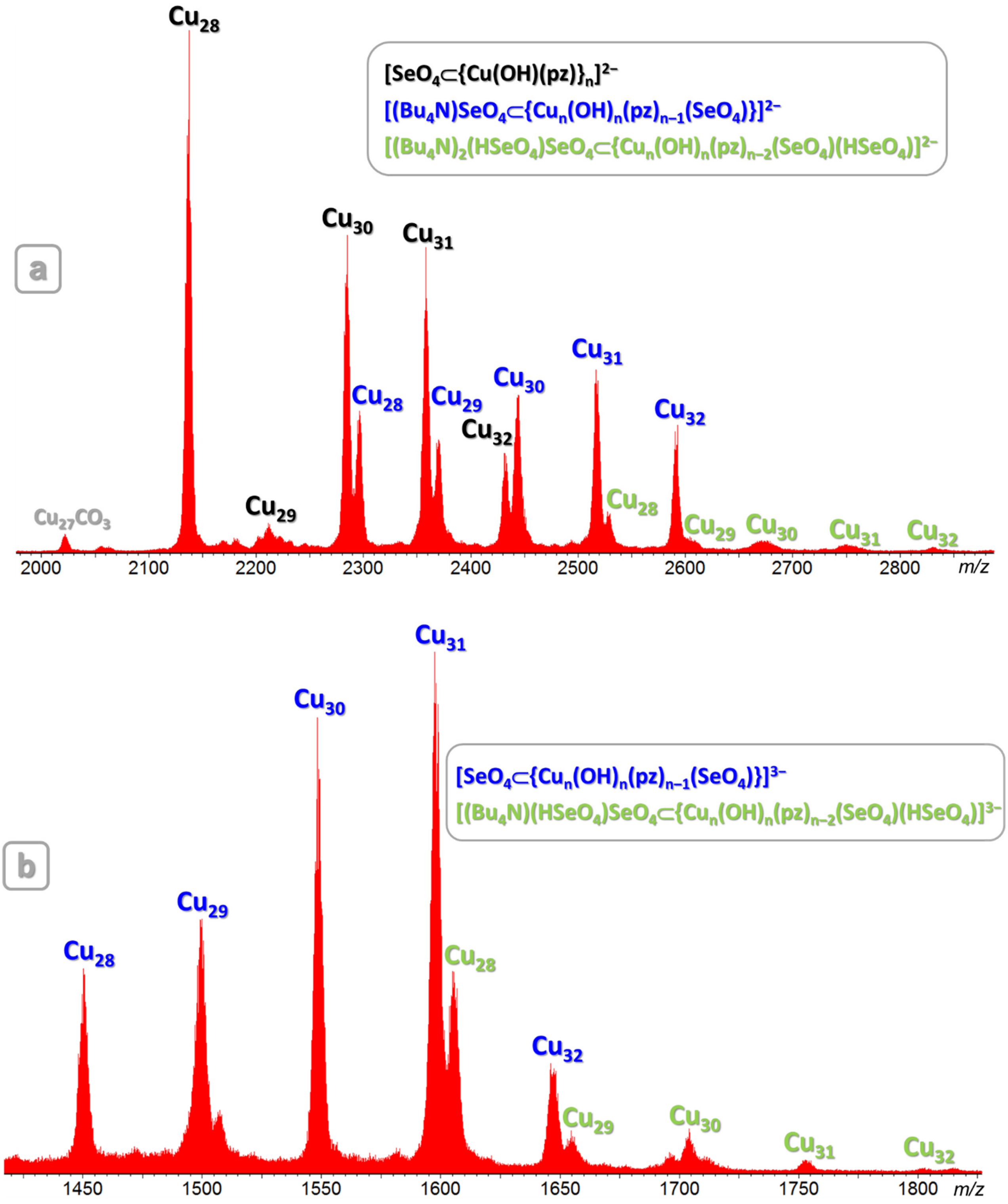

Figure S13. ESI-MS(−) spectra (in CH_3CN) of the **Cu_nSeO_4** nanojar mixture obtained by the depolymerization of [*trans*-Cu^{II}(μ-OH)(μ-pz)]$_\infty$ in the presence of an excess $(Bu_4N)_2SeO_4$ in refluxing toluene displaying (a) 2− and (b) 3− species.

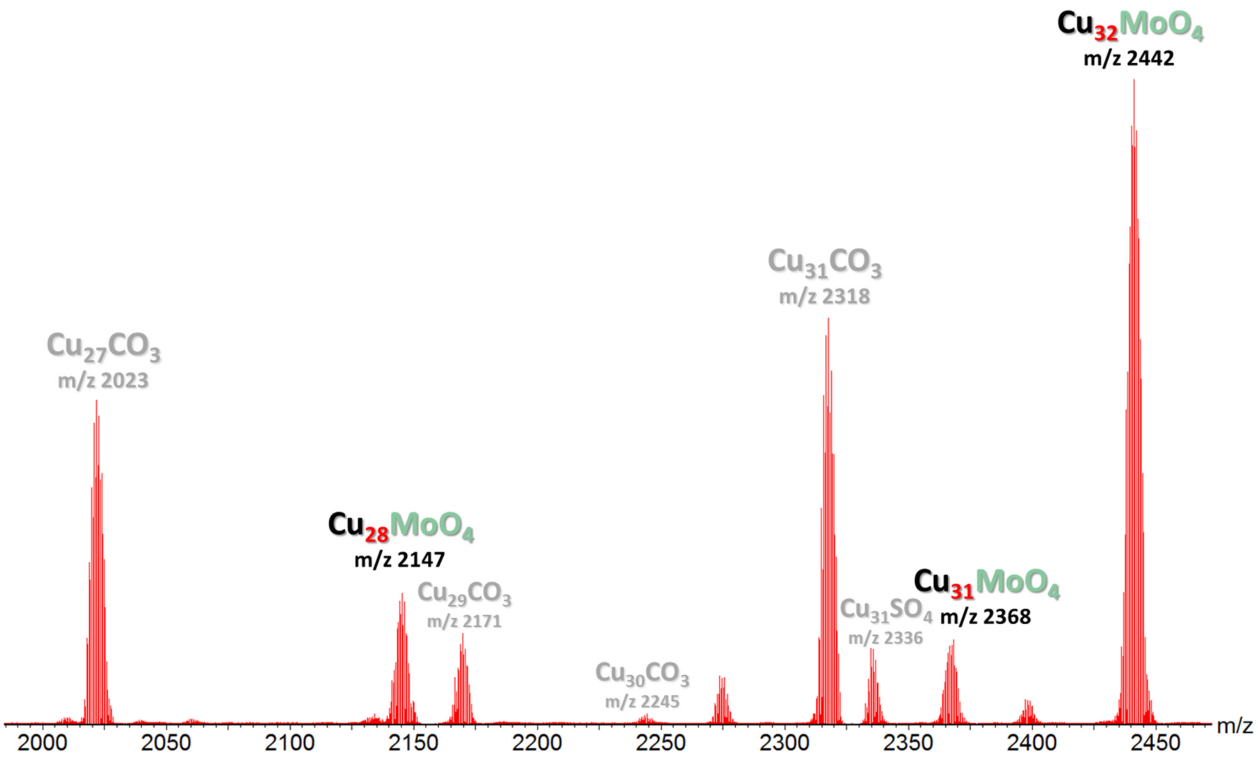

Figure S14. ESI-MS(−) spectrum (in CH_3CN) of the product of the reaction of $Cu_3(MoO_4)_2(OH)_2$ with pyrazole, NaOH and Bu_4NOH (1:3:3.78:0.22 molar ratio) in THF.

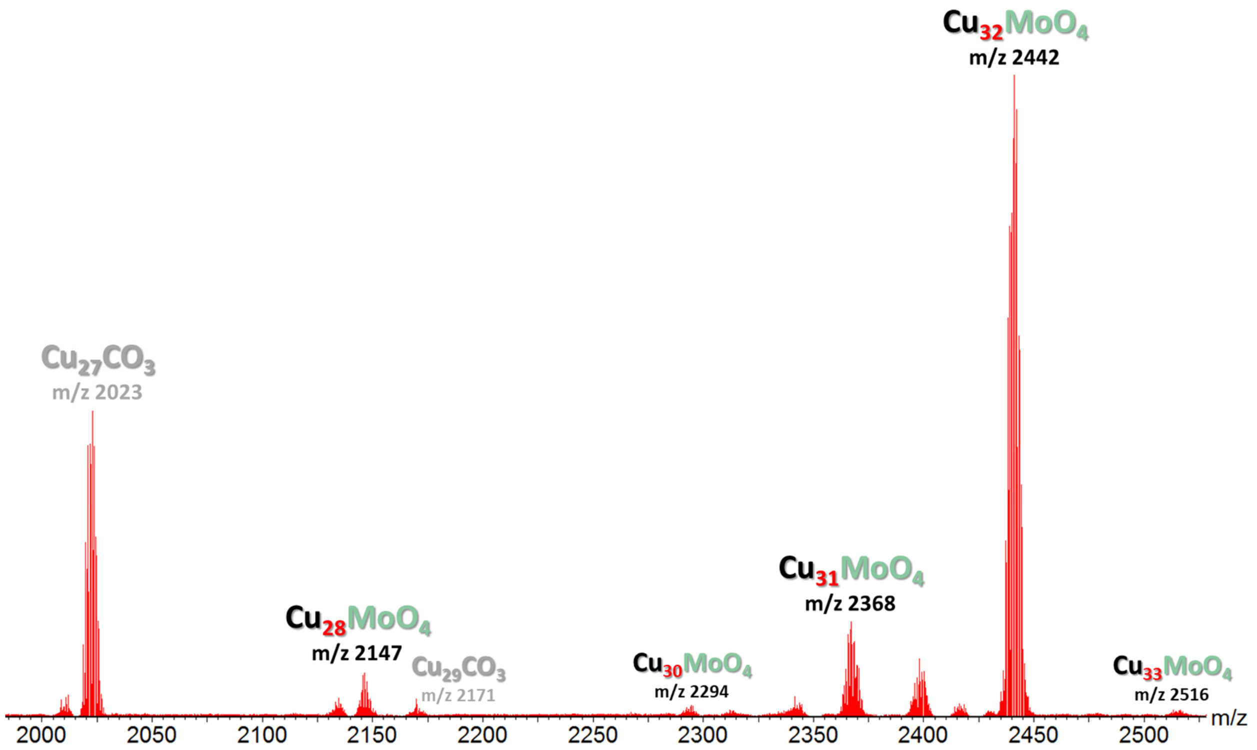

Figure S15. ESI-MS(−) spectrum (in CH_3CN) of the product of the reaction of $Cu(NO_3)_2{\cdot}2.5H_2O$, pyrazole, NaOH, Bu_4NOH and $Na_2MoO_4{\cdot}2H_2O$ (1:1:1.93:0.07:1 molar ratio) in THF.

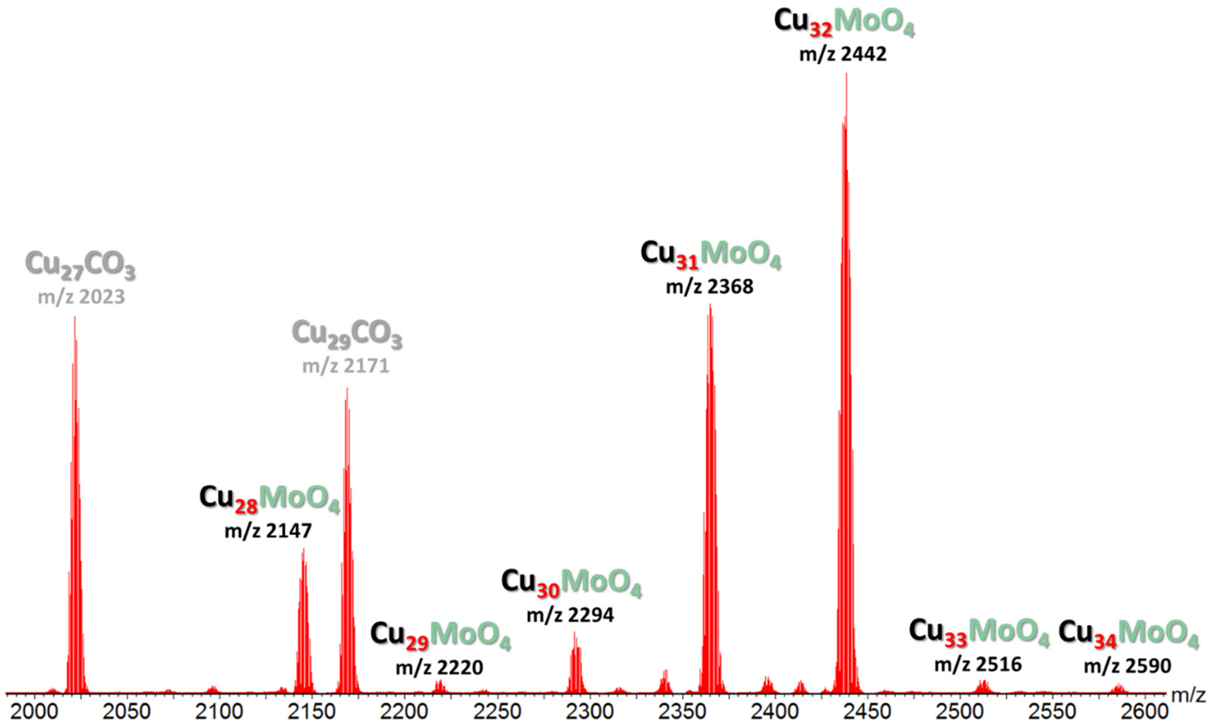

Figure S16. ESI-MS(−) spectrum (in CH_3CN) of the product of the reaction of $Cu(NO_3)_2{\cdot}2.5H_2O$, pyrazole, Et_3N and $(Bu_4N)_2MoO_4$ (1:1:2:1 molar ratio) in THF.

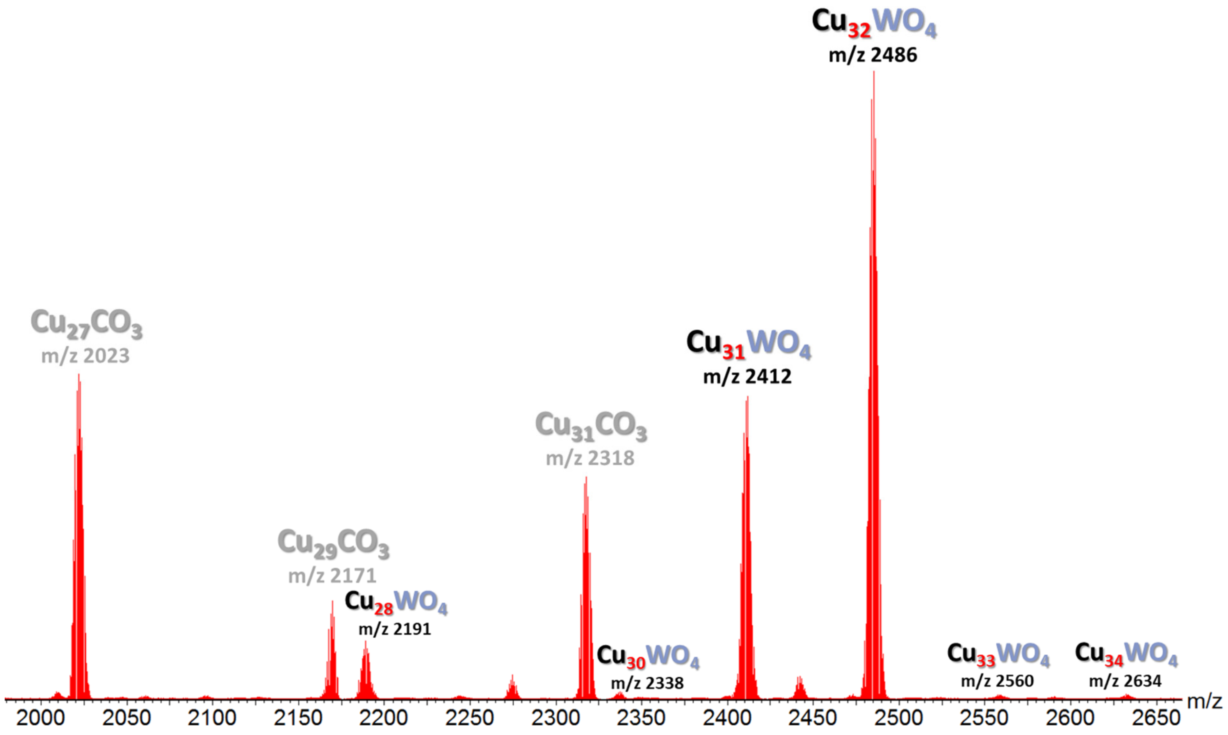

Figure S17. ESI-MS(−) spectrum (in CH_3CN) of the product of the reaction of $CuWO_4{\cdot}2H_2O$ with pyrazole, NaOH and Bu_4NOH (1:1:1.93:0.07 molar ratio) in THF.

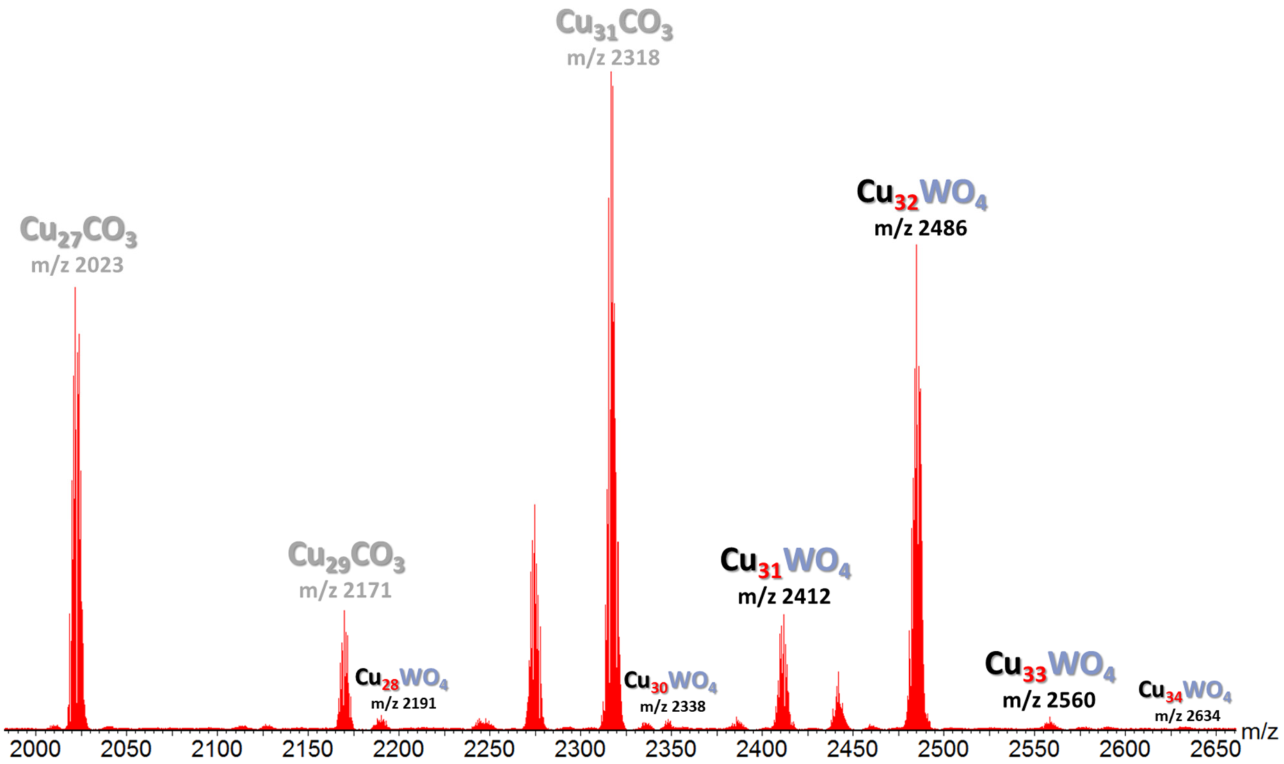

Figure S18. ESI-MS(−) spectrum (in CH_3CN) of the product of the reaction of $Cu(NO_3)_2{\cdot}2.5H_2O$, pyrazole, NaOH, Bu_4NOH and $Na_2WO_4{\cdot}2H_2O$ (1:1:1.93:0.07:1 molar ratio) in THF.

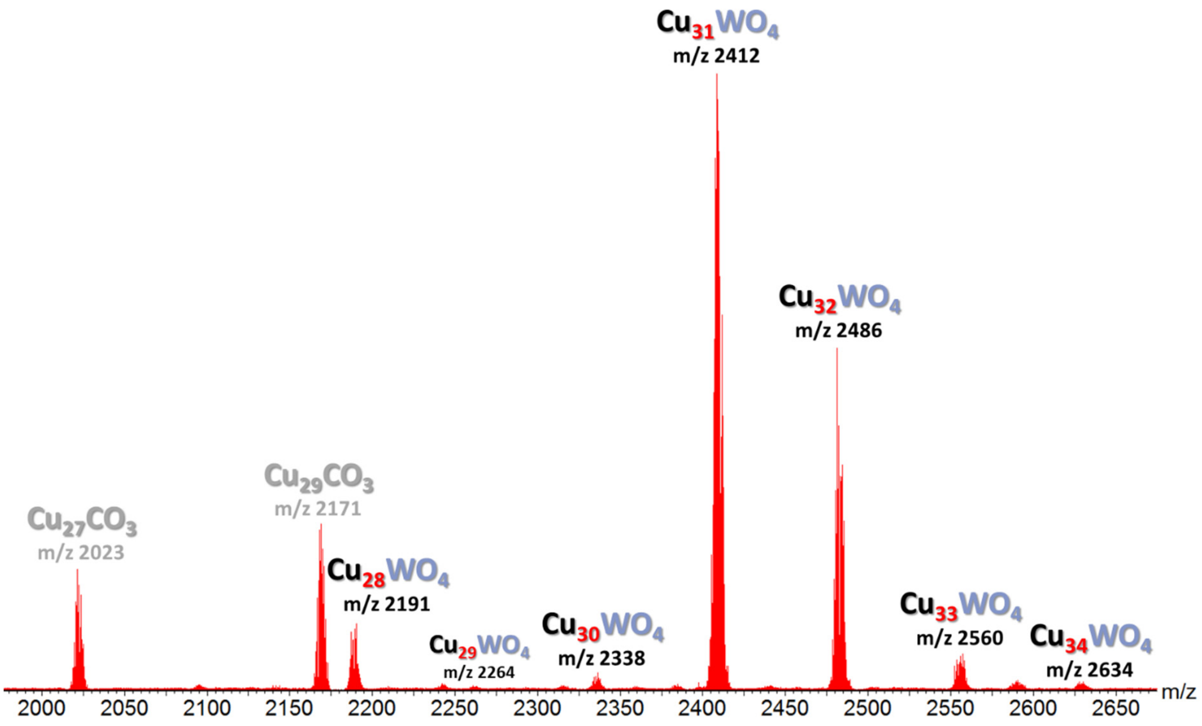

Figure S19. ESI-MS(−) spectrum (in CH_3CN) of the product of the reaction of $Cu(NO_3)_2{\cdot}2.5H_2O$, pyrazole, Et_3N and $(Bu_4N)_2WO_4$ (1:1:2:1 molar ratio) in THF (nanojar region).

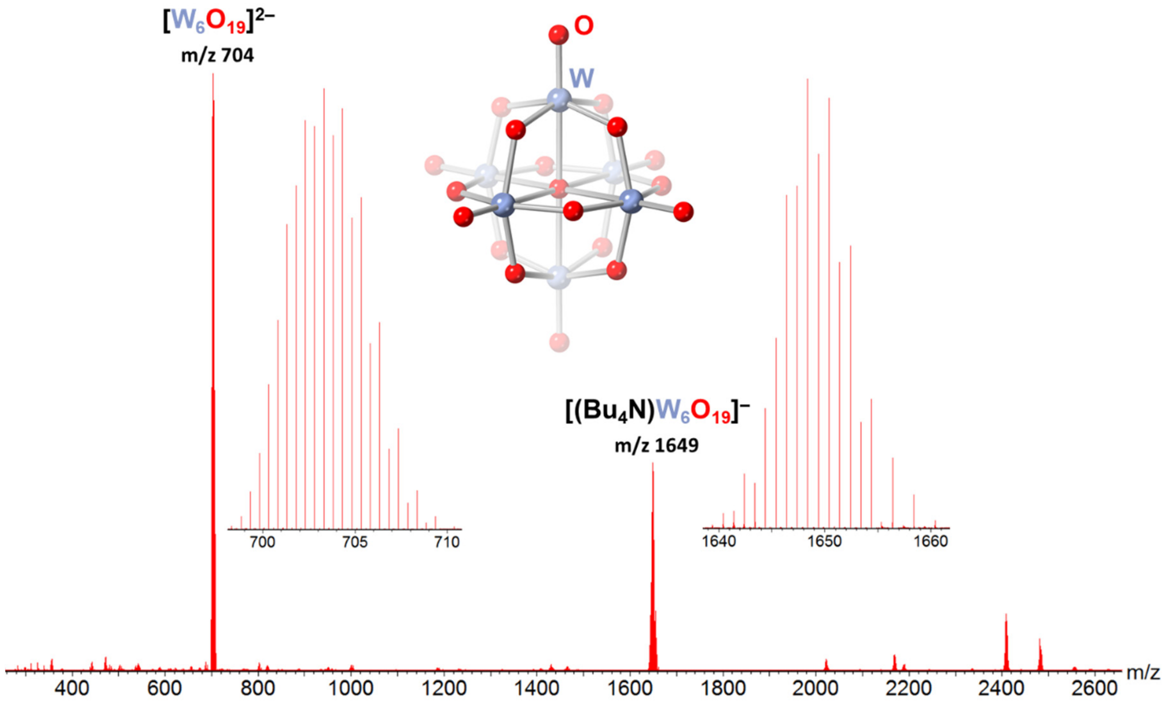

Figure S20. ESI-MS(–) spectrum (in CH_3CN) of the product of the reaction of $Cu(NO_3)_2{\cdot}2.5H_2O$, pyrazole, Et_3N and $(Bu_4N)_2WO_4$ (1:1:2:1 molar ratio) in THF (full spectrum). In addition to nanojars, the presence of the Lindqvist hexatungstate $W_6O_{19}^{2-}$ along with its Bu_4N^+ adduct is also evident.

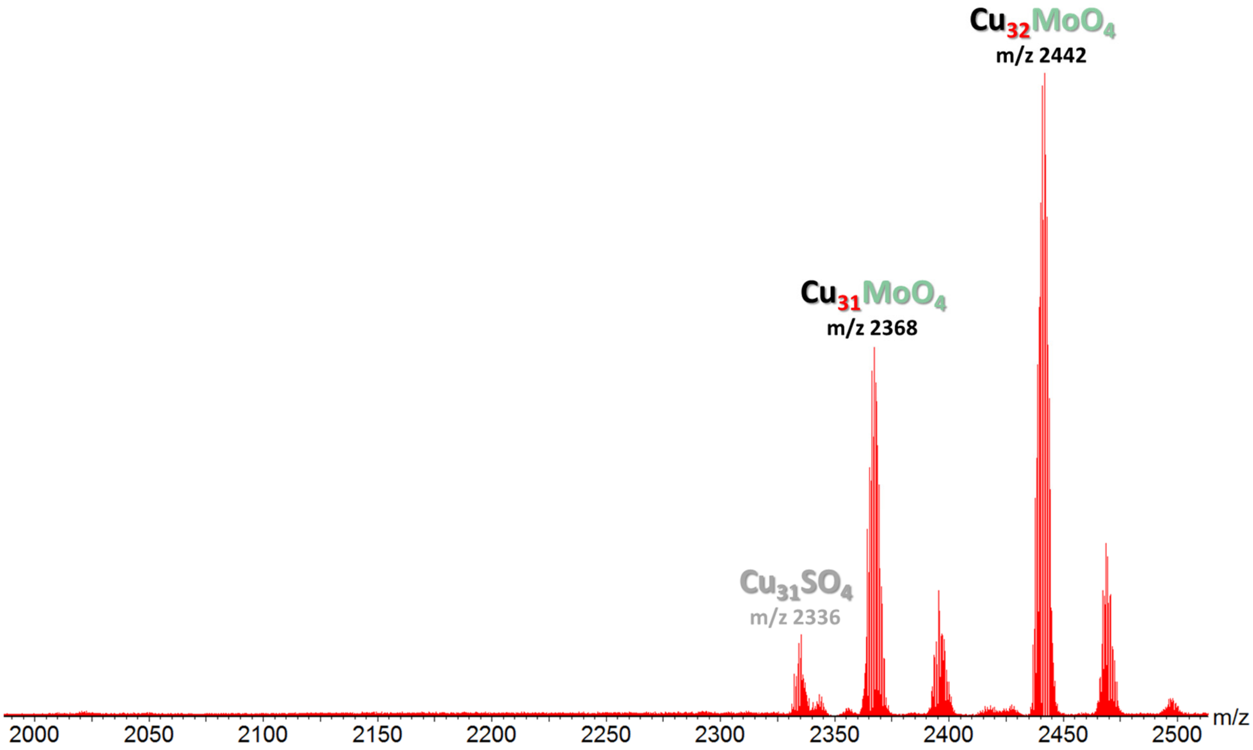

Figure S21. ESI-MS(–) spectrum (in CH_3CN) of the product of the reaction of $Cu(NO_3)_2$, pyrazole, NaOH, Bu_4NOH and $(NH_4)_2MoS_4$ (1:1:1.93:0.07:0.07 molar ratio) in THF.

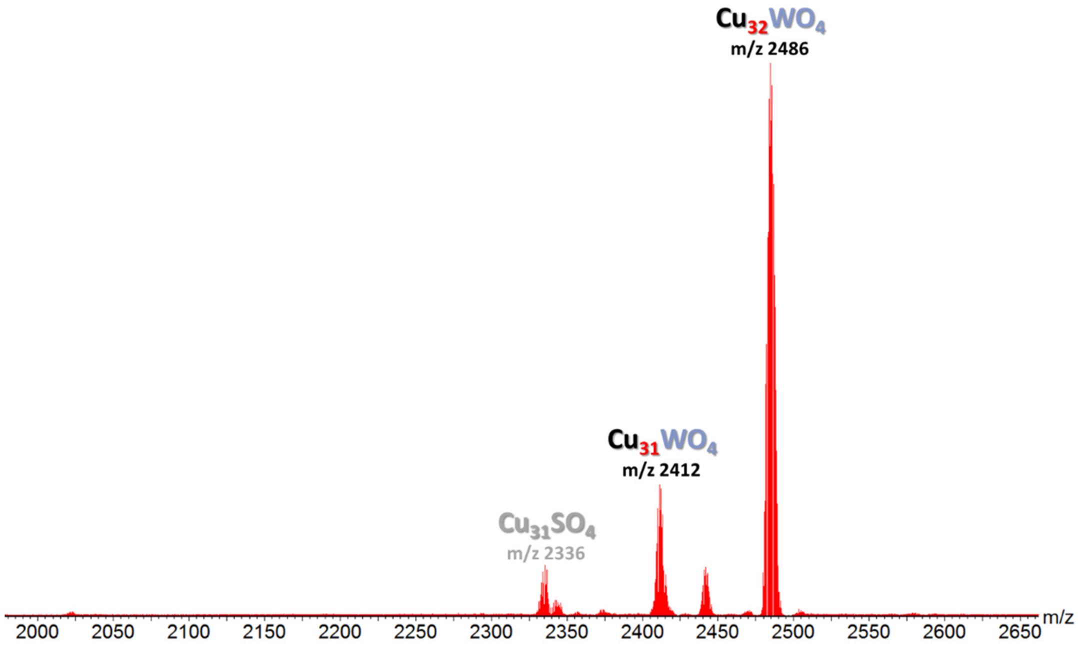

Figure S22. ESI-MS(−) spectrum (in CH_3CN) of the product of the reaction of $Cu(NO_3)_2$, pyrazole, NaOH, Bu_4NOH and $(NH_4)_2WS_4$ (1:1:1.93:0.07:0.07 molar ratio) in THF.

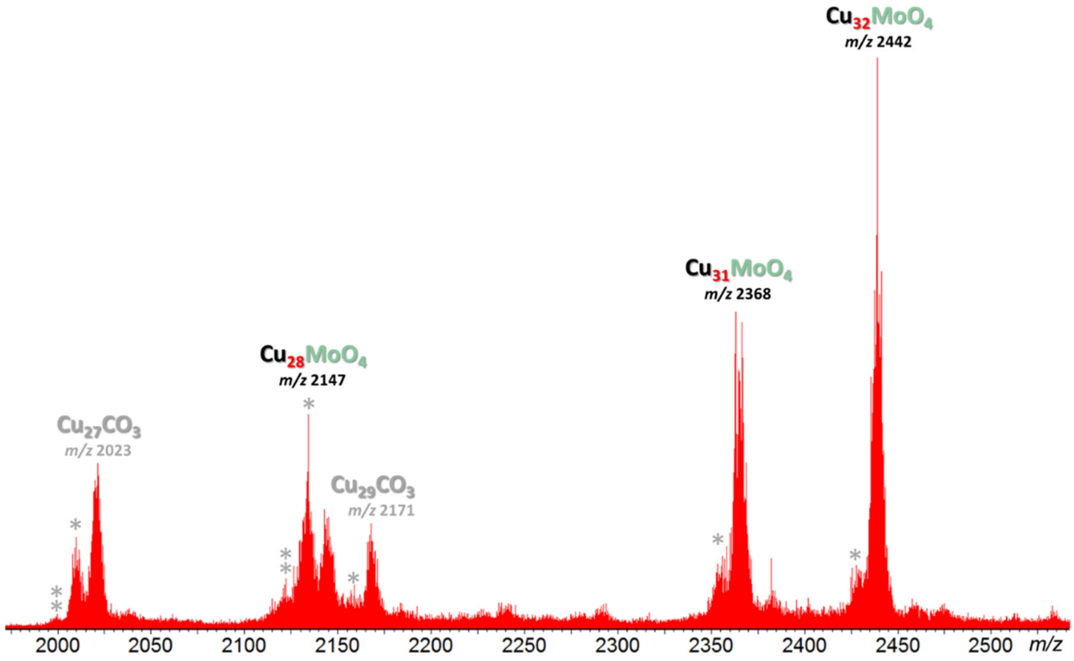

Figure S23. ESI-MS(−) spectrum (in CH_3CN) of the $[MoO_4{\subset}\{Cu(OH)(pz)\}_n]^{2-}$ (**Cu_nMoO_4**; n = 28, 31, 32) nanojar mixture obtained from the reaction of $[Mo^{VI}_8O_{12}(\mu\text{-}O)_9(\mu\text{-}pz)_6(pzH)_6{\cdot}3pzH]$ with $Cu(NO_3)_2$ and Bu_4NOH in THF. Grey asterisks denote singly or doubly formate-substituted species, wherein one or two pz^- moieties are exchanged by $HCOO^-$. Nanojars in general are extremely sensitive to even traces of formic acid in the mass spectrometer, which is commonly used as an additive to improve peak shapes and to promote ionization. Therefore, substituted species such as $[MoO_4^{2-}{\subset}\{Cu_{28}(OH)_{28}(pz)_{28-y}(HCOO)_y\}]$ (y = 1, *m/z* 2136; y = 2, *m/z* 2125) are sometimes observed in the mass spectra of nanojars, at 11 *m/z* units less than the parent species.

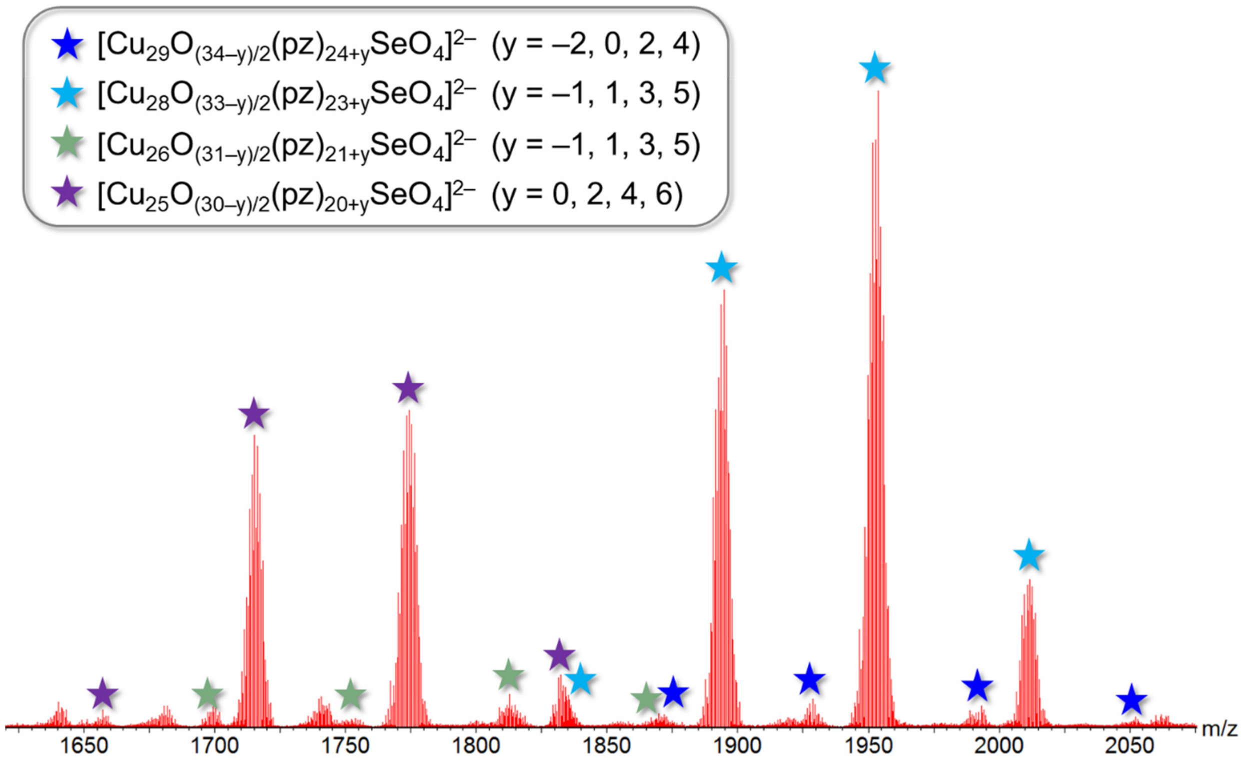

Figure S24. ESI-MS(−) spectrum in CH_3CN of **Cu_nSeO_4** (n = 28−32) at a sampling cone voltage of 100 V, showing shrunken nanojar species.

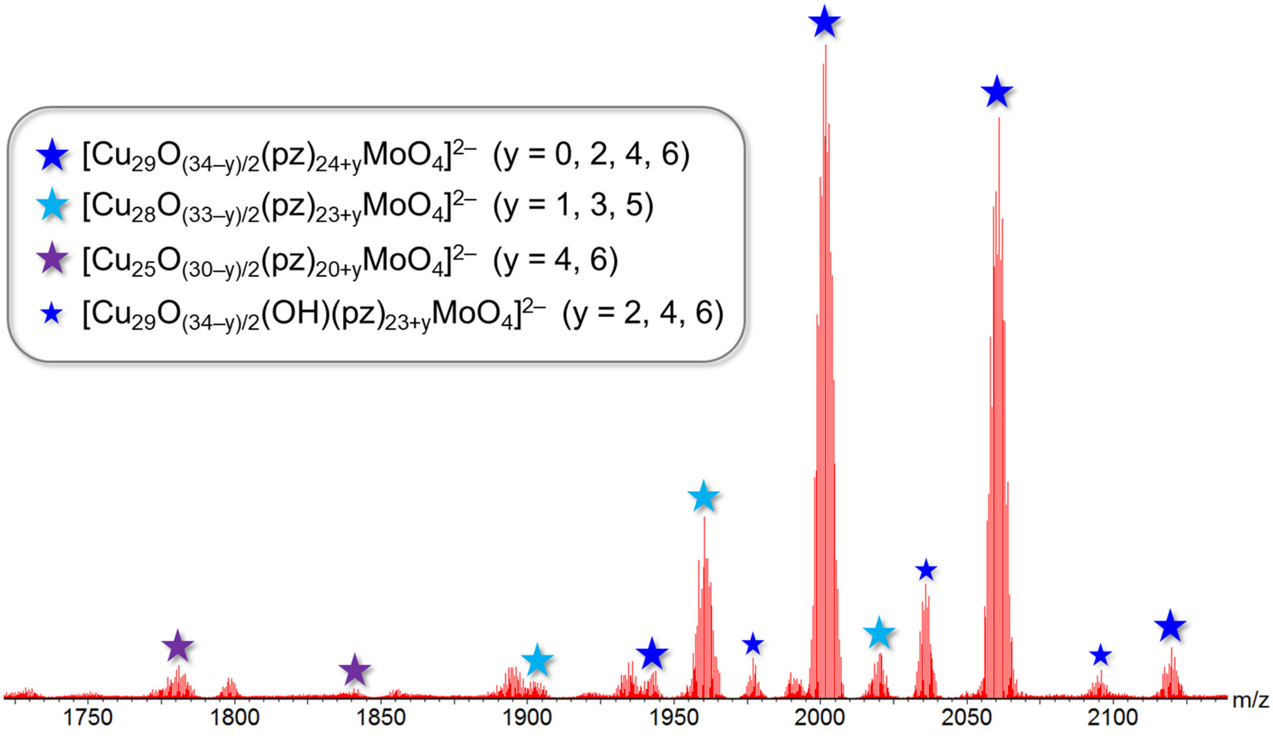

Figure S25. ESI-MS(−) spectrum in CH_3CN of **Cu_nMoO_4** (n = 28, 31, 32) at a sampling cone voltage of 100 V, showing shrunken nanojar species.

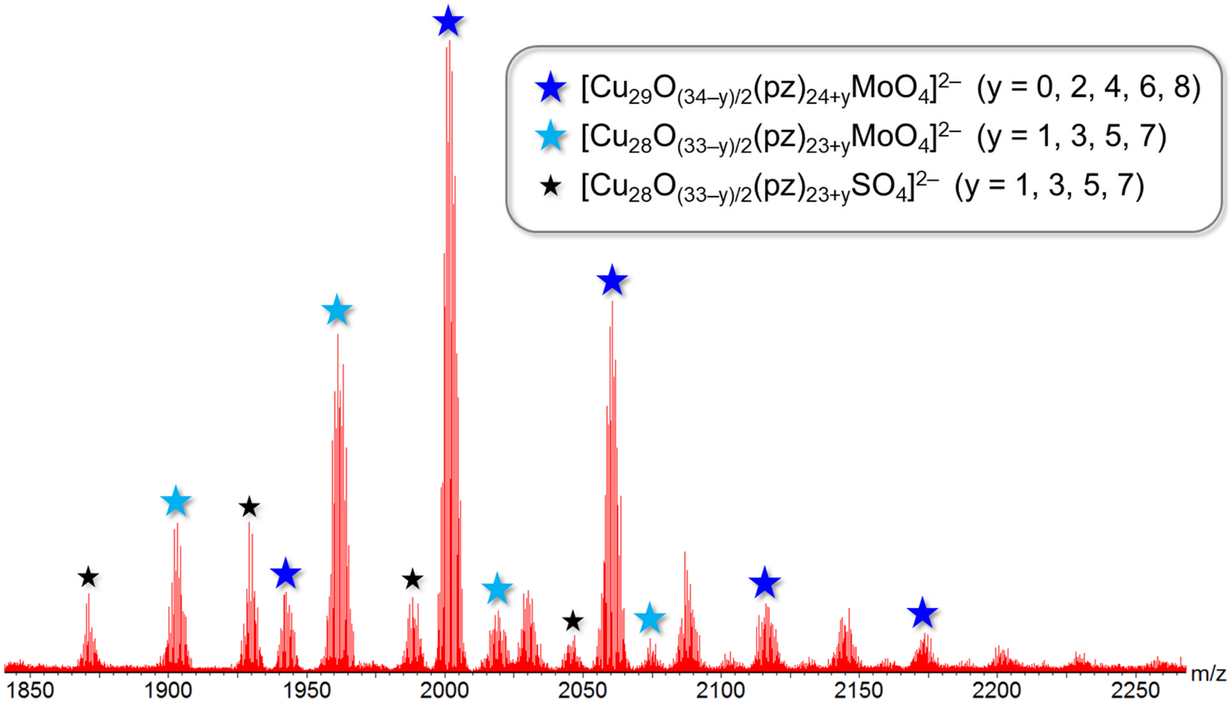

Figure S26. ESI-MS(−) spectrum in CH_3CN of the $\mathbf{Cu_nMoO_4}$ (n = 31, 32) and $\mathbf{Cu_{31}SO_4}$ mixture at a sampling cone voltage of 100 V, showing shrunken nanojar species.

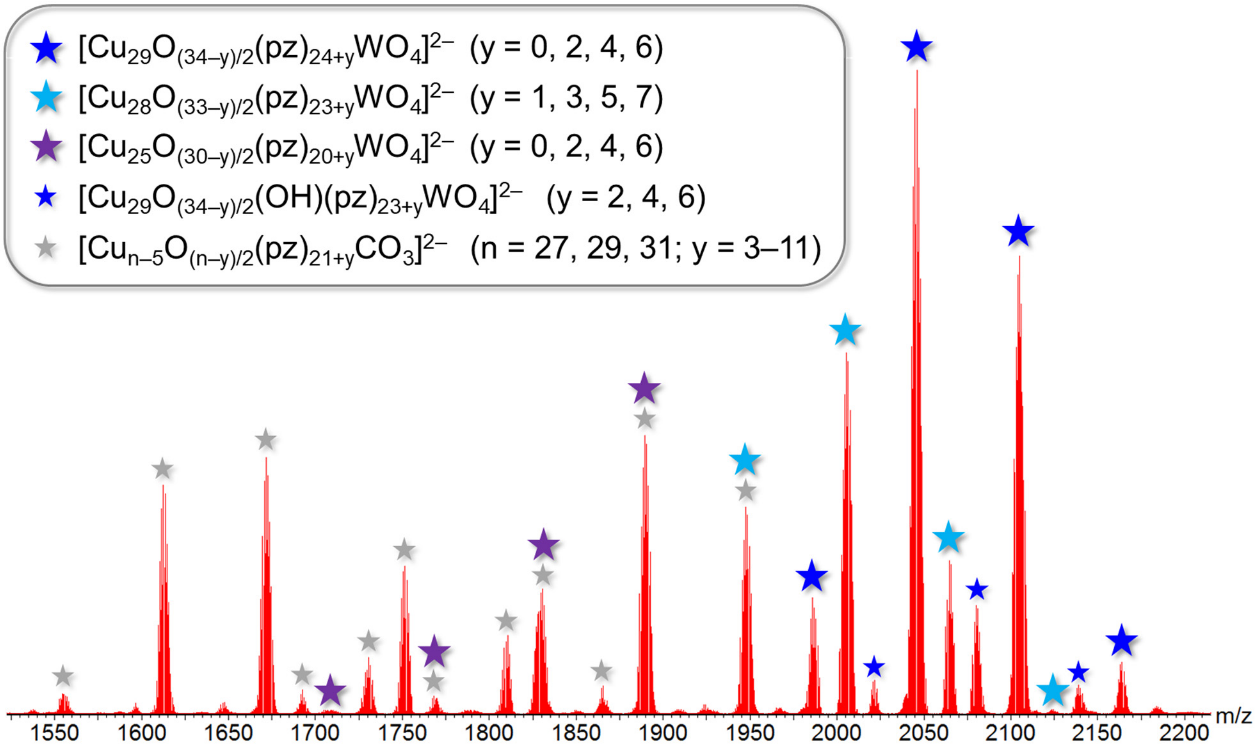

Figure S27. ESI-MS(−) spectrum in CH_3CN of $\mathbf{Cu_nWO_4}$ (n = 28, 31, 32) at a sampling cone voltage of 100 V, showing shrunken nanojar species.

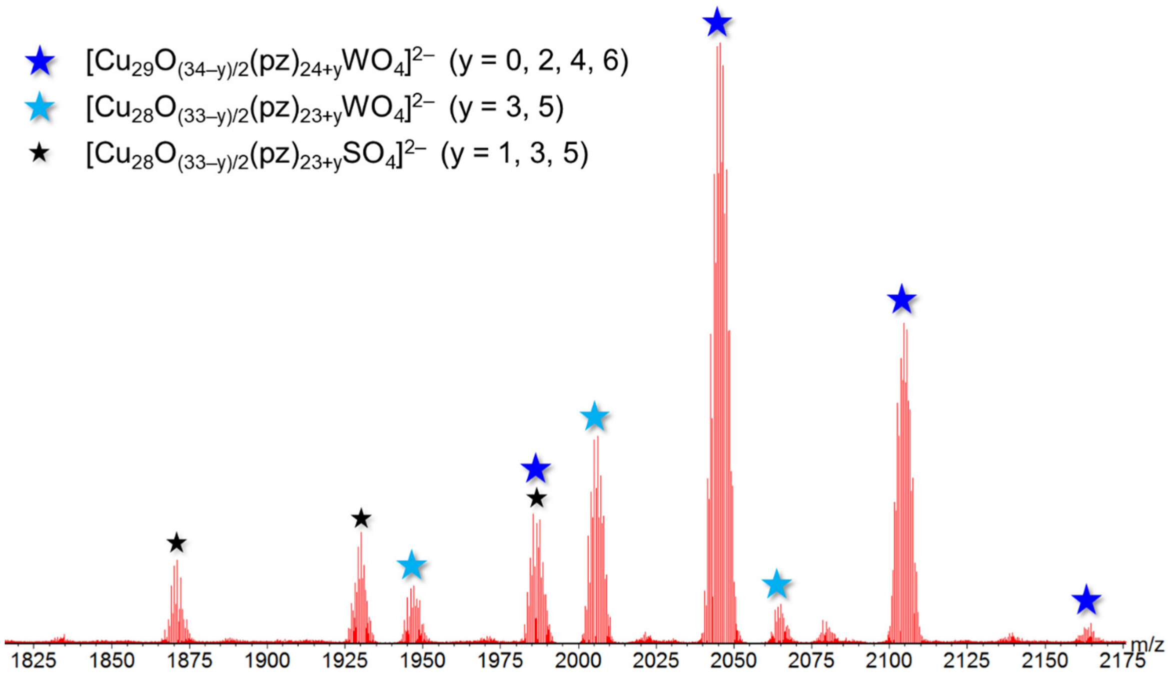

Figure S28. ESI-MS(−) spectrum in CH_3CN of the **Cu_nWO_4** (n = 31, 32) and **$Cu_{31}SO_4$** mixture at a sampling cone voltage of 100 V, showing shrunken nanojar species.

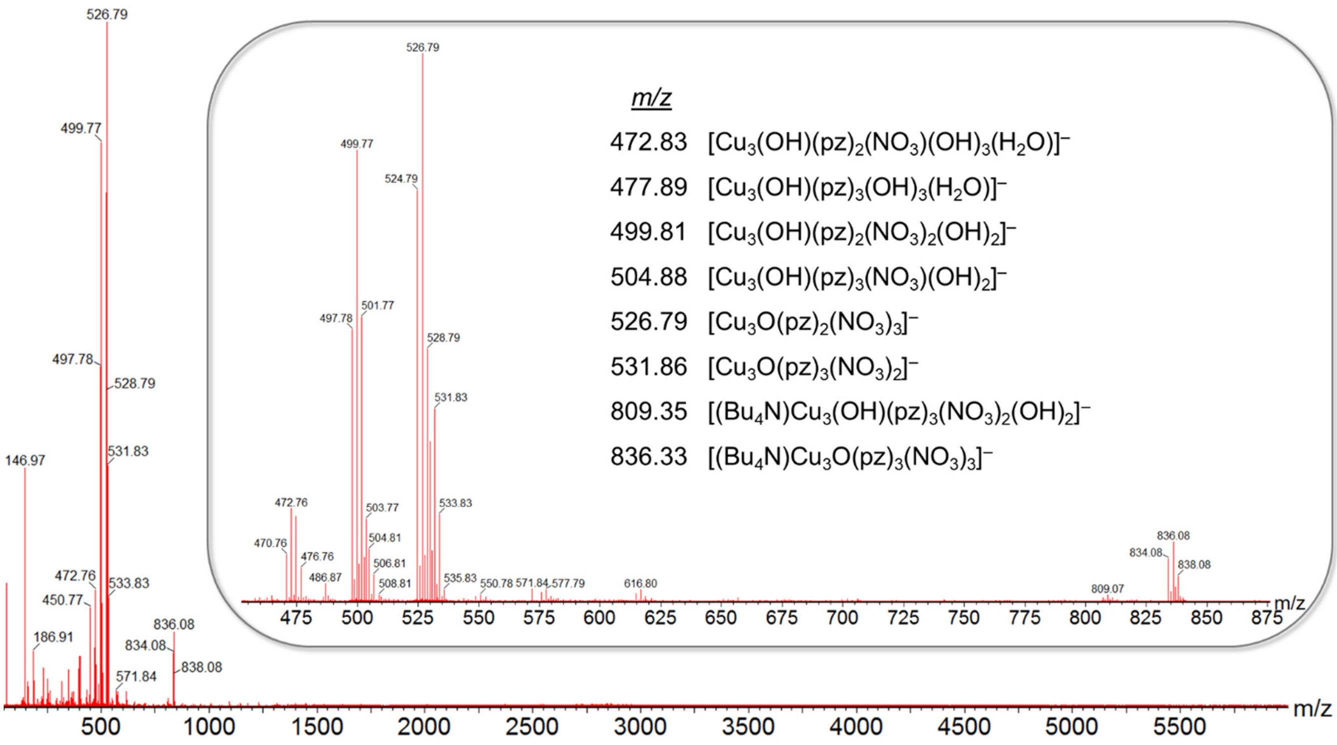

Figure S29. ESI-MS(−) spectrum (in CH_3CN) of the reaction mixture of $Cu(NO_3)_2 \cdot 2.5H_2O$, pyrazole, NaOH, Bu_4NOH and $H_2TeO_4 \cdot 2H_2O$ (1:1:1.93:0.065:0.033 molar ratio) in THF (*m/z* values listed are averages of isotopic distributions).

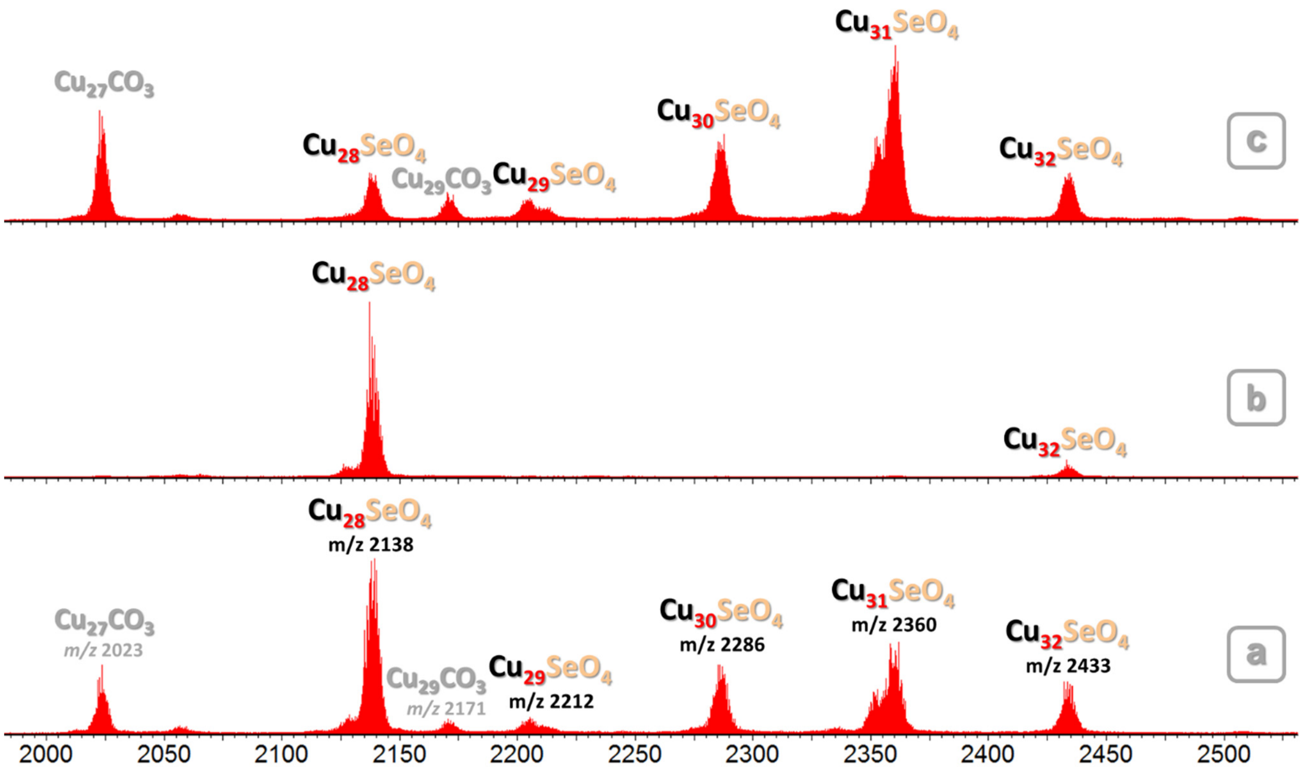

Figure S30. ESI-MS(−) spectra in CH_3CN of (a) the as-synthesized $\mathbf{Cu_nSeO_4}$ (n = 28−33) mixture, (b) the solid fraction crystallized out of a toluene solution, (c) the filtrate containing the toluene-soluble fraction.

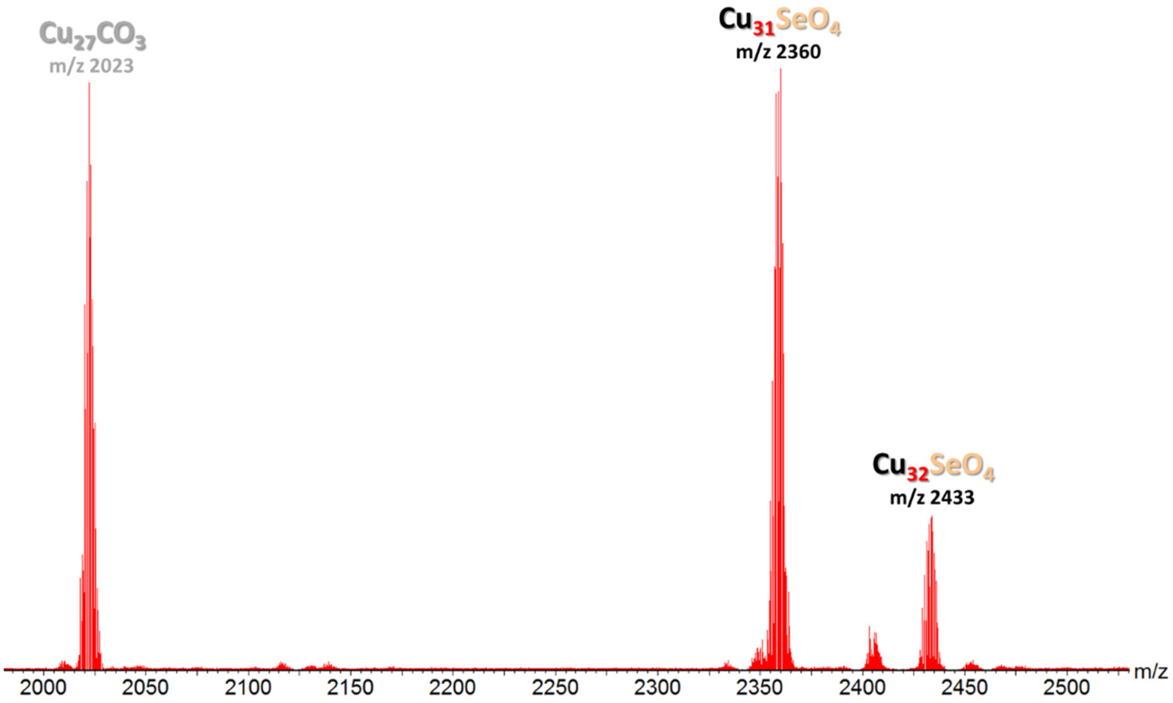

Figure S31. ESI-MS(−) spectrum in CH_3CN of a $\mathbf{Cu_nSeO_4}$ (n = 28−33) and $\mathbf{Cu_nCO_3}$ (n = 27, 29−31) nanojar mixture after standing for 10 weeks in THF solution saturated with NH_3.

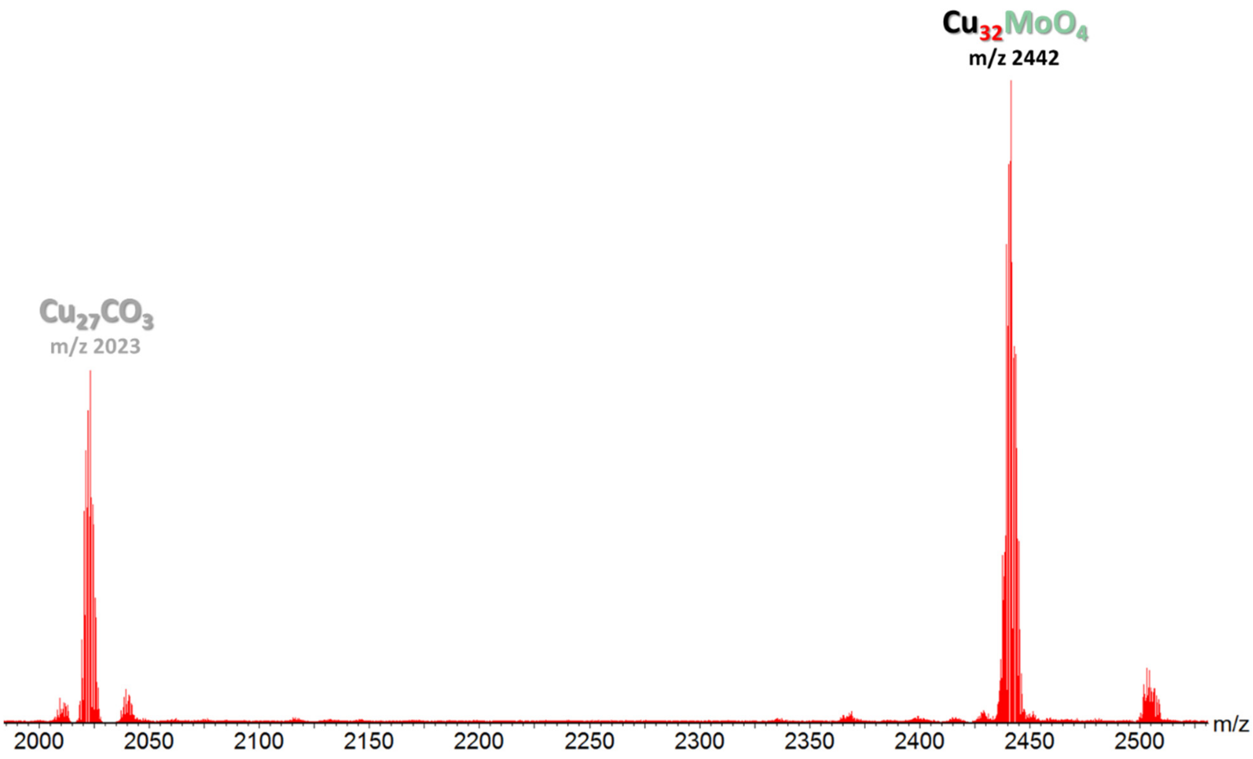

Figure S32. ESI-MS(−) spectrum in CH_3CN of a $\mathbf{Cu_nMoO_4}$ (n = 28−33) and $\mathbf{Cu_nCO_3}$ (n = 27, 29−31) nanojar mixture after standing for 10 weeks in THF solution saturated with NH_3.

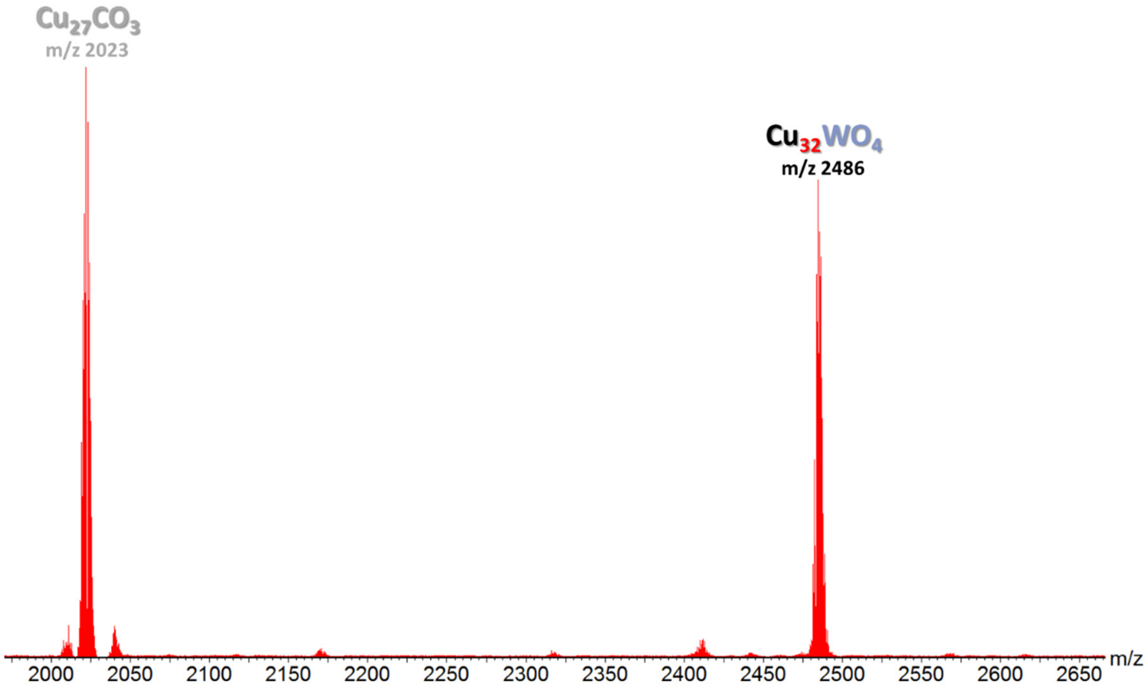

Figure S33. ESI-MS(−) spectrum in CH_3CN of a $\mathbf{Cu_nWO_4}$ (n = 28−33) and $\mathbf{Cu_nCO_3}$ (n = 27, 29−31) nanojar mixture after standing for 10 weeks in THF solution saturated with NH_3.

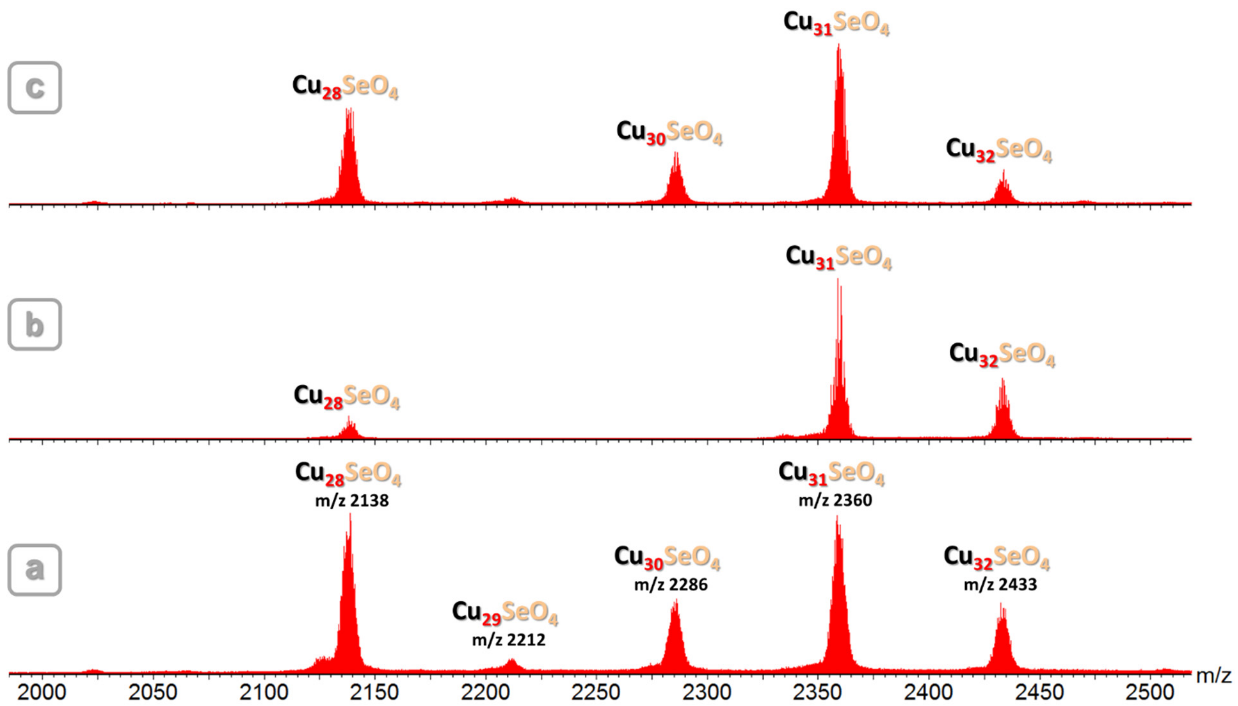

Figure S34. ESI-MS(−) spectra in CH_3CN of (a) the as-synthesized $[SeO_4 \subset \{Cu(OH)(pz)\}_n]^{2-}$ (**Cu_nSeO_4**; n = 28−32) nanojar mixture, (b) the 2-MeTHF solution of **Cu_nSeO_4** after stirring for 1h with an aqueous $Ba(NO_3)_2$ solution, and (c) the 2-MeTHF solution of **Cu_nSeO_4** containing $Ba(DOSS)_2$ after stirring for 1h.

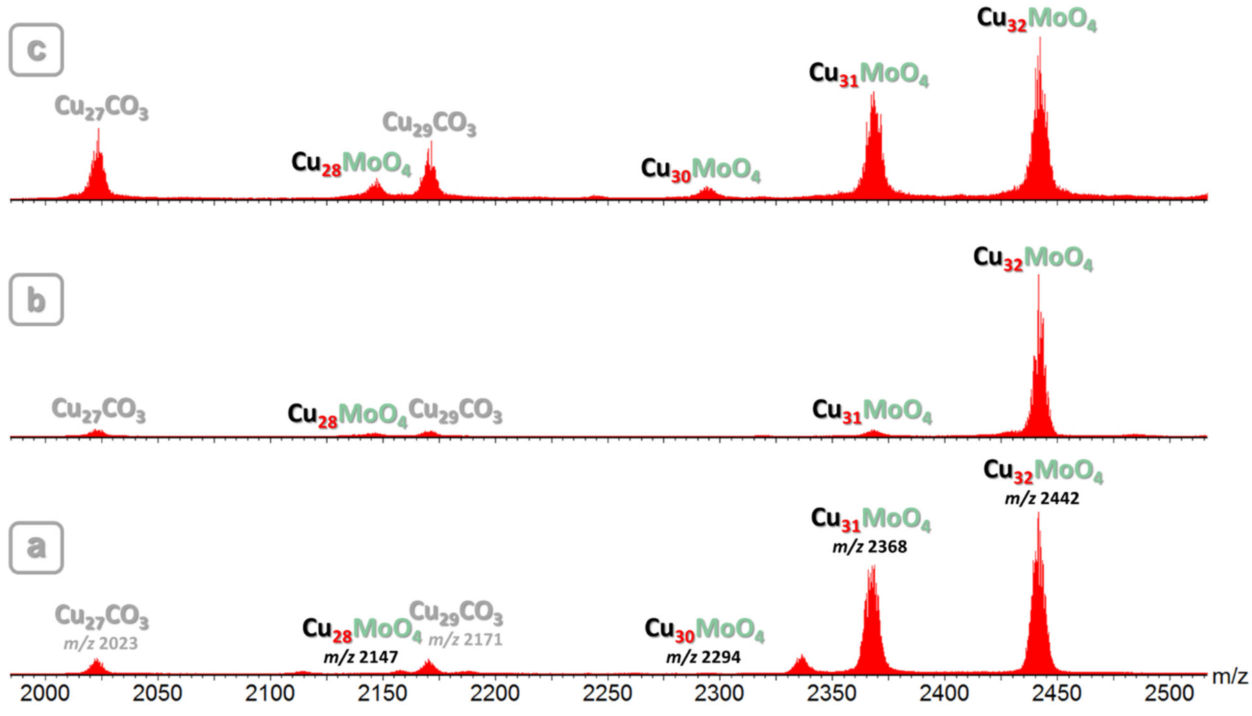

Figure S35. ESI-MS(−) spectra in CH_3CN of (a) the as-synthesized $[MoO_4 \subset \{Cu(OH)(pz)\}_n]^{2-}$ (**Cu_nMoO_4**; n = 28−32) nanojar mixture, (b) the 2-MeTHF solution of **Cu_nMoO_4** after stirring for 1h with an aqueous $Ba(NO_3)_2$ solution, and (c) the 2-MeTHF solution of **Cu_nMoO_4** containing $Ba(DOSS)_2$ after stirring for 1h.

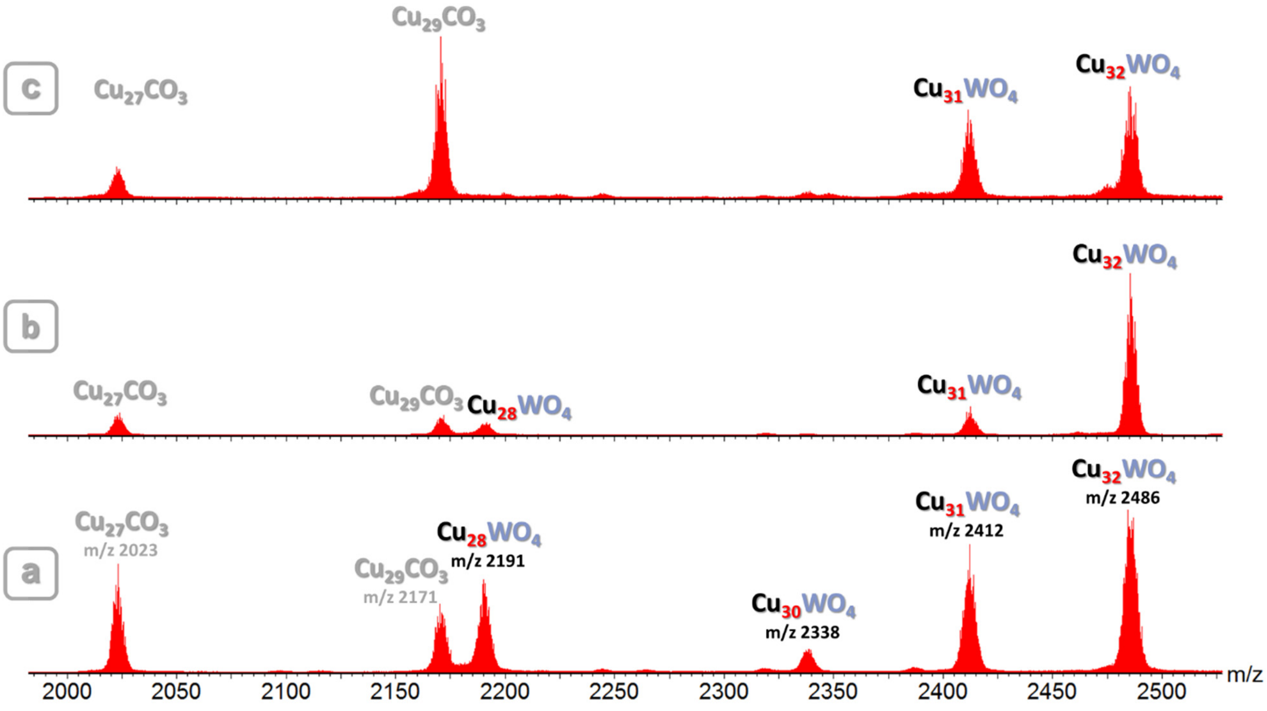

Figure S36. ESI-MS(−) spectra in CH_3CN of (a) the as-synthesized $[WO_4{\subset}\{Cu(OH)(pz)\}_n]^{2-}$ (**Cu_nWO_4**; n = 28−32) nanojar mixture, (b) the 2-MeTHF solution of **Cu_nWO_4** after stirring for 1h with an aqueous $Ba(NO_3)_2$ solution, and (c) the 2-MeTHF solution of **Cu_nWO_4** containing $Ba(DOSS)_2$ after stirring for 1h.

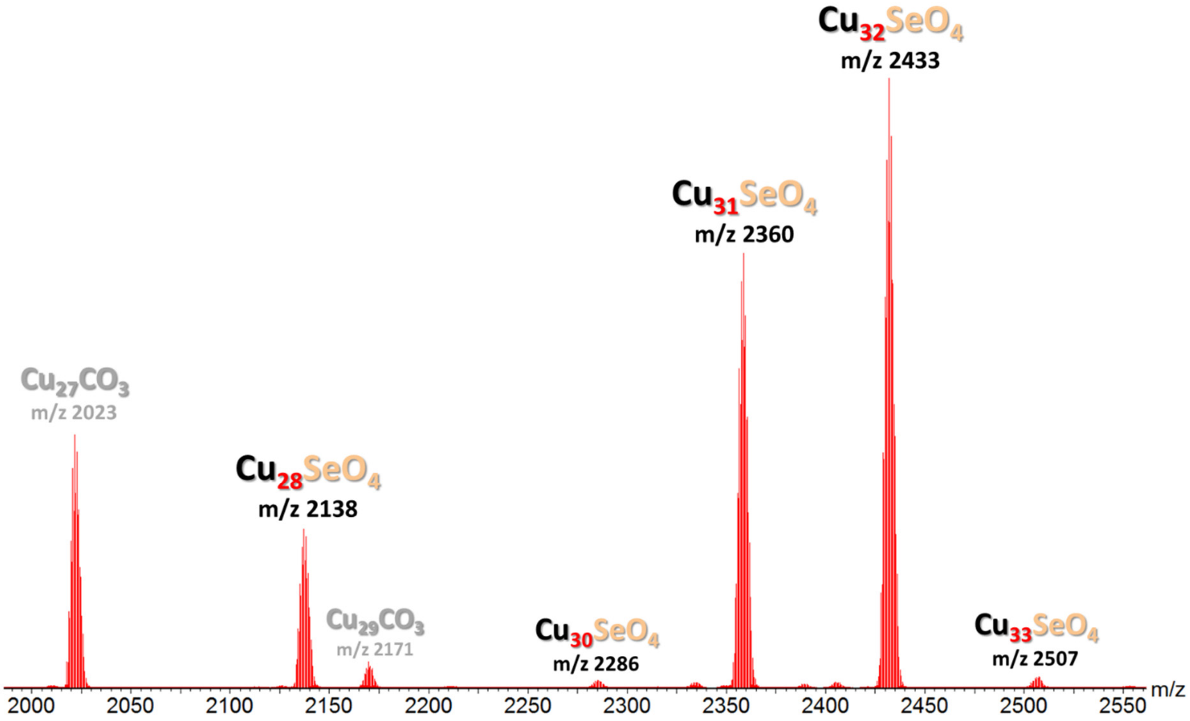

Figure S37. ESI-MS(−) spectrum in CH_3CN of the $[SeO_4{\subset}\{Cu(OH)(pz)\}_n]^{2-}$ (**Cu_nSeO_4**; n = 28−33) obtained by the extraction of the SeO_4^{2-} ion from water into THF.

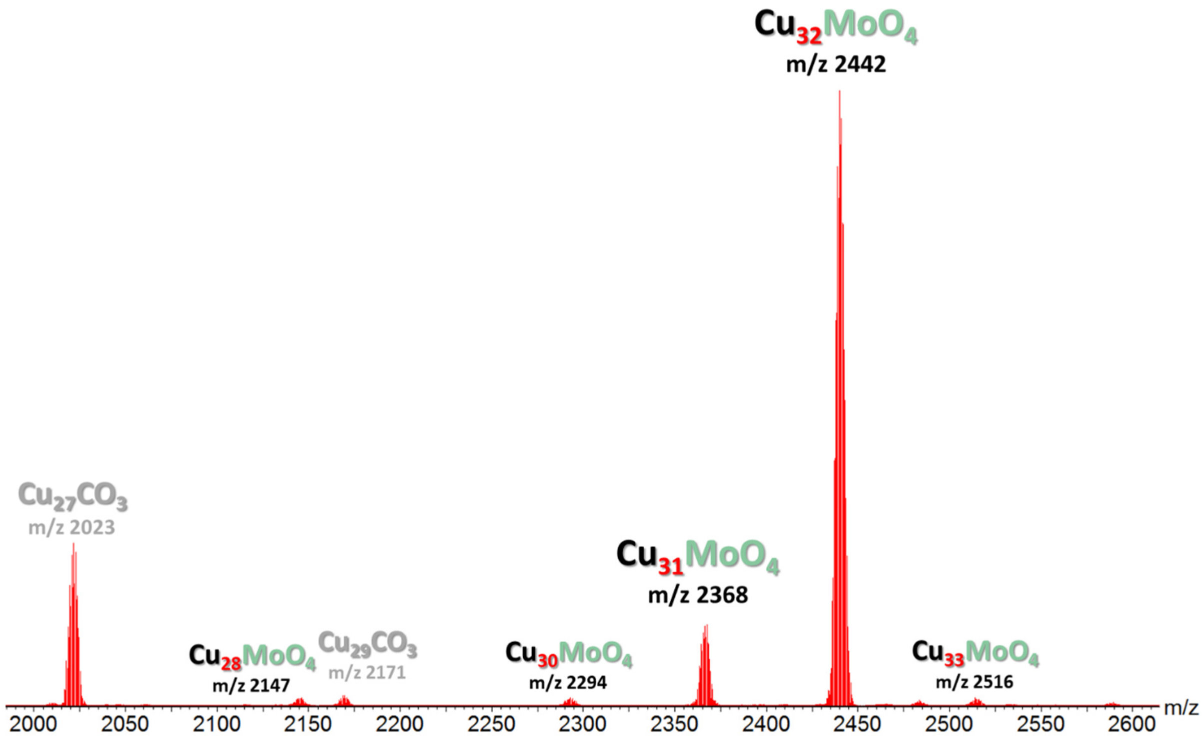

Figure S38. ESI-MS(−) spectrum in CH_3CN of the $[MoO_4\subset\{Cu(OH)(pz)\}_n]^{2-}$ (**Cu_nMoO_4**; n = 28−33) obtained by the extraction of the MoO_4^{2-} ion from water into THF.

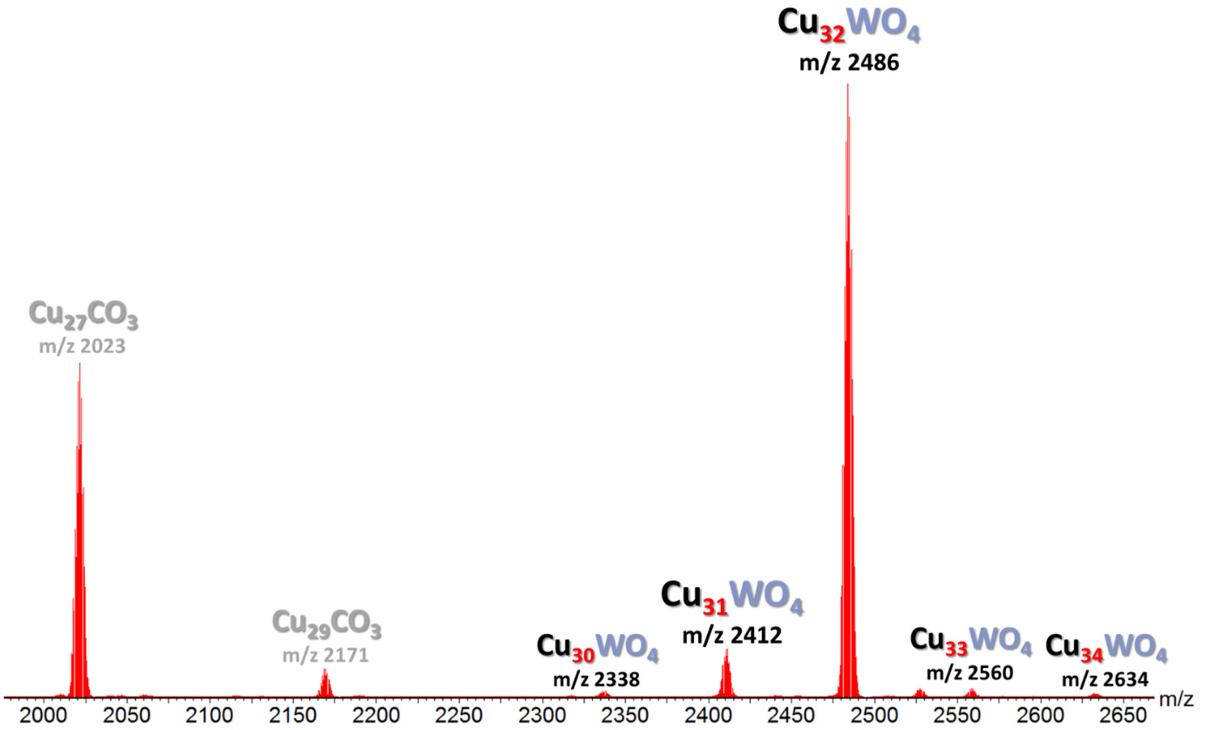

Figure S39. ESI-MS(−) spectrum in CH_3CN of the $[WO_4\subset\{Cu(OH)(pz)\}_n]^{2-}$ (**Cu_nWO_4**; n = 30−34) obtained by the extraction of the WO_4^{2-} ion from water into THF.

3. X-RAY CRYSTALLOGRAPHIC DATA

1: The crystal under investigation was found to be non-merohedrally twinned. The orientation matrices for the two components were identified using the program Cell_Now, with the two components being related by a 180° rotation around the reciprocal axis (0 −1 1). The two components were integrated using Saint and corrected for absorption using Twinabs, resulting in the following statistics:

47554 data (19144 unique) involve domain 1 only, mean I/σ 18.6
47269 data (19127 unique) involve domain 2 only, mean I/σ 18.4
72490 data (30560 unique) involve 2 domains, mean I/σ 30.3
35 data (35 unique) involve 3 domains, mean I/σ 28.9

The exact twin matrix identified by the integration program was found to be:

−0.99999	0.00007	−0.00014
0.40764	0.23170	−0.76800
−0.40764	−1.23208	−0.23172

The structure was solved using direct methods with only the non-overlapping reflections of component 1. The structure was refined using the HKLF 5 routine with all reflections of component 1 (including the overlapping ones), resulting in a BASF value of 0.4884(5). The R_{int} value given is for all reflections and is based on agreement between observed single and composite intensities and those calculated from refined unique intensities and twin fractions.[1]

The SeO_4^{2-} ion was refined as three-fold disordered. The disordered moieties were restrained to have similar geometries. U_{ij} components of ADPs for disordered atoms closer to each other than 2.0 Å were restrained to be similar. Subject to these conditions the occupancy ratio refined to 0.492(4)/0.351(3)/0.156(3).

Two pyrazole ligands (of N21 and N37) were refined as disordered. Major and minor disordered moieties were each restrained to have similar geometries. For the ligand of N21, equivalent Cu–N distances were restrained to be similar. For the ligand of N37, the pyrazole moieties were restrained to be coplanar with the Cu ions. U_{ij} components of ADPs for disordered atoms closer to each other than 2.0 Å were restrained to be similar (for the ligand of N37 the Cu ions were also

included in this restraint). Subject to these conditions, the occupancy ratio refined to 0.206(19)/0.794(19) and to 0.823(12)/0.177(12).

Hydroxyl H atom positions were refined and O–H distances were restrained to a target value of 0.84(2) Å. One of the Bu_4N^+ cations (N63) exhibits minor disorder of one butyl chain. The other exhibits whole-cation disorder. In the former, atoms C100 and C101 were refined as disordered and major and minor moieties were restrained to have similar geometries. The moieties of the second cation (N64) were restrained to have similar geometries as the major moiety of the first cation. U_{ij} components of ADPs for disordered atoms closer to each other than 2.0 Å were restrained to be similar. Subject to these conditions, the occupancy ratio refined to 0.270(18)/0.730(18) for C101 and C102, and to 0.459(9)/0.541(9) for the second cation.

Chlorobenzene solvate molecules are extensively disordered. All chlorobenzene molecules were each restrained to have similar geometries. For the chlorobenzene molecules of the following chlorine atoms the Cl to *ortho*-C atoms were restrained to be similar: Cl2, Cl32, Cl33, Cl41, Cl42, Cl52. The chlorobenzene molecules of the following chlorine atoms were restrained to be close to planar: Cl1, Cl2, Cl4, Cl31, Cl32, Cl33, Cl34, Cl41, Cl42, Cl43, Cl44, Cl52. U_{ij} components of ADPs for disordered atoms closer to each other than 2.0 Å were restrained to be similar, and some solvate atoms were restrained to be close to isotropic. A global anti-bumping restraint was applied. Subject to these conditions, the occupancy rates refined to the values given in the CIF file.

A pentane molecule was refined as partially occupied. A rigid bond restraint was applied for its thermal parameters. Subject to these conditions the occupancy rate refined to 0.825(14).

In addition to the twinning and disorder, the structure also exhibits large volume sections consisting of highly disordered solvate or other small molecules. No satisfactory model for the solvate molecules could be developed, and the contribution of the solvate molecules was instead taken into account by reverse Fourier transform methods. The data were first de-twinned (using the LIST 8 function of Shelxl 2018) and then the CIF and FCF files were subjected to the SQUEEZE routine as implemented in the program Platon.[2] The resultant files were used for further refinement. Both the HKLF 5 type HKL file and the de-twinned FAB file are appended to the CIF file. A volume of 451 $Å^3$ per unit cell containing 98 electrons was corrected for.

2: The structure is mostly isomorphous to its BeF_4^{2-} counterpart and was solved by isomorphous replacement. Disorder of the SeO_4^{2-} anion, several pyrazoles, and of the solvate model was adjusted as necessary. Several solvate molecules relatively well-resolved in the BeF_4^{2-} analogue (1,2-dichlorobenzene, pentane) were not resolved in the SeO_4^{2-} structure, or are replaced by highly disordered heptane too ill-defined to refine. The atom-naming scheme for the remaining 1,2-dichlorobenzene was retained for ease of comparison.

Hydroxyl H atom positions were refined and O–H distances were restrained to 0.84(2) Å. Some hydroxyl H atom positions were further restrained based on hydrogen bonding considerations.

The SeO_4^{2-} anion was refined as two-fold disordered by rotation. The disordered moieties were restrained to have similar geometries. U_{ij} components of ADPs for disordered atoms closer to each other than 2.0 Å were restrained to be similar. Subject to these conditions, the occupancy ratio refined to 0.659(12)/0.341(12).

Several pyrazole ligands were refined as disordered. For all, the two disordered moieties were restrained to have similar geometries. Each moiety was restrained to be coplanar with the copper ions it is bonded to. For the pyrazole of N1 and N2, and N5 and N6, the Cu–N distances were also restrained to be similar to each other. U_{ij} components of ADPs for disordered atoms closer to each other than 2.0 Å were restrained to be similar. Subject to these conditions, the occupancy ratios refined to 0.59(4)/0.41(4) for the pyrazole of N1/N2, to 0.52(3)/0.48(3) for the pyrazole of N1/N2, to 0.571(10)/0.429(10) for the pyrazole of N5/N6, to 0.64(3)/0.36(3) for the pyrazole of N11/N12, to 0.614(16)/0.386(16) for the pyrazole of N49/N50, and to 0.645(16)/0.355(16) for the pyrazole of N51/N52.

One of the two Bu_4N^+ cations exhibits disorder of three of the butyl chains. The N-atom was included in the disorder. The other Bu_4N^+ cation exhibits whole-cation disorder. The six moieties of the disordered butyl chains were restrained to have a similar geometry as another non-disordered butyl chain. The two moieties of the disordered cation were restrained to have a similar geometry as the other less-disordered cation. U_{ij} components of ADPs for disordered atoms closer to each other than 2.0 Å were restrained to be similar. Subject to these conditions the occupancy ratio refined to 0.571(10)/0.429(10) for the three disordered butyl chains, and to 0.572(9)/0.428(9) for the whole-cation disorder.

Two 1,2-chlorobenzene solvate molecules were refined as disordered. Another 1,2-chlorobenzene molecule, nearby the disordered cation, was refined as partially occupied. All 1,2-chlorobenzene molecules were restrained to have similar geometries, and all benzene rings were constrained to resemble ideal hexagons with C–C bond distances of 1.39 Å. U_{ij} components of ADPs for disordered atoms closer to each other than 2.0 Å were restrained to be similar. No attempts were made to ensure full occupancy for all solvate sites. Minor ill-defined residual electron density was ignored. Subject to these conditions the occupancy ratios refined to 0.543(10)/0.457(10) and 0.711(8)/0.289(8) for the disordered 1,2-chlorobenzene molecules (Cl1/Cl2, Cl9/Cl10). The occupancy rate for the molecule disordered with pentane refined to 0.572(10) (Cl3/Cl3).

The structure contains additional 3328 $Å^3$ of solvent-accessible voids. No substantial electron density peaks were found in the solvent accessible voids (less than 2.32 electron/$Å^3$) and the residual electron density peaks are not arranged in an interpretable pattern (it vaguely resembled highly disordered heptane). The structure factors were instead augmented via reverse Fourier transform methods using the SQUEEZE routine as implemented in the program Platon.[2] The resultant FAB file containing the structure factor contribution from the electron content of the void space was used together with the original HKL file for further refinement. The FAB file with details of the SQUEEZE results is appended to the CIF file. The SQUEEZE procedure corrected for 1016 electrons within the solvent accessible voids.

3: Hydroxyl H atom positions were refined and O–H distances were restrained to a target value of 0.84(2) Å. The water H atoms were further restrained to be 1.36 Å apart from each other, and one of the water H atoms was restrained based on hydrogen bond considerations (2.00(2) Å from O21).

One of the Bu_4N^+ cations (N57) exhibits disorder of two butyl chains. The other cation exhibits ill-defined whole-cation disorder and was refined as two-fold disordered. Apparent but ill-defined additional disorder was omitted from the refinement model. Associated with this disorder is a partially occupied water molecule, sharing the occupancy of the minor moiety. For the first Bu_4N^+ cation (N57), the disordered butyl chains were restrained to have similar geometries as another better-defined fragment of the same kind. The moieties of the second Bu_4N^+ cation (N58) were restrained to have a similar geometry as the major moiety of the first cation. For all cations, all N–

C distances as well as all N–C–C angles were each restrained to be similar. U_{ij} components of ADPs for disordered atoms closer to each other than 2.0 Å were restrained to be similar. Subject to these conditions the occupancy ratio for butyl chains of the first Bu_4N^+ cation (N63) refined to 0.603(11)/0.397(11) and 0.518(8)/0.485(8). The occupancy ratio for the second cation refined to 0.575(4)/0.425(4).

A nitrobenzene molecule was refined as two-fold disordered. A second nitrobenzene was refined as disordered with two slightly differently oriented bromobenzene molecules. All nitrobenzene moieties and all bromobenzene moieties were each restrained to have similar geometries. The benzene rings of two nitrobenzene molecules were constrained to resemble ideal hexagons with C–C bond distances of 1.39 Å. Subject to these conditions the occupancy ratio refined to 0.754(6)/0.246(6) for the disordered nitrobenzene, and to 0.559(3)/0.310(2)/0.132(3) for the bromobenzene/nitrobenzene/bromobenzene disorder.

The structure contains additional 2444 $Å^3$ of solvent-accessible voids. No substantial electron density peaks were found in the solvent-accessible voids and the residual electron density peaks are not arranged in an interpretable pattern. The structure factors were instead augmented via reverse Fourier transform methods using the SQUEEZE routine as implemented in the program Platon.[2] The resultant FAB file containing the structure factor contribution from the electron content of the void space was used together with the original HKL file for further refinement. The FAB file with details of the SQUEEZE results is appended to the CIF file. The SQUEEZE procedure corrected for 613 electrons within the solvent-accessible voids.

4: The crystal under investigation was found to be non-merohedrally twinned. The orientation matrices for the two components were identified using the program Cell_Now, with the two components being related by a 180° rotation around the reciprocal axis (0 –1 1). The two components were integrated using Saint and corrected for absorption using Twinabs, resulting in the following statistics:

126663 data (24908 unique) involve domain 1 only, mean I/σ 15.0
126218 data (24776 unique) involve domain 2 only, mean I/σ 12.0
162276 data (33787 unique) involve 2 domains, mean I/σ 21.9
3 data (3 unique) involve 3 domains, mean I/σ 28.8

The exact twin matrix identified by the integration program was found to be:

−0.99805	0.00571	−0.00327
0.41940	0.24108	−0.75509
−0.42076	−1.24433	−0.24303

The structure was solved using dual methods with only the non-overlapping reflections of component 1. The structure was refined using the HKLF 5 routine with all reflections of component 1 (including the overlapping ones), resulting in a BASF value of 0.4184(4). The R_{int} value given is for all reflections and is based on agreement between observed single and composite intensities and those calculated from refined unique intensities and twin fractions.[1]

Hydroxyl H atom positions were refined and O–H distances were restrained to 0.84(2) Å.

One non-disordered hexane molecule and another disordered hexane molecule were refined to have similar geometries. One pyrazole ligand was refined as disordered. The two disordered moieties were restrained to have similar geometries as another non-disordered pyrazole ligand. The disorder is correlated with that of the disordered hexane molecule and a common occupancy ratio was used. U_{ij} components of ADPs for disordered atoms closer to each other than 2.0 Å were restrained to be similar. The atoms of the disordered hexane molecule were also restrained to be close to isotropic. Subject to these conditions, the occupancy ratio refined to 0.479(14)/0.521(14).

One butyl group of one Bu_4N^+ cation was refined as disordered. The other Bu_4N^+ cation was refined to exhibit whole-cation disorder. Equivalent disordered moieties were restrained to have similar geometries. U_{ij} components of ADPs for disordered atoms closer to each other than 2.0 Å were restrained to be similar. Subject to these conditions the occupancy ratio refined to 0.562(18)/0.438(18) for the butyl group, and to 0.540(7)/0.460(7) for the whole-cation disorder.

One of three chlorobenzene molecules was refined as non-disordered, one as two-fold disordered, and one as three-fold disordered. All chlorobenzene moieties were restrained to have similar geometries. The moieties of the three-fold disordered molecule were restrained to be close to planar. U_{ij} components of ADPs for disordered atoms closer to each other than 2.0 Å were restrained to be similar. Subject to these conditions the occupancy ratio refined to 0.721(8)/0.279(8), and to 0.382(3)/0.380(3)/0.237(3).

In addition to the twinning and disorder, the structure also exhibits large volume sections consisting of highly disordered solvate or other small molecules. No satisfactory model for the solvate molecules could be developed, and the contribution of the solvate molecules was instead taken into account by reverse Fourier transform methods. The data were first de-twinned (using the LIST 8 function of Shelxl2018) and then the CIF and FCF files were subjected to the SQUEEZE routine as implemented in the program Platon.[2] The resultant files were used in further refinement. Both the HKLF 5 type HKL file and the de-twinned FAB file are appended to the CIF file. A volume of 484 Å^3 per unit cell containing 97 electrons was corrected for.

5: The crystal under investigation was found to be non-merohedrally twinned. The orientation matrices for the two components were identified using the program Cell_Now, with the two components being related by a 180° rotation around the reciprocal *a*-axis. The two components were integrated using Saint and corrected for absorption using Twinabs, resulting in the following statistics:

262773 data (70198 unique) involve domain 1 only, mean I/σ 3.9
261753 data (70097 unique) involve domain 2 only, mean I/σ 3.1
266397 data (66549 unique) involve 2 domains, mean I/σ 5.9

The exact twin matrix identified by the integration program was found to be:

0.99998	0.00020	0.10076
0.00017	−1.00000	0.00000
0.00047	0.00001	−0.99998

The structure was solved using direct methods with only the non-overlapping reflections of component 1. The structure was refined using the HKLF 5 routine with all reflections of component 1 (including the overlapping ones), resulting in a BASF value of 0.4057(4). The R_{int} value given is for all reflections and is based on agreement between observed single and composite intensities and those calculated from refined unique intensities and twin fractions.[1]

The structure features two nanojars of different sizes (with 31 and 32 Cu atoms, respectively) as well as two different types of cations. The major cation sites balancing the charges of the MoO_4^{2-}

anions are four $MePh_3P^+$ cations. An additional Bu_4N^+ cation is present, which is compensated by a mixed nitrate/bromide anion site.

Hydroxyl H atom positions were refined and O–H distances were restrained to a target value of 0.84(2) Å. Ill-defined hydroxyl H atom positions were further restrained based on hydrogen bonding considerations.

One pyrazole ligand (of N27_2) was refined as disordered. The disordered moieties were restrained to have similar geometries as another better-defined pyrazole ligand. U_{ij} components of ADPs for disordered atoms closer to each other than 2.0 Å were restrained to be similar. Subject to these conditions, the occupancy ratio refined to 0.51(3)/0.49(3).

Seven phenyl groups of the $MePh_3P^+$ cations are disordered by slight rotations. The disordered moieties were restrained to have similar geometries as another better-defined phenyl ring. The P–C distances between P1 and C14 and C14B were restrained to be similar. U_{ij} components of ADPs for disordered atoms closer to each other than 2.0 Å were restrained to be similar. Subject to these conditions, the occupancy ratio refined to 0.71(3)/0.29(3) (ring of C2_4), 0.344(15)/0.656(15) (ring of C14_4), 0.39(3)/0.61(3) (ring of C8_5), 0.319(18)/0.681(18) (ring of C14_5), 0.449(15)/0.551(15) (ring of C2_6), 0.690(11)/0.310(11) (ring of C8_6) and 0.56(4)/0.44(4) (ring of C14_6).

The Bu_4N^+ cation (N1_7) exhibits disorder of two butyl chains including the nitrogen atom. The disordered butyl chains were restrained to have similar geometries as another better-defined butyl chain. All N–C distances as well as all N–C–C angles were restrained to be similar to each other. Subject to these conditions, the occupancy ratio refined to 0.383(8)/0.617(8).

The ADPs of the nitrate N atom and the bromide atom were constrained to be identical. The nitrate N–O bonds lengths were restrained to be similar, and the nitrate anion was restrained to be flat. U_{ij} components of ADPs for disordered atoms closer to each other than 2.0 Å were restrained to be similar. Subject to these conditions, the occupancy ratio refined to 0.483(7)/0.517(7) in favor of nitrate.

Several dichlorobenzene molecules were refined as disordered (Cl1/Cl2, Cl5/Cl6) or partially occupied (Cl3/Cl4, Cl7/Cl8, Cl9/Cl10). All but one dichlorobenzene molecule (Cl3/Cl4) were restrained to be flat. All dichlorobenzene moieties were restrained to have similar geometries. U_{ij}

components of ADPs for disordered atoms closer to each other than 2.0 Å were restrained to be similar. Subject to these conditions, the occupancy ratio refined to 0.522(9)/0.478(9) ((Cl1/Cl2) to 0.427(11)/0.366(11) (Cl5/Cl6, site not fully occupied). Occupancy rates refined to 0.741(9) (Cl3/Cl4), 0.631(8) (Cl7/Cl8) and 0.631(9) (Cl9/Cl10). A mild anti-bumping restraint was applied.

In addition to the twinning, the structure also exhibits large volume sections consisting of highly disordered solvate or other small molecules. No satisfactory model for the solvate molecules could be developed, and the contribution of the solvate molecules was instead taken into account by reverse Fourier transform methods. The data were first de-twinned (using the LIST 8 function of Shelxl 2018) and then the CIF and FCF files were subjected to the SQUEEZE routine as implemented in the program Platon.[2] The resultant files were used for further refinement. Both the HKLF 5 type HKL file and the de-twinned FAB file are appended to the CIF file. A volume of 16659 $Å^3$ per unit cell containing 3572 electrons was corrected for.

6: Hydroxyl H atom positions were refined and O–H distances were restrained to 0.84(2) Å.

Four pyrazole ligands (of N3, N7, N21, and N37) were refined as disordered over two positions. Some of the disorder is correlated with solvate molecule disorder (chlorobenzene disorder) and cation disorder (Bu_4N^+), and occupancy ratios are shared with some of the solvate molecules and cation moieties. For the pyrazole of N7, the adjacent Cu atoms (Cu4 and Cu5) were included in the disorder. ADPs of the major and minor Cu atoms were each constrained to be identical. A fifth pyrazole ligand (of N15) shows signs of presence of a minor carboxylate moiety it is disordered with. The alkyl substituent associated with the carboxylate is very ill-defined and modelling of this disorder was omitted. The disordered moieties and this fifth pyrazole were restrained to have similar geometries as another better-defined pyrazole ligand. U_{ij} components of ADPs for the involved atoms closer to each other than 2.0 Å were restrained to be similar. Subject to these conditions the occupancy ratio refined to 0.672(11)/0.328(11) (pyrazole of N3), to 0.675(6)/0.325(6) (N7), to 0.807(17)/0.193(17) (N21) and to 0.582(12)/0.418(12).

Both Bu_4N^+ cations were refined as disordered over two positions each with the exception of atoms C5–C8 of the second cation (residue 4) which were omitted from the disorder. Signs of additional disorder are present, but are not resolved well enough for an unambiguous refinement and were omitted. The disordered moieties were restrained to have similar geometries. All C–N

bond lengths were restrained to be similar in length and C1–C9 and C1B–C9B were restrained to be similar. Some C–C bonds were restrained to expected target values (C1–C2, C2–C3 and C3–C4 for the first cation, C5–C6, C6–C7 and C7–C8 for the second cation). U_{ij} components of ADPs for disordered atoms closer to each other than 2.0 Å were restrained to be similar. Subject to these conditions the occupancy ratio refined to 0.711(3)/0.289(3) for the first cation (residue 3) and 0.398(9)/0.602(9) for the second cation (residue 4).

Three sites occupied by chlorobenzene were refined with two, three and two moieties each (residues 5, 6 and 8, respectively, with the site of residue 8 being not fully occupied). The three-fold disorder of residue 6 is correlated with cation and pyrazole disorder and the distances of C11_1 to C12B_3 and C6B_6 to C12_3 were restrained to be at least 3.30(2) and 3.60(2) Å to avoid unreasonably close contacts. All chlorobenzene moieties were restrained to have similar geometries. The lower occupancy moieties of residue 6 were restrained to be close to planar. U_{ij} components of ADPs for disordered atoms closer to each other than 2.0 Å were restrained to be similar. Subject to these conditions, the solvate occupancy ratio refined to the values listed in the atom table of the CIF file.

A hexane-occupied site was refined as disordered between either two or one hexane molecules. Hexane C–C bonds and angles were restrained to expected target values. U_{ij} components of ADPs for disordered atoms closer to each other than 2.0 Å were restrained to be similar. Subject to these conditions, the occupancy ratio refined to 0.634(11)/0.366(11) in favor of two hexane molecules.

The structure contains an additional 502 Å^3 of solvent accessible voids. No substantial electron density peaks were found in the solvent-accessible voids (less than 1.75 electron per Å^3) and the residual electron density peaks are not arranged in an interpretable pattern. The structure factors were instead augmented via reverse Fourier transform methods using the SQUEEZE routine as implemented in the program Platon.[2] The resultant FAB file containing the structure factor contribution from the electron content of the void space was used together with the original HKL file for further refinement. The FAB file with details of the SQUEEZE results is appended to the CIF file. The SQUEEZE procedure corrected for 90 electrons within the solvent accessible voids.

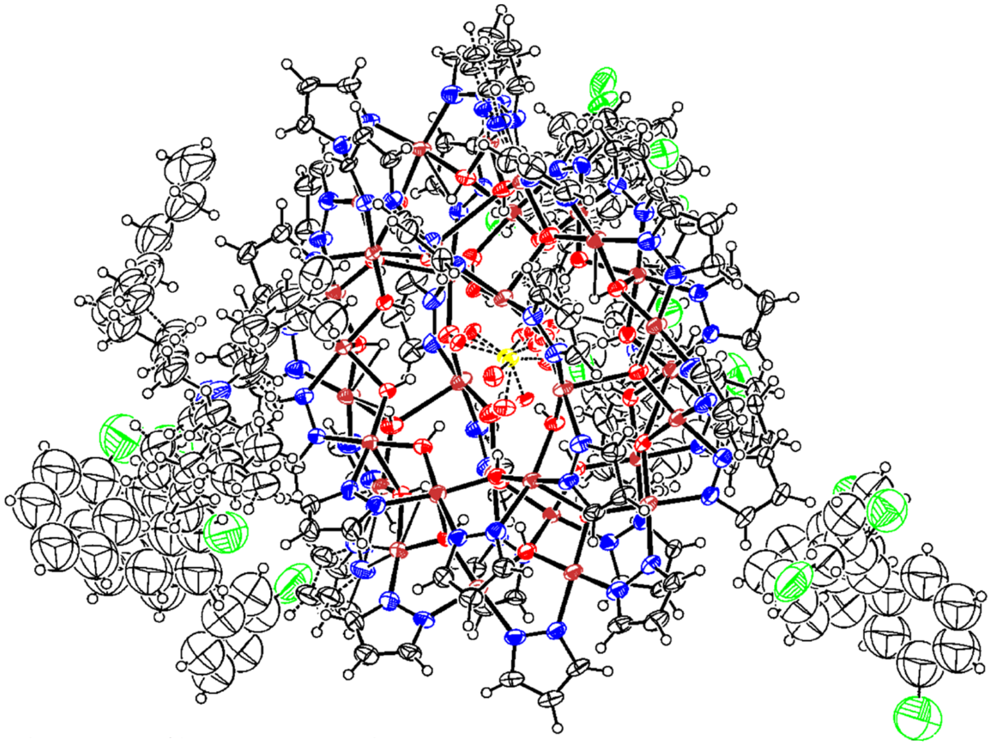

Figure S40. Thermal ellipsoid plot of the crystal structure of **1**.

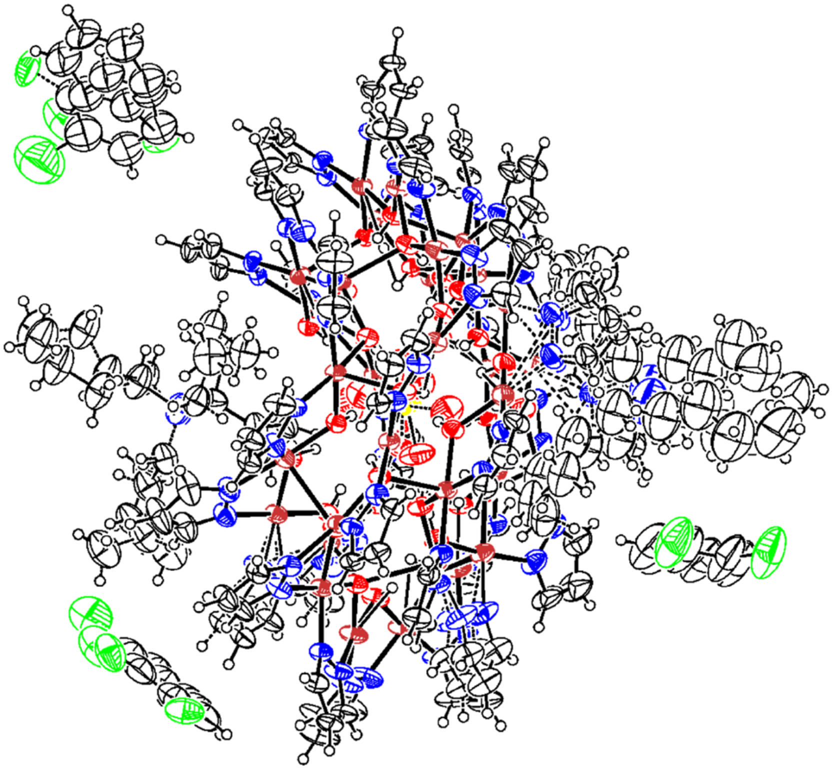

Figure S41. Thermal ellipsoid plot of the crystal structure of **2**.

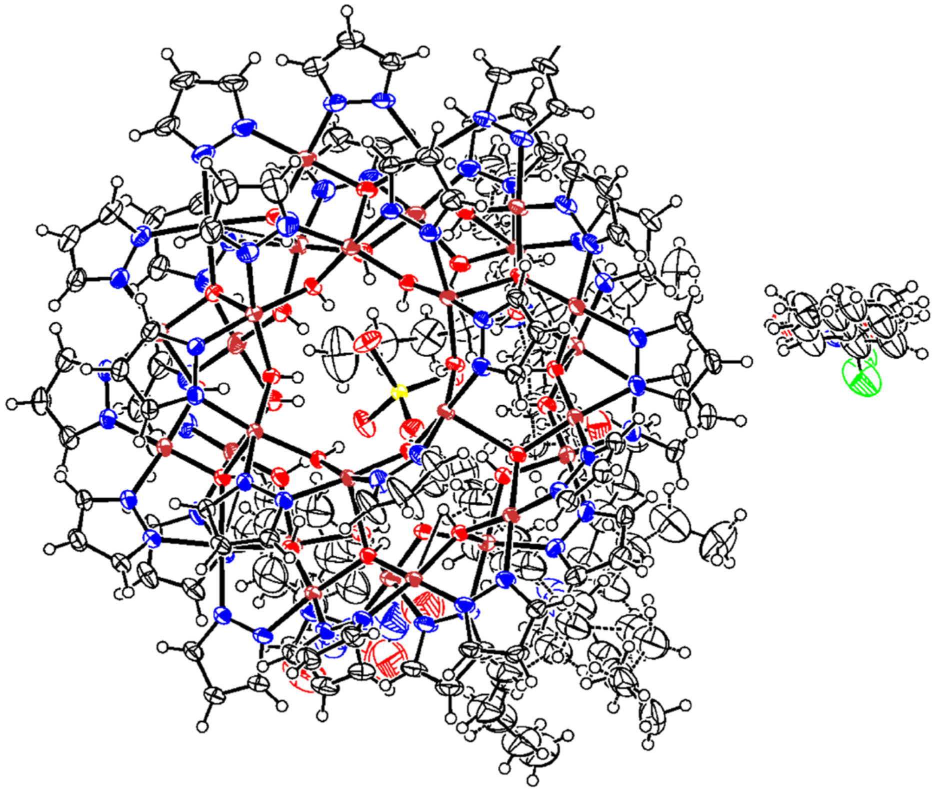

Figure S42. Thermal ellipsoid plot of the crystal structure of **3**.

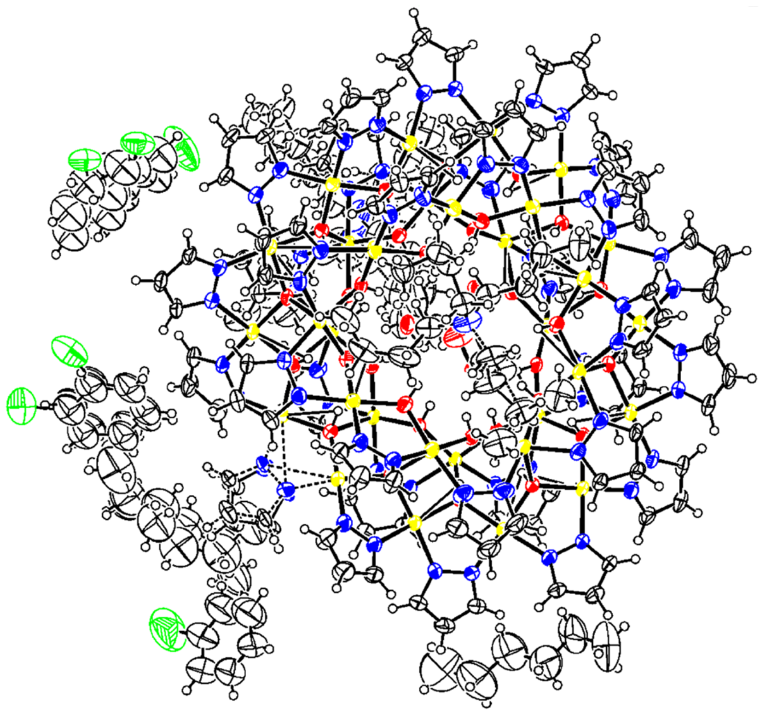

Figure S43. Thermal ellipsoid plot of the crystal structure of **4**.

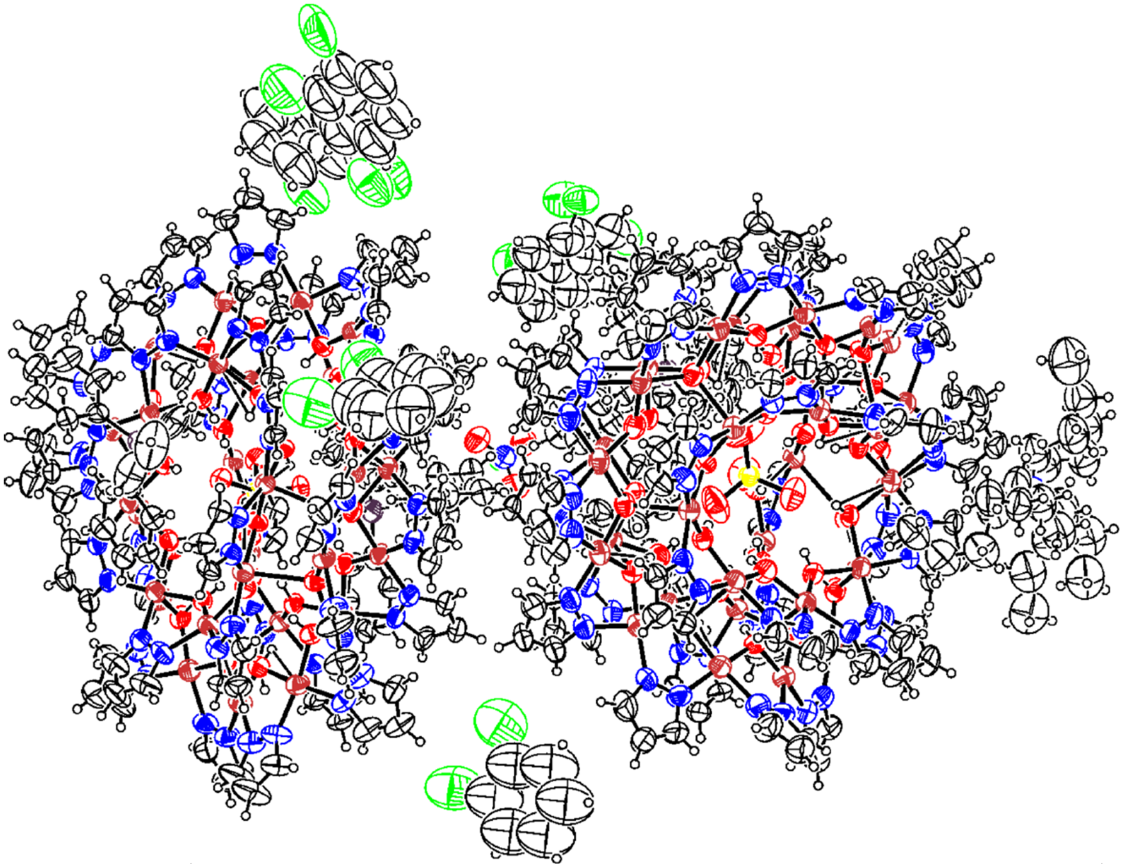

Figure S44. Thermal ellipsoid plot of the crystal structure of **5**.

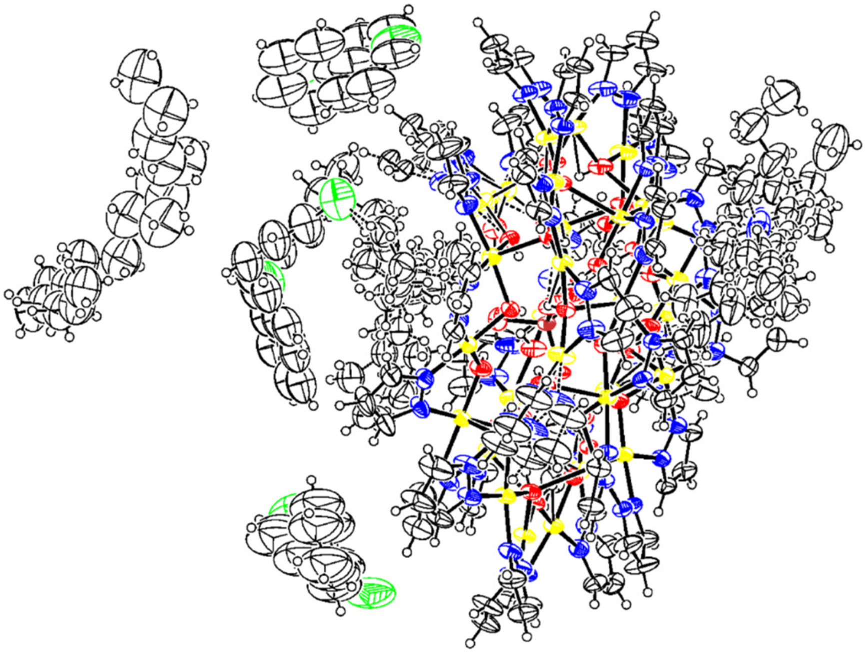

Figure S45. Thermal ellipsoid plot of the crystal structure of **6**.

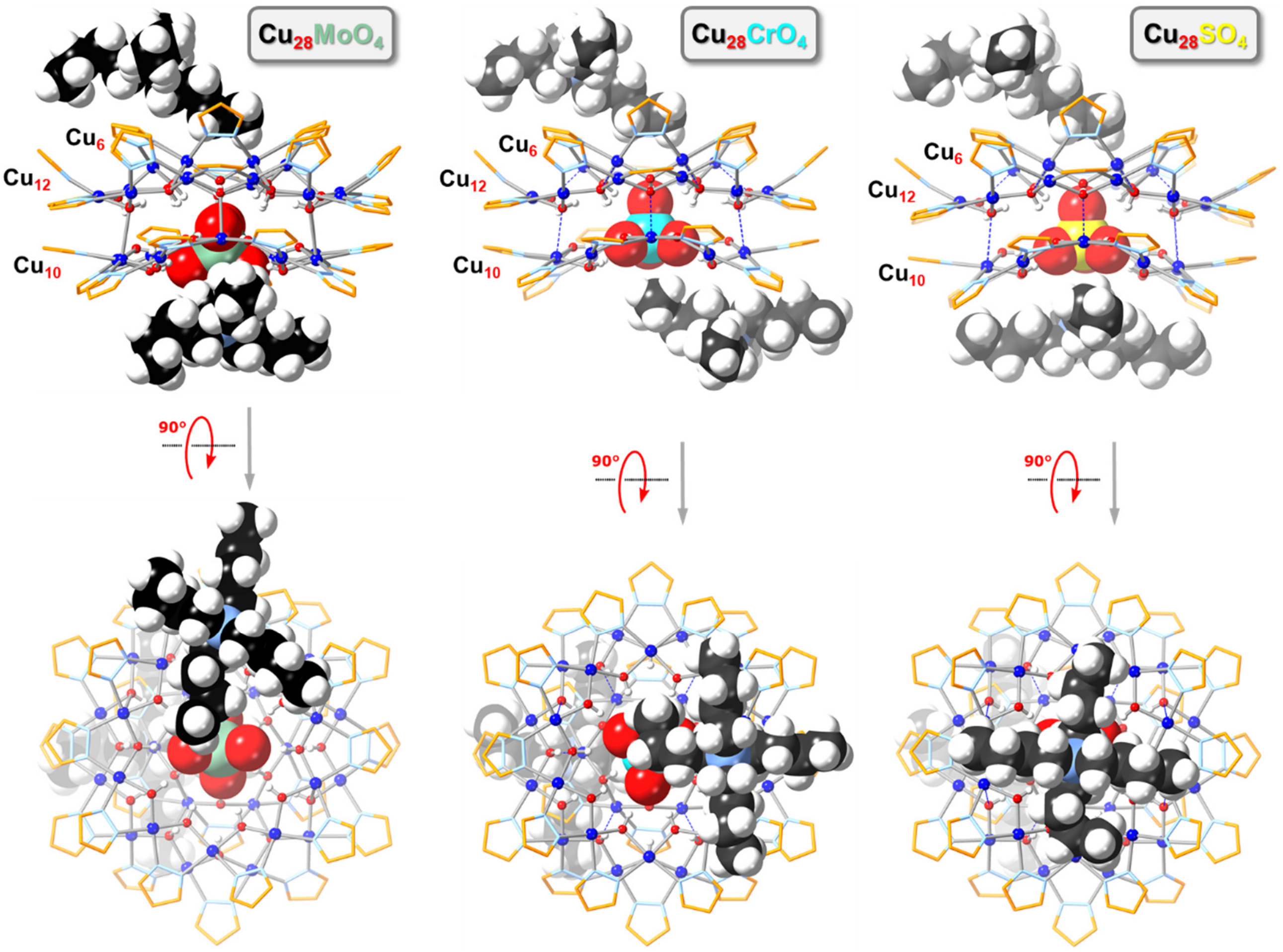

Figure S46. Comparison (side- and top-views) of the position of the two Bu_4N^+ counterions relative to the nanojar in the X-ray crystal structures of $\mathbf{Cu_{28}EO_4}$ (E = Mo, Cr, S). Pyrazole H atoms are omitted for clarity and only the major component is shown for disordered moieties.

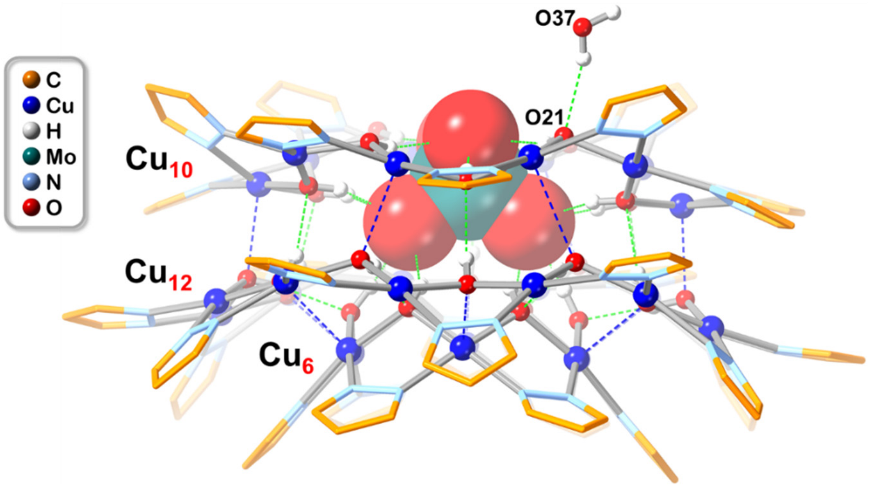

Figure S47. Illustration of the H-bonding between the Cu_{10} ring of $\mathbf{Cu_{28}MoO_4}$ and an H_2O molecule. Pyrazole H atoms are omitted for clarity.

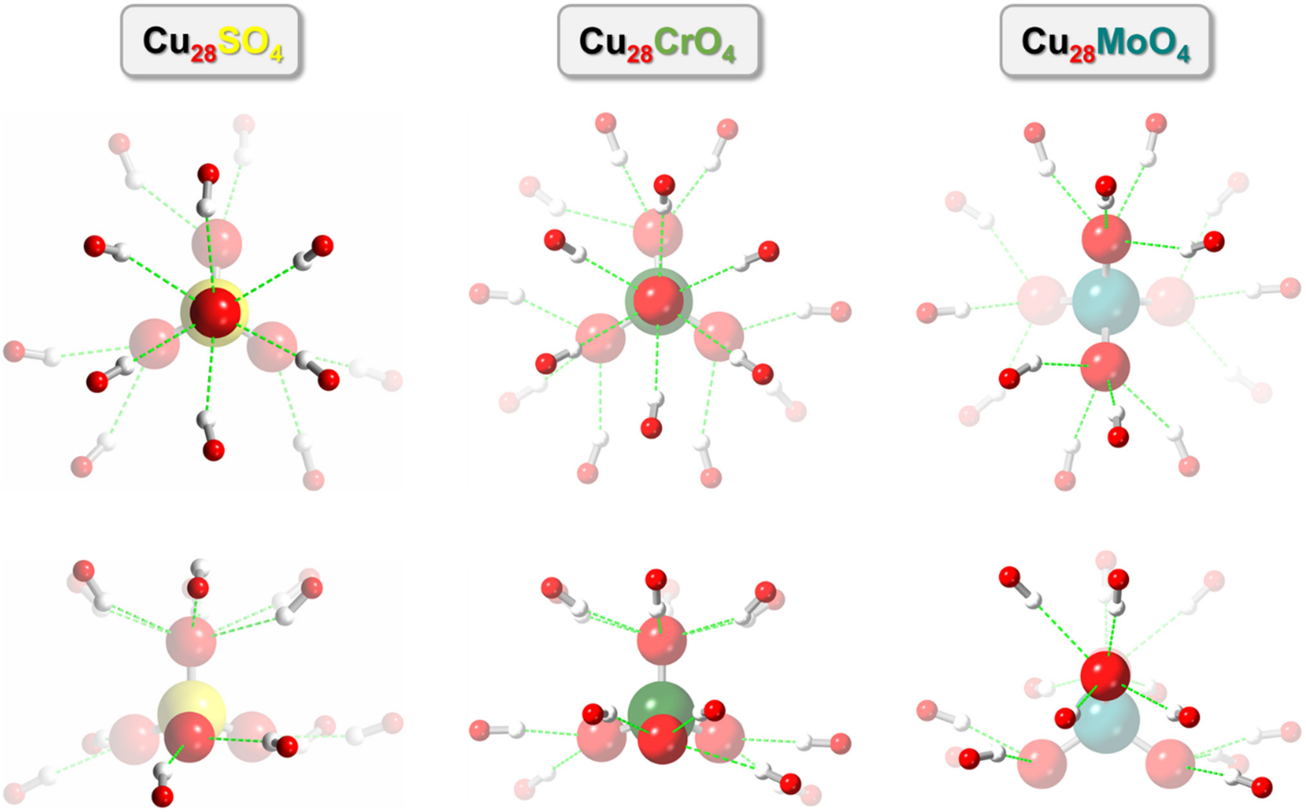

Figure S48. Comparison of the H-bonding patterns in different $\mathbf{Cu_{28}EO_4}$ (E = S, Cr, Mo) nanojars.

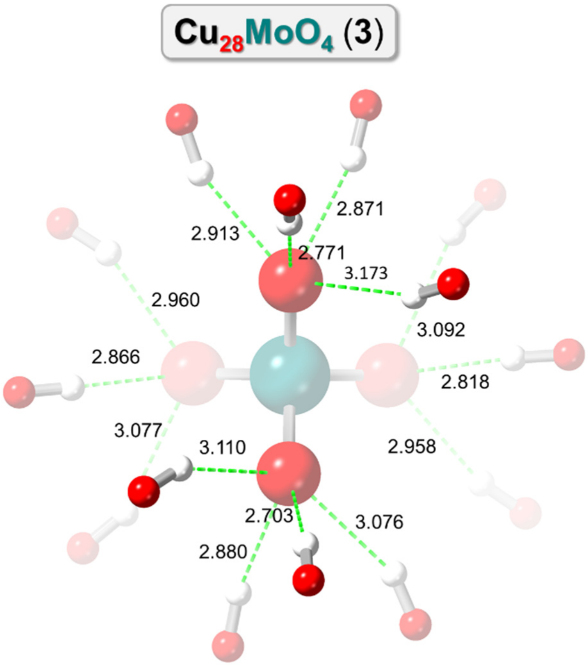

Figure S49. Details of the H-bonding to the MoO_4^{2-} ion in $\mathbf{Cu_{28}MoO_4}$ (**3**), showing donor-acceptor O⋯O distances in Å.

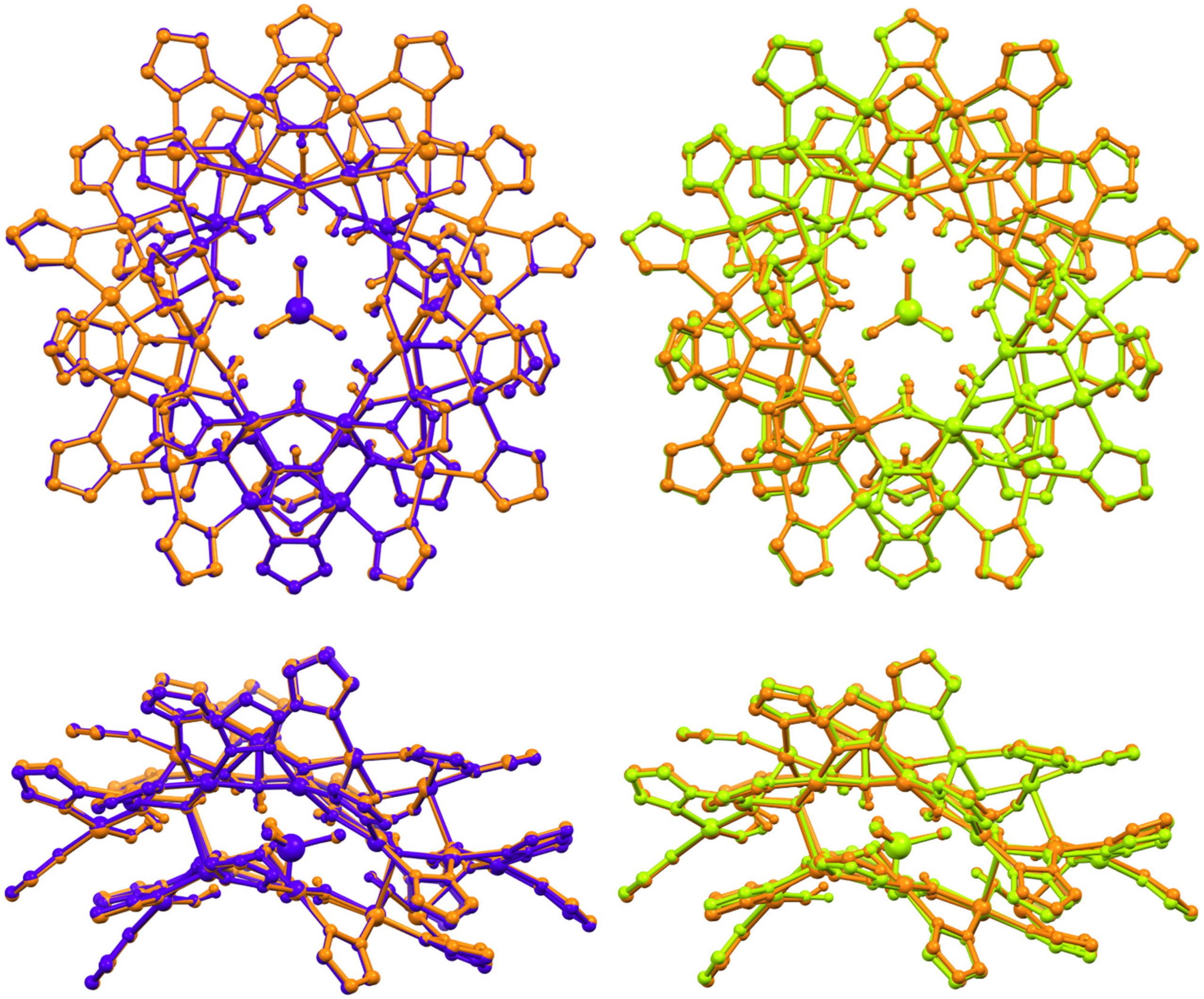

Figure S50. Overlays (top- and side-views) of the crystal structure of $\mathbf{Cu_{31}SeO_4}$ (**1**) (orange) with those of $\mathbf{Cu_{31}MoO_4}$ (**4**) (blue) and $\mathbf{Cu_{31}WO_4}$ (**6**) (green). C–H bond hydrogen atoms, counterions and solvent molecules are omitted for clarity, and only the major component is shown for disordered moieties.

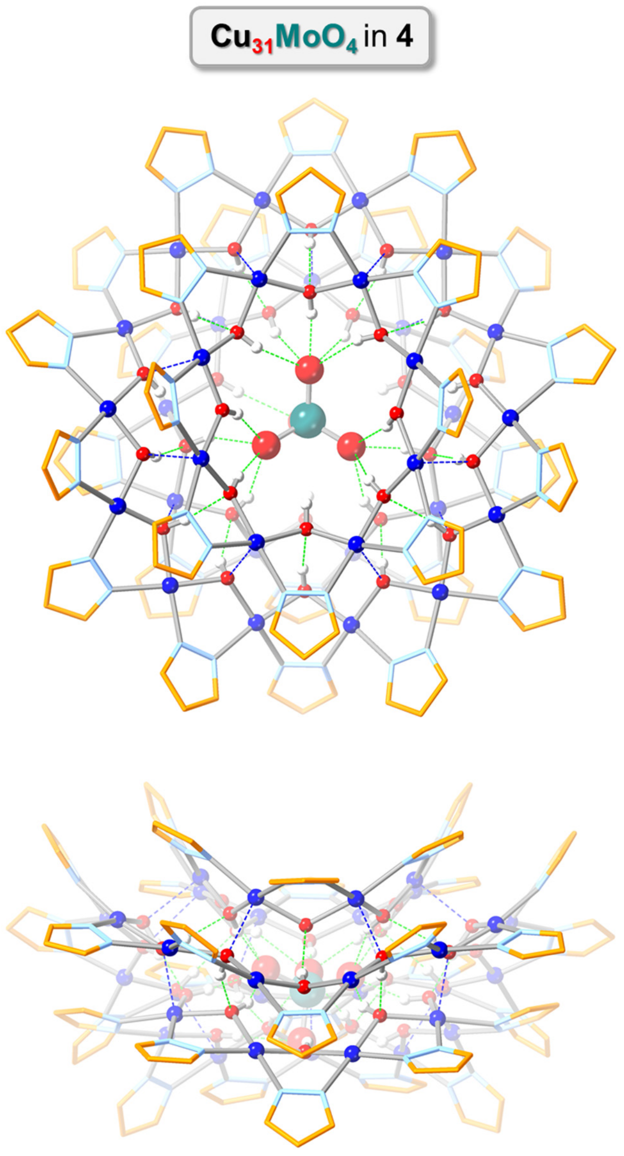

Figure S51. Comparison of the crystal structures of **$Cu_{31}MoO_4$** in **4** and **5** (top- and side-views), showing the opposite orientation of the MoO_4^{2-} anion within the nanojar cavity. C–H bond hydrogen atoms, counterions and solvent molecules are omitted for clarity, and only the major component is shown for disordered moieties.

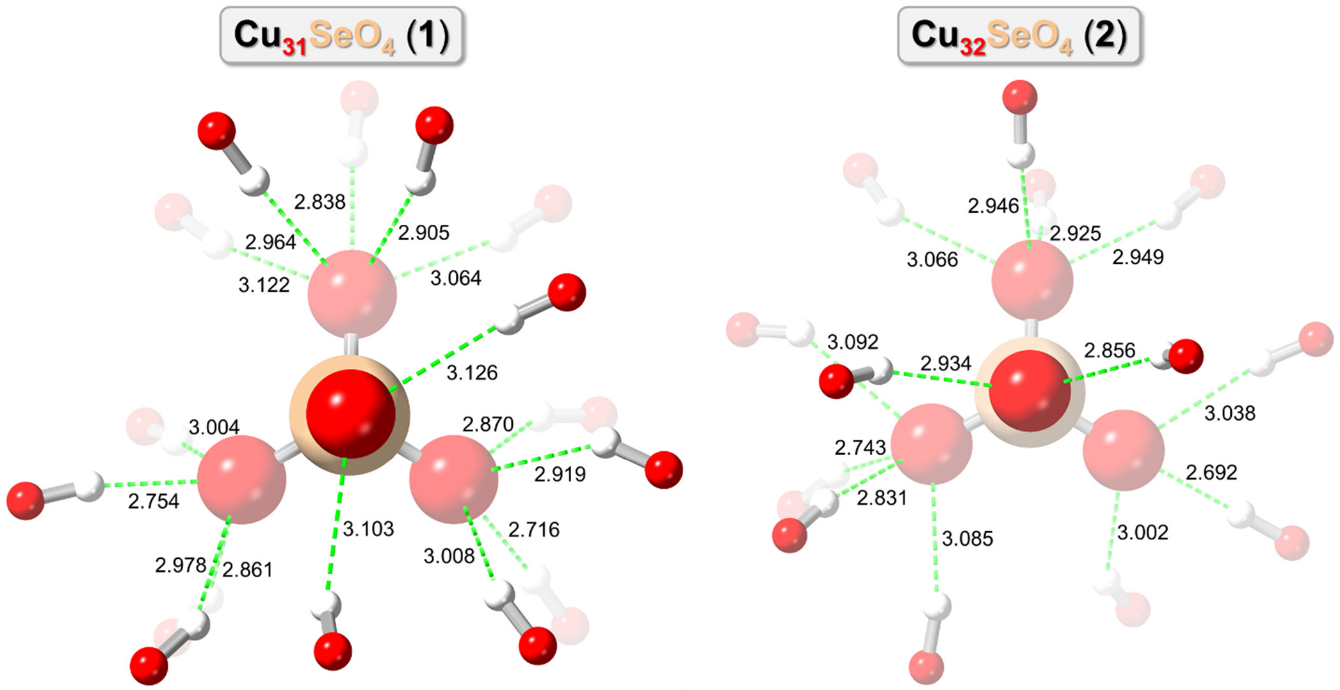

Figure S52. Details of the H-bonding to the SeO_4^{2-} ion in **$Cu_{31}SeO_4$** (**1**) and **$Cu_{32}SeO_4$** (**2**), showing donor-acceptor O⋯O distances in Å.

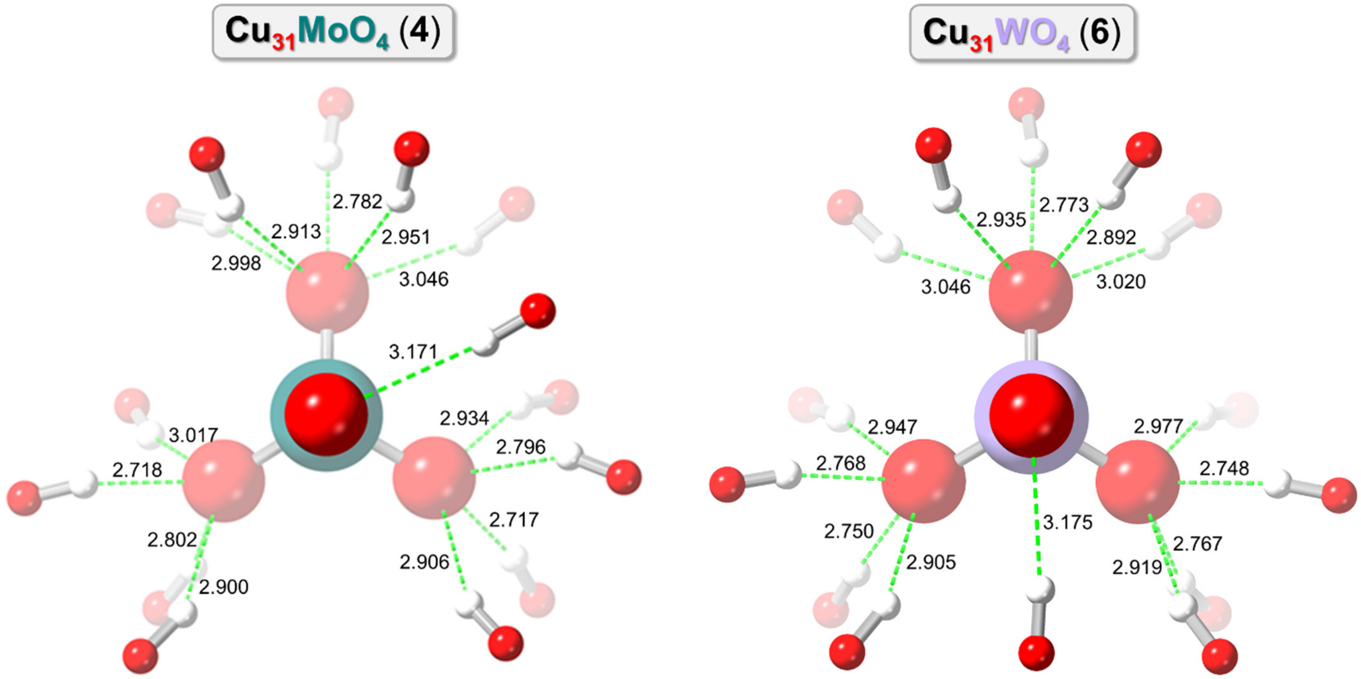

Figure S53. Details of the H-bonding to the MoO_4^{2-} and WO_4^{2-} ions in **$Cu_{31}MoO_4$** (**4**) and **$Cu_{31}WO_4$** (**6**), showing donor-acceptor O⋯O distances in Å.

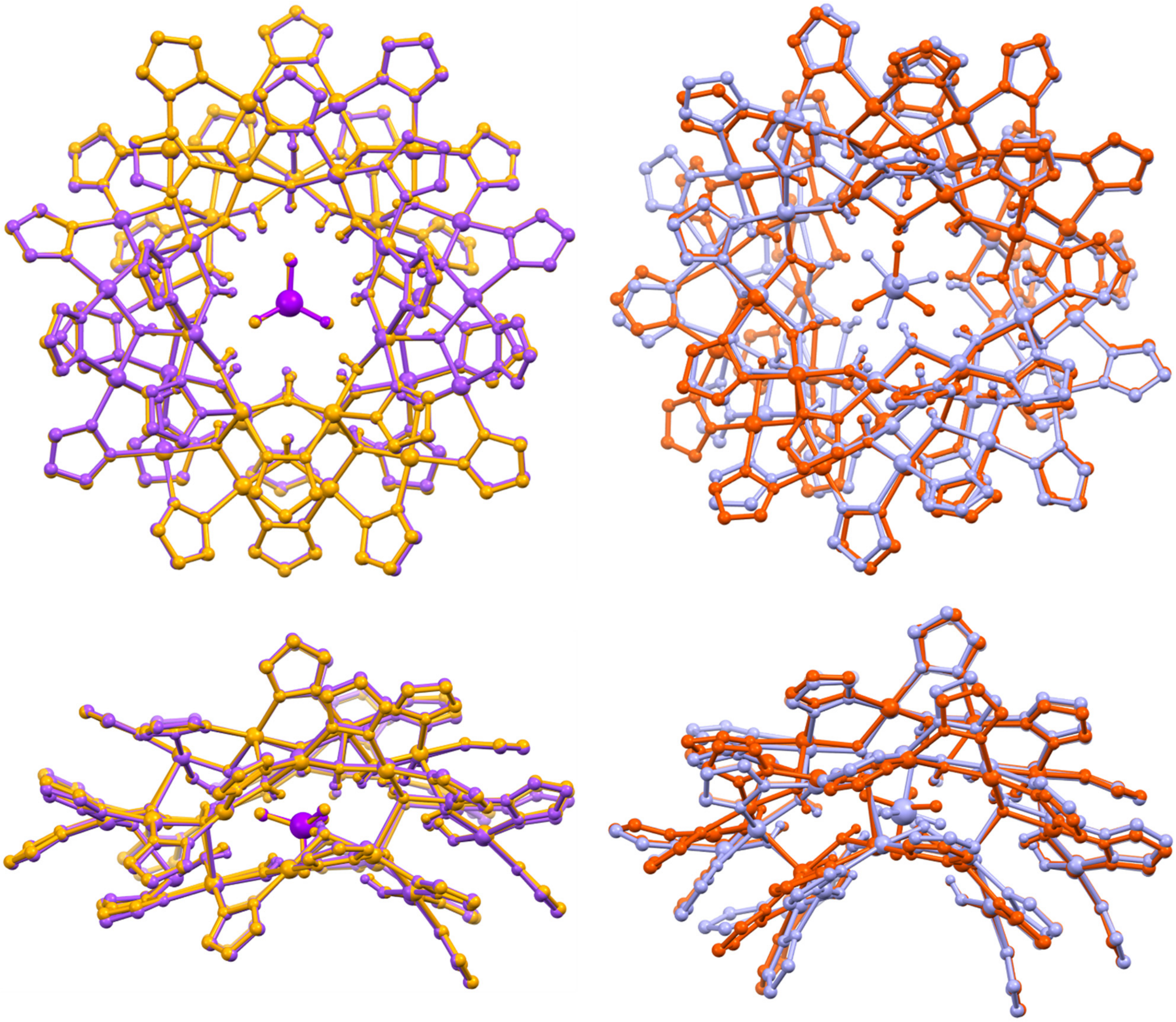

Figure S54. Overlays (top- and side-views) of the crystal structure of $\mathbf{Cu_{31}SeO_4}$ (**1**) (orange) with that of $\mathbf{Cu_{31}BeF_4}$ (dark violet) on the left, and of $\mathbf{Cu_{32}SeO_4}$ (**2**) (persimmon) with that of $\mathbf{Cu_{32}BeF_4}$ (lavender) on the right. C–H bond hydrogen atoms, counterions and solvent molecules are omitted for clarity, and only the major component is shown for disordered moieties.

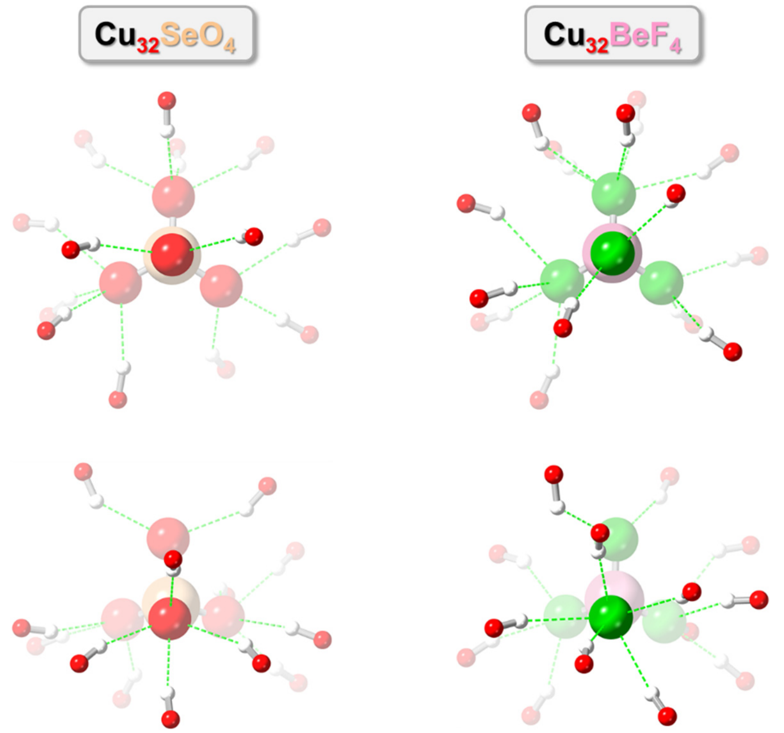

Figure S55. Comparison of the H-bonding patterns in **$Cu_{9+14+9}SeO_4$** (**2**) and **$Cu_{9+14+9}BeF_4$** (only the major orientation of the disordered anions is shown).

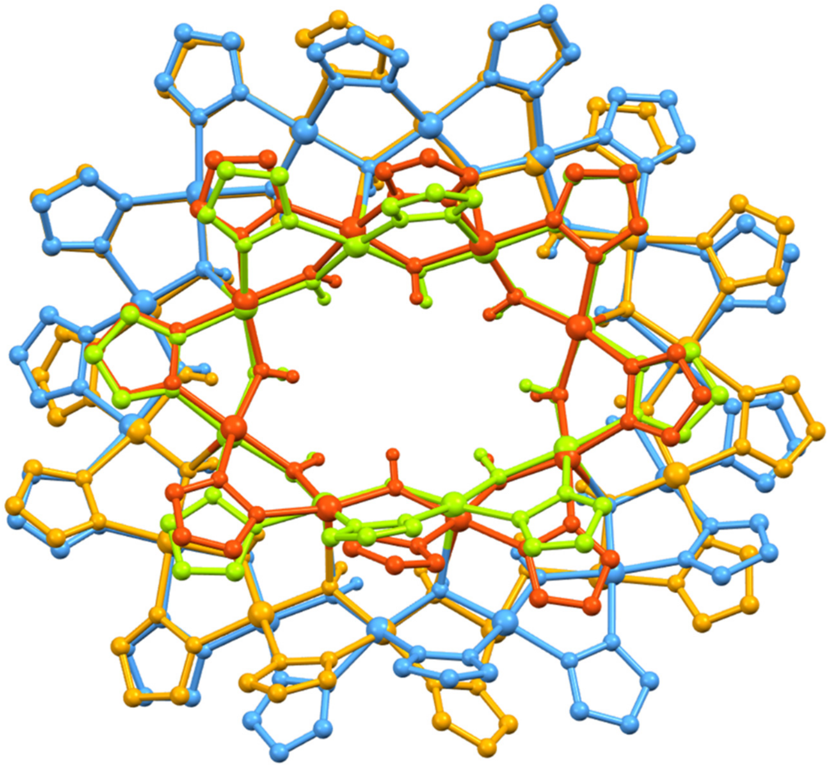

Figure S56. Overlay of the Cu_8 and Cu_{14} rings found in the crystal structure of the cocrystallized **$Cu_{8+14+9}MoO_4$** (Cu_8 – persimmon; Cu_{14} – orange) and **$Cu_{8+14+10}MoO_4$** (Cu_8 – lime; Cu_{14} – blue) (**5**).

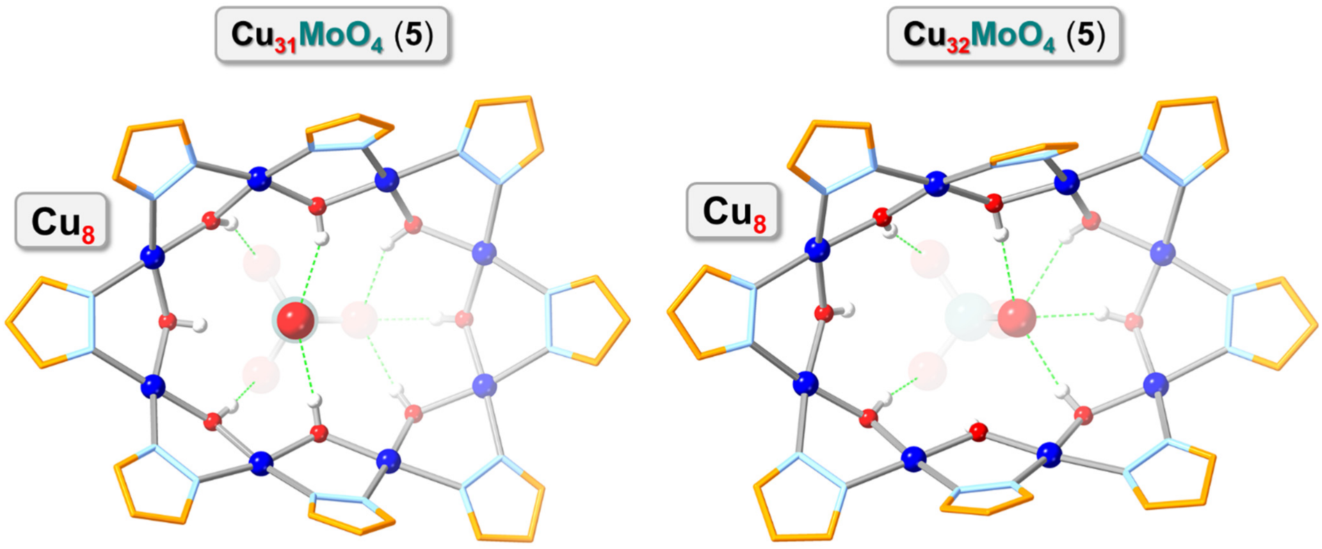

Figure S57. Comparison of the H-bonding to the MoO_4^{2-} ion to the Cu_8 ring in **$Cu_{31}MoO_4$** (**5**) and **$Cu_{32}MoO_4$** (**5**).

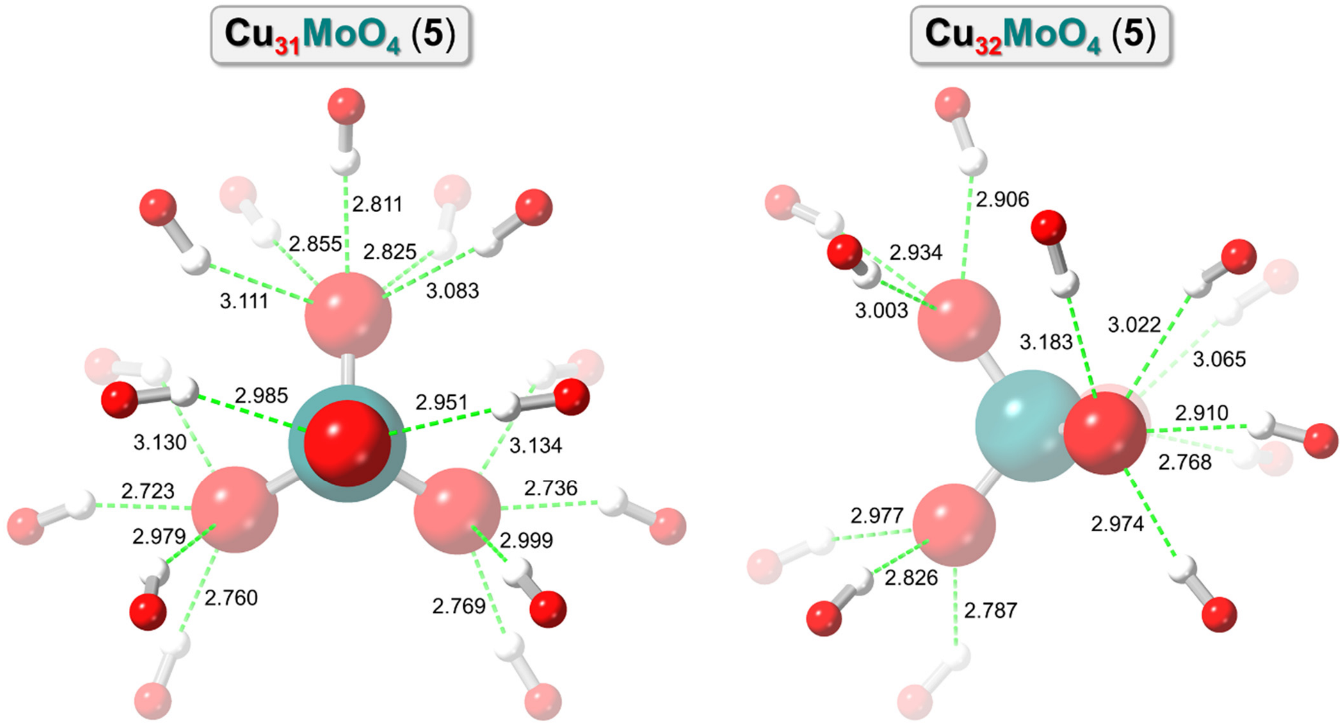

Figure S58. Details of the H-bonding to the MoO_4^{2-} ion in **$Cu_{31}MoO_4$** (**5**) and **$Cu_{32}MoO_4$** (**5**), showing donor-acceptor O···O distances in Å.

Table S4. Crystallographic data of the selenate (**1**, **2**) and tungstate (**6**) nanojars.

	**1**	**2**	**6**
Formula (sum)	$C_{154.86}H_{227.35}Cl_{4.29}Cu_{31}N_{64}O_{35}Se$	$C_{143.44}H_{210.29}Cl_{5.15}Cu_{32}N_{66}O_{36}Se$	$C_{152.08}H_{233.99}Cl_{2.80}Cu_{31}N_{64}O_{35}W$
Formula (moiety)	$C_{93}H_{124}Cu_{31}N_{62}O_{31}$, SeO_4, 2($C_{16}H_{36}N$), 4.29(C_6H_5Cl), 0.83(C_5H_{12}), solvent	$C_{96}H_{128}Cu_{32}N_{64}O_{32}$, SeO_4, 2($C_{16}H_{36}N$), 2.57($C_6H_4Cl_2$), solvent	$C_{93}H_{124}Cu_{31}N_{62}O_{31}$, WO_4, 2($C_{16}H_{36}N$), 2.80(C_6H_5Cl), 1.71(C_6H_{14}), solvent
FW (g·mol^{-1})	5746.46	5730.28	5771.89
Crystal system	Triclinic	Orthorhombic	Triclinic
Space group	$P\bar{1}$ (No. 2)	$P2_12_12_1$ (No. 19)	$P\bar{1}$ (No. 2)
a (Å)	20.6474(7)	24.7735(11)	20.6021(5)
b (Å)	23.0756(7)	29.2061(13)	23.0223(5)
c (Å)	25.6604(8)	31.8218(15)	25.9041(6)
α (deg)	103.1190(10)	90.000	103.5890(10)
β (deg)	94.9090(10)	90.000	94.978(2)
γ (deg)	110.7890(10)	90.000	110.9230(10)
V (Å^3)	10944.8(6)	23024.3(18)	10956.2(4)
Z	2	4	2
D_{calc} (g·cm^{-3})	1.744	1.653	1.750
μ (mm^{-1})	4.433	4.394	5.000
θ range (deg)	1.80–80.03	2.72–79.75	1.79–80.34
Reflns collected	164816	130717	115676
R_{int}	0.0624	0.0686	0.0535
Obsd reflns [$I > 2\sigma(I)$]	60688	37940	35432
Data/restraints/parameters	68822/4832/3671	45550/3603/3307	43818/3810/3400
GOF (on F^2)	1.012	1.036	1.045
R factors [$I > 2\sigma(I)$]	R_1 = 0.0600<br>wR_2 = 0.1591	R_1 = 0.0482<br>wR_2 = 0.1227	R_1 = 0.0664<br>wR_2 = 0.1534
R factors (all data)	R_1 = 0.0656<br>wR_2 = 0.1628	R_1 = 0.0623<br>wR_2 = 0.1329	R_1 = 0.0819<br>wR_2 = 0.1625
Maximum peak/hole (e·Å^{-3})	2.001/−0.886	1.326/−0.715	1.677/−1.743
CCDC number	2392070	2392071	2392075

Table S5. Crystallographic data of the molybdate nanojars (**3–5**).

	**3**	**4**	**5**
Formula (sum)	$C_{128}H_{194.86}Br_{0.69}Cu_{28}MoN_{59.31}O_{35.05}$	$C_{155}H_{239}Cl_{3}Cu_{31}MoN_{64}O_{35}$	$C_{303.78}H_{375.19}Br_{0.48}Cl_{7.59}Cu_{63}Mo_{2}N_{127.52}O_{72.55}P_{4}$
Formula (moiety)	$C_{84}H_{112}Cu_{28}N_{56}O_{28}$, MoO_4, 2($C_{16}H_{36}N$), 1.31($C_6H_5NO_2$), 0.69(C_6H_5Br), 0.43(H_2O), solvent	$C_{93}H_{124}Cu_{31}N_{62}O_{31}$, MoO_4, 2($C_{16}H_{36}N$), 3(C_6H_5Cl), 2(C_6H_{14}), solvent	$C_{96}H_{128}Cu_{32}N_{64}O_{32}$, $C_{93}H_{124}Cu_{31}N_{62}O_{31}$, 2($MoO_4$), 4($C_{19}H_{18}P$), $C_{16}H_{36}N$, 0.52(NO_3), 0.48(Br), 3.80($C_6H_4Cl_2$), solvent
FW (g·mol^{-1})	5051.60	5731.12	11601.09
Crystal system	Monoclinic	Triclinic	Monoclinic
Space group	$P2_1$/n (No. 14)	$P\bar{1}$ (No. 2)	$P2_1$/c (No. 14)
a (Å)	24.124(7)	20.617(2)	36.178(9)
b (Å)	34.334(4)	23.078(2)	33.141(7)
c (Å)	25.134(6)	25.866(2)	46.436(10)
α (deg)	90.000	103.431(5)	90.000
β (deg)	109.28(3)	94.865(6)	93.698(10)
γ (deg)	90.000	110.769(7)	90.000
V (Å^3)	19651(8)	11001.8(19)	55558(22)
Z	4	2	4
D_{calc} (g·cm^{-3})	1.708	1.730	1.387
μ (mm^{-1})	3.210	4.521	3.845
θ range (deg)	2.04–32.06	1.79–80.14	1.81–70.27
Reflns collected	386912	283911	528143
R_{int}	0.0422	0.0754	0.0974
Obsd reflns [$I > 2σ(I)$]	51761	42244	70565
Data/restraints/parameters	68476/2128 /2807	46229/2195/3196	103776/5237/6156
GOF (on F^2)	1.095	1.013	1.004
R factors [$I > 2\ σ(I)$]	R_1 = 0.0453<br>wR_2 = 0.1120	R_1 = 0.0470<br>wR_2 = 0.1235	R_1 = 0.0660<br>wR_2 = 0.1520
R factors (all data)	R_1 = 0.0674<br>wR_2 = 0.1313	R_1 = 0.0541<br>wR_2 = 0.1301	R_1 = 0.1025<br>wR_2 = 0.1760
Maximum peak/hole (e·Å^{-3})	1.219/−1.246	0.893/−0.938	1.161/−0.628
CCDC number	2392072	2392073	2392074

Table S6. Comparison of bond lengths (Å) and angles (°), Cu···O distances shorter than the sum of the van der Waals radii of Cu and O (2.92 Å) and H-bonding (with D···A distances shorter than 3.2 Å) in **1**, **2** and **6** (based on the major component in the case of disordered moieties).

	**$Cu_{31}SeO_4$ (1)**	**$Cu_{32}SeO_4$ (2)**	**$Cu_{31}WO_4$ (6)**
Cu–O within Cu_n rings	1.896(4)–1.960(4)<br>avg: 1.926(4)	1.895(7)–1.970(6)<br>avg: 1.931(7)	1.864(6)–1.976(6)<br>avg: 1.924(6)
Cu–N within Cu_n rings	1.945(6)–2.024(5)<br>avg: 1.975(6)	1.941(9)–2.021(8)<br>avg: 1.973(8)	1.924(8)–2.031(6)<br>avg: 1.973(7)
N–Cu–O (*trans*) within Cu_{14} ring	162.5(3)–175.5(2)<br>avg: 169.7(2)	164.2(3)–176.6(3)<br>avg: 171.5(4)	163.3(8)–175.1(2)<br>avg: 169.4(3)
N–Cu–O (*trans*) within Cu_9 ring	169.44(19)–179.7(3)<br>avg: 174.2(2)	166.9(3)–178.5(4)<br>avg: 173.1(4)	169.7(4)–177.8(4)<br>avg: 173.6(3)
N–Cu–O (*trans*) within Cu_8 or Cu_9 ring	166.1(2)–177.2(2)<br>avg: 172.3(2)	160.2(4)–178.4(6)<br>avg: 172.4(4)	165.2(2)–177.9(2)<br>avg: 172.3(2)
Average of all N–Cu–O (trans) angles	*172.1(2)*	*172.3(4)*	*171.8(3)*
N–Cu–O (*cis*) within Cu_{14} ring	86.3(2)–88.94(19)<br>avg: 87.8(2)	85.3(3)–88.9(3)<br>avg: 87.2(3)	85.4(2)–89.9(5)<br>avg: 87.7(2)
N–Cu–O (*cis*) within Cu_9 ring	82.93(18)–87.0(2)<br>avg: 84.5(2)	81.1(6)–87.0(9)<br>avg: 84.9(3)	80.5(3)–87.7(3)<br>avg: 84.9(3)
N–Cu–O (*cis*) within Cu_8 or Cu_9 ring	82.37(18)–86.5(2)<br>avg: 84.5(2)	81.8(6)–86.3(3)<br>avg: 85.0(3)	82.5(2)–86.2(2)<br>avg: 84.3(2)
Average of all N–Cu–O (cis) angles	*85.6(2)*	*85.7(3)*	*85.6(2)*
Cu···O between Cu_9 and Cu_{14} rings	2.393(4)–2.718(3)<br>(5 interactions)<br>avg: 2.523(4)	2.366(6)–2.807(6)<br>(7 interactions)<br>avg: 2.515(7)	2.377(5)–2.683(6)<br>(5 interactions)<br>avg: 2.506(5)
Cu···O between Cu_8 (or Cu_9) and Cu_{14} rings	2.418(4)–2.889(3)<br>(8 interactions)<br>avg: 2.529(5)	2.362(7)–2.916(6)<br>(7 interactions)<br>avg: 2.526(7)	2.439(5)–2.901(5)<br>(8 interactions)<br>avg: 2.543(5)
Average of all Cu···O interactions between Cu_n rings	*2.526(5)*<br>*(13 interactions)*	*2.520(7)*<br>*(14 interactions)*	*2.524(5)*<br>*(13 interactions)*
Cu···Cu distances in Cu_{14} ring	3.1416(13)–3.3693(17)<br>avg: 3.256(2)	3.1781(15)–3.3595(18)<br>avg: 3.282(2)	3.1582(16)–3.373(2)<br>avg: 3.262(2)
Cu···Cu distances in Cu_9 ring	3.1368(15)–3.3948(13)<br>avg: 3.299(2)	3.140(2)–3.403(2)<br>avg: 3.302(2)	3.1700(18)–3.4276(14)<br>avg: 3.307(2)
Cu···Cu distances in Cu_8 (or Cu_9) ring	3.3094(14)–3.3982(11)<br>avg: 3.356(2)	3.1359(15)–3.4308(18)<br>avg: 3.302(2)	3.3203(17)–3.3922(13)<br>avg: 3.363(2)
Average of all Cu···Cu distances in Cu_n rings	*3.304(2)*	*3.295(2)*	*3.311(2)*
H-bonded O···O between Cu_9 and Cu_{14} rings	2.794(7)–2.904(6)<br>(6 interactions)<br>avg: 2.845(7)	2.714(8)–2.940(8)<br>(7 interactions)<br>avg: 2.816(9)	2.785(8)–2.885(7)<br>(6 interactions)<br>avg: 2.824(7)
H-bonded O···O between Cu_8 (or Cu_9) and Cu_{14} rings	2.791(6)–2.916(5)<br>(6 interactions)<br>avg: 2.858(6)	2.750(8)–2.963(10)<br>(7 interactions)<br>avg: 2.800(8)	2.781(7)–2.941(5)<br>(6 interactions)<br>avg: 2.855(7)
Average of all H-bonded O···O distances between Cu_n rings	*2.851(7)*<br>*(12 interactions)*	*2.808(8)*<br>*(14 interactions)*	*2.839(7)*<br>*(12 interactions)*
H-bonded O···O distance range between Cu_n rings and SeO_4^{2-} (or WO_4^{2-})	2.716(11)–3.125(11)<br>(15 interactions)<br>avg: 2.95(1)	2.693(12)–3.094(13)<br>(13 interactions)<br>avg: 2.94(1)	2.748(7)–3.175(12)<br>(14 interactions)<br>avg: 2.90(1)

Table S7. Comparison of bond lengths (Å) and angles (°), Cu···O distances shorter than the sum of the van der Waals radii of Cu and O (2.92 Å) and H-bonding (with D···A distances shorter than 3.2 Å) in **3**–**5** (based on the major component in the case of disordered moieties).

	**$Cu_{28}MoO_4$ (3)**	**$Cu_{31}MoO_4$ (4)**	**$Cu_{31}MoO_4$ (5)**	**$Cu_{32}MoO_4$ (5)**
Cu–O within Cu_n rings	1.896(2)–1.957(2)<br>avg: 1.931(2)	1.891(4)–1.970(4)<br>avg: 1.926(4)	1.891(6)–1.977(6)<br>avg: 1.925(6)	1.890(7)–1.979(6)<br>avg: 1.930(7)
Cu–N within Cu_n rings	1.947(3)–2.019(3)<br>avg: 1.976(3)	1.946(5)–2.025(4)<br>avg: 1.977(5)	1.931(9)–2.015(8)<br>avg: 1.970(8)	1.938(8)–2.007(8)<br>avg: 1.969(8)
N–Cu–O (*trans*) within Cu_{12} or Cu_{14} ring	165.71(11)–175.32(11)<br>avg: 171.8(1)	161.6(13)–175.60(18)<br>avg: 169.5(2)	160.9(3)–175.2(3)<br>avg: 169.4(3)	163.6(3)–174.8(3)<br>avg: 169.9(3)
N–Cu–O (*trans*) within Cu_{10} or Cu_9 ring	154.43(12)–177.37(12)<br>avg: 171.3(1)	169.82(18)–177.85(19)<br>avg: 174.1(2)	168.5(3)–176.8(3)<br>avg: 173.2(3)	169.3(3)–177.7(3)<br>avg: 173.5(3)
N–Cu–O (*trans*) within Cu_6, Cu_8 or Cu_9 ring	166.31(14)–175.88(10)<br>avg: 171.0(1)	165.32(17)–177.65(16)<br>avg: 172.3(2)	163.7(3)–177.7(3)<br>avg: 171.0(3)	165.9(3)–177.1(3)<br>avg: 171.5(3)
Average of all N–Cu–O (trans) angles	*171.4(1)*	*172.0(2)*	*171.2(3)*	*171.6(3)*
N–Cu–O (*cis*) within Cu_{12} or Cu_{14} ring	85.56(10)–87.39(12)<br>avg: 86.6(1)	86.16(18)–88.98(16)<br>avg: 87.7(2)	85.1(3)–89.2(3)<br>avg: 87.4(3)	84.4(3)–88.9(3)<br>avg: 87.0(3)
N–Cu–O (*cis*) within Cu_{10} or Cu_9 ring	83.80(10)–87.63(11)<br>avg: 85.3(1)	83.13(17)–87.55(18)<br>avg: 84.7(2)	82.0(3)–86.9(3)<br>avg: 84.4(3)	84.5(3)–88.0(3)<br>avg: 85.6(3)
N–Cu–O (*cis*) within Cu_6, Cu_8 or Cu_9 ring	83.95(10)–85.52(9)<br>avg: 84.7(1)	81.90(16)–86.41(18)<br>avg: 84.2(2)	82.2(3)–87.0(3)<br>avg: 84.6(3)	83.3(3)–88.4(3)<br>avg: 85.2(3)
Average of all N–Cu–O (cis) angles	*85.5(1)*	*85.5(2)*	*85.5(3)*	*85.9(3)*
Cu···O between Cu_{10} (or Cu_9) and Cu_{12} (or Cu_{14}) rings	2.329(2)–2.850(2)<br>(5 interactions)<br>avg: 2.591(2)	2.386(3)–2.672(4)<br>(5 interactions)<br>avg: 2.510(4)	2.333(5)–2.702(7)<br>(5 interactions)<br>avg: 2.516(6)	2.387(6)–2.910(7)<br>(6 interactions)<br>avg: 2.579(7)
Cu···O between Cu_6 (or Cu_8 or Cu_9) and Cu_{12} (or Cu_{14}) rings	2.349(2)–2.543(3)<br>(6 interactions)<br>avg: 2.412(2)	2.434(3)–2.709(5)<br>(7 interactions)<br>avg: 2.492(4)	2.386(6)–2.619(6)<br>(8 interactions)<br>avg: 2.502(6)	2.370(6)–2.842(6)<br>(8 interactions)<br>avg: 2.516(7)
Average of all Cu···O interactions between Cu_n rings	*2.501(2)<br>(11 interactions)*	*2.501(4)<br>(12 interactions)*	*2.509(6)<br>(13 interactions)*	*2.548(7)<br>(14 interactions)*
Cu···Cu distances in Cu_{12} or Cu_{14} ring	3.1976(10)–3.4104(11)<br>avg: 3.307(1)	3.1595(11)–3.3754(15)<br>avg: 3.261(2)	3.167(2)–3.358(2)<br>avg: 3.266(2)	3.172(2)–3.416(2)<br>avg: 3.291(2)
Cu···Cu distances in Cu_{10} or Cu_9 ring	3.0988(10)–3.3882(10)<br>avg: 3.276(1)	3.1633(14)–3.4112(12)<br>avg: 3.310(2)	3.1284(19)–3.379(2)<br>avg: 3.287(2)	3.139(2)–3.370(2)<br>avg: 3.294(2)
Cu···Cu distances in Cu_6, Cu_8 or Cu_9 ring	3.2838(12)–3.3506(10)<br>avg: 3.311(1)	3.3209(10)–3.3939(10)<br>avg: 3.364(2)	3.3265(17)–3.4038(19)<br>avg: 3.369(2)	3.265(2)–3.436(2)<br>avg: 3.338(2)
Average of all Cu···Cu distances in Cu_n rings	*3.298(1)*	*3.312(2)*	*3.307(2)*	*3.308(2)*
H-bonded O···O distances between Cu_{10} (or Cu_9) and Cu_{12} (or Cu_{14}) rings	2.723(3)–2.885(3)<br>(6 interactions)<br>avg: 2.813(3)	2.785(6)–2.882(5)<br>(6 interactions)<br>avg: 2.833(6)	2.750(8)–2.910(8)<br>(6 interactions)<br>avg: 2.818(8)	2.803(9)–2.886(10)<br>(6 interactions)<br>avg: 2.833(9)
H-bonded O···O distances between Cu_6 (or Cu_8 or Cu_9) and Cu_{12} (or Cu_{14}) rings	2.702(3)–2.829(4)<br>(6 interactions)<br>avg: 2.769(3)	2.768(5)–2.931(5)<br>(6 interactions)<br>avg: 2.850(5)	2.799(8)–2.978(8)<br>(6 interactions)<br>avg: 2.864(8)	2.789(9)–2.860(9)<br>(6 interactions)<br>avg: 2.830(9)
Average of all H-bonded O···O distances between Cu_n rings	*2.791(3)<br>(12 interactions)*	*2.842(6)<br>(12 interactions)*	*2.841(8)<br>(12 interactions)*	*2.832(9)<br>(12 interactions)*
H-bonded O···O distance range between Cu_n rings and MoO_4^{2-}	2.703(3)–3.173(3)<br>(14 interactions)<br>avg: 2.948(3)	2.716(6)–3.170(9)<br>(14 interactions)<br>avg: 2.904(6)	2.723(8)–3.134(8)<br>(15 interactions)<br>avg: 2.923(8)	2.768(12)–3.183(11)<br>(12 interactions)<br>avg: 2.95(1)

Table S8. Comparison of the dihedral, twist and fold angles (°) between pyrazolate moieties and adjacent Cu–O–Cu units in **1**–**6** (based on the major component in the case of disordered moieties).

	DIHEDRAL ANGLE	TWIST ANGLE	FOLD ANGLE
**$Cu_{31}SeO_4$ (1)** Cu_{14}-ring	2.9(3)–54.8(3) avg: 40.9(4)	0.3(3)–12.0(3) avg: 6.0(3)	2.1(4)–58.1(6) avg: 41.0(4)
**$Cu_{31}SeO_4$ (1)** Cu_9-ring	35.7(4)–79.0(3) avg: 52.0(4)	0.2(3)–8.7(3) avg: 4.4(3)	35.0(4)–79.1(3) avg: 51.7(4)
**$Cu_{31}SeO_4$(1)** Cu_8-ring	18.7(3)–63.4(3) avg: 46.4(4)	0.5(2)–9.6(3) avg: 3.2(3)	18.1(3)–64.4(3) avg: 46.3(4)
Avg. **$Cu_{31}SeO_4$ (1)**	*46.4(4)*	*4.5(3)*	*46.3(4)*
**$Cu_{32}SeO_4$ (2)** Cu_{14}-ring	33.9(5)–64.0(4) avg: 44.3(5)	0.0(4)–9.3(3) avg: 5.2(4)	35.5(5)–63.9(4) avg: 45.2(5)
**$Cu_{32}SeO_4$ (2)** Cu_9-ring	38.5(5)–66.8(4) avg: 50.5(5)	0.0(7)–6.1(5) avg: 2.6(5)	39.9(5)–66.8(4) avg: 51.2(5)
**$Cu_{32}SeO_4$ (2)** Cu_9-ring	25.6(7)–66.1(4) avg: 47.5(5)	0.0(6)–6.9(3) avg: 1.9(4)	25.6(7)–66.1(4) avg: 47.4(5)
Avg. **$Cu_{32}SeO_4$ (2)**	*47.4(5)*	*3.2(5)*	*47.9(5)*
**$Cu_{28}MoO_4$ (3)** Cu_{12}-ring	29.29(19)–53.50(15) avg: 43.6(2)	0.74(11)–8.44(12) avg: 4.7(2)	29.04(19)–53.41(15) avg: 43.3(2)
**$Cu_{28}MoO_4$ (3)** Cu_{10}-ring	8.94(19)–67.30(18) avg: 46.7(2)	0.00(15)–12.83(11) avg: 3.4(2)	8.79(19)–67.30(18) avg: 46.5(2)
**$Cu_{28}MoO_4$ (3)** Cu_6-ring	41.9(3)–59.65(16) avg: 51.8(2)	0.16(11)–5.32(11) avg: 2.2(2)	41.9(3)–59.65(16) avg: 51.8(2)
Avg. **$Cu_{28}MoO_4$ (3)**	*47.4(2)*	*3.4(2)*	*47.2(2)*
**$Cu_{31}MoO_4$ (4)** Cu_{14}-ring	1.2(3)–55.7(3) avg: 41.2(3)	0.4(3)–11.8(2) avg: 5.9(3)	0.1(4)–57.9(5) avg: 41.3(3)
**$Cu_{31}MoO_4$ (4)** Cu_9-ring	32.3(3)–79.8(3) avg: 50.6(3)	0.5(2)–9.5(3) avg: 4.3(2)	31.0(4)–79.9(3) avg: 50.3(3)
**$Cu_{31}MoO_4$ (4)** Cu_8-ring	16.2(3)–64.0(3) avg: 47.1(3)	0.24(19)–9.0(2) avg: 3.2(2)	15.7(3)–64.7(3) avg: 47.0(3)
Avg. **$Cu_{31}MoO_4$ (4)**	*46.3(3)*	*4.5(2)*	*46.2(3)*
**$Cu_{31}MoO_4$ (5)** Cu_{14}-ring	25.2(5)–54.4(5) avg: 43.4(5)	0.1(3)–13.7(3) avg: 5.2(4)	25.0(5)–71.1(10) avg: 47.0(5)
**$Cu_{31}MoO_4$ (5)** Cu_9-ring	36.0(4)–73.2(4) avg: 51.5(5)	0.0(3)–5.8(3) avg: 2.3(4)	35.6(4)–73.3(4) avg: 51.4(5)
**$Cu_{31}MoO_4$ (5)** Cu_8-ring	27.2(5)–66.4(4) avg: 46.8(5)	0.0(3)–7.0(3) avg: 3.4(3)	27.0(5)–66.4(4) avg: 50.9(5)
Avg. **$Cu_{31}MoO_4$ (5)**	*47.2(5)*	*3.6(4)*	*49.8(2)*
**$Cu_{32}MoO_4$ (5)** Cu_{14}-ring	2.3(5)–52.3(5) avg: 39.7(5)	1.8(5)–13.2(3) avg: 6.1(4)	1.4(6)–56.5(7) avg: 39.6(5)
**$Cu_{32}MoO_4$ (5)** Cu_{10}-ring	18.9(5)–72.0(5) avg: 47.7(5)	0.2(3)–9.0(4) avg: 3.8(3)	17.9(5)–72.0(5) avg: 48.1(5)
**$Cu_{32}MoO_4$ (5)** Cu_8-ring	24.4(5)–64.1(5) avg: 45.2(5)	0.5(4)–9.1(4) avg: 3.8(4)	22.7(5)–64.4(5) avg: 45.8(5)
Avg. **$Cu_{32}MoO_4$ (5)**	*44.2(5)*	*4.6(4)*	*44.5(5)*
**$Cu_{31}WO_4$ (6)** Cu_{14}-ring	1.1(6)–53.8(4) avg: 40.1(4)	0.3(4)–10.4(4) avg: 5.3(4)	0.1(7)–61.2(8) avg: 40.7(4)
**$Cu_{31}WO_4$ (6)** Cu_9-ring	33.5(6)–81.9(4) avg: 50.5(5)	0.6(3)–8.6(4) avg: 3.6(4)	32.5(6)–82.1(4) avg: 50.4(5)
**$Cu_{31}WO_4$ (6)** Cu_8-ring	18.4(4)–63.3(4) avg: 47.2(4)	0.5(3)–9.3(3) avg: 3.0(3)	18.0(4)–63.8(4) avg: 47.1(4)
Avg. **$Cu_{31}WO_4$ (6)**	*45.9(4)*	*4.0(4)*	*46.1(4)*

Table S9. Comparison of the dihedral, twist and fold angles (°) between adjacent pyrazolate moieties in **1–6** (based on the major component in the case of disordered moieties).

	DIHEDRAL ANGLE	TWIST ANGLE	FOLD ANGLE	CENTROID-CENTROID DISTANCE	
				D. A. > 35°	D. A. < 35°
**$Cu_{31}SeO_4$ (1)** Cu_{14}-ring	46.8(4)–66.6(3) avg: 55.5(4)	41.8(7)–66.8(4) avg: 55.3(4)	0.5(4)–37.1(8) avg: 16.2(4)	4.641(7)–4.916(5) avg. of 14: 4.806(5)	–
**$Cu_{31}SeO_4$ (1)** Cu_9-ring	11.3(3)–65.0(3) avg: 40.2(3)	6.8(3)–63.1(3) avg: 38.4(3)	0.1(4)–22.7(4) avg: 12.0(4)	4.837(4)–4.956(5) avg of 5: 4.897(5)	4.947(4)–5.178(5) avg of 4: 5.074(5)
**$Cu_{31}SeO_4$(1)** Cu_8-ring	26.7(3)–57.1(3) avg: 36.7(3)	17.5(3)–53.5(3) avg: 32.0(3)	1.5(4)–25.0(4) avg: 16.7(4)	4.880(6)–4.960(5) avg of 3: 4.916(5)	4.961(6)–5.144(4) avg of 5: 5.067(5)
Avg. **$Cu_{31}SeO_4$ (1)**	*44.1(3)*	*41.9(3)*	*15.0(4)*	*4.873(5)*	*5.071(5)*
**$Cu_{32}SeO_4$ (2)** Cu_{14}-ring	42.6(4)–70.2(4) avg: 56.8(4)	43.9(4)–70.1(4) avg: 57.2(4)	0.2(5)–32.1(5) avg: 14.8(5)	4.674(6)–4.914(6) avg. of 14: 4.825(6)	–
**$Cu_{32}SeO_4$ (2)** Cu_9-ring	20.5(7)–46.4(8) avg: 34.2(8)	7.6(8)–48.1(8) avg: 29.0(8)	4.8(10)–28.8(4) avg: 17.9(8)	4.796(13)–5.021(6) avg of 5: 4.947(6)	4.895(13)–5.100(6) avg of 4: 5.009(6)
**$Cu_{32}SeO_4$ (2)** Cu_9-ring	31.7(5)–49.3(6) avg: 37.9(5)	19.0(5)–41.9(5) avg: 31.9(5)	1.0(5)–35.0(5) avg: 17.7(5)	4.835(9)–4.940(6) avg of 6: 4.896(6)	4.991(5)–5.021(7) avg of 3: 5.007(6)
Avg. **$Cu_{32}SeO_4$ (2)**	*43.0(5)*	*39.4(5)*	*16.8(5)*	*4.889(6)*	*5.008(6)*
**$Cu_{28}MoO_4$ (3)** Cu_{12}-ring	48.55(15)–67.86(15) avg: 57.0(2)	50.03(15)–64.96(16) avg: 57.0(2)	2.62(17)–33.77(18) avg: 16.2(2)	4.734(3)–4.902(3) avg. of 12: 4.824(3)	–
**$Cu_{28}MoO_4$ (3)** Cu_{10}-ring	11.42(15)–58.05(16) avg: 38.4(2)	9.06(15)–54.57(17) avg: 38.3(2)	0.50(17)–25.40(18) avg: 6.9(2)	4.839(3)–4.914(3) avg of 7: 4.884(3)	4.942(3)–5.133(3) avg of 3: 5.010(3)
**$Cu_{28}MoO_4$ (3)** Cu_6-ring	27.88(14)–45.3(2) avg: 35.8(2)	1.88(14)–21.7(2) avg: 14.7(2)	24.0(3)–42.0(3) avg: 32.2(3)	4.876(3)–4.988(2) avg of 3: 4.933(3)	4.996(3)–5.022(3) avg of 3: 5.011(3)
Avg. **$Cu_{28}MoO_4$ (3)**	*43.7(2)*	*36.7(2)*	*18.4(2)*	*4.880(3)*	*5.011(3)*
**$Cu_{31}MoO_4$ (4)** Cu_{14}-ring	46.1(3)–64.4(3) avg: 55.4(3)	43.5(11)–65.6(3) avg: 55.5(3)	0.7(3)–37.0(6) avg: 15.5(3)	4.691(7)–4.925(4) avg. of 14: 4.818(4)	–
**$Cu_{31}MoO_4$ (4)** Cu_9-ring	12.8(3)–67.0(3) avg: 40.8(3)	7.9(3)–65.2(3) avg: 38.9(3)	0.1(3)–22.9(3) avg: 12.4(3)	4.846(3)–4.976(5) avg of 6: 4.903(5)	5.066(4)–5.157(5) avg of 3: 5.116(5)
**$Cu_{31}MoO_4$ (4)** Cu_8-ring	24.8(3)–57.9(3) avg: 36.4(3)	15.6(3)–55.1(3) avg: 32.1(3)	4.4(3)–23.2(3) avg: 16.2(3)	4.888(5)–4.973(4) avg of 3: 4.920(5)	4.966(4)–5.163(4) avg of 5: 5.078(4)
Avg. **$Cu_{31}MoO_4$ (4)**	*44.2(3)*	*42.2(3)*	*14.7(3)*	*4.880(5)*	*5.097(5)*
**$Cu_{31}MoO_4$ (5)** Cu_{14}-ring	42.7(5)–66.8(6) avg: 56.3(5)	25.8(5)–66.7(5) avg: 55.0(5)	12.1(5)–37.2(11) avg: 29.1(5)	4.660(6)–4.914(6) avg. of 14: 4.825(6)	–
**$Cu_{31}MoO_4$ (5)** Cu_9-ring	15.4(4)–59.9(4) avg: 43.4(4)	3.0(4)–60.8(4) avg: 38.6(4)	3.3(5)–43.3(8) avg: 23.6(5)	4.855(6)–5.066(6) avg of 7: 4.907(6)	5.115(6), 5.182(6) avg of 2: 5.149(6)
**$Cu_{31}MoO_4$ (5)** Cu_8-ring	32.8(5)–57.2(4) avg: 42.0(5)	26.7(5)–56.7(4) avg: 38.4(5)	15.6(4)–42.0(12) avg: 25.0(5)	4.905(6)–5.076(7) avg of 6: 4.988(6)	5.049(6), 5.141(8) avg of 2: 5.095(6)
Avg. **$Cu_{31}MoO_4$ (5)**	*47.2(5)*	*44.0(5)*	*25.9(5)*	*4.907(6)*	*5.122(6)*
**$Cu_{32}MoO_4$ (5)** Cu_{14}-ring	40.5(13)–64.4(5) avg: 53.3(5)	37.1(12)–63.0(5) avg: 53.2(5)	0.1(6)–48.1(11) avg: 17.7(6)	4.674(15)–4.912(6) avg. of 14: 4.801(6)	–
**$Cu_{32}MoO_4$ (5)** Cu_{10}-ring	24.2(4)–61.0(4) avg: 42.3(4)	23.2(4)–59.4(4) avg: 42.2(4)	1.6(4)–33.4(9) avg: 13.3(4)	4.858(6)–4.957(6) avg of 8: 4.887(6)	5.110(6), 5.124(6) avg of 2: 5.117(6)
**$Cu_{32}MoO_4$ (5)** Cu_8-ring	22.0(5)–63.0(5) avg: 39.7(5)	2.7(5)–60.6(5) avg: 30.0(5)	3.4(5)–45.0(10) avg: 23.6(5)	4.800(7)–4.960(7) avg of 4: 4.865(7)	4.918(8)–5.073(7) avg of 4: 5.012(7)
Avg. **$Cu_{32}MoO_4$ (5)**	*45.1(5)*	*41.8(5)*	*18.2(5)*	*4.851(7)*	*5.065(7)*
**$Cu_{31}WO_4$ (6)** Cu_{14}-ring	45.6(8)–64.0(4) avg: 54.9(5)	42.2(8)–65.7(4) avg: 54.9(5)	0.8(5)–36.6(9) avg: 14.4(5)	4.595(9)–4.922(6) avg. of 14: 4.809(6)	–
**$Cu_{31}WO_4$ (6)** Cu_9-ring	10.9(4)–68.5(5) avg: 40.6(5)	5.7(4)–67.4(5) avg: 38.5(5)	2.0(5)–23.8(5) avg: 12.3(5)	4.744(6)–5.068(8) avg of 6: 4.895(6)	5.061(6)–5.171(6) avg of 3: 5.128(6)
**$Cu_{31}WO_4$ (6)** Cu_8-ring	24.4(4)–58.3(3) avg: 36.9(4)	14.9(4)–55.5(4) avg: 32.3(4)	4.2(4)–23.8(4) avg: 16.4(4)	4.881(6)–4.984(7) avg of 4: 4.929(6)	5.048(5)–5.161(5) avg of 4: 5.102(5)
Avg. **$Cu_{31}WO_4$ (6)**	*44.1(5)*	*41.9(5)*	*14.4(5)*	*4.878(6)*	*5.115(6)*

Table S10. Copper coordination geometry indexes, $\tau_4 = (360 - \beta - \alpha)/141$ and $\tau_5 = (\beta - \alpha)/60$ in different Cu_x rings in **1–6** (where β and α are the two largest angles in the four- or five-coordinate species.

	τ_4	τ_5
**$Cu_{31}SeO_4$ (1)** Cu_{14}-ring	0.08–0.20<br>avg: 0.15	0.00–0.11<br>avg: 0.04
**$Cu_{31}SeO_4$ (1)** Cu_9-ring	0.03–0.13<br>avg: 0.08	0.00–0.09<br>avg: 0.04
**$Cu_{31}SeO_4$(1)** Cu_8-ring	0.08–0.12<br>avg: 0.11	0.01–0.18<br>avg: 0.10
Avg. **$Cu_{31}SeO_4$ (1)**	*0.11*	*0.06*
**$Cu_{32}SeO_4$ (2)** Cu_{14}-ring	0.05–0.18<br>avg: 0.12	0.00–0.10<br>avg: 0.04
**$Cu_{32}SeO_4$ (2)** Cu_9-ring	0.03–0.13<br>avg: 0.10	0.01–0.14<br>avg: 0.08
**$Cu_{32}SeO_4$ (2)** Cu_9-ring	0.03–0.17<br>avg: 0.11	0.01–0.30<br>avg: 0.10
Avg. **$Cu_{32}SeO_4$ (2)**	*0.11*	*0.07*
**$Cu_{28}MoO_4$ (3)** Cu_{12}-ring	0.08–0.16<br>avg: 0.12	0.00–0.12<br>avg: 0.06
**$Cu_{28}MoO_4$ (3)** Cu_{10}-ring	0.05–0.33<br>avg: 0.12	0.01–0.32<br>avg: 0.09
**$Cu_{28}MoO_4$ (3)** Cu_6-ring	0.12–0.15<br>avg: 0.13	0.03–0.16<br>avg: 0.11
Avg. **$Cu_{28}MoO_4$ (3)**	*0.12*	*0.09*
**$Cu_{31}MoO_4$ (4)** Cu_{14}-ring	0.07–0.20<br>avg: 0.15	0.00–0.13<br>avg: 0.04
**$Cu_{31}MoO_4$ (4)** Cu_9-ring	0.05–0.13<br>avg: 0.08	0.00–0.07<br>avg: 0.03
**$Cu_{31}MoO_4$ (4)** Cu_8-ring	0.08–0.12<br>avg: 0.11	0.01–0.21<br>avg: 0.11
Avg. **$Cu_{31}MoO_4$ (4)**	*0.11*	*0.06*
**$Cu_{31}MoO_4$ (5)** Cu_{14}-ring	0.08–0.23<br>avg: 0.14	0.01–0.08<br>avg: 0.04
**$Cu_{31}MoO_4$ (5)** Cu_9-ring	0.10–0.23<br>avg: 0.14	0.00–0.23<br>avg: 0.12
**$Cu_{31}MoO_4$ (5)** Cu_8-ring	0.06–0.16<br>avg: 0.10	0.01–0.12<br>avg: 0.03
Avg. **$Cu_{31}MoO_4$ (5)**	*0.13*	*0.06*
**$Cu_{32}MoO_4$ (5)** Cu_{14}-ring	0.08–0.20<br>avg: 0.14	0.00–0.12<br>avg: 0.05
**$Cu_{32}MoO_4$ (5)** Cu_{10}-ring	0.06–0.12<br>avg: 0.09	0.01–0.10<br>avg: 0.05
**$Cu_{32}MoO_4$ (5)** Cu_8-ring	0.09–0.14<br>avg: 0.12	0.02–0.17<br>avg: 0.09
Avg. **$Cu_{32}MoO_4$ (5)**	*0.12*	*0.06*
**$Cu_{31}WO_4$ (6)** Cu_{14}-ring	0.07–0.19<br>avg: 0.14	0.00–0.18<br>avg: 0.04
**$Cu_{31}WO_4$ (6)** Cu_9-ring	0.05–0.13<br>avg: 0.09	0.02–0.14<br>avg: 0.06
**$Cu_{31}WO_4$ (6)** Cu_8-ring	0.09–0.12<br>avg: 0.11	0.00–0.21<br>avg: 0.10
Avg. **$Cu_{31}WO_4$ (6)**	*0.11*	*0.07*

Table S11. Comparison of the deviations (Å) of Cu atoms in different Cu_x rings from the Cu_x mean-plane in **1–6**.

	**Deviation from Cu_x mean-plane**
**$Cu_{31}SeO_4$ (1)** Cu_{14}-ring	0.076–1.476<br>avg: 0.697
**$Cu_{31}SeO_4$ (1)** Cu_9-ring	0.055–0.754<br>avg: 0.411
**$Cu_{31}SeO_4$(1)** Cu_8-ring	0.261–0.505<br>avg: 0.384
Avg. **$Cu_{31}SeO_4$ (1)**	*0.497*
**$Cu_{32}SeO_4$ (2)** Cu_{14}-ring	0.166–1.949<br>avg: 1.108
**$Cu_{32}SeO_4$ (2)** Cu_9-ring	0.190–1.120<br>avg: 0.822
**$Cu_{32}SeO_4$ (2)** Cu_9-ring	0.015–1.156<br>avg: 0.800
Avg. **$Cu_{32}SeO_4$ (2)**	*0.910*
**$Cu_{28}MoO_4$ (3)** Cu_{12}-ring	0.018–0.567<br>avg: 0.324
**$Cu_{28}MoO_4$ (3)** Cu_{10}-ring	0.027–0.774<br>avg: 0.279
**$Cu_{28}MoO_4$ (3)** Cu_6-ring	0.036–0.174<br>avg: 0.100
Avg. **$Cu_{28}MoO_4$ (3)**	*0.234*
**$Cu_{31}MoO_4$ (4)** Cu_{14}-ring	0.087–1.437<br>avg: 0.685
**$Cu_{31}MoO_4$ (4)** Cu_9-ring	0.083–0.718<br>avg: 0.405
**$Cu_{31}MoO_4$ (4)** Cu_8-ring	0.263–0.485<br>avg: 0.375
Avg. **$Cu_{31}MoO_4$ (4)**	*0.488*
**$Cu_{31}MoO_4$ (5)** Cu_{14}-ring	0.060–1.612<br>avg: 0.709
**$Cu_{31}MoO_4$ (5)** Cu_9-ring	0.045–0.714<br>avg: 0.407
**$Cu_{31}MoO_4$ (5)** Cu_8-ring	0.305–0.448<br>avg: 0.372
Avg. **$Cu_{31}MoO_4$ (5)**	*0.496*
**$Cu_{32}MoO_4$ (5)** Cu_{14}-ring	0.178–1.536<br>avg: 0.945
**$Cu_{32}MoO_4$ (5)** Cu_{10}-ring	0.160–0.992<br>avg: 0.675
**$Cu_{32}MoO_4$ (5)** Cu_8-ring	0.246–0.650<br>avg: 0.450
Avg. **$Cu_{32}MoO_4$ (5)**	*0.690*
**$Cu_{31}WO_4$ (6)** Cu_{14}-ring	0.100–1.489<br>avg: 0.695
**$Cu_{31}WO_4$ (6)** Cu_9-ring	0.070–0.662<br>avg: 0.382
**$Cu_{31}WO_4$ (6)** Cu_8-ring	0.272–0.483<br>avg: 0.380
Avg. **$Cu_{31}WO_4$ (6)**	*0.486*

Table S12. Selected bond lengths for **1** (Cu1–Cu9: Cu_9-ring; Cu10–Cu23: Cu_{14}-ring; Cu24–Cu31: Cu_8-ring). The SeO_4^{2-} anion is disordered over three positions in a 0.49/0.35/0.16 ratio.

Se1–O34 1.603(12)	Cu6–N11 2.009(5)	Cu15–O15 1.928(4)	Cu23–N45 1.945(6)
Se1–O32 1.626(11)	Cu6–O17 2.396(4)	Cu15–O14 1.931(5)	Cu23–N44 1.953(5)
Se1–O35 1.639(10)	Cu7–O7 1.903(5)	Cu15–N28 1.956(5)	Cu24–O31 1.917(4)
Se1–O33 1.651(11)	Cu7–O6 1.921(4)	Cu15–N29 1.962(6)	Cu24–O24 1.948(4)
Se1B–O34B 1.599(12)	Cu7–N13 1.955(6)	Cu16–O16 1.924(4)	Cu24–N47 1.997(5)
Se1B–O35B 1.602(13)	Cu7–N12 1.977(5)	Cu16–O15 1.934(4)	Cu24–N62 1.997(5)
Se1B–O33B 1.602(13)	Cu8–O8 1.913(4)	Cu16–N30 1.952(6)	Cu25–O25 1.904(4)
Se1B–O32B 1.655(14)	Cu8–O7 1.917(4)	Cu16–N31 1.964(6)	Cu25–O24 1.936(4)
Se1C–O34C 1.597(19)	Cu8–N14 1.963(6)	Cu17–O17 1.917(4)	Cu25–N48 1.973(5)
Se1C–O35C 1.621(18)	Cu8–N15 1.980(5)	Cu17–O16 1.930(4)	Cu25–N49 1.992(6)
Se1C–O32C 1.626(19)	Cu9–O9 1.941(4)	Cu17–N32 1.966(6)	Cu26–O25 1.916(4)
Se1C–O33C 1.64(2)	Cu9–O8 1.944(4)	Cu17–N33 1.972(6)	Cu26–O26 1.930(4)
Cu1–O1 1.917(5)	Cu9–N17 2.008(5)	Cu18–O18 1.920(4)	Cu26–N50 1.971(6)
Cu1–O9 1.932(4)	Cu9–N16 2.024(5)	Cu18–O17 1.928(4)	Cu26–N51 2.015(5)
Cu1–N1 1.974(5)	Cu10–O10 1.925(5)	Cu18–N34 1.950(6)	Cu27–O26 1.928(4)
Cu1–N18 1.985(6)	Cu10–O23 1.928(4)	Cu18–N35 1.965(5)	Cu27–O27 1.949(4)
Cu2–O1 1.902(5)	Cu10–N46 1.963(6)	Cu19–O18 1.912(4)	Cu27–N53 1.982(5)
Cu2–O2 1.941(4)	Cu10–N19 1.964(5)	Cu19–O19 1.928(4)	Cu27–N52 2.005(6)
Cu2–N2 1.956(5)	Cu11–O11 1.915(5)	Cu19–N37B 1.96(3)	Cu28–O28 1.929(4)
Cu2–N3 1.976(5)	Cu11–O10 1.917(4)	Cu19–N37 1.966(7)	Cu28–O27 1.944(4)
Cu3–O3 1.939(4)	Cu11–N21 1.95(2)	Cu19–N36 1.969(5)	Cu28–N55 1.998(5)
Cu3–O2 1.960(4)	Cu11–N20 1.952(6)	Cu20–O19 1.911(4)	Cu28–N54 2.000(5)
Cu3–N5 1.984(5)	Cu11–N21B 1.961(8)	Cu20–O20 1.911(4)	Cu29–O29 1.926(4)
Cu3–N4 1.985(5)	Cu12–O12 1.903(4)	Cu20–N39 1.960(6)	Cu29–O28 1.942(4)
Cu3–O13 2.393(4)	Cu12–O11 1.934(4)	Cu20–N38 1.968(7)	Cu29–N57 1.972(5)
Cu4–O3 1.914(4)	Cu12–N22 1.94(2)	Cu20–N38B 2.00(3)	Cu29–N56 2.005(5)
Cu4–O4 1.917(4)	Cu12–N22B 1.958(8)	Cu21–O21 1.923(4)	Cu29–O18 2.437(4)
Cu4–N7 1.956(5)	Cu12–N23 1.965(6)	Cu21–O20 1.927(4)	Cu30–O29 1.896(4)
Cu4–N6 1.976(5)	Cu13–O13 1.921(5)	Cu21–N40 1.959(6)	Cu30–O30 1.931(4)
Cu5–O5 1.919(4)	Cu13–O12 1.923(4)	Cu21–N41 1.973(5)	Cu30–N59 1.963(5)
Cu5–O4 1.922(4)	Cu13–N25 1.959(5)	Cu22–O22 1.912(4)	Cu30–N58 1.972(5)
Cu5–N9 1.983(5)	Cu13–N24 1.969(6)	Cu22–O21 1.929(4)	Cu31–O31 1.934(4)
Cu5–N8 1.984(5)	Cu14–O13 1.919(4)	Cu22–N42 1.953(5)	Cu31–O30 1.953(4)
Cu6–O5 1.940(4)	Cu14–O14 1.928(4)	Cu22–N43 1.969(5)	Cu31–N60 1.993(5)
Cu6–O6 1.952(4)	Cu14–N27 1.957(6)	Cu23–O22 1.908(4)	Cu31–N61 2.018(5)
Cu6–N10 1.989(5)	Cu14–N26 1.967(6)	Cu23–O23 1.918(4)	Cu31–O21 2.418(4)

Table S13. Selected bond lengths for **2** (Cu1–Cu9: Cu_9-ring; Cu10–Cu23: Cu_{14}-ring; Cu24–Cu32: Cu_9-ring). SeO_4^{2-} is disordered over two positions in a 0.66/0.34 ratio.

Se1–O36 1.544(11)	Cu7–O6 1.915(8)	Cu16–O15 1.939(6)	Cu25–O24 1.949(6)
Se1–O34 1.561(11)	Cu7–O7 1.933(6)	Cu16–O16 1.940(6)	Cu25–N48 1.981(7)
Se1–O33 1.564(12)	Cu7–N13 1.963(8)	Cu16–N31 1.951(8)	Cu25–N49 2.006(17)
Se1–O35 1.731(12)	Cu7–N12 1.990(12)	Cu16–N30 1.974(7)	Cu25–N49B 2.03(3)
Se1B–O36B 1.556(16)	Cu7–N12B 1.996(17)	Cu17–O17 1.934(6)	Cu26–O26 1.934(7)
Se1B–O33B 1.583(17)	Cu8–O8 1.941(6)	Cu17–O16 1.946(6)	Cu26–O25 1.950(7)
Se1B–O34B 1.588(16)	Cu8–O7 1.941(6)	Cu17–N32 1.960(8)	Cu26–N51B 1.96(3)
Se1B–O35B 1.621(17)	Cu8–N14 1.981(8)	Cu17–N33 1.969(8)	Cu26–N50 1.962(17)
Cu1–O9 1.911(7)	Cu8–N15 1.981(7)	Cu18–O17 1.928(6)	Cu26–N51 1.995(17)
Cu1–O1 1.933(8)	Cu8–O21 2.378(7)	Cu18–O18 1.929(6)	Cu26–N50B 2.05(3)
Cu1–N1B 1.962(17)	Cu9–O9 1.948(6)	Cu18–N34 1.966(8)	Cu26–O14 2.432(7)
Cu1–N18 1.967(8)	Cu9–O8 1.949(6)	Cu18–N35 1.969(7)	Cu27–O27 1.904(6)
Cu1–N1 1.997(13)	Cu9–N16 1.980(7)	Cu19–O18 1.927(6)	Cu27–O26 1.915(7)
Cu2–O2 1.905(7)	Cu9–N17 1.996(8)	Cu19–O19 1.940(6)	Cu27–N52 1.940(15)
Cu2–O1 1.919(8)	Cu9–O23 2.379(6)	Cu19–N36 1.971(8)	Cu27–N53 1.984(9)
Cu2–N3 1.952(10)	Cu10–O23 1.920(6)	Cu19–N37 1.972(8)	Cu27–N52B 2.00(3)
Cu2–N2B 1.979(17)	Cu10–O10 1.929(6)	Cu20–O20 1.925(6)	Cu28–O28 1.919(6)
Cu2–N2 1.986(14)	Cu10–N19 1.965(7)	Cu20–O19 1.933(6)	Cu28–O27 1.922(6)
Cu3–O3 1.932(6)	Cu10–N46 1.972(7)	Cu20–N38 1.956(8)	Cu28–N55 1.974(8)
Cu3–O2 1.939(6)	Cu11–O10 1.927(6)	Cu20–N39 1.978(8)	Cu28–N54 1.977(9)
Cu3–N4 1.992(9)	Cu11–O11 1.944(6)	Cu21–O20 1.922(6)	Cu29–O29 1.942(6)
Cu3–N5B 2.004(16)	Cu11–N21 1.967(8)	Cu21–O21 1.929(6)	Cu29–O28 1.942(6)
Cu3–N5 2.014(13)	Cu11–N20 1.968(8)	Cu21–N41 1.958(8)	Cu29–N56 1.961(8)
Cu3–O13 2.397(7)	Cu12–O12 1.927(6)	Cu21–N40 1.958(8)	Cu29–N57 1.974(7)
Cu4–O3 1.925(6)	Cu12–O11 1.928(6)	Cu22–O22 1.943(6)	Cu29–O18 2.411(7)
Cu4–O4 1.953(6)	Cu12–N22 1.941(9)	Cu22–O21 1.945(6)	Cu30–O29 1.931(6)
Cu4–N7 1.974(7)	Cu12–N23 1.973(7)	Cu22–N42 1.964(7)	Cu30–O30 1.954(6)
Cu4–N6 1.991(12)	Cu13–O13 1.925(7)	Cu22–N43 1.983(7)	Cu30–N59 1.979(8)
Cu4–N6B 2.005(15)	Cu13–O12 1.929(6)	Cu23–O23 1.925(6)	Cu30–N58 1.993(8)
Cu5–O5 1.931(6)	Cu13–N24 1.947(8)	Cu23–O22 1.931(6)	Cu30–O20 2.362(7)
Cu5–O4 1.949(7)	Cu13–N25 1.986(8)	Cu23–N45 1.947(7)	Cu31–O31 1.923(6)
Cu5–N8 1.990(7)	Cu14–O14 1.918(6)	Cu23–N44 1.964(7)	Cu31–O30 1.924(6)
Cu5–N9 2.021(8)	Cu14–O13 1.918(7)	Cu24–O32 1.933(6)	Cu31–N61 1.952(7)
Cu5–O17 2.366(6)	Cu14–N27 1.943(9)	Cu24–O24 1.970(6)	Cu31–N60 1.972(7)
Cu6–O6 1.895(7)	Cu14–N26 1.960(9)	Cu24–N47 1.985(6)	Cu32–O31 1.908(6)
Cu6–O5 1.912(7)	Cu15–O14 1.930(6)	Cu24–N64 1.993(7)	Cu32–O32 1.927(6)
Cu6–N10 1.959(8)	Cu15–N29 1.946(8)	Cu24–O10 2.402(6)	Cu32–N63 1.977(7)
Cu6–N11 1.963(12)	Cu15–O15 1.956(6)	Cu25–O25 1.944(6)	Cu32–N62 1.980(7)
Cu6–N11B 2.007(16)	Cu15–N28 1.985(8)		

Table S14. Selected bond lengths for **3** (Cu1–Cu6: Cu$_6$-ring; Cu7–Cu18: Cu$_{12}$-ring; Cu19–Cu28: Cu$_{10}$-ring).

Mo1–O29 1.751(2)	Cu6–N11 2.007(3)	Cu14–O14 1.950(2)	Cu22–O22 1.913(2)
Mo1–O31 1.752(2)	Cu6–O16 2.376(2)	Cu14–N27 1.970(3)	Cu22–O21 1.915(2)
Mo1–O30 1.767(3)	Cu7–O7 1.933(2)	Cu14–N26 1.975(3)	Cu22–N42 1.972(3)
Mo1–O32 1.768(2)	Cu7–O18 1.936(2)	Cu15–O15 1.937(2)	Cu22–N43 1.975(3)
Cu1–O6 1.921(2)	Cu7–N13 1.964(3)	Cu15–O14 1.943(2)	Cu23–O23 1.940(2)
Cu1–O1 1.947(2)	Cu7–N36 1.981(3)	Cu15–N29 1.965(3)	Cu23–O22 1.946(2)
Cu1–N1 1.966(3)	Cu8–O7 1.938(2)	Cu15–N28 1.977(3)	Cu23–N44 2.014(3)
Cu1–N12 1.982(3)	Cu8–O8 1.942(2)	Cu16–O16 1.922(2)	Cu23–N45 2.019(3)
Cu2–O1 1.949(2)	Cu8–N15 1.969(3)	Cu16–O15 1.943(2)	Cu23–O11 2.329(2)
Cu2–O2 1.954(2)	Cu8–N14 1.972(3)	Cu16–N30 1.951(3)	Cu24–O24 1.896(2)
Cu2–N3 1.992(3)	Cu9–O8 1.938(2)	Cu16–N31 1.988(3)	Cu24–O23 1.909(2)
Cu2–N2 2.007(3)	Cu9–O9 1.940(2)	Cu17–O17 1.930(2)	Cu24–N46 1.961(3)
Cu2–O8 2.349(2)	Cu9–N16 1.973(3)	Cu17–O16 1.934(2)	Cu24–N47 1.974(3)
Cu3–O3 1.933(2)	Cu9–N17 1.973(3)	Cu17–N33 1.968(3)	Cu25–O25 1.919(2)
Cu3–O2 1.946(2)	Cu10–O10 1.9194(19)	Cu17–N32 1.973(3)	Cu25–O24 1.925(2)
Cu3–N4 1.980(2)	Cu10–O9 1.933(2)	Cu18–O17 1.923(2)	Cu25–N48 1.960(3)
Cu3–N5 1.991(3)	Cu10–N18 1.950(3)	Cu18–O18 1.938(2)	Cu25–N49 1.978(3)
Cu3–O10 2.417(2)	Cu10–N19 1.974(3)	Cu18–N35 1.952(3)	Cu26–O26 1.915(2)
Cu4–O3 1.9237(19)	Cu11–O11 1.926(2)	Cu18–N34 1.968(3)	Cu26–O25 1.927(2)
Cu4–O4 1.956(2)	Cu11–O10 1.938(2)	Cu19–O19 1.900(2)	Cu26–N50 1.968(3)
Cu4–N7 1.978(2)	Cu11–N20 1.956(3)	Cu19–O28 1.907(2)	Cu26–N51 1.973(3)
Cu4–N6 2.006(3)	Cu11–N21 1.972(2)	Cu19–N56 1.964(3)	Cu27–O26 1.906(2)
Cu4–O12 2.435(2)	Cu12–O11 1.922(2)	Cu19–N37 1.983(3)	Cu27–O27 1.936(2)
Cu5–O4 1.942(2)	Cu12–O12 1.930(2)	Cu20–O19 1.919(2)	Cu27–N53 1.961(3)
Cu5–O5 1.945(2)	Cu12–N22 1.954(3)	Cu20–O20 1.934(2)	Cu27–N52 1.976(3)
Cu5–N9 1.987(2)	Cu12–N23 1.965(3)	Cu20–N38 1.979(3)	Cu28–O28 1.928(3)
Cu5–N8 2.000(2)	Cu13–O12 1.922(2)	Cu20–N39 1.999(3)	Cu28–O27 1.953(2)
Cu5–O14 2.350(2)	Cu13–O13 1.927(2)	Cu21–O20 1.922(2)	Cu28–N55 1.972(3)
Cu6–O6 1.926(2)	Cu13–N25 1.947(3)	Cu21–O21 1.933(2)	Cu28–N54 1.991(3)
Cu6–O5 1.957(2)	Cu13–N24 1.979(3)	Cu21–N40 1.969(3)	Cu28–O17 2.395(3)
Cu6–N10 1.985(2)	Cu14–O13 1.938(2)	Cu21–N41 1.973(3)	

Table S15. Selected bond lengths for **4** (Cu1–Cu8: Cu$_8$-ring; Cu9–Cu22: Cu$_{14}$-ring; Cu23–Cu31: Cu$_9$-ring).

Mo1 O3 1.725(5)	Cu8–O8_1 1.891(4)	Cu16–O15_1 1.917(4)	Cu24–O23_1 1.911(4)
Mo1 O2 1.740(4)	Cu8–O7_1 1.930(3)	Cu16–N30_1 1.940(18)	Cu24–O24_1 1.925(4)
Mo1 O4 1.744(4)	Cu8–N14_1 1.974(4)	Cu16–N31_1 1.960(5)	Cu24–N47_1 1.982(5)
Mo1 O1 1.760(4)	Cu8–N15_1 1.981(5)	Cu16–N30B_1 2.012(16)	Cu24–N46_1 1.987(5)
Cu1–O8_1 1.933(4)	Cu9–O22_1 1.921(4)	Cu17–O17_1 1.925(3)	Cu25–O25_1 1.912(3)
Cu1–O1_1 1.940(4)	Cu9–O9_1 1.935(3)	Cu17–O16_1 1.929(4)	Cu25–O24_1 1.913(4)
Cu1–N16_1 1.982(5)	Cu9–N17_1 1.960(5)	Cu17–N33_1 1.961(5)	Cu25–N48_1 1.959(4)
Cu1–N1_1 2.014(5)	Cu9–N44_1 1.964(5)	Cu17–N32_1 1.965(5)	Cu25–N49_1 1.979(5)
Cu2–O1_1 1.916(3)	Cu10–O9_1 1.916(4)	Cu18–O18_1 1.908(3)	Cu26–O25_1 1.941(4)
Cu2–O2_1 1.950(3)	Cu10–O10_1 1.939(3)	Cu18–O17_1 1.922(3)	Cu26–O26_1 1.959(4)
Cu2–N3_1 1.995(4)	Cu10–N19_1 1.957(5)	Cu18–N34_1 1.952(5)	Cu26–N50_1 1.972(5)
Cu2–N2_1 1.999(4)	Cu10–N18_1 1.969(5)	Cu18–N35_1 1.964(5)	Cu26–N51_1 1.978(4)
Cu3–O3_1 1.917(3)	Cu11–O10_1 1.924(4)	Cu19–O18_1 1.908(3)	Cu26–O13_1 2.386(3)
Cu3–O2_1 1.953(3)	Cu11–O11_1 1.932(4)	Cu19–O19_1 1.927(3)	Cu27–O27_1 1.910(4)
Cu3–N4_1 1.981(4)	Cu11–N21_1 1.946(5)	Cu19–N36_1 1.957(4)	Cu27–O26_1 1.925(4)
Cu3–N5_1 2.011(4)	Cu11–N20_1 1.968(5)	Cu19–N37_1 1.959(4)	Cu27–N53_1 1.947(5)
Cu4–O4_1 1.928(4)	Cu12–O11_1 1.929(4)	Cu20–O19_1 1.921(3)	Cu27–N52_1 1.965(5)
Cu4–O3_1 1.930(3)	Cu12–O12_1 1.933(4)	Cu20–O20_1 1.929(4)	Cu28–O28_1 1.927(3)
Cu4–N7_1 1.977(5)	Cu12–N23_1 1.963(5)	Cu20–N39_1 1.954(4)	Cu28–O27_1 1.933(4)
Cu4–N6_1 2.012(4)	Cu12–N22_1 1.975(5)	Cu20–N38_1 1.963(5)	Cu28–N54_1 1.979(5)
Cu4–O14_1 2.440(4)	Cu13– O13_1 1.913(3)	Cu21–O20_1 1.905(4)	Cu28–N55_1 1.990(5)
Cu5–O4_1 1.898(4)	Cu13–O12_1 1.934(4)	Cu21–O21_1 1.907(4)	Cu29–O28_1 1.940(3)
Cu5–O5_1 1.928(3)	Cu13–N24_1 1.952(5)	Cu21–N41_1 1.967(5)	Cu29–O29_1 1.940(3)
Cu5–N8_1 1.983(5)	Cu13–N25_1 1.970(4)	Cu21–N40_1 1.972(5)	Cu29–N57_1 2.003(4)
Cu5–N9_1 1.984(4)	Cu14–O14_1 1.917(3)	Cu22–O22_1 1.907(4)	Cu29–N56_1 2.009(5)
Cu6–O6_1 1.912(3)	Cu14–O13_1 1.926(3)	Cu22–O21_1 1.929(4)	Cu30–O29_1 1.925(3)
Cu6–O5_1 1.962(3)	Cu14–N26_1 1.961(5)	Cu22–N42_1 1.952(5)	Cu30–O30_1 1.929(4)
Cu6–N10_1 1.994(4)	Cu14–N27_1 1.968(5)	Cu22–N43_1 1.974(5)	Cu30–N58_1 1.978(4)
Cu6–N11_1 2.005(4)	Cu15–O14_1 1.904(4)	Cu23–O23_1 1.942(4)	Cu30–N59_1 1.980(5)
Cu7–O6_1 1.924(3)	Cu15–O15_1 1.923(4)	Cu23–O31_1 1.947(4)	Cu31–O30_1 1.913(4)
Cu7–O7_1 1.970(4)	Cu15–N29_1 1.940(17)	Cu23–N45_1 1.989(5)	Cu31–O31_1 1.916(4)
Cu7–N13_1 1.983(4)	Cu15–N29B_1 1.980(18)	Cu23–N62_1 2.004(5)	Cu31–N60_1 1.948(5)
Cu7–N12_1 2.025(4)	Cu15–N28_1 1.987(5)	Cu23–O9_1 2.398(4)	Cu31–N61_1 1.983(5)
Cu7–O19_1 2.434(3)	Cu16–O16_1 1.913(4)		

Table S16. Selected bond lengths for nanojar unit 1 (Cu_{31}) in **5** (Cu1–Cu9: Cu_9-ring; Cu10–Cu23: Cu_{14}-ring; Cu24–Cu31: Cu_8-ring).

Mo1–O34 1.747(5)	Cu7–N12 1.978(8)	Cu16–O16 1.910(6)	Cu24–O24 1.940(6)
Mo1–O32 1.751(5)	Cu8–O7 1.928(6)	Cu16–O15 1.930(6)	Cu24–N62 1.977(7)
Mo1–O33 1.754(5)	Cu8–O8 1.940(6)	Cu16–N31 1.953(8)	Cu24–N47 2.011(7)
Mo1–O35 1.774(5)	Cu8–N15 1.991(7)	Cu16–N30 1.968(8)	Cu24–O10 2.386(6)
Cu1–O1 1.901(6)	Cu8–N14 2.015(8)	Cu17–O16 1.901(6)	Cu25–O24 1.923(5)
Cu1–O9 1.909(6)	Cu9–O8 1.914(6)	Cu17–O17 1.927(6)	Cu25–O25 1.956(6)
Cu1–N18 1.945(7)	Cu9–O9 1.924(6)	Cu17–N33 1.940(9)	Cu25–N49 1.974(7)
Cu1–N1 1.972(8)	Cu9–N16 1.954(7)	Cu17–N32 1.973(8)	Cu25–N48 2.003(7)
Cu2–O2 1.933(6)	Cu9–N17 1.981(7)	Cu18–O18 1.919(7)	Cu26–O26 1.927(6)
Cu2–O1 1.973(6)	Cu10–O10 1.902(6)	Cu18–O17 1.919(6)	Cu26–O25 1.955(6)
Cu2–N3 1.990(8)	Cu10–N46 1.937(8)	Cu18–N35 1.931(9)	Cu26–N50 1.977(7)
Cu2–N2 1.994(7)	Cu10–O23 1.945(6)	Cu18–N34 1.945(8)	Cu26–N51 2.007(7)
Cu2–O11 2.362(5)	Cu10–N19 1.975(8)	Cu19–O19 1.924(7)	Cu27–O27 1.936(6)
Cu3–O2 1.900(6)	Cu11–O11 1.920(6)	Cu19–O18 1.924(7)	Cu27–O26 1.942(6)
Cu3–O3 1.918(6)	Cu11–O10 1.922(6)	Cu19–N36 1.956(9)	Cu27–N53 1.987(7)
Cu3–N5 1.946(8)	Cu11–N20 1.942(8)	Cu19–N37 1.985(9)	Cu27–N52 1.989(7)
Cu3–N4 1.969(7)	Cu11–N21 1.966(8)	Cu20–O20 1.903(6)	Cu27–O16 2.386(6)
Cu4–O4 1.911(6)	Cu12–O11 1.915(6)	Cu20–O19 1.919(7)	Cu28–O27 1.920(6)
Cu4–O3 1.912(6)	Cu12–O12 1.928(6)	Cu20–N38 1.954(8)	Cu28–O28 1.940(6)
Cu4–N6 1.934(8)	Cu12–N23 1.954(7)	Cu20–N39 1.966(8)	Cu28–N54 1.988(7)
Cu4–N7 1.969(8)	Cu12–N22 1.972(8)	Cu21–O20 1.905(7)	Cu28–N55 1.991(7)
Cu5–O4 1.924(6)	Cu13–O12 1.931(6)	Cu21–O21 1.915(6)	Cu29–O29 1.920(6)
Cu5–O5 1.977(6)	Cu13–O13 1.933(6)	Cu21–N40 1.955(8)	Cu29–O28 1.960(6)
Cu5–N9 1.986(7)	Cu13–N25 1.955(8)	Cu21–N41 1.967(10)	Cu29–N56 1.983(7)
Cu5–N8 1.996(7)	Cu13–N24 1.970(8)	Cu22–O22 1.917(6)	Cu29–N57 1.985(8)
Cu5–O15 2.333(5)	Cu14–O13 1.926(6)	Cu22–O21 1.923(6	Cu30–O29 1.926(6)
Cu6–O6 1.918(6)	Cu14–O14 1.930(6)	Cu22–N42 1.945(8)	Cu30–O30 1.967(6)
Cu6–O5 1.925(6)	Cu14–N26 1.950(9)	Cu22–N43 1.966(9)	Cu30–N59 1.987(8)
Cu6–N11 1.946(8)	Cu14–N27 1.992(8)	Cu23–O22 1.891(6)	Cu30–N58 1.999(8)
Cu6–N10 1.962(8)	Cu15–O15 1.897(6)	Cu23–O23 1.911(6)	Cu31–O31 1.914(6)
Cu7–O6 1.908(6)	Cu15–O14 1.930(6)	Cu23–N45 1.948(8)	Cu31–O30 1.924(6)
Cu7–O7 1.920(6)	Cu15–N28 1.938(8)	Cu23–N44 1.961(8)	Cu31–N61 1.959(7)
Cu7–N13 1.968(7)	Cu15–N29 1.952(8)	Cu24–O31 1.928(5)	Cu31–N60 1.984(8)

Table S17. Selected bond lengths for nanojar unit 2 (Cu_{32}) in **5** (Cu1–Cu8: Cu_8-ring; Cu9–Cu22: Cu_{14}-ring; Cu23–Cu31: Cu_9-ring).

Mo1–O33 1.697(9)	Cu8–O7 1.903(7)	Cu16–N31 1.947(8)	Cu25–O24 1.932(7)
Mo1–O35 1.728(9)	Cu8–O8 1.931(6)	Cu16–O16 1.951(6)	Cu25–O25 1.963(6)
Mo1–O36 1.732(8)	Cu8–N14 1.953(8)	Cu16–N30 1.973(8)	Cu25–N48 1.988(8)
Mo1–O34 1.751(9)	Cu8–N15 1.968(8)	Cu17–O16 1.932(6)	Cu25–N49 2.005(8)
Cu1–O8 1.957(6)	Cu9–O22 1.922(7)	Cu17–O17 1.933(7)	Cu25–O12 2.387(6)
Cu1–O1 1.969(6)	Cu9–O9 1.933(7)	Cu17–N33 1.959(8)	Cu26–O26 1.917(6)
Cu1–N1 1.979(9)	Cu9–N17 1.961(9)	Cu17–N32 1.967(9)	Cu26–O25 1.918(6)
Cu1–N16 2.006(8)	Cu9–N44 1.985(9)	Cu18–O18 1.938(6)	Cu26–N51 1.945(7)
Cu1–O9 2.390(7)	Cu10–O10 1.923(7)	Cu18–O17 1.943(7)	Cu26–N50 1.961(8)
Cu2–O1 1.934(6)	Cu10–O9 1.934(7)	Cu18–N34 1.945(8)	Cu27–O26 1.931(6)
Cu2–O2 1.943(6)	Cu10–N19 1.955(9)	Cu18–N35 1.958(9)	Cu27–O27 1.937(6)
Cu2–N2 1.980(8)	Cu10–N18 1.960(9)	Cu19–O19 1.907(7)	Cu27–N53 1.980(7)
Cu2–N3 2.000(8)	Cu11–O11 1.926(7)	Cu19–O18 1.913(7)	Cu27–N52 1.982(8)
Cu2–O11 2.392(7)	Cu11–O10 1.938(7)	Cu19–N37 1.957(9)	Cu28–O28 1.923(6)
Cu3–O2 1.924(6)	Cu11–N20 1.940(8)	Cu19–N36 1.963(8)	Cu28–O27 1.945(6)
Cu3–O3 1.925(6)	Cu11–N21 1.968(9)	Cu20–O20 1.916(7)	Cu28–N54 1.956(7)
Cu3–N5 1.975(8)	Cu12–O12 1.915(6)	Cu20–O19 1.945(7)	Cu28–N55 1.986(8)
Cu3–N4 1.992(8)	Cu12–O11 1.958(7)	Cu20–N38 1.945(9)	Cu29–O29 1.896(7)
Cu4–O3 1.904(6)	Cu12–N22 1.959(8)	Cu20–N39 2.007(8)	Cu29–O28 1.940(7)
Cu4–O4 1.925(6)	Cu12–N23 1.983(9)	Cu21–O20 1.920(7)	Cu29–N56 1.953(8)
Cu4–N6 1.963(7)	Cu13–O13 1.930(6)	Cu21–O21 1.941(6)	Cu29–N57 1.963(8)
Cu4–N7 1.979(8)	Cu13–O12 1.930(7)	Cu21–N40 1.952(9)	Cu30–O30 1.948(7)
Cu5–O4 1.944(6)	Cu13–N25 1.949(8)	Cu21–N41 1.960(9)	Cu30–O29 1.979(6)
Cu5–O5 1.948(6)	Cu13–N24 1.972(8)	Cu22–O22 1.919(7)	Cu30–N58 1.986(9)
Cu5–N9 1.983(8)	Cu14–N27 1.87(2)	Cu22–O21 1.926(7)	Cu30–N59 1.989(8)
Cu5–N8 1.994(8)	Cu14–O13 1.907(6)	Cu22–N42 1.957(9)	Cu30–O19 2.418(7)
Cu5–O16 2.370(6)	Cu14–O14 1.929(6)	Cu22–N43 1.967(9)	Cu31–O31 1.890(7)
Cu6–O6 1.941(7)	Cu14–N26 1.975(8)	Cu23–O23 1.907(6)	Cu31–O30 1.922(6)
Cu6–O5 1.942(6)	Cu14–N27B 2.02(2)	Cu23–O32 1.936(6)	Cu31–N60 1.956(8)
Cu6–N11 1.983(9)	Cu15–O15 1.913(6)	Cu23–N45 1.949(8)	Cu31–N61 1.960(8)
Cu6–N10 1.988(7)	Cu15–O14 1.929(6)	Cu23–N64 1.972(8)	Cu32–O31 1.916(6)
Cu7–O7 1.918(7)	Cu15–N28B 1.94(2)	Cu24–O24 1.899(6)	Cu32–O32 1.949(7)
Cu7–O6 1.941(7)	Cu15–N29 1.953(8)	Cu24–O23 1.922(6)	Cu32–N63 1.978(7)
Cu7–N12 1.979(8)	Cu15–N28 2.02(2)	Cu24–N46 1.938(8)	Cu32–N62 2.005(8)
Cu7–N13 2.005(8)	Cu16–O15 1.925(6)	Cu24–N47 1.945(8)	Cu32–O22 2.427(7)

Table S18. Selected bond lengths for **6** (Cu1–Cu9: Cu$_9$-ring; Cu10–Cu23: Cu$_{14}$-ring; Cu24–Cu31: Cu$_8$-ring).

W1–O4 1.737(7)
W1–O3 1.741(6)
W1–O2 1.745(5)
W1–O1 1.772(4)
Cu1–O1 1.930(5)
Cu1–O9 1.917(5)
Cu1–N1 1.973(6)
Cu1–N18 1.980(6)
Cu2–O1 1.912(5)
Cu2–O2 1.914(6)
Cu2–N2 1.940(8)
Cu2–N3 2.013(9)
Cu2–N3B 1.927(19)
Cu3–O2 1.961(5)
Cu3–O3 1.930(5)
Cu3–N4 1.968(9)
Cu3–N4B 2.073(18)
Cu3–N5 1.982(6)
Cu3–O13 2.395(6)
Cu4–O3 1.891(6)
Cu4–O4 1.958(6)
Cu4B–O3 2.004(11)
Cu4B–O4 1.824(11)
Cu4–N6 1.944(7)
Cu4–N7 2.007(9)
Cu4B–N6 2.105(11)
Cu4B–N7B 1.868(18)
Cu5–O4 1.976(6)
Cu5–O5 1.864(6)
Cu5B O4 1.762(10)
Cu5B–O5 2.025(10)
Cu5–N8 1.975(8)
Cu5–N9 1.924(8)
Cu5B–N8B 1.924(17)
Cu5B–N9 2.160(11)
Cu6–O5 1.939(5)
Cu6–O6 1.969(5)
Cu6–N11 1.979(6)
Cu6–N10 1.993(6)
Cu6–O17 2.377(5)
Cu7–O7 1.906(5)
Cu7–O6 1.918(5)
Cu7–N13 1.940(6)
Cu7–N12 1.983(6)
Cu8–O8 1.922(4)
Cu8 O7 1.926(5)
Cu8 N14 1.968(6)
Cu8 N15 1.996(6)
Cu9–O9 1.933(5)
Cu9–O8 1.936(5)
Cu9–N17 1.998(6)
Cu9–N16 2.008(7)
Cu10–O23 1.920(5)
Cu10–O10 1.921(5)
Cu10–N19 1.955(6)
Cu10–N46 1.971(6)
Cu11–O11 1.907(5)
Cu11–O10 1.912(5)
Cu11–N20 1.964(7)
Cu11–N21 1.936(13)
Cu11–N21B 2.07(5)
Cu12–O11 1.929(5)
Cu12–O12 1.907(5)
Cu12–N22 1.928(12)
Cu12–N22B 2.02(4)
Cu12–N23 1.965(7)
Cu13–O12 1.914(5)
Cu13–O13 1.926(5)
Cu13–N25 1.942(7)
Cu13–N24 1.959(7)
Cu14–O13 1.914(5)
Cu14–O14 1.936(5)
Cu14–N27 1.962(7)
Cu14–N26 1.971(7)
Cu15–O14 1.928(5)
Cu15–O15 1.930(5)
Cu15–N29 1.949(7)
Cu15–N28 1.958(7)
Cu16–O15 1.928(5)
Cu16–O16 1.928(5)
Cu16–N30 1.965(7)
Cu16–N31 1.969(7)
Cu17–O17 1.917(5)
Cu17–O16 1.931(5)
Cu17–N32 1.960(6)
Cu17–N33 1.964(6)
Cu18–O18 1.919(5)
Cu18–O17 1.926(5)
Cu18–N34 1.953(6)
Cu18–N35 1.967(6)
Cu19–O18 1.891(5)
Cu19–O19 1.923(5)
Cu19–N36 1.966(7)
Cu19–N37 2.000(11)
Cu19–N37B 1.931(17)
Cu20–O19 1.912(5)
Cu20–O20 1.920(5)
Cu20–N38 1.962(13)
Cu20–N38B 1.989(18)
Cu20–N39 1.955(7)
Cu21–O21 1.918(5)
Cu21–O20 1.925(5)
Cu21–N41 1.955(6)
Cu21–N40 1.964(6)
Cu22–O22 1.905(4)
Cu22–O21 1.927(5)
Cu22–N42 1.954(6)
Cu22–N43 1.964(6)
Cu23–O22 1.916(5)
Cu23–O23 1.917(5)
Cu23–N45 1.953(6)
Cu23–N44 1.969(6)
Cu24–O31 1.920(4)
Cu24–O24 1.963(5)
Cu24–N47 1.986(5)
Cu24–N62 2.031(6)
Cu24–O23 2.439(5)
Cu25–O25 1.896(5)
Cu25–O24 1.927(5)
Cu25–N49 1.962(7)
Cu25–N48 1.971(6)
Cu26–O25 1.913(5)
Cu26–O26 1.938(5)
Cu26–N50 1.982(6)
Cu26–N51 2.000(7)
Cu27–O26 1.923(5)
Cu27–O27 1.950(5)
Cu27–N53 1.992(6)
Cu27–N52 2.004(6)
Cu28–O28 1.914(4)
Cu28–O27 1.956(5)
Cu28–N54 1.984(6)
Cu28–N55 2.010(7)
Cu29–O29 1.927(5)
Cu29–O28 1.929(5)
Cu29–N57 1.972(6)
Cu29–N56 2.012(6)
Cu30–O29 1.901(5)
Cu30–O30 1.929(4)
Cu30–N58 1.988(6)
Cu30–N59 1.990(5)
Cu31–O31 1.915(4)
Cu31–O30 1.956(4)
Cu31–N60 1.984(6)
Cu31–N61 2.000(5)

Table S19. Hydrogen bonding data for **1** (Cu1–Cu9: Cu_9-ring; Cu10–Cu23: Cu_{14}-ring; Cu24–Cu31: Cu_8-ring; O32–O35, O32b–O35b, O32c–O35c: SeO_4^{2-} anion disordered over three positions in a 0.49/0.35/0.16 ratio).

D*—H···*A	***D*—H (Å)**	**H···*A* (Å)**	***D*···*A* (Å)**	***D*—H···*A* (°)**
O1—H1O···O33	0.83(3)	2.30(3)	3.125(11)	171(8)
O1—H1O···O33B	0.83(3)	2.16(6)	2.845(13)	140(8)
O1—H1O···O32C	0.83(3)	2.33(7)	3.13(3)	163(8)
O2—H2O···O34	0.83(2)	2.22(5)	2.919(11)	141(6)
O2—H2O···O33B	0.83(2)	2.04(5)	2.762(12)	145(7)
O2—H2O···O33C	0.83(2)	1.93(8)	2.66(4)	146(7)
O3—H3O···O34	0.84(2)	2.27(5)	3.008(12)	147(6)
O3—H3O···O33B	0.84(2)	1.94(3)	2.762(11)	165(7)
O3—H3O···O33C	0.84(2)	2.11(8)	2.87(4)	152(5)
O4—H4O···O33	0.81(2)	2.43(5)	3.103(12)	140(6)
O4—H4O···O35B	0.81(2)	2.56(3)	3.335(13)	161(6)
O5—H5O···O35	0.84(2)	2.20(4)	2.978(12)	153(6)
O5—H5O···O35B	0.84(2)	2.10(5)	2.826(12)	144(7)
O5—H5O···O35C	0.84(2)	2.07(5)	2.88(3)	161(7)
O6—H6O···O35	0.82(2)	1.95(3)	2.754(10)	168(7)
O6—H6O···O35B	0.82(2)	1.98(4)	2.752(12)	158(7)
O6—H6O···O35C	0.82(2)	2.20(6)	2.99(3)	161(6)
O7—H7O···O35	0.83(3)	2.57(5)	3.320(12)	150(8)
O7—H7O···O35B	0.83(3)	2.11(5)	2.854(13)	149(8)
O7—H7O···O32C	0.83(3)	2.48(7)	3.18(2)	143(8)
O8—H8O···O32	0.83(2)	2.14(3)	2.965(17)	171(7)
O8—H8O···O32B	0.83(2)	2.04(4)	2.87(2)	171(6)
O8—H8O···O32C	0.83(2)	2.35(7)	3.03(2)	139(6)
O9—H9O···O32	0.82(2)	2.10(4)	2.91(2)	165(6)
O9—H9O···O32B	0.82(2)	2.22(4)	3.01(3)	162(5)
O9—H9O···O32C	0.82(2)	2.37(8)	3.01(3)	135(5)
O10—H10O···O24	0.83(2)	2.11(3)	2.917(6)	165(7)
O11—H11O···O2	0.84(2)	2.35(4)	3.138(6)	158(7)
O12—H12O···O2	0.83(2)	2.04(3)	2.842(6)	164(7)
O13—H13O···O26	0.84(2)	1.96(3)	2.791(6)	171(8)
O14—H14O···O3	0.84(2)	2.02(3)	2.830(6)	163(7)
O15—H15O···O27	0.84(2)	2.06(3)	2.894(6)	176(7)
O16—H16O···O5	0.83(2)	1.97(3)	2.804(6)	174(7)
O17—H17O···O28	0.82(2)	2.01(3)	2.815(6)	167(7)
O18—H18O···O6	0.83(2)	2.00(3)	2.794(5)	161(7)
O20—H20O···O30	0.83(2)	2.10(3)	2.916(6)	166(7)
O21—H21O···O8	0.82(2)	2.10(3)	2.904(6)	167(7)
O22—H22O···O31	0.82(2)	2.00(3)	2.812(6)	172(7)
O23—H23O···O9	0.84(2)	2.07(3)	2.897(6)	171(7)
O24—H24O···O32	0.83(2)	2.24(3)	3.063(15)	170(7)
O24—H24O···O32B	0.83(2)	2.47(3)	3.29(2)	170(7)
O24—H24O···O34C	0.83(2)	2.20(4)	2.99(2)	159(7)
O25—H25O···O34	0.82(2)	2.17(5)	2.870(12)	143(7)
O25—H25O···O34B	0.82(2)	2.46(4)	3.228(13)	155(7)

Table S19 (continued).

D*—H···*A	***D*—H (Å)**	**H···*A* (Å)**	***D*···*A* (Å)**	***D*—H···*A* (°)**
O25—H25O···O33C	0.82(2)	2.39(6)	3.06(3)	139(7)
O26—H26O···O34	0.83(2)	1.89(3)	2.716(11)	174(8)
O26—H26O···O34B	0.83(2)	2.46(5)	3.142(13)	140(6)
O26—H26O···O33C	0.83(2)	2.18(6)	2.99(3)	167(7)
O27—H27O···O34B	0.82(2)	2.11(3)	2.921(12)	170(7)
O28—H28O···O35	0.84(2)	2.06(3)	2.859(12)	160(5)
O28—H28O···O34B	0.84(2)	2.37(5)	3.088(12)	143(6)
O28—H28O···O35C	0.84(2)	1.95(8)	2.78(4)	168(5)
O29—H29O···O35	0.83(2)	2.18(3)	3.004(13)	173(7)
O29—H29O···O34B	0.83(2)	2.50(6)	3.167(13)	138(7)
O29—H29O···O35C	0.83(2)	2.37(6)	3.19(4)	173(10)
O30—H30O···O32	0.83(2)	2.31(3)	3.12(2)	167(5)
O30—H30O···O32B	0.83(2)	2.20(4)	3.02(3)	168(6)
O30—H30O···O34C	0.83(2)	2.07(7)	2.87(3)	161(5)
O31—H31O···O32	0.82(2)	2.03(3)	2.838(12)	171(7)
O31—H31O···O32B	0.82(2)	2.12(3)	2.931(16)	174(7)
O31—H31O···O34C	0.82(2)	2.20(8)	2.92(3)	148(6)

Table S20. Hydrogen bonding data for **2** (O1–O9: Cu_9-ring; O10–O23: Cu_{14}-ring; O24–O32: Cu_9-ring; O33–O36 and O33B–O36B: SeO_4^{2-} anion disordered over two positions in a 0.66/0.34 ratio).

D*—H···*A	***D*—H (Å)**	**H···*A* (Å)**	***D*···*A* (Å)**	***D*—H···*A* (°)**
O1—H1O···O34	0.85(3)	2.55(5)	3.373(15)	164(13)
O2—H2O···O36	0.83(3)	2.30(3)	3.003(15)	143(5)
O2—H2O···O36B	0.83(3)	2.32(4)	3.11(2)	160(7)
O3—H3O···O36	0.84(3)	1.86(3)	2.693(12)	174(12)
O3—H3O···O36B	0.84(3)	2.29(5)	3.09(2)	161(10)
O4—H4O···O36	0.83(3)	2.24(5)	3.038(16)	163(11)
O4—H4O···O35B	0.83(3)	2.66(7)	3.41(3)	151(10)
O5—H5O···O33	0.83(3)	2.15(5)	2.948(16)	160(11)
O5—H5O···O35B	0.83(3)	2.13(8)	2.82(3)	140(11)
O6—H6O···O33	0.84(3)	2.10(4)	2.925(16)	167(12)
O6—H6O···O35B	0.84(3)	1.98(6)	2.76(2)	156(12)
O7—H7O···O33	0.84(3)	2.31(6)	3.066(16)	150(10)
O8—H8O···O34	0.85(2)	2.37(5)	3.094(13)	143(8)
O8—H8O···O34B	0.85(2)	1.97(2)	2.786(19)	161(8)
O9—H9O···O34	0.83(3)	1.97(5)	2.747(12)	156(11)
O9—H9O···O34B	0.83(3)	2.49(5)	3.29(3)	160(10)
O11—H11O···O24	0.85(3)	2.06(5)	2.850(9)	154(10)
O12—H12O···O2	0.85(3)	2.15(5)	2.940(9)	155(10)
O13—H13O···O25	0.83(3)	2.01(3)	2.756(9)	149(6)
O14—H14O···O3	0.83(3)	2.06(6)	2.803(9)	152(11)
O15—H15O···O26	0.84(3)	2.13(4)	2.961(9)	169(11)
O16—H16O···O4	0.84(3)	2.13(6)	2.900(10)	153(10)
O17—H17O···O28	0.82(3)	1.95(4)	2.754(9)	165(11)
O18—H18O···O5	0.85(3)	2.10(5)	2.850(9)	147(4)
O19—H19O···O29	0.83(3)	1.95(4)	2.755(8)	163(11)
O20—H20O···O7	0.84(3)	1.92(3)	2.755(9)	174(11)
O21—H21O···O30	0.84(3)	1.93(3)	2.770(9)	173(10)
O22—H22O···O8	0.85(3)	2.02(6)	2.753(8)	145(9)
O23—H23O···O32	0.83(2)	1.97(2)	2.750(8)	157(7)
O24—H24O···O34	0.86(3)	2.33(3)	3.088(13)	148(6)
O24—H24O···O36B	0.86(3)	2.61(8)	3.28(3)	137(8)
O25—H25O···O36	0.84(3)	2.57(6)	3.336(19)	152(10)
O25—H25O···O36B	0.84(3)	1.94(5)	2.748(17)	161(11)
O26—H26O···O36	0.84(3)	2.62(8)	3.293(19)	138(10)
O26—H26O···O36B	0.84(3)	2.24(6)	3.02(2)	154(11)
O27—H27O···O35	0.84(3)	2.16(8)	2.854(15)	140(10)
O27—H27O···O33B	0.84(3)	2.20(7)	2.97(3)	151(11)
O28—H28O···O33B	0.84(3)	2.03(6)	2.81(2)	154(10)
O29—H29O···O33	0.82(3)	2.16(4)	2.947(14)	161(8)
O29—H29O···O33B	0.82(3)	2.28(6)	3.01(2)	148(9)
O30—H30O···O33B	0.84(3)	2.35(5)	3.15(3)	159(9)
O30—H30O···O34B	0.84(3)	2.58(7)	3.28(3)	141(8)
O31—H31O···O35	0.83(3)	2.20(3)	2.935(16)	147(6)
O31—H31O···O33B	0.83(3)	2.61(4)	3.40(3)	158(8)
O31—H31O···O34B	0.83(3)	2.35(7)	3.07(3)	145(8)
O32—H32O···O34	0.76(10)	2.08(11)	2.832(12)	168(11)

Table S21. Hydrogen bonding data for **3** (O1–O6: Cu_6-ring; O7–O18: Cu_{12}-ring; O19–O28: Cu_9-ring; O29–O32: MoO_4^{2-} anion).

D*—H···*A	***D*—H (Å)**	**H···*A* (Å)**	***D*···*A* (Å)**	***D*—H···*A* (°)**
O1—H1O···O30	0.824(19)	2.35(4)	3.110(4)	153(4)
O3—H3O···O32	0.836(19)	1.94(2)	2.771(3)	176(4)
O4—H4O···O32	0.835(19)	2.37(3)	3.173(3)	162(4)
O6—H6O···O30	0.821(19)	1.89(3)	2.703(3)	173(4)
O7—H7O···O1	0.826(19)	1.97(3)	2.759(3)	160(4)
O8—H8O···O20	0.831(19)	2.03(3)	2.806(3)	155(4)
O9—H9O···O2	0.815(19)	2.03(4)	2.818(3)	163(4)
O10—H10O···O22	0.831(19)	2.02(2)	2.844(3)	172(4)
O11—H11O···O3	0.805(19)	1.90(4)	2.702(3)	176(4)
O12—H12O···O23	0.831(19)	2.03(3)	2.839(3)	164(3)
O13—H13O···O4	0.828(19)	1.97(3)	2.799(3)	173(4)
O14—H14O···O25	0.826(19)	1.91(3)	2.723(3)	168(4)
O15—H15O···O5	0.828(19)	2.04(5)	2.829(4)	160(3)
O16—H16O···O27	0.832(19)	1.96(2)	2.778(3)	168(4)
O17—H17O···O6	0.848(19)	1.87(5)	2.706(4)	169(4)
O18—H18O···O28	0.839(19)	2.08(4)	2.885(3)	162(4)
O19—H19O···O29	0.829(19)	2.32(3)	3.077(3)	152(5)
O20—H20O···O29	0.830(19)	2.04(4)	2.866(4)	172(4)
O21—H21O···O29	0.817(19)	2.21(5)	2.960(4)	152(4)
O22—H22O···O32	0.840(19)	2.12(4)	2.913(4)	157(4)
O23—H23O···O32	0.827(19)	2.06(4)	2.871(4)	167(4)
O24—H24O···O31	0.829(19)	2.35(4)	3.092(4)	150(5)
O25—H25O···O31	0.837(19)	2.00(4)	2.818(4)	167(4)
O26—H26O···O31	0.834(19)	2.15(5)	2.958(5)	163(4)
O27—H27O···O30	0.826(19)	2.31(5)	3.076(5)	154(4)
O28—H28O···O30	0.825(19)	2.10(4)	2.880(4)	160(4)
O37—H37A···O21	0.84(2)	2.00(11)	2.766(10)	153(6)

Table S22. Hydrogen bonding data for **4** (O1_1–O8_1: Cu_8-ring; O9_1–O22_1: Cu_{14}-ring; O23_1–O31_1: Cu_9-ring; O1_2–O4_2: MoO_4^{2-} anion).

D*—H···*A	***D*—H (Å)**	**H···*A* (Å)**	***D*···*A* (Å)**	***D*—H···*A* (°)**
O1_1—H1O_1···O2_2	0.83(2)	2.03(3)	2.803(6)	156(7)
O3_1—H3O_1···O4_2	0.84(2)	1.89(2)	2.716(6)	168(7)
O4_1—H4O_1···O4_2	0.84(2)	2.13(3)	2.933(6)	158(7)
O5_1—H5O_1···O1_2	0.83(2)	2.33(4)	3.046(5)	145(6)
O6_1—H6O_1···O1_2	0.83(2)	1.97(2)	2.782(5)	169(7)
O7_1—H7O_1···O1_2	0.84(2)	2.19(6)	2.998(6)	161(5)
O8_1—H8O_1···O2_2	0.83(2)	2.21(3)	3.018(6)	165(7)
O9_1—H9O_1···O1_1	0.84(2)	1.96(2)	2.801(5)	172(7)
O10_1—H10O_1···O23_1	0.83(2)	1.99(2)	2.821(5)	178(7)
O11_1—H11O_1···O2_1	0.84(2)	2.09(3)	2.892(5)	160(7)
O12_1—H12O_1···O25_1	0.85(2)	2.05(6)	2.823(6)	150(5)
O13_1—H13O_1···O3_1	0.84(2)	1.94(2)	2.768(5)	169(7)
O14_1—H14O_1···O26_1	0.84(2)	2.01(3)	2.824(5)	165(7)
O15_1—H15O_1···O26_1	0.83(2)	2.40(3)	3.195(5)	159(7)
O16_1—H16O_1···O5_1	0.84(2)	2.08(2)	2.914(5)	178(8)
O17_1—H17O_1···O28_1	0.83(2)	2.06(3)	2.864(5)	162(7)
O18_1—H18O_1···O6_1	0.82(2)	2.01(3)	2.794(5)	160(7)
O19_1—H19O_1···O29_1	0.83(2)	2.07(3)	2.882(5)	165(7)
O20_1—H20O_1···O7_1	0.83(2)	2.11(3)	2.931(5)	167(7)
O21_1—H21O_1···O8_1	0.84(2)	2.63(6)	3.247(6)	131(7)
O22_1—H22O_1···O31_1	0.84(2)	1.95(2)	2.784(5)	175(7)
O23_1—H23O_1···O2_2	0.85(2)	2.12(4)	2.900(6)	152(7)
O24_1—H24O_1···O3_2	0.83(2)	2.48(4)	3.256(7)	156(7)
O25_1—H25O_1···O4_2	0.83(2)	2.14(3)	2.907(5)	155(7)
O26_1—H26O_1···O4_2	0.84(2)	2.03(4)	2.796(6)	152(7)
O27_1—H27O_1···O3_2	0.83(2)	2.35(2)	3.170(9)	173(8)
O28_1—H28O_1···O1_2	0.84(2)	2.15(3)	2.951(5)	159(7)
O29_1—H29O_1···O1_2	0.83(2)	2.13(3)	2.912(5)	155(6)
O31_1—H31O_1···O2_2	0.83(2)	1.96(5)	2.718(6)	155(6)

Table S23. Hydrogen bonding data for nanojar unit 1 (Cu_{31}) in **5** (O1–O9: Cu_9-ring; O10–O23: Cu_{14}-ring; O24–O31: Cu_8-ring; O32_1–O35_1: MoO_4^{2-} anion).

D*—H···*A	***D*—H (Å)**	**H···*A* (Å)**	***D*···*A* (Å)**	***D*—H···*A* (°)**
O1_1—H1O_1···O35_1	0.82(2)	1.986(19)	2.723(8)	149(6)
O2_1—H2O_1···O35_1	0.81(2)	2.085(16)	2.760(8)	140(3)
O4_1—H4O_1···O32_1	0.84(2)	1.97(4)	2.769(8)	161(7)
O5_1—H5O_1···O32_1	0.829(19)	2.00(2)	2.736(8)	147(4)
O6_1—H6O_1···O32_1	0.83(2)	2.46(4)	3.134(8)	140(6)
O7_1—H7O_1···O33_1	0.827(19)	2.09(6)	2.825(8)	147(6)
O8_1—H8O_1···O33_1	0.845(16)	2.01(6)	2.855(7)	173(7)
O9_1—H9O_1···O35_1	0.905(16)	2.42(6)	3.130(8)	135(3)
O10_1—H10O_1···O1_1	0.83(2)	1.95(7)	2.750(8)	162(8)
O11_1—H11O_1···O24_1	0.83(2)	2.11(6)	2.799(8)	139(8)
O12_1—H12O_1···O2_1	0.84(2)	1.99(4)	2.811(8)	164(8)
O13_1—H13O_1···O25_1	0.85(2)	2.18(5)	2.978(8)	157(9)
O14_1—H14O···O4_1	0.846(19)	2.01(4)	2.792(8)	154(5)
O15_1—H15O_1···O26_1	0.83(2)	2.00(7)	2.818(8)	166(7)
O16_1—H16O_1···O5_1	0.84(2)	1.97(7)	2.761(8)	155(7)
O18_1—H18O_1···O28_1	0.83(2)	2.17(9)	2.907(9)	148(9)
O19_1—H19O_1···O7_1	0.84(2)	2.16(9)	2.884(9)	144(9)
O20_1—H20O_1···O29_1	0.848(17)	1.97(4)	2.803(8)	169(10)
O21_1—H21O_1···O8_1	0.873(17)	2.04(4)	2.910(8)	171(10)
O22_1—H22O_1···O30_1	0.83(2)	2.05(9)	2.879(9)	173(9)
O24_1—H24O_1···O35_1	0.83(2)	2.21(6)	2.979(8)	154(9)
O26_1—H26O_1···O32_1	0.833(18)	2.19(5)	2.999(8)	162(7)
O27_1—H27O_1···O34_1	0.852(18)	2.12(3)	2.951(8)	163(9)
O28_1—H28O_1···O33_1	0.860(17)	2.23(7)	3.083(9)	171(6)
O29_1—H29O_1···O33_1	0.82(2)	2.03(4)	2.811(8)	159(10)
O30_1—H30O_1···O33_1	0.84(2)	2.38(7)	3.111(8)	144(6)
O31_1—H31O_1···O34_1	0.836(18)	2.21(3)	2.985(8)	154(7)

Table S24. Hydrogen bonding data for nanojar unit 2 (Cu_{32}) in **5** (O1–O8: Cu_8-ring; O9–O22: Cu_{14}-ring; O23–O32: Cu_{10}-ring; O33_2–O35_2: MoO_4^{2-} anion).

D*—H⋯*A	***D*—H (Å)**	**H⋯*A* (Å)**	***D*⋯*A* (Å)**	***D*—H⋯*A* (°)**
O2_2—H2O_2⋯O35_2	0.83(2)	2.19(5)	3.003(10)	165(7)
O3_2—H3O_2⋯O36_2	0.84(2)	2.34(8)	3.183(11)	176(9)
O4_2—H4O_2⋯O36_2	0.83(2)	2.37(6)	3.022(11)	136(6)
O5_2—H5O_2⋯O36_2	0.83(2)	2.11(5)	2.910(12)	162(8)
O6_2—H6O_2⋯O36_2	0.84(2)	2.15(8)	2.974(11)	170(9)
O8_2—H8O_2⋯O33_2	0.83(2)	2.02(8)	2.826(12)	164(8)
O9_2—H9O_2⋯O32_2	0.835(17)	2.00(7)	2.828(9)	168(7)
O10_2—H10O_2⋯O1_2	0.871(15)	2.33(10)	2.821(9)	81(5)
O11_2—H11O_2⋯O24_2	0.85(2)	1.97(10)	2.803(9)	164(11)
O12_2—H12O_2⋯O2_2	0.843(17)	2.02(8)	2.838(8)	167(7)
O13_2—H13O_2⋯O25_2	0.839(18)	2.00(5)	2.821(8)	164(6)
O15_2—H15O_2⋯O4_2	0.83(2)	2.03(7)	2.813(9)	157(10)
O16_2—H16O_2⋯O27_2	0.83(2)	2.00(8)	2.805(9)	161(9)
O17_2—H17O_2⋯O5_2	0.828(19)	2.10(7)	2.860(9)	154(7)
O18_2—H18O_2⋯O29_2	0.85(2)	2.09(11)	2.886(10)	155(11)
O19_2—H19O_2⋯O6_2	0.84(2)	2.12(8)	2.856(9)	146(8)
O20_2—H20O_2⋯O30_2	0.844(16)	2.01(6)	2.852(9)	172(9)
O22_2—H22O_2⋯O8_2	0.83(2)	2.00(9)	2.789(9)	158(10)
O24_2—H24O_2⋯O35_2	0.814(18)	2.18(4)	2.934(12)	154(6)
O25_2—H25O_2⋯O35_2	0.84(2)	2.16(8)	2.906(14)	146(9)
O27_2—H27O_2⋯O34_2	0.858(17)	2.22(4)	3.065(11)	166(8)
O28_2—H28O_2⋯O34_2	0.83(2)	2.06(9)	2.768(12)	143(8)
O31_2—H31O_2⋯O33_2	0.83(2)	2.26(5)	2.787(12)	122(6)
O32_2—H32O_2⋯O33_2	0.83(2)	2.30(8)	2.977(13)	139(5)

Table S25. Hydrogen bonding data for **6** (O1_1–O9_1: Cu_9-ring; O10_1–O23_1: Cu_{14}-ring; O24_1–O31_1: Cu_8-ring; O1_2–O4_2: WO_4^{2-} anion).

D*—H···*A	***D*—H (Å)**	**H···*A* (Å)**	***D*···*A* (Å)**	***D*—H···*A* (°)**
O2_1—H2O_1···O2_2	0.83(2)	1.94(7)	2.748(7)	165(9)
O3_1—H3O_1···O2_2	0.84(2)	2.14(6)	2.919(8)	154(10)
O4_1—H4O_1···O4_2	0.83(2)	2.34(11)	3.175(12)	172(7)
O5_1—H5O_1···O3_2	0.84(2)	2.13(8)	2.905(9)	153(5)
O6_1—H6O_1···O3_2	0.84(2)	1.95(7)	2.768(9)	167(8)
O8_1—H8O_1···O1_2	0.83(2)	2.17(9)	2.935(8)	154(8)
O9_1—H9O_1···O1_2	0.82(2)	2.10(5)	2.892(6)	166(7)
O10_1—H10O_1···O24_1	0.83(2)	2.13(4)	2.941(5)	161(9)
O12_1—H12O_1···O2_1	0.83(2)	1.98(7)	2.785(8)	163(10)
O13_1—H13O_1···O26_1	0.83(2)	1.98(4)	2.800(5)	167(10)
O14_1—H14O_1···O3_1	0.85(2)	2.04(11)	2.801(9)	150(8)
O15_1—H15O_1···O27_1	0.83(2)	2.17(7)	2.903(7)	145(9)
O16_1—H16O_1···O5_1	0.84(2)	2.00(9)	2.796(8)	161(7)
O17_1—H17O_1···O28_1	0.83(2)	1.96(7)	2.781(7)	169(10)
O18_1—H18O_1···O6_1	0.83(2)	2.00(5)	2.822(5)	166(7)
O20_1—H20O_1···O30_1	0.83(2)	2.10(8)	2.907(8)	165(7)
O21_1—H21O_1···O8_1	0.84(2)	2.04(5)	2.852(6)	165(7)
O22_1—H22O_1···O31_1	0.84(2)	1.96(10)	2.799(7)	171(8)
O23_1—H23O_1···O9_1	0.83(2)	2.07(7)	2.885(7)	174(7)
O24_1—H24O_1···O1_2	0.84(2)	2.22(8)	3.020(7)	158(8)
O25_1—H25O_1···O2_2	0.84(2)	2.20(6)	2.977(10)	157(10)
O26_1—H26_1···O2_2	0.84(2)	1.97(11)	2.767(10)	162(10)
O28_1—H28O_1···O3_2	0.84(2)	1.91(5)	2.750(8)	173(8)
O29_1—H29O_1···O1_2	0.83(2)	2.13(6)	2.947(7)	168(7)
O30_1—H30O_1···O1_2	0.83(2)	2.28(4)	3.046(5)	154(8)
O31_1—H31O_1···O1_2	0.83(2)	1.98(8)	2.773(7)	161(8)

4. NMR SPECTROSCOPIC DATA

Table S26. Variable-temperature ^{1}H NMR chemical shifts (ppm) in DMSO-d_6 of **Cu_nWO_4**, **Cu_nMoO_4**, **Cu_nCrO_4**, **Cu_nSeO_4** and **Cu_nSO_4** nanojars. Missing values are due to lack of significant amounts of a particular species in the mixture and/or to impossibility of unambiguous assignments due to overlap or excessive broadening.

NANOJAR	22 °C	30 °C	40 °C	50 °C	60 °C	70 °C	80 °C	90 °C	100 °C	110 °C	120 °C	130 °C	140 °C	150 °C
**Cu_{28} (6+12+10)**														
Cu_{10} ring, pz-4-*H*	34.95	34.63	34.25	33.88	33.52	33.16	32.81	32.47	32.13	31.81	31.48	–	–	–
	35.24	34.90	34.51	34.13	33.78	33.45	33.12	32.77	32.43	32.09	31.80	31.47	~–31.1	–
	37.48	37.08	36.63	36.19	35.76	35.34	34.93	34.53	34.15	33.77	33.39	33.03	–	–
	37.66	37.28	36.82	36.36	35.92	35.48	35.06	34.66	34.26	33.87	33.49	33.13	32.78	–
	40.27	39.99	39.46	38.94	38.42	37.92	37.43	36.94	36.47	36.01	35.56	35.12	34.69	34.28
Cu_{10} ring, pz-3,5-H_2	30.64	30.37	30.05	29.73	29.42	29.11	28.81	28.51	28.22	27.94	27.65	–	–	–
	30.88	30.59	30.26	29.94	29.63	29.34	29.05	28.75	28.45	28.20	27.94	27.65	~27.4	–
	32.58	32.24	31.85	31.47	31.10	~30.74	30.39	30.03	29.70	29.37	~29.05	28.73	–	–
	32.67	32.35	31.95	31.56	31.18	30.82	30.46	30.11	29.77	29.43	29.10	28.79	28.48	–
	34.50	34.27	33.81	33.37	32.94	32.51	32.08	31.67	31.27	30.89	30.51	30.15	29.81	29.41
Cu_6 ring, pz-4-*H*	34.45	34.47	34.46	34.42	34.35	34.24	34.12	33.97	33.81	33.63	33.43	–	–	–
	34.38	34.41	34.40	34.36	34.29	34.20	34.08	33.95	33.81	33.66	33.50	33.31	~33.1	–
	33.71	33.78	33.82	33.83	33.79	33.74	33.65	33.55	33.42	33.28	33.13	~32.97	–	–
	34.18	34.21	34.22	34.19	34.12	34.03	33.92	33.79	33.64	33.48	33.30	33.11	32.93	–
	33.61	33.63	33.67	33.67	33.64	33.60	33.52	33.41	33.31	33.17	33.03	32.86	32.70	32.54
Cu_6 ring, pz-3,5-H_2	31.37	31.39	31.37	31.32	31.25	31.16	31.04	30.91	30.77	30.63	30.45	–	–	–
	31.31	31.32	31.30	31.26	31.19	31.11	31.00	30.88	30.75	30.62	30.47	30.30	~30.1	–
	30.88	30.92	~30.93	~30.91	30.86	~30.74	30.70	30.59	30.47	30.33	30.18	30.02	–	–
	31.21	31.23	31.22	31.17	31.11	31.01	30.91	30.78	30.64	30.49	30.33	30.16	29.99	–
	30.77	30.79	30.80	30.79	30.74	30.68	30.59	30.48	30.37	30.24	30.10	29.92	29.61	29.41
Cu_{12} ring, pz-4-*H(10)*	29.09	29.13	29.17	29.20	29.22	29.23	29.23	29.22	29.19	29.16	29.12	–	–	–
	29.07	29.11	29.16	29.19	29.21	29.22	29.22	29.21	29.19	29.16	29.13	29.08	~29.0	–
	29.09	29.13	29.17	29.20	29.21	29.22	29.21	29.19	29.17	29.13	29.08	29.03	–	–
	29.06	29.11	29.15	29.19	29.21	29.22	29.22	29.21	29.19	29.15	29.11	29.06	29.00	–
	28.97	29.00	29.06	29.09	29.12	29.14	29.14	29.12	29.11	29.08	29.03	28.98	28.92	28.86
Cu_{12} ring, pz-4-*H(6)*	27.27	27.30	27.33	27.36	27.36	27.36	27.35	27.32	27.29	27.24	27.19	–	–	–
	27.28	27.31	27.34	27.36	27.37	27.37	27.36	27.34	27.31	27.27	27.23	27.17	~27.1	–
	27.44	27.48	27.51	27.53	27.54	27.53	27.52	27.49	27.45	27.41	27.35	27.29	–	–
	27.44	27.48	27.52	27.54	27.55	27.55	27.54	27.52	27.48	27.44	27.39	27.33	27.26	–
	27.48	27.50	27.56	27.60	27.61	27.63	27.61	27.60	27.56	27.52	27.47	27.41	27.35	~27.3
Cu_{12} ring, pz-3,5-H_2*(10)*	23.37	23.38	23.39	23.39	23.38	23.37	23.34	23.31	23.27	23.23	23.18	–	–	–
	23.36	23.38	23.39	23.39	23.38	23.37	23.35	23.32	23.29	23.25	23.20	23.15	~23.1	–
	23.44	23.45	23.46	23.46	23.45	23.44	23.41	23.38	23.34	23.29	23.24	23.18	–	–
	23.42	23.44	23.45	23.46	23.45	23.44	23.42	23.39	23.35	23.31	23.26	23.20	23.14	–
	23.40	23.41	23.43	23.44	23.46	23.44	23.43	23.40	23.37	23.32	23.27	23.23	23.17	23.09
Cu_{12} ring, pz-3,5-H_2*(6)*	23.25	23.27	23.28	23.29	23.29	23.28	23.27	23.25	23.22	23.18	23.14	–	–	–
	23.24	23.26	23.28	23.29	23.29	23.28	23.27	23.25	23.22	23.19	23.16	23.12	~23.0	–
	23.28	23.30	23.31	23.31	23.31	23.30	23.28	23.25	23.22	23.18	23.14	23.09	–	–
	23.25	23.27	23.29	23.30	23.30	23.29	23.28	23.26	23.23	23.19	23.15	23.10	23.05	–
	23.23	23.24	23.27	23.29	23.29	23.29	23.27	23.24	23.23	23.18	23.14	23.09	23.05	22.98
Cu_{12} ring, O*H(6)*	−29.74	−29.67	−29.55	−29.40	−29.26	−29.10	−28.93	−28.75	−28.59	−28.43	~−28.3	–	–	–
	−29.96	−29.86	−29.72	−29.59	−29.44	−29.29	−29.14	−28.97	−28.81	−28.63	−28.47	−28.37	~−28.3	–
	−31.70	−31.57	−31.41	−31.23	−31.05	−30.86	−30.67	−30.46	−30.26	−30.05	−29.83	−29.61	–	–
	−31.37	−31.26	−31.10	−30.94	−30.77	−30.59	−30.41	−30.22	−30.02	−29.81	−29.60	−29.39	~−29.2	–
	−32.98	−32.91	−32.74	−32.57	−32.42	−32.19	−32.01	−31.79	−31.53	−31.32	−31.06	−30.79	−30.53	~−30.2
Cu_{12} ring, O*H(10)*	−44.07	−44.06	−44.03	−43.96	−43.88	−43.78	−43.66	−43.50	~−43.4	~−43.2	~−43.0	–	–	–
	−44.00	−44.01	−43.98	−43.93	−43.86	−43.76	−43.65	−43.51	−43.38	~−43.2	~−43.0	~−42.7	~−42.5	–
	−43.85	−43.91	−43.95	−43.96	−43.93	−43.89	−43.82	−43.71	−43.59	−43.43	−43.26	−43.08	–	–
	−43.82	−43.87	−43.92	−43.93	−43.91	−43.87	−43.80	−43.68	−43.56	−43.42	−43.23	−43.07	~−42.9	–
	−43.47	−43.56	−43.69	−43.78	−43.81	−43.84	−43.85	−43.76	−43.61	−43.41	~−43.2	~−43.1	~−43.0	~−42.9
Cu_6 ring, O*H*	−46.19	~−46.6	−47.05	−47.40	−47.66	−47.83	−47.91	−47.92	−47.82	−47.68	~−47.4	–	–	–
	−46.03	−46.54	−46.88	−47.25	−47.49	−47.64	−47.72	−47.74	−47.67	−47.54	−47.38	−47.17	−46.9	–
	−49.49	−49.91	−50.29	−50.57	~−50.7	−50.80	−50.80	−50.72	−50.55	−50.33	−50.05	−49.73	–	–
	−50.76	−51.14	~−51.6	−51.79	−51.91	−51.96	−51.90	−51.77	−51.56	−51.29	−50.94	−50.61	~−50.3	–
	−51.49	−51.66	−51.95	−52.13	−52.27	−52.16	~−52.0	−52.07	−51.88	−51.66	−51.35	−51.08	−50.69	−50.27
Cu_{10} ring, O*H*	−46.66	−46.47	−46.08	−45.71	−45.32	−44.90	−44.46	−44.01	−43.54	−43.08	~−42.6	–	–	–
	−47.24	−46.90	−46.52	−46.09	−45.66	−45.23	−44.79	−44.33	−43.87	−43.36	−42.93	−42.50	~−42.0	–
	−54.03	−53.23	−52.34	−51.46	~−50.7	−49.76	−48.94	−48.13	−47.34	−46.55	−45.79	−45.04	–	–
	−53.12	−52.51	~−51.7	−50.90	−50.10	−49.31	−48.54	−47.78	−47.02	−46.28	−45.55	−44.86	~−44.2	–
	−57.75	−57.18	−56.10	−55.05	−53.95	−52.94	~−51.9	~−51.0	−50.02	−49.09	−48.19	−47.29	−46.48	−45.67

Table S26 (continued).

NANOJAR	22 °C	30 °C	40 °C	50 °C	60 °C	70 °C	80 °C	90 °C	100 °C	110 °C	120 °C	130 °C	140 °C	150 °C
**Cu_{31} (8+14+9)**														
	–	–	~30.8	30.63	30.46	30.30	30.13	29.97	29.81	29.65	29.49	29.34	29.19	29.04
	–	–	30.65	30.49	30.34	30.20	30.06	29.91	29.78	29.64	29.51	29.37	29.28	29.15
Cu_9 ring, pz-4-*H*	~31.2	~31.1	~31.0	~30.9	30.77	30.62	30.46	30.32	30.17	30.03	29.88	29.73	29.59	29.45
	–	–	–	~31.1	30.91	30.73	30.57	30.41	30.24	30.08	29.92	29.83	29.67	29.45
	32.95	32.79	32.62	32.39	32.19	31.99	31.77	31.60	31.40	31.21	31.03	30.86	30.70	30.51
	–	–	~27.1	27.02	26.90	26.78	26.65	26.53	26.40	26.27	26.15	26.02	25.89	25.77
	~27.2	27.17	27.06	26.95	26.85	26.74	26.63	26.52	26.41	26.29	26.18	26.06	25.96	25.89
Cu_9 ring, pz-3,5-H_2	~27.6	~27.6	~27.5	27.33	27.21	27.10	26.98	26.86	26.74	26.61	26.49	26.36	26.24	~26.10
	–	–	27.56	27.45	27.33	27.21	27.07	26.94	26.81	26.67	26.54	26.45	26.32	26.13
	28.73	28.76	28.57	28.40	28.25	28.10	27.93	27.78	27.63	27.49	27.33	27.20	27.06	26.91
	–	–	~29.4	29.41	29.42	29.40	29.39	29.36	29.33	29.29	29.25	29.20	29.14	29.07
	29.42	29.45	29.47	29.48	29.48	29.46	29.45	29.42	29.39	29.35	29.31	29.27	29.22	29.17
Cu_8 ring, pz-4-*H*	29.71	29.76	29.77	29.77	29.76	29.74	29.72	29.68	29.64	29.59	29.53	29.47	29.40	29.30
	–	29.57	29.59	29.61	29.62	29.61	29.60	29.57	29.53	29.49	29.44	29.40	29.34	29.24
	30.16	30.18	30.19	30.19	30.19	30.16	30.12	30.08	30.04	29.99	29.93	29.85	29.79	29.72
	–	~26.1	~26.1	26.15	26.15	26.14	26.13	26.11	26.08	26.04	26.00	25.95	25.89	25.82
	26.14	26.16	26.18	26.20	26.20	26.19	26.18	26.16	26.13	26.09	26.05	26.04	26.03	26.01
Cu_8 ring, pz-3,5-H_2	26.45	26.47	26.48	26.49	26.48	26.47	26.45	26.42	26.38	26.33	26.27	26.21	26.15	26.10
	–	26.37	26.41	26.43	26.42	26.42	26.40	26.37	26.34	26.29	26.24	26.20	26.14	26.05
	26.83	26.86	26.88	26.88	26.88	26.86	26.83	26.78	26.74	26.69	26.63	26.57	26.51	26.44
	28.29	28.34	28.41	28.46	28.49	28.52	28.54	28.54	28.54	28.52	28.49	28.46	28.41	28.36
	28.30	28.35	28.41	28.46	28.49	28.52	28.53	28.54	28.53	28.52	28.50	28.47	28.43	28.39
Cu_{14} ring, pz-4-*H*	28.45	28.47	28.53	28.58	28.62	28.64	28.65	28.65	28.64	28.62	28.59	28.55	28.51	28.46
	28.58	28.63	28.69	28.73	28.76	28.78	28.79	28.78	28.77	28.74	28.71	28.66	28.61	28.55
	28.73	28.76	28.80	28.85	28.88	28.89	28.91	28.89	28.88	28.86	28.83	28.78	28.73	28.67
	22.60	22.62	22.64	22.65	22.66	22.66	22.65	22.63	22.61	22.58	22.54	22.50	22.45	22.39
	22.60	22.62	22.64	22.65	22.66	22.66	22.65	22.64	22.61	22.59	22.56	22.52	22.48	22.43
Cu_{14} ring, pz-3,5-H_2	22.69	22.70	22.72	22.73	22.74	22.73	22.72	22.71	22.68	22.65	22.61	22.56	22.52	22.46
	22.78	22.80	22.82	22.83	22.84	22.83	22.82	22.80	22.77	22.73	22.69	22.64	22.59	22.52
	22.91	22.93	22.94	22.94	22.95	22.94	22.92	22.91	22.88	22.85	22.80	22.76	22.71	22.66
	–	–	–	–	−27.17	−27.09	−27.00	−26.90	−26.79	−26.66	−26.52	−26.38	−26.22	−26.05
	–	~−27.2	−27.17	−27.14	−27.06	−27.00	−26.93	−26.84	−26.75	−26.64	−26.53	−26.41	−26.23	−26.08
Cu_{14} ring, O*H*	~−28.1	~−27.5	~−27.4	−27.37	−27.33	−27.28	−27.22	−27.14	−27.05	−26.95	−26.84	−26.72	−26.59	−26.44
	–	–	−27.55	−27.48	−27.42	−27.35	−27.28	−27.19	−27.09	−26.97	−26.84	−26.69	−26.55	−26.40
	−28.28	−28.02	−27.93	−27.95	−27.81	−27.76	−27.72	−27.63	−27.53	−27.43	−27.33	−27.23	−27.09	−26.97
	–	–	–	–	~−35.0	−34.94	~−34.8	−34.73	−34.70	−34.65	−34.59	−34.53	−34.47	−34.40
	–	–	–	–	–	~−34.9	~−34.8	−34.76	−34.70	−34.62	−34.55	−34.49	−34.38	−34.29
Cu_8 ring, O*H*	–	–	–	–	–	~−35.3	−35.23	−35.14	−35.04	−34.95	−34.85	−34.73	−34.63	~−34.6
	–	–	~−36.6	−36.52	−36.47	−36.41	−36.31	−36.20	−36.09	−35.97	−35.91	−35.78	−35.65	−35.56
	–	–	−36.50	−35.92	−35.72	−35.67	−35.61	−35.53	−35.47	−35.37	−35.27	−35.20	−35.06	−35.00
	–	–	–	–	−40.21	−39.72	−39.25	−38.82	−38.40	−37.99	−37.60	−37.24	−36.88	−36.52
	–	–	–	−39.88	−39.42	−39.01	−38.58	−38.20	−37.85	−37.51	−37.16	−36.84	−36.48	−36.16
Cu_9 ring, O*H*	–	–	–	–	–	~−37.3	−37.00	−36.64	−36.24	−35.90	−35.58	−35.26	−34.94	~−34.6
	–	–	–	–	−39.13	−38.83	−38.28	−37.81	−37.37	−36.96	−36.80	−36.44	−36.07	−35.68
	–	–	−41.73	−40.19	−39.47	−38.89	−38.36	−37.85	−37.37	−36.92	−36.53	−36.16	−35.81	−35.41

Table S26 (continued).

NANOJAR	22 °C	30 °C	40 °C	50 °C	60 °C	70 °C	80 °C	90 °C	100 °C	110 °C	120 °C	130 °C	140 °C	150 °C
**Cu_{32} (8+14+10)**														
Cu_{10} ring, pz-4-*H*	–	–	–	–	–	~31.9	31.74	31.59	31.43	31.27	31.10	30.94	30.77	30.61
	–	–	–	–	–	–	~31.8	~31.65	31.50	31.35	31.19	31.02	30.87	30.70
	–	–	–	–	–	–	–	–	–	–	–	–	–	–
	–	–	–	~31.06	~30.90	~30.74	~30.58	~30.41	~30.24	~30.07	~29.90	~29.75	~29.60	~29.44
	39.69	39.52	39.12	38.73	38.33	37.93	~37.5	–	–	–	–	–	–	–
Cu_{10} ring, pz-3,5-H_2	–	–	–	–	~28.1	~28.0	27.86	27.72	27.57	27.43	27.28	27.13	26.97	26.82
	–	–	–	–	–	–	~27.9	27.79	27.64	27.50	27.35	27.20	27.06	26.90
	–	–	–	–	–	–	–	–	–	–	–	–	–	–
	–	–	–	27.32	27.17	27.03	26.89	26.76	26.62	26.48	26.34	~26.19	~26.08	~25.98
	33.85	33.70	33.35	33.02	32.65	–	–	–	–	–	–	–	–	–
Cu_8 ring, pz-4-*H*	–	–	–	–	–	31.45	31.32	31.21	31.09	30.97	30.85	30.71	30.58	30.45
	–	–	–	–	–	–	~31.4	31.25	31.13	31.00	30.88	30.75	30.63	30.48
	–	–	–	–	–	–	–	–	–	–	–	–	–	–
	–	–	–	~29.62	~29.62	~29.62	~29.60	~29.57	~29.52	~29.45	~29.38	~29.32	~29.24	~29.18
	35.82	35.72	35.37	35.23	34.97	34.70	34.43	34.16	33.91	33.66	33.40	33.15	~32.92	–
Cu_8 ring, pz-3,5-H_2	–	–	–	–	~28.1	~28.0	27.93	27.83	27.74	27.64	27.54	27.43	27.31	27.19
	–	–	–	–	–	–	~28.0	27.86	27.76	27.66	27.56	27.46	27.35	27.22
	–	–	–	–	–	–	–	–	–	–	–	–	–	–
	–	~26.1	26.11	26.14	26.14	26.13	26.10	26.08	26.03	25.97	25.91	25.84	25.78	25.72
	31.41	31.32	31.11	30.92	30.71	30.48	30.27	~30.0	29.82	29.62	29.41	29.20	~29.0	–
Cu_{14} ring, pz-4-*H*	–	–	–	–	~28.4	28.41	28.44	28.47	28.48	28.48	~28.5	28.46	~28.4	28.39
	–	–	–	–	–	–	~28.4	28.45	28.47	28.47	28.47	~28.46	~28.43	~28.40
	–	–	–	–	–	–	–	–	–	–	–	–	–	–
	27.94	27.99	28.06	28.11	28.16	28.19	28.21	28.22	28.22	28.21	28.19	28.16	28.13	28.09
	29.15	29.18	29.21	29.23	29.24	29.24	29.24	29.21	29.18	29.15	29.11	29.06	~29.0	–
Cu_{14} ring, pz-3,5-H_2	–	–	–	–	22.57	22.58	22.59	22.59	22.58	22.57	22.54	22.51	22.47	22.43
	–	–	–	–	–	22.59	22.59	~22.60	~22.59	~22.57	~22.54	~22.51	~22.48	~22.44
	–	–	–	–	–	–	–	–	–	–	–	–	–	–
	22.36	22.39	22.41	22.43	22.45	22.45	22.45	22.44	22.42	22.39	22.36	22.33	22.29	22.24
	23.41	23.43	23.44	23.45	23.45	23.44	23.43	23.40	23.37	23.34	23.29	23.24	23.20	23.14
Cu_{14} ring, O*H*	–	–	–	–	–	~−27.7	−27.90	~−28.1	~−28.3	−28.44	−28.48	−28.44	−28.38	−28.30
	–	–	–	–	–	–	~−29.2	~−29.0	~−28.8	~−28.7	~−28.6	−28.49	−28.41	−28.33
	–	–	–	–	–	–	–	–	–	–	–	–	–	–
	–	–	–	~−26.1	−26.04	−25.99	−25.95	−25.90	−25.82	−25.72	−25.61	−25.52	~−25.4	~−25.3
	–	–	–	–	–	–	–	–	–	–	–	–	–	–
Cu_8 ring, O*H*	–	–	–	–	–	~−35.0	~−34.7	−34.27	−33.87	−33.52	−33.15	−32.81	−32.48	−32.14
	–	–	–	–	–	–	~−34.4	~−34.0	−33.59	−33.19	−32.84	−32.51	−32.17	−31.86
	–	–	–	–	–	–	–	–	–	–	–	–	–	–
	–	–	–	–	~−33.0	−32.92	−32.84	−32.74	−32.62	−32.48	−32.32	~−32.2	~−32.1	~−32.0
	−36.07	−35.99	−35.96	−35.84	−35.76	−35.64	~−35.5	–	–	–	–	–	–	–
Cu_{10} ring, O*H*	–	–	–	–	–	–	–	–	–	–	–	–	–	–
	–	–	–	–	–	–	–	–	–	–	–	–	–	–
	–	–	–	–	–	–	–	–	–	–	–	–	–	–
	–	–	–	–	~−41.6	−40.89	−40.20	−39.54	−38.89	−38.26	−37.63	~−37.1	~−36.5	~−35.9
	−42.21	−41.99	−41.73	−41.46	−41.09	−40.74	−40.39	~−40.0	~−39.6	~−39.2	~−38.8	~−38.4	~−38.1	~−37.7

Table S26 (continued).

NANOJAR	22 °C	30 °C	40 °C	50 °C	60 °C	70 °C	80 °C	90 °C	100 °C	110 °C	120 °C	130 °C	140 °C	150 °C
**Cu_{32} (9+14+9)**														
Cu_9 ring, pz-4-*H*	32.46	32.39	32.28	32.15	32.01	31.86	31.70	~31.5	31.35	–	–	–	–	–
	32.68	32.59	32.47	32.33	32.18	32.02	31.85	31.68	31.50	~31.3	~31.1	~30.9	~30.7	~30.5
	–	–	–	–	–	–	–	–	–	–	–	–	–	–
	34.32	34.16	33.95	33.74	33.52	33.30	33.07	32.84	32.61	–	–	–	–	–
	–	–	–	–	–	–	–	–	–	–	–	–	–	–
Cu_9 ring, pz-3,5-H_2	28.85	28.79	28.70	~28.6	28.44	28.34	28.19	28.04	27.88	–	–	–	–	–
	29.06	28.98	28.87	28.75	28.62	28.48	28.33	28.18	28.02	27.85	27.68	~27.5	~27.3	~27.1
	–	–	–	–	–	–	–	–	–	–	–	–	–	–
	30.38	30.24	30.07	29.89	29.70	29.52	29.32	~29.1	~28.9	–	–	–	–	–
	–	–	–	–	–	–	–	–	–	–	–	–	–	–
Cu_{14} ring, pz-4-*H*	28.46	28.51	28.57	28.60	28.63	28.64	28.64	28.63	28.60	28.57	28.52	~28.5	~28.4	~28.4
	28.54	28.59	28.64	28.68	28.69	28.70	28.70	28.68	28.65	28.62	28.57	28.51	~28.4	~28.4
	–	–	–	–	–	–	–	–	–	–	–	–	–	–
	29.12	29.15	29.16	29.17	29.17	29.15	29.13	29.10	29.07	–	–	–	–	–
	–	–	–	–	–	–	–	–	–	–	–	–	–	–
Cu_{14} ring, pz-3,5-H_2	22.95	22.98	23.01	23.02	23.02	23.02	23.00	22.98	22.94	22.90	22.85	22.80	22.73	22.67
	23.02	23.04	23.07	23.08	23.08	23.07	23.06	23.03	22.99	22.95	22.90	22.85	22.80	22.73
	–	–	–	–	–	–	–	–	–	–	–	–	–	–
	23.42	23.43	23.43	23.42	23.41	23.39	23.37	23.34	23.31	–	–	–	–	–
	–	–	–	–	–	–	–	–	–	–	–	–	–	–
Cu_{14} ring, O*H*	−32.02	−32.02	−32.01	−31.98	−31.92	−31.83	−31.73	~−31.6	~−31.4	~−31.2	~−31.0	~−30.8	~−30.6	~−30.4
	~−32.6	~−32.6	−32.58	−32.52	−32.44	−32.32	−32.18	−32.02	−31.84	~−31.6	~−31.4	~−31.2	~−31.0	~−30.8
	–	–	–	–	–	–	–	–	–	–	–	–	–	–
	−35.53	−35.43	−35.30	−35.16	−35.01	−34.84	−34.66	−34.46	–	–	–	–	–	–
	–	–	–	–	–	–	–	–	–	–	–	–	–	–
Cu_9 ring, O*H*	−39.07	−38.85	−38.57	−38.29	−38.01	−37.70	−37.39	−37.09	−36.76	−36.43	−36.09	~−35.7	~−35.4	~−35.0
	−38.55	−38.36	−38.13	−37.89	−37.64	−37.38	−37.12	−36.85	−36.56	−36.26	~−36.0	~−35.6	~−35.3	~−35.0
	–	–	–	–	–	–	–	–	–	–	–	–	–	–
	−39.92	−39.77	−39.57	−39.35	−39.10	−38.83	−38.53	~−38.2	–	–	–	–	–	–
	–	–	–	–	–	–	–	–	–	–	–	–	–	–

Table S26 (continued).

NANOJAR	22 °C	30 °C	40 °C	50 °C	60 °C	70 °C	80 °C	90 °C	100 °C	110 °C	120 °C	130 °C	140 °C	150 °C
**Cu_{30} (7+14+9)**														
Cu_9 ring, pz-4-*H*	–	–	–	–	–	–	–	–	–	–	–	–	–	–
	–	–	–	–	–	–	–	–	–	–	–	–	–	–
	–	–	–	–	–	–	–	–	–	–	–	–	–	–
	37.19	36.88	36.52	36.15	~35.8	~35.4	~35.0	~34.6	–	–	–	–	–	–
	–	–	–	–	–	–	–	–	–	–	–	–	–	–
Cu_9 ring, pz-3,5-H_2	–	–	–	–	–	–	–	–	–	–	–	–	–	–
	–	–	–	–	–	–	–	–	–	–	–	–	–	–
	–	–	–	–	–	–	–	–	–	–	–	–	–	–
	29.25	~29.1	28.95	~28.8	~28.7	28.58	28.53	28.49	–	–	–	–	–	–
	–	–	–	–	–	–	–	–	–	–	–	–	–	–
Cu_7 ring, pz-4-*H*	–	–	–	–	–	–	–	–	–	–	–	–	–	–
	–	–	–	–	–	–	–	–	–	–	–	–	–	–
	–	–	–	–	–	–	–	–	–	–	–	–	–	–
	31.58	31.55	31.50	~31.45	31.38	31.30	31.22	31.14	–	–	–	–	–	–
	–	–	–	–	–	–	–	–	–	–	–	–	–	–
Cu_7 ring, pz-3,5-H_2	–	–	–	–	–	–	–	–	–	–	–	–	–	–
	–	–	–	–	–	–	–	–	–	–	–	–	–	–
	–	–	–	–	–	–	–	–	–	–	–	–	–	–
	27.73	27.78	27.82	27.86	27.89	27.90	27.91	27.91	–	–	–	–	–	–
	–	–	–	–	–	–	–	–	–	–	–	–	–	–
Cu_{14} ring, pz-4-*H*	–	–	–	–	–	–	–	–	–	–	–	–	–	–
	–	–	–	–	–	–	–	–	–	–	–	–	–	–
	–	–	–	–	–	–	–	–	–	–	–	–	–	–
	28.44	28.50	28.56	28.61	28.63	28.65	28.65	28.64	–	–	–	–	–	–
	–	–	–	–	–	–	–	–	–	–	–	–	–	–
Cu_{14} ring, pz-3,5-H_2	–	–	–	–	–	–	–	–	–	–	–	–	–	–
	–	–	–	–	–	–	–	–	–	–	–	–	–	–
	–	–	–	–	–	–	–	–	–	–	–	–	–	–
	22.86	22.90	22.93	22.96	22.97	22.97	22.96	22.95	–	–	–	–	–	–
	–	–	–	–	–	–	–	–	–	–	–	–	–	–
Cu_{14} ring, O*H*	–	–	–	–	–	–	–	–	–	–	–	–	–	–
	–	–	–	–	–	–	–	–	–	–	–	–	–	–
	–	–	–	–	–	–	–	–	–	–	–	–	–	–
	−29.71	−29.72	−29.72	−29.70	−29.72	−29.71	−29.65	~−29.6	–	–	–	–	–	–
	–	–	–	–	–	–	–	–	–	–	–	–	–	–
Cu_7 ring, O*H*	–	–	–	–	–	–	–	–	–	–	–	–	–	–
	–	–	–	–	–	–	–	–	–	–	–	–	–	–
	–	–	–	–	–	–	–	–	–	–	–	–	–	–
	~−41.3	−40.90	~−40.8	−40.66	−40.51	−40.35	−40.19	~−40.0	–	–	–	–	–	–
	–	–	–	–	–	–	–	–	–	–	–	–	–	–
Cu_9 ring, O*H*	–	–	–	–	–	–	–	–	–	–	–	–	–	–
	–	–	–	–	–	–	–	–	–	–	–	–	–	–
	–	–	–	–	–	–	–	–	–	–	–	–	–	–
	~−50.2	~−49.2	~−48.2	−47.34	−46.49	−45.66	−44.84	~−44.0	–	–	–	–	–	–
	–	–	–	–	–	–	–	–	–	–	–	–	–	–

Table S26 (continued).

NANOJAR	22 °C	30 °C	40 °C	50 °C	60 °C	70 °C	80 °C	90 °C	100 °C	110 °C	120 °C	130 °C	140 °C	150 °C
**Cu_{30} (8+14+8)**														
	–	–	–	–	–	–	–	–	–	–	–	–	–	–
	–	–	–	–	–	–	–	–	–	–	–	–	–	–
Cu_8 ring, pz-4-*H*	–	–	–	–	–	–	–	–	–	–	–	–	–	–
	32.59	32.46	32.26	32.07	31.88	31.68	31.49	31.30	–	–	–	–	–	–
	–	–	–	–	–	–	–	–	–	–	–	–	–	–
	–	–	–	–	–	–	–	–	–	–	–	–	–	–
	–	–	–	–	–	–	–	–	–	–	–	–	–	–
Cu_8 ring, pz-3,5-H_2	–	–	–	–	–	–	–	–	–	–	–	–	–	–
	29.35	29.21	29.04	28.88	~28.7	~28.5	~28.3	~28.1	–	–	–	–	–	–
	–	–	–	–	–	–	–	–	–	–	–	–	–	–
	–	–	–	–	–	–	–	–	–	–	–	–	–	–
	–	–	–	–	–	–	–	–	–	–	–	–	–	–
Cu_{14} ring, pz-4-*H*	–	–	–	–	–	–	–	–	–	–	–	–	–	–
	27.02	27.08	27.15	27.20	27.24	27.27	27.29	~27.3	–	–	–	–	–	–
	–	–	–	–	–	–	–	–	–	–	–	–	–	–
	–	–	–	–	–	–	–	–	–	–	–	–	–	–
	–	–	–	–	–	–	–	–	–	–	–	–	–	–
Cu_{14} ring, pz-3,5-H_2	–	–	–	–	–	–	–	–	–	–	–	–	–	–
	22.30	22.33	22.36	22.39	22.40	22.41	22.41	22.42	–	–	–	–	–	–
	–	–	–	–	–	–	–	–	–	–	–	–	–	–
	–	–	–	–	–	–	–	–	–	–	–	–	–	–
	–	–	–	–	–	–	–	–	–	–	–	–	–	–
Cu_{14} ring, O*H*	–	–	–	–	–	–	–	–	–	–	–	–	–	–
	−33.41	−33.41	−33.41	−33.41	−33.39	−33.38	−33.37	−33.36	–	–	–	–	–	–
	–	–	–	–	–	–	–	–	–	–	–	–	–	–
	–	–	–	–	–	–	–	–	–	–	–	–	–	–
	–	–	–	–	–	–	–	–	–	–	–	–	–	–
Cu_8 ring, O*H*	–	–	–	–	–	–	–	–	–	–	–	–	–	–
	−44.56	~−44.0	−43.52	−42.90	−42.34	−41.83	~−41.3	~−40.8	–	–	–	–	–	–
	–	–	–	–	–	–	–	–	–	–	–	–	–	–

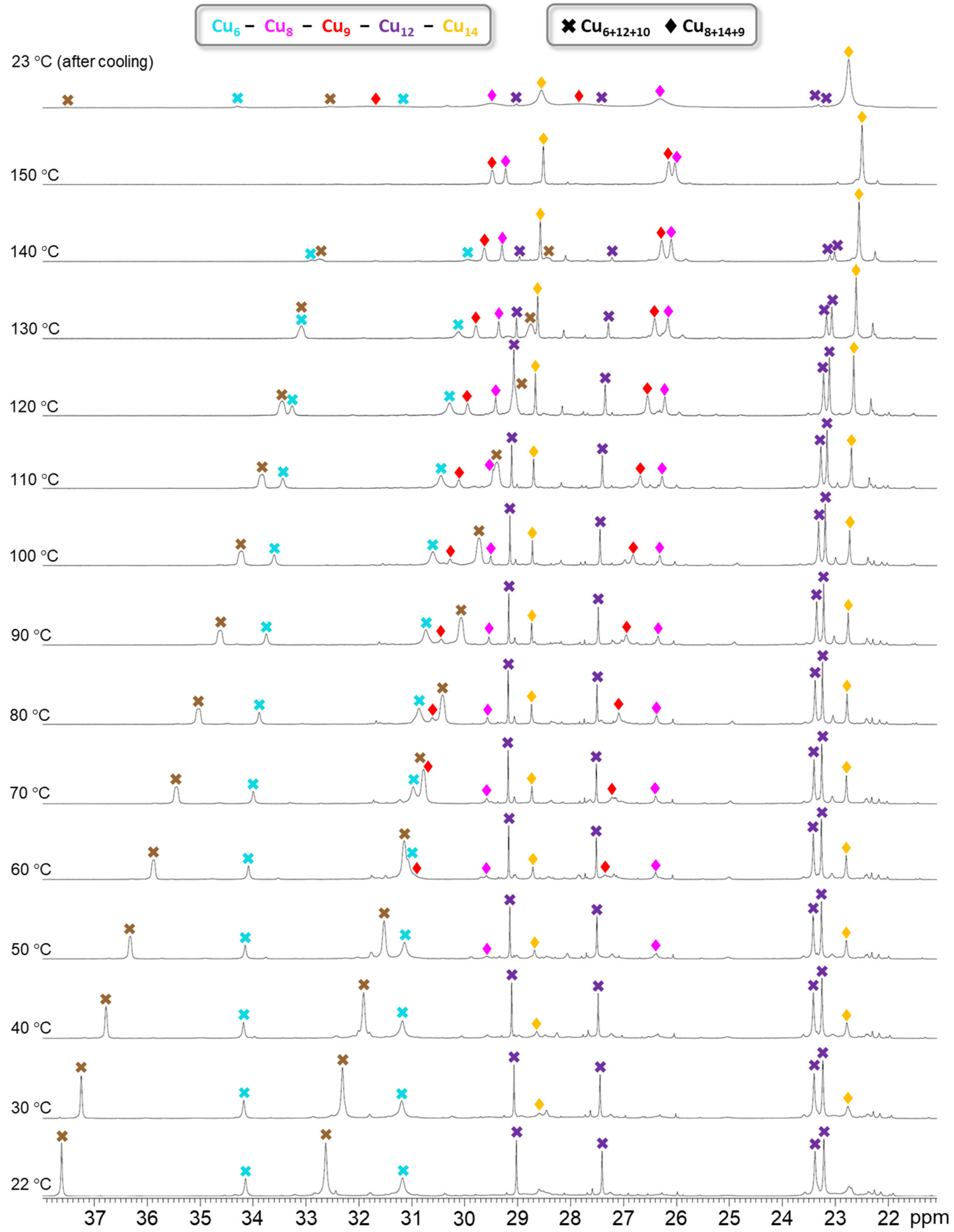

Figure S59. Variable-temperature ^{1}H NMR spectra of the $\mathbf{Cu_{28}SeO_4}$ nanojar in DMSO-d_6, showing pyrazolate proton signals in the 22−38 ppm window. The given temperatures are the target temperatures of the probe.

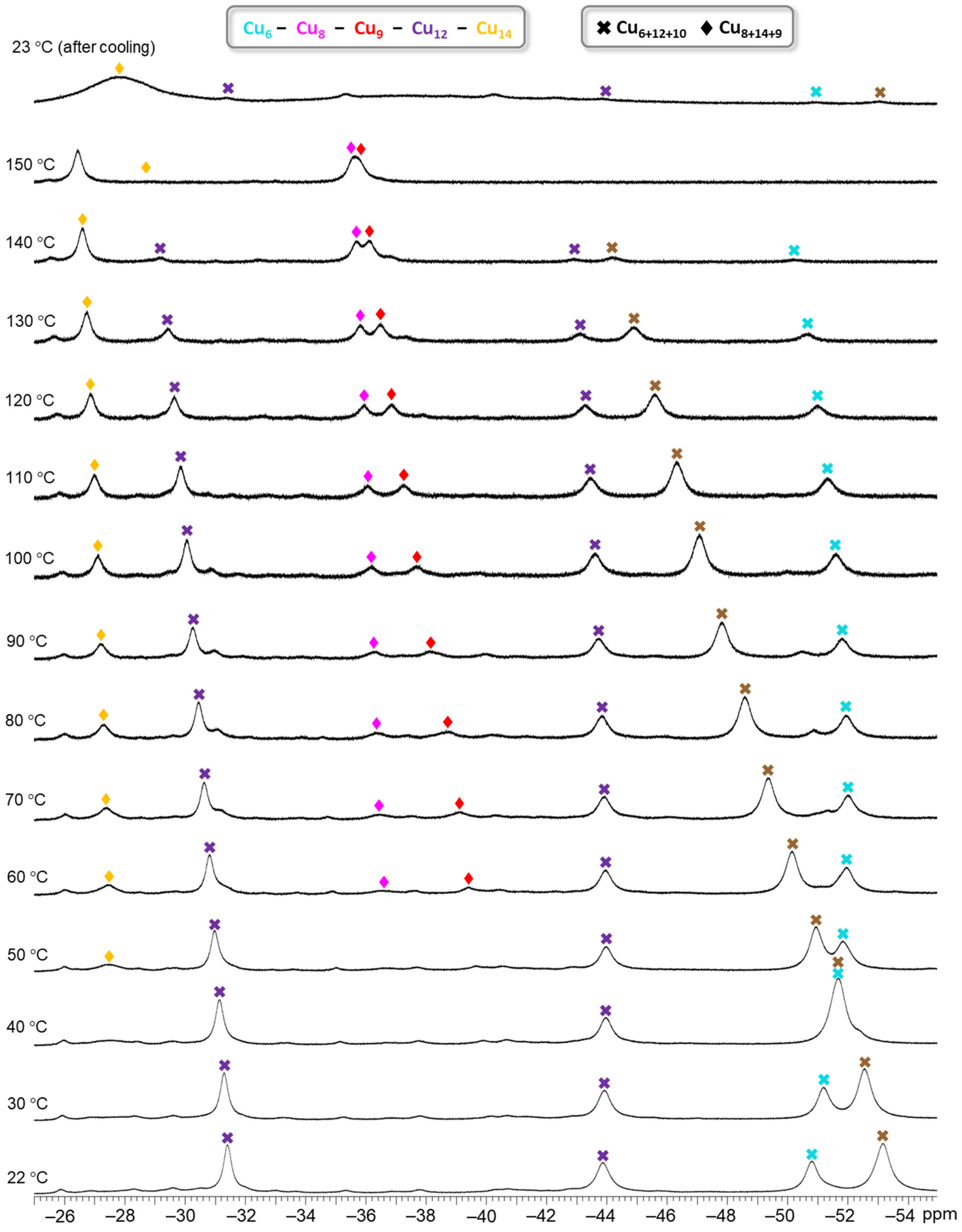

Figure S60. Variable-temperature ${}^{1}H$ NMR spectra of the **$Cu_{28}SeO_4$** nanojar in DMSO-d_6, showing OH proton signals in the −26 to −54 ppm window. The given temperatures are the target temperatures of the probe.

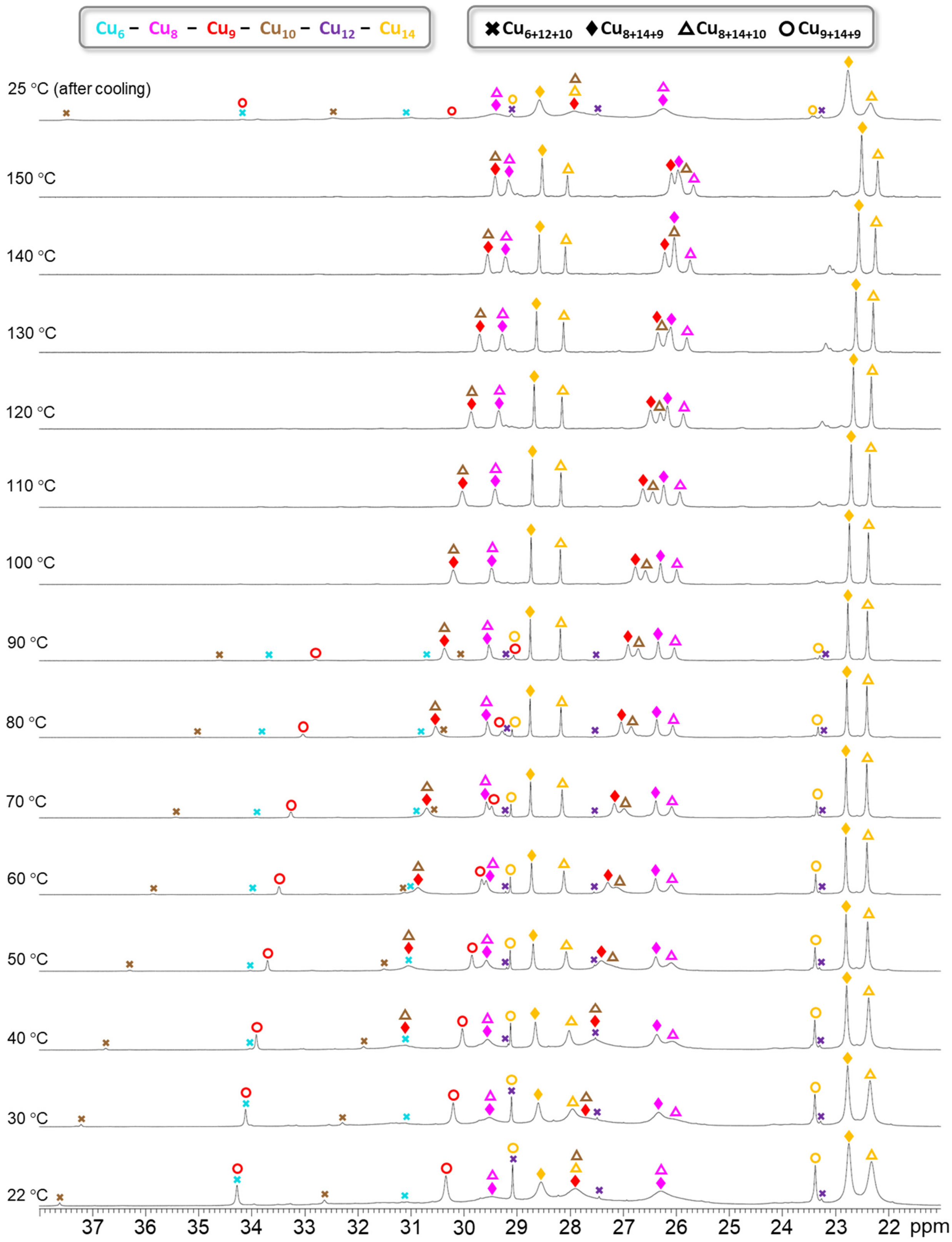

Figure S61. Variable-temperature ^{1}H NMR spectra in DMSO-d_6 of the $\mathbf{Cu_nSeO_4}$ (n = 28, 31, 32) nanojar mixture obtained by depolymerization in refluxing chlorobenzene, showing pyrazolate proton signals in the 22−38 ppm window. The given temperatures are the target temperatures of the probe.

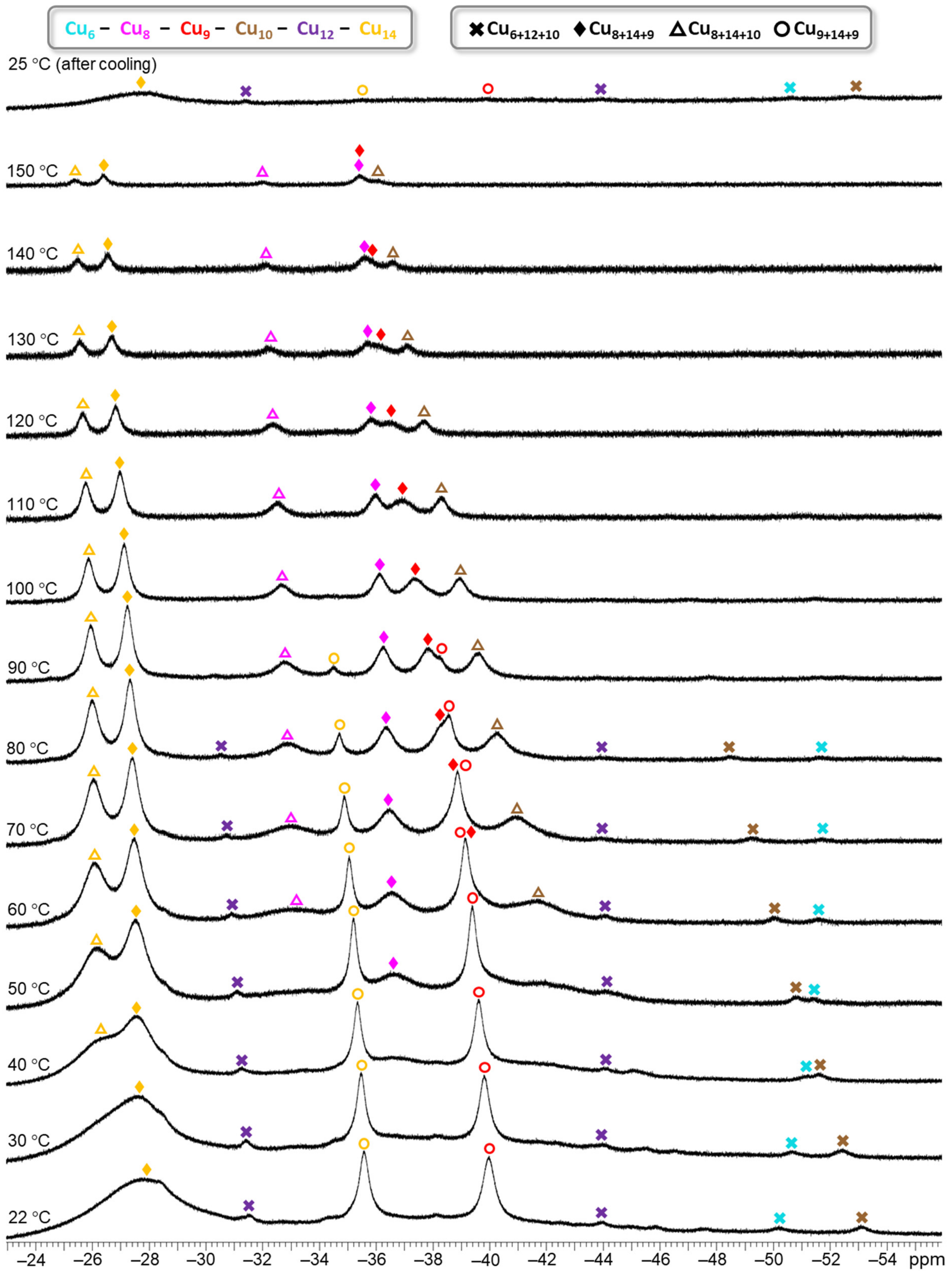

Figure S62. Variable-temperature ^{1}H NMR spectra in DMSO-d_6 of the $\mathbf{Cu_nSeO_4}$ (n = 28, 31, 32) nanojar mixture obtained by depolymerization in refluxing chlorobenzene, showing OH proton signals in the −24 to −54 ppm window. The given temperatures are the target temperatures of the probe.

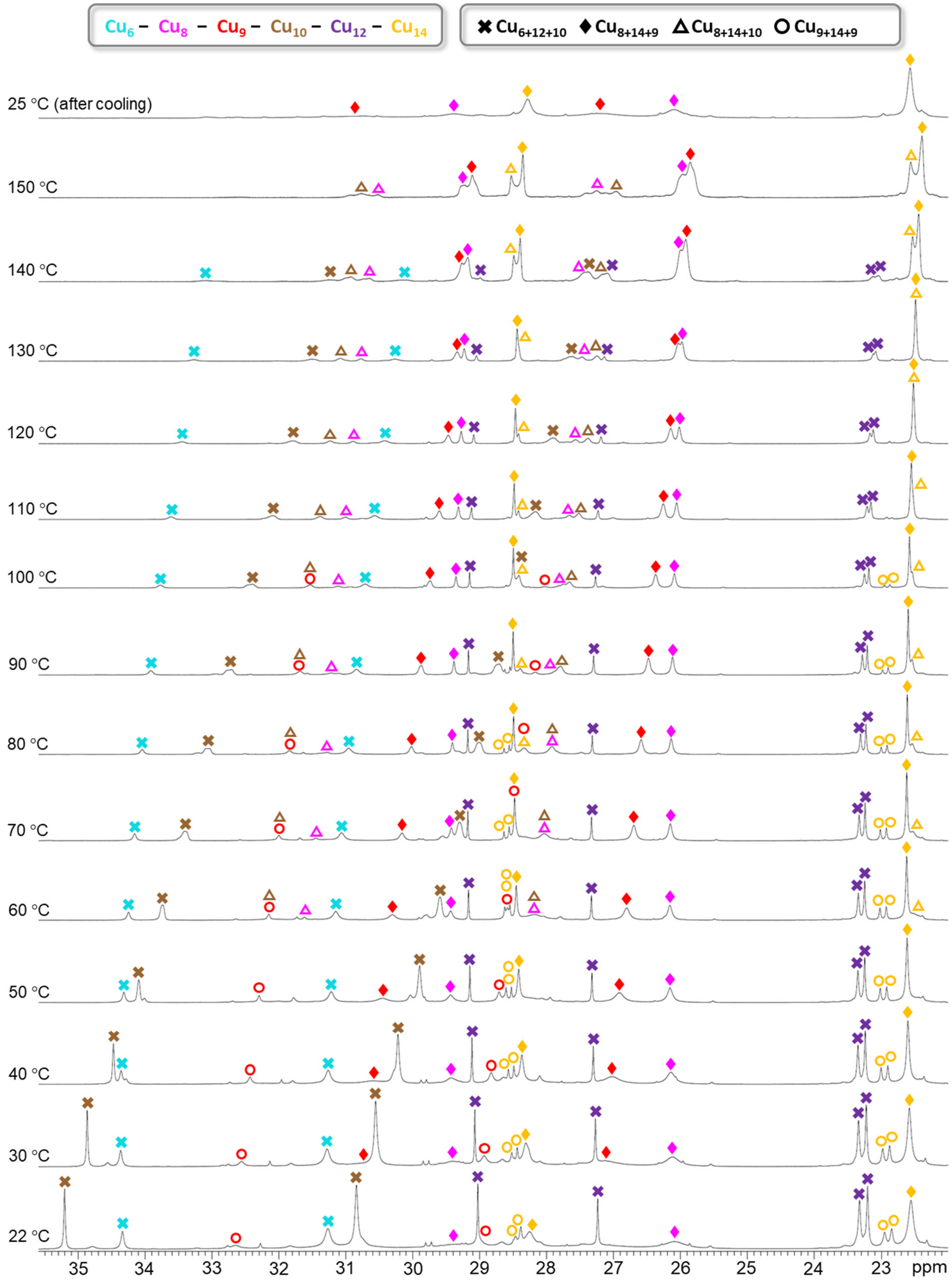

Figure S63. Variable-temperature ¹H NMR spectra of the $\mathbf{Cu_nMoO_4}$ (n = 28–32) nanojar mixture in DMSO-d_6, showing pyrazolate proton signals in the 22−36 ppm window. The given temperatures are the target temperatures of the probe.

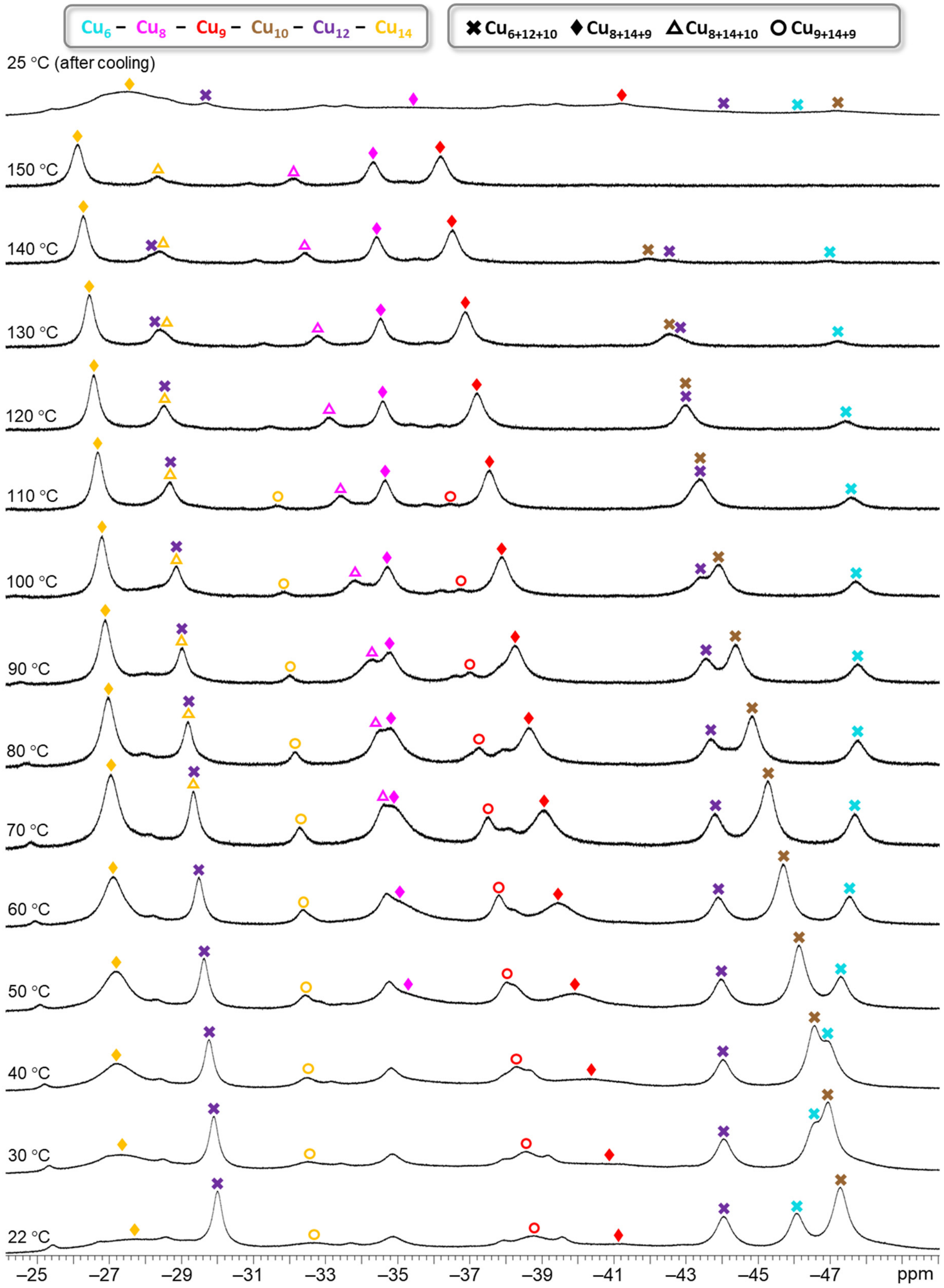

Figure S64. Variable-temperature ^{1}H NMR spectra of the **Cu_nMoO_4** (n = 28–32) nanojar mixture in DMSO-d_6, showing OH proton signals in the −25 to −48 ppm window. The given temperatures are the target temperatures of the probe.

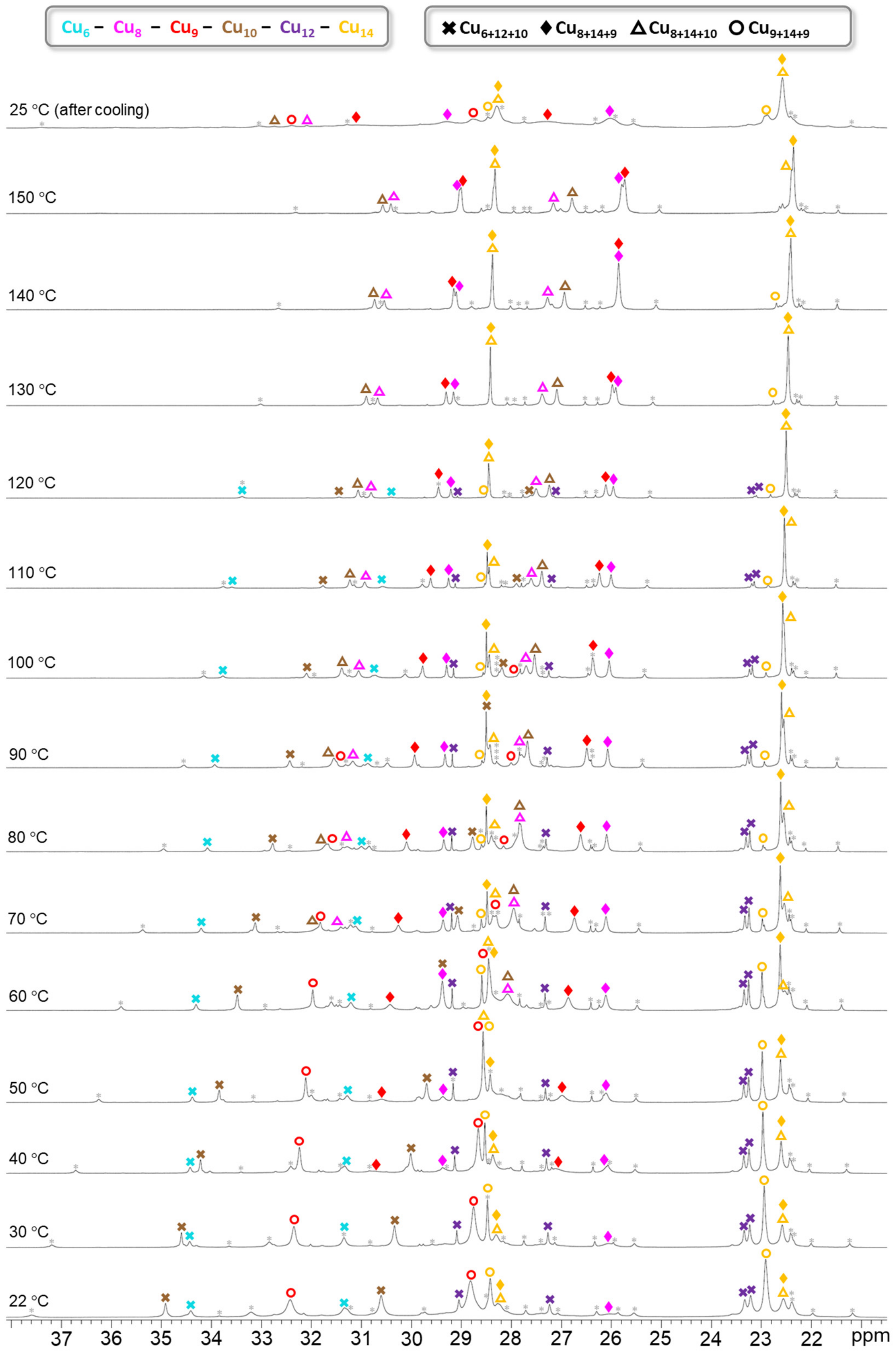

Figure S65. Variable-temperature ¹H NMR spectra of the **Cu_nWO_4** (n = 28–32) nanojar mixture in DMSO-*d_6*, showing pyrazolate proton signals in the 22–36 ppm window. Minor peaks corresponding to carbonate nanojars (**$Cu_{6+12+9}CO_3$**, **$Cu_{7+13+9}CO_3$** and **$Cu_{8+13+8}CO_3$**) are designated with an asterisc. The given temperatures are the target temperatures of the probe.

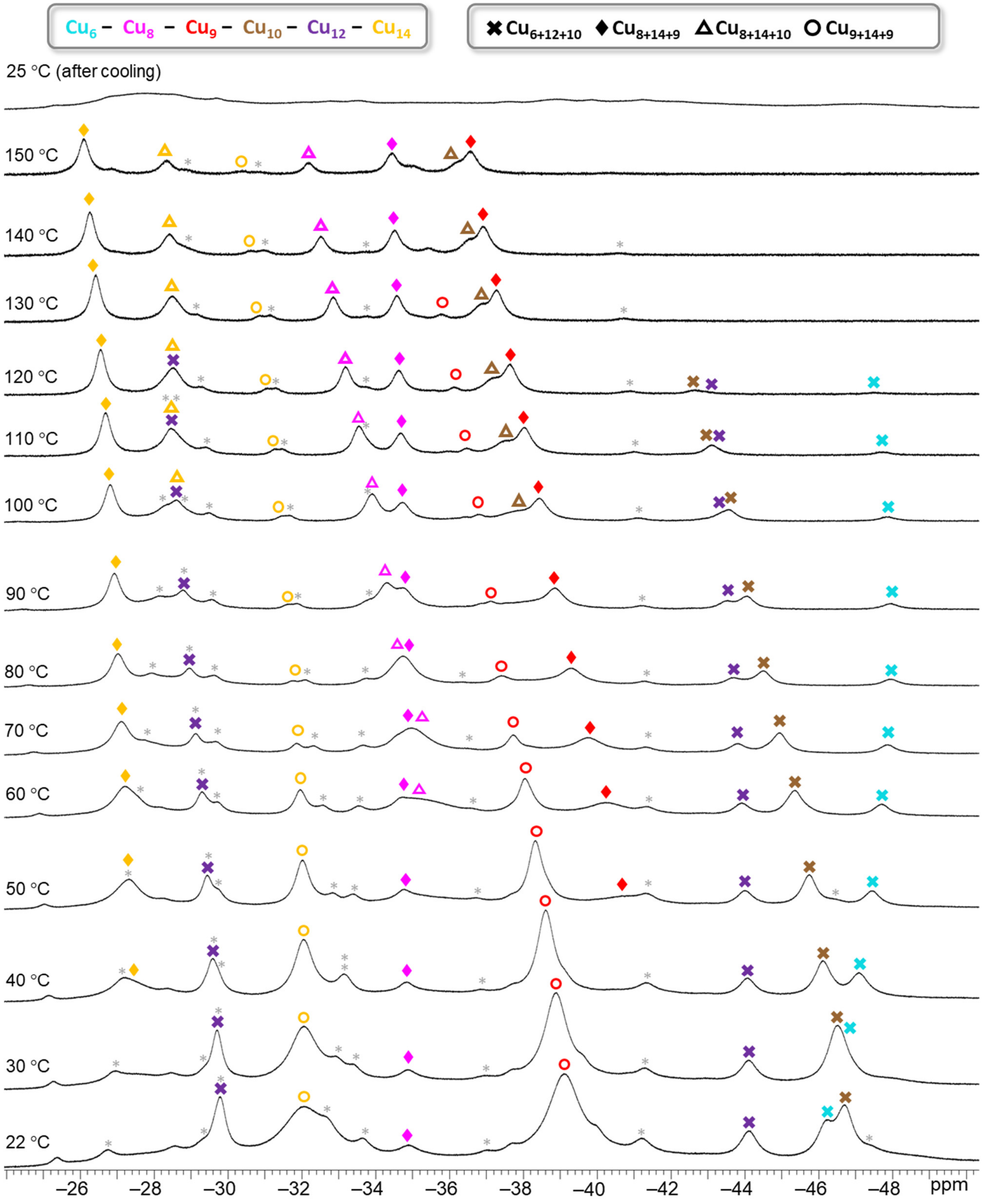

Figure S66. Variable-temperature ^{1}H NMR spectra of the **Cu_nWO_4** (n = 28–32) nanojar mixture in DMSO-d_6, showing OH proton signals in the −25 to −49 ppm window. Minor peaks corresponding to carbonate nanojars (**$Cu_{6+12+9}CO_3$**, **$Cu_{7+13+9}CO_3$** and **$Cu_{8+13+8}CO_3$**) are designated with an asterisc. The given temperatures are the target temperatures of the probe.

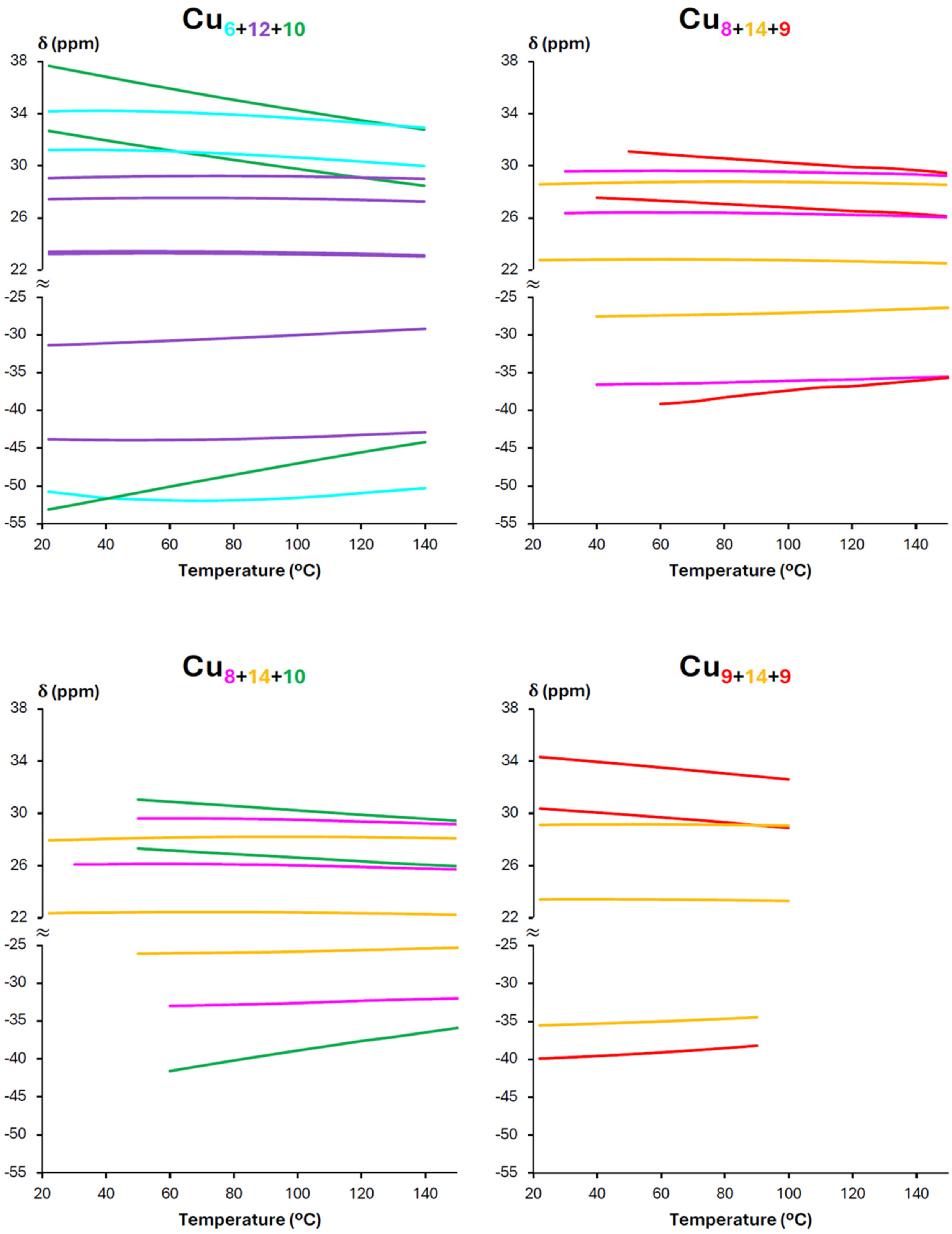

Figure S67. Temperature-dependent variation of the chemical shifts (δ) in DMSO-d_6 of different Cu_x ring protons in various $\mathbf{Cu_nSeO_4}$ nanojars.

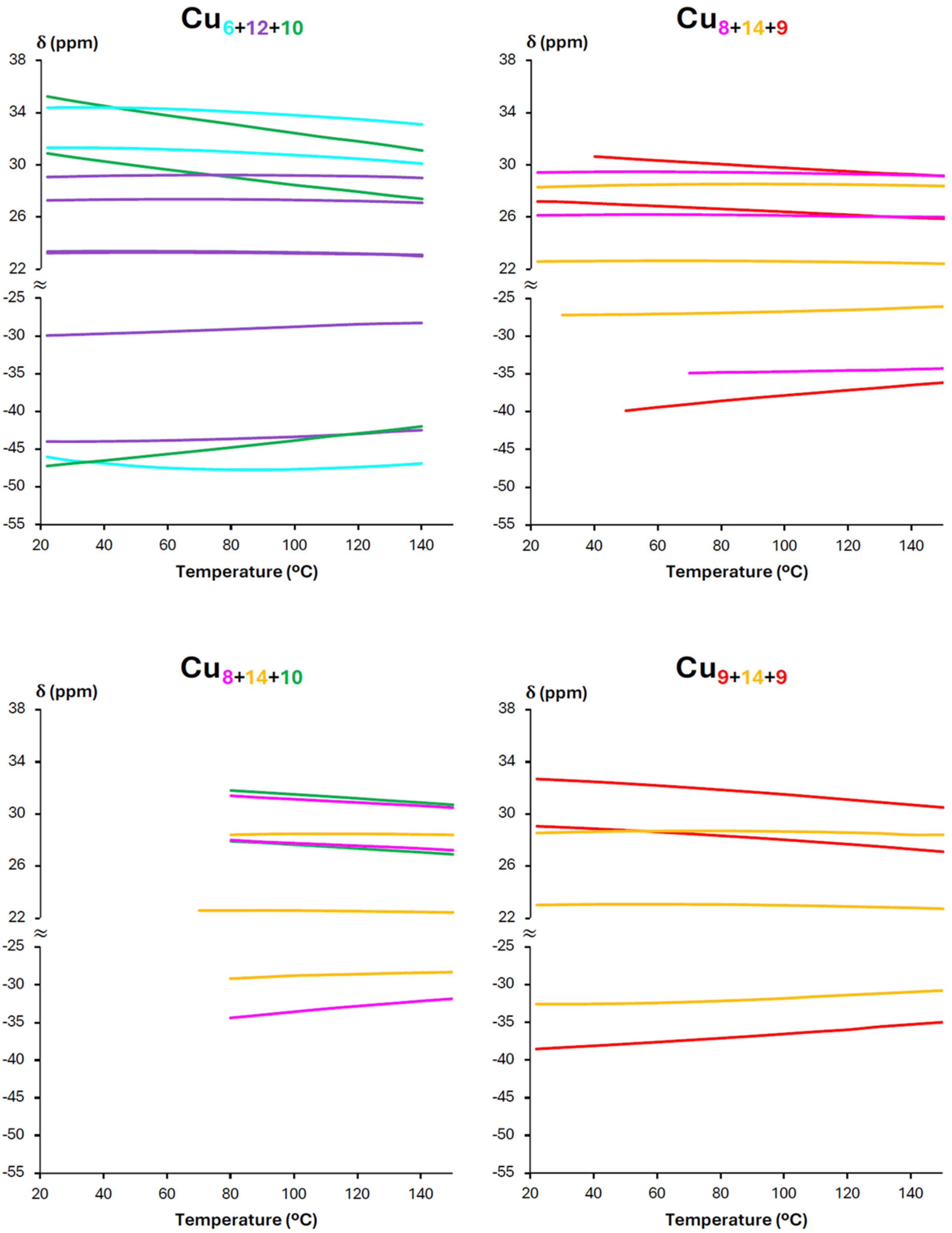

Figure S68. Temperature-dependent variation of the chemical shifts (δ) in DMSO-d_6 of different Cu_x ring protons in various $\mathbf{Cu_nMoO_4}$ nanojars.

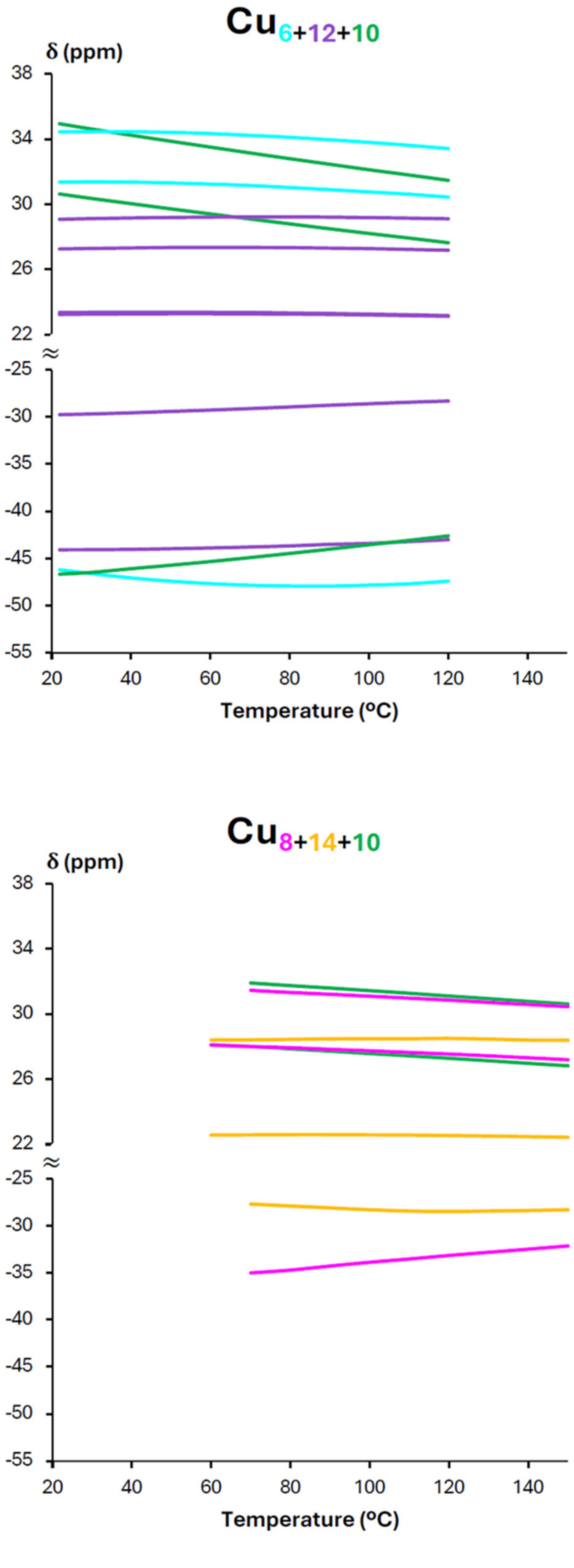

Figure S69. Temperature-dependent variation of the chemical shifts (δ) in DMSO-d_6 of different Cu_x ring protons in various $\mathbf{Cu_nWO_4}$ nanojars.

5. REFERENCES

[1] Ahmed, B. M.; Szymczyna, B. R.; Jianrattanasawat, S.; Surmann, S. A.; Mezei, G. Survival of the Fittest Nanojar: Stepwise Breakdown of Polydisperse Cu_{27}–Cu_{31} Nanojar Mixtures into Monodisperse $Cu_{27}(CO_3)$ and $Cu_{31}(SO_4)$ Nanojars. *Chem. Eur. J*. **2016**, *22*, 5499–5503.

[2] Ahmed, B. M.; Hartman, C. K.; Mezei, G. Sulfate-Incarcerating Nanojars: Solution and Solid-State Studies, Sulfate Extraction from Water, and Anion Exchange with Carbonate. *Inorg. Chem*. **2016**, *55*, 10666–10679.

[3] Al Isawi, W. A.; Zeller, M.; Mezei, G. Capped Nanojars: Synthesis, Solution and Solid-State Characterization, and Atmospheric CO_2 Sequestration by Selective Binding of Carbonate. *Inorg. Chem*. **2021**, *60*, 13479–13492.

[4] Sheldrick, G. M. TWINABS. Ver. 2012/1. Bruker, Madison, Wisconsin, 2012.

[5] van der Sluis, P.; Spek, A. L. BYPASS: An Effective Method for the Refinement of Crystal Structures Containing Disordered Solvent Regions. *Acta Crystallogr., Sect. A: Found. Crystallogr.* **1990**, *46*, 194–201.
